# Access to Carbonyl Azides via Iodine(III)-Mediated Cross-Coupling

**DOI:** 10.1021/acs.orglett.4c03212

**Published:** 2024-10-17

**Authors:** Qing Yan, Lanlan Lv, Li Xu, Elena V. Stepanova, Gregory R. Alvey, Andrey Shatskiy, Markus D. Kärkäs, Xiang-Shan Wang

**Affiliations:** † School of Chemistry and Materials Science, Jiangsu Key Laboratory of Green Synthesis for Functional Materials, Jiangsu Normal University, Xuzhou, Jiangsu 221116, China; ‡ Department of Chemistry, KTH Royal Institute of Technology, SE-100 44 Stockholm, Sweden; ∥ Research School of Chemistry & Applied Biomedical Sciences, Tomsk Polytechnic University, 634050 Tomsk, Russia

## Abstract

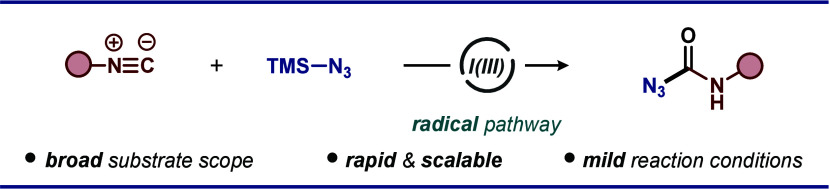

Herein, we present a prominent metal-free C–N cross-coupling platform that enables access to carbamoyl- and ketoazides from isocyanides or silyl enol ethers and trimethylsilyl azide (TMSN_3_) with an aid of iodine(III) promoter. This offers a rapid route to a diverse set of synthetically valuable azide decorated fragments with excellent substrate scope and good to excellent yields. The disclosed platform exemplifies the use of TMSN_3_ for incorporation of the azide fragment without the loss of N_2_.

The construction of carbon–nitrogen (C–N) bonds occupies a pivotal role in modern organic synthesis, serving as the primary route for the synthesis of nitrogen-containing compounds.^1,2^ These compounds have diverse applications as pharmaceuticals, natural products, and functional materials, and exhibit significant biological and pharmacological activities.^3,4^ Despite significant progress, there is a continued need for innovative C–N coupling technologies applicable to a wide variety of coupling partners. Isocyanides are versatile building blocks in chemical synthesis that demonstrate carbene-like reactivity similar to carbon monoxide, making them suitable for realizing numerous reaction manifolds.^5^ Beside the consistent prosperity of isocyanides in multicomponent cross-couplings and C–H functionalization manifolds, isocyanides have also been extensively explored in free-radical settings.^6,7^ While these technologies are primarily focused on the formation of C–C bonds, it is relatively rare for isocyanides to engage in C–N bond formation. Hitherto, only three types of such reactions have been established, including: (1) copper-catalyzed insertion of isocyanides into amines,^8^ (2) transition-metal-catalyzed cross-coupling reactions of azides with isocyanides,^9^ and (3) transition-metal-catalyzed tandem insertion of isocyanides into C–H and N–H bonds for accessing N-heterocycles (Figure 1).^10^ These protocols often impose strict limitations on the compatible substrates and attainable products, highlighting the need for more general C–N cross-coupling platforms.

**Figure 1:**
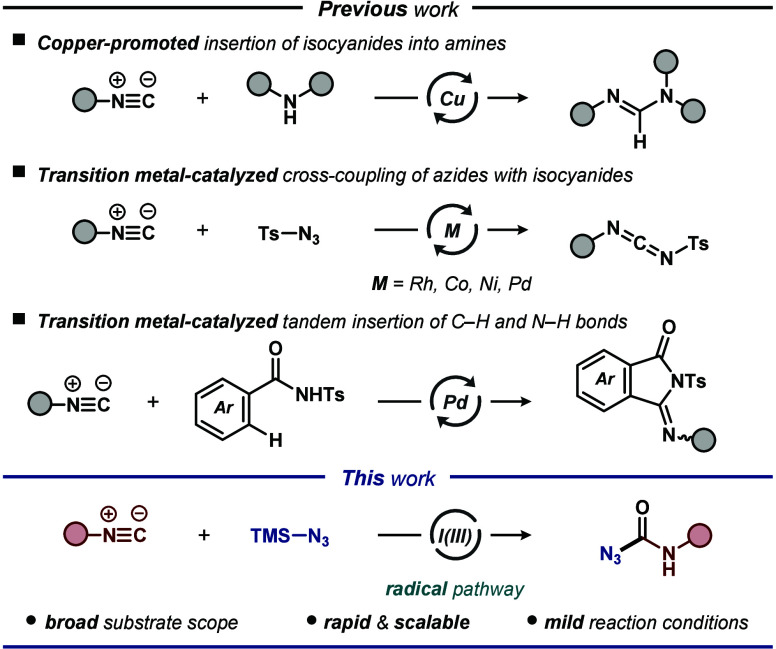
Isocyanide-based cross-coupling technologies for the construction of carbon–nitrogen bonds.

Recently, iodine(III)-promoted radical cross-couplings involving azides have emerged as a powerful tool for C–N bond formation.^11,12^ Although several methodologies have been disclosed for the synthesis of carbamoyl azides,^13,14^ these manifolds are associated with harsh reaction conditions, moderate yields and the use of unstable and/or air sensitive reagents. We recently disclosed a silver-catalyzed protocol for decarboxylative cross-coupling between carboxylic acids and isocyanides leading to linear amide products through a free-radical mechanism.^15^ Inspired by these results and our previous explorations in isocyanide chemistry,^16^ herein we report a transition metal– and base-free, mild, and rapid protocol for radical cross-coupling of TMSN_3_ with various isocyanides and silyl enol ethers, allowing general entry to decorated carbonyl azides (Figure 1). The disclosed approach involves activation of the azide through presumed one-electron oxidation to furnish the corresponding azidyl radical, effectively averting the highly thermodynamically favorable extrusion of N_2_ and enabling C–N_3_ bond formation.

We commenced the search for the envisioned reaction conditions with 4-bromophenylisocyanide (**1a**) and TMSN_3_ (**2**) as the coupling partners, using a range of common oxidants and solvents (Table 1). Chemical oxidants, such as K_2_S_2_O_8_, TBHP, Fe(acac)_3_, Mn(OAc)_3_, PhI(OAc)_2_ and PhI(TFA)_2_, that should be able to promote the formation of the azidyl radical from TMSN_3_, were initially evaluated in MeCN as solvent (Table 1, entries 1–6).^17^ This revealed PhI(OAc)_2_ as the optimal oxidant to give carbamoyl azide product **3a** in 60% yield (Table 1, entry 5). The subsequent screening of solvents (Table 1, entries 7–11) revealed that conducting the reaction in DMSO increased the yield of the desired product **3a** up to 68%. Finally, using a slight excess of azide **2** resulted in a dramatically increased yield of **3a** up to ca. 85% (Table 1, entries 12 and 13). Control experiments conducted in the absence of a chemical oxidant resulted in no product, emphasizing the crucial role of the iodine(III) reagent for the disclosed reaction. Additionally, the structure of product **3a** was unequivocally confirmed by single-crystal X-ray analysis (CCDC 2351700, Scheme 1).

**Scheme 1:**
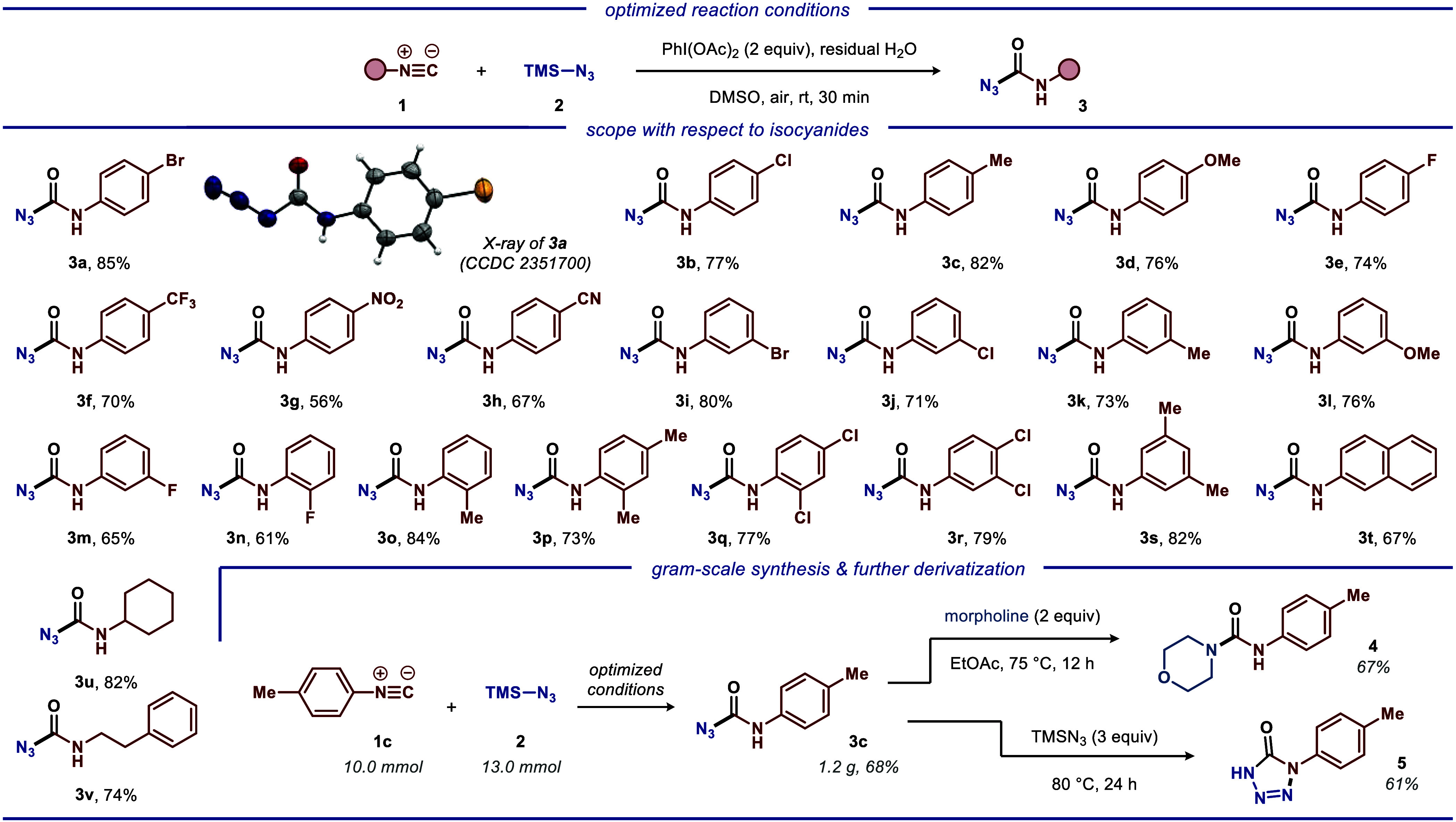
Reaction Scope for the Iodine(III)-Mediated Cross-Coupling for the Synthesis of Carbamoyl Azides Reaction conditions: isocyanide **1** (0.5 mmol, 1.0 equiv), azide **2** (0.65 mmol, 1.3 equiv), PhI(OAc)_2_ (1.0 mmol, 2.0 equiv), DMSO (2.0 mL), ambient temperature, air, 30 min. All yields are of isolated products.

**Table 1:**
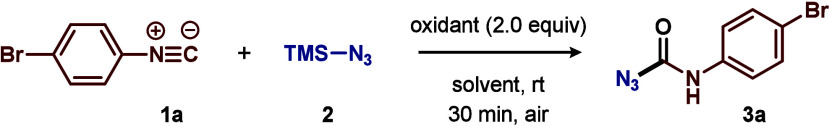
Optimization of the Reaction Conditions^a^

entry	ratio **1a**/**2**	oxidant	solvent	yield (%)^b^
1	1:1	K_2_S_2_O_8_	MeCN	20
2	1:1	TBHP	MeCN	trace
3^c^	1:1	Fe(acac)_3_	MeCN	26
4^d^	1:1	Mn(OAc)_3_	MeCN	31
5	1:1	PhI(OAc)_2_	MeCN	60
6	1:1	PhI(TFA)_2_	MeCN	47
7	1:1	PhI(OAc)_2_	DMSO	68
8	1:1	PhI(OAc)_2_	DMF	27
9	1:1	PhI(OAc)_2_	THF	20
10	1:1	PhI(OAc)_2_	CH_2_Cl_2_	8
11	1:1	PhI(OAc)_2_	DCE	10
12	1:1.3	PhI(OAc)_2_	DMSO	85
13	1:1.5	PhI(OAc)_2_	DMSO	86

a Reaction conditions: Reactions were carried out with **1a** (0.5 mmol), **2** (0.5–0.75 mmol), and oxidant (2 equiv) in solvent (2.0 mL) under air for 30 min.

b Isolated yields of **3a** after purification by column chromatography.

c 30 mol % catalyst.

d 20 mol % catalyst.

With the optimized reaction conditions in hand, we investigated the scope of compatible aryl isocyanide cross-coupling partners (Scheme 1). Aromatic isocyanides featuring a large variety of functional groups, such as F, Cl, Br, Me, OMe, CN, NO_2_ and CF_3_, are readily tolerated, affording the corresponding carbamoyl azides in good to excellent yields (56–85%). Disubstituted aryl isocyanides were also efficiently transformed under the optimized reaction conditions, affording the corresponding products **3p**–**3s** in 79–82% yield. Utilization of fused aromatic **1t** also provided the expected product **3t** in 67% yield. Furthermore, a gram-scale synthesis was carried out under the same conditions employing 10 mmol of isocyanide **1c** to assess the potential for further application of the developed technology. Delightfully, the gram-scale reaction of **1c** and **2** proceeded smoothly, albeit with a slight decrease in the yield of product **3c** (68%). Products **3** provide various opportunities for further synthetic manipulations. For example, product **3c** could be functionalized into the corresponding urea and tetrazole derivatives **4** and **5**, respectively, in good yields by subjection to morpholine or TMSN_3_.^13c,18,19^

Silyl enol ethers display partially related properties to isocyanides and have been widely applied in related cross-coupling reactions, allowing interception with various electrophiles^20^ or free-radical species.^21^ Thereby, various functionalized silyl enol ethers (**6**) were investigated as suitable coupling partners. Gratifyingly, a diverse array of substituted silyl enol ethers **6** could readily participate in the developed radical coupling reaction with TMSN_3_ (**2**), delivering the corresponding ketoazide products **7** in good to excellent yields (55–88%, Scheme 2).

**Scheme 2:**
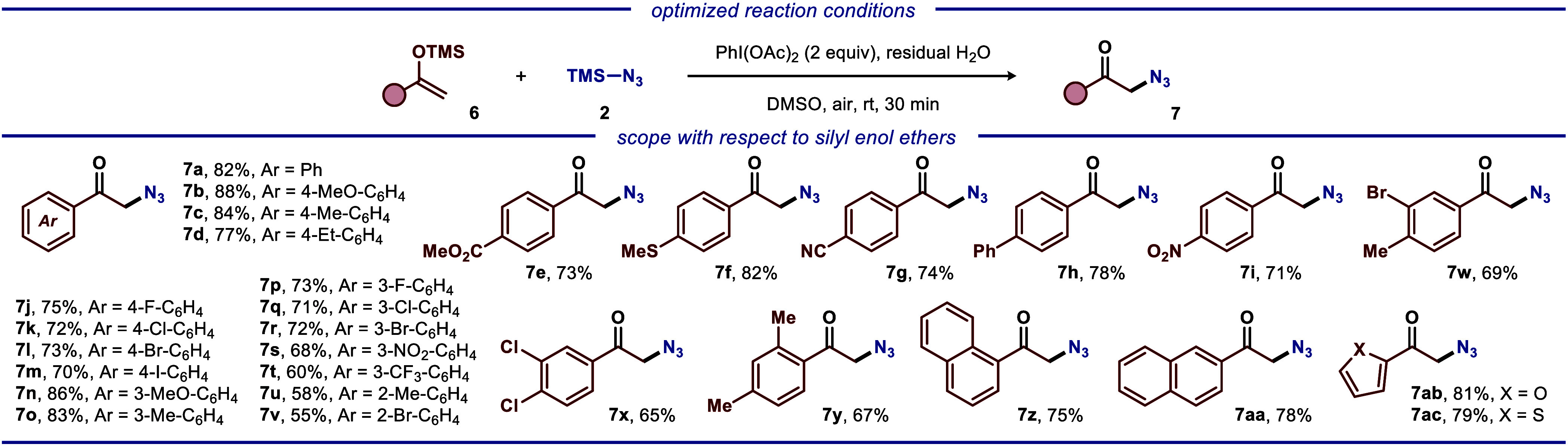
Reaction Scope for the Iodine(III)-Mediated Cross-Coupling for the Synthesis of Ketoazides Reaction conditions: silyl enol ethers **6** (0.5 mmol, 1.0 equiv), azide **2** (0.65 mmol, 1.3 equiv), PhI(OAc)_2_ (1.0 mmol, 2.0 equiv), DMSO (2.0 mL), ambient temperature, air, 30 min. All yields are of isolated products.

Next, several experiments were conducted to gain mechanistic insight (Scheme 3). The reaction between 4-bromophenylisocyanide (**1a**) and TMSN_3_ (**2**) was completely suppressed by the addition of radical inhibitors, such as TEMPO or BHT, thereby suggesting that the reaction proceeds through a free-radical pathway. Furthermore, conducting the reaction with a Zhdankin-type iodine(III)-N_3_ reagent did not provide the desired product **3a** (Figure S3). In the presence of H_2_^18^O, the reaction between **1a** and **2** provides [^18^O]-**3a** in 68%, implying that water is the main oxygen source for the reaction. Based on the results of the mechanistic investigations, relevant literature precedents^6c,16b,17a,17c,22^ and computational studies, a plausible reaction mechanism was proposed (Scheme 3 and Figures S1 and S2). In the initiation step, TMSN_3_ reacts with PhI(OAc)_2_ to form a highly unstable azide-based hypervalent iodine species PhI(N_3_)_2_, which rapidly decomposes at room temperature to release the azidyl radical. Subsequently, the azidyl radical is intercepted by isocyanide **1** to form carbon-centered radical **A**. Finally, the latter undergoes one-electron oxidation to intermediate **B**, which delivers the desired product **3** upon reaction with residual water. Although the mechanistic experiments suggest that a radical pathway is operating, an alternative polar pathway cannot be fully ruled out.

**Scheme 3:**
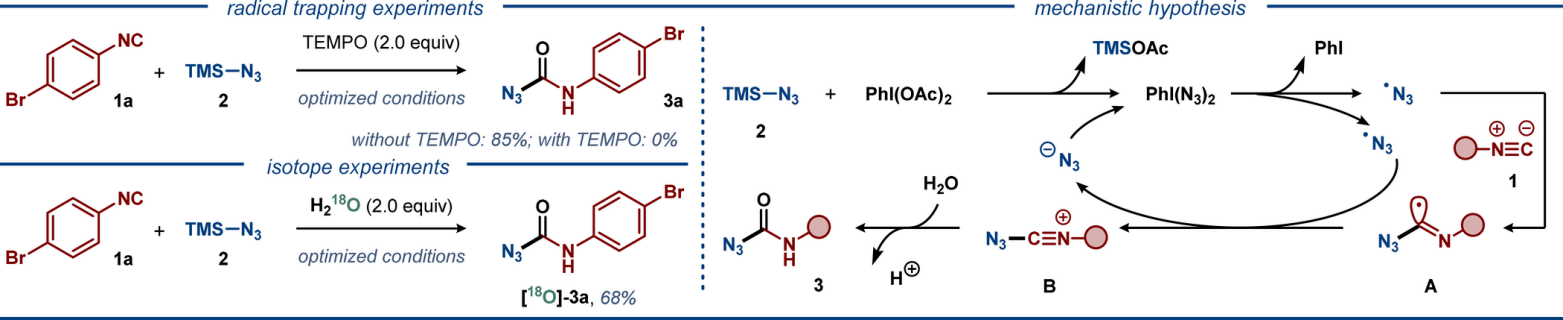
Mechanistic Considerations

In summary, we have developed highly practical metal-free cross-coupling protocols between TMSN_3_ and isocyanides or silyl enol ethers, enabling rapid access to a diverse array of synthetically valuable carbamoyl- and ketoazides. This work represents the first example of a coupling reaction between an isocyanide and azide, proceeding without the loss of N_2_. The disclosed platforms demonstrate excellent substrate scope, delivering the desired azide products in good to excellent yields, and are readily amenable for scale-up. The developed C–N bond coupling technologies relies on simple and readily available building blocks and are expected to gain widespread adoption, aiding the development of novel coupling technologies.

## Data Availability

The data underlying this study are available in the published article and its online Supporting Information.

## Supporting Information Available

Experimental procedures and X-ray crystallography data of **3a** as well as characterization data and copies of NMR spectra for all obtained products (PDF)
FAIR data, including the primary NMR FID files, for compounds **3**, **4**, **5**, and **7** (ZIP)
