# Supplementary material for: Access to Carbonyl Azides via Iodine(III)-Mediated Cross-Coupling
Source: Org Lett. 2024 Oct 17;26(43):9215–20. doi: 10.1021/acs.orglett.4c03212 (PMC11536401; doi:10.1021/acs.orglett.4c03212)
Supplement: Supplementary file 1 — ol4c03212_si_001.pdf [file ol4c03212_si_001.pdf]

Supporting Information

Access to Carbonyl Azides via Iodine(III)-Mediated Cross-Coupling

Qing Yan,[†] Lanlan Lv,[†,*]✉ Li Xu,[†] Elena V. Stepanova,[‡,∥] Gregory R. Alvey,[‡] Andrey Shatskiy,[‡] Markus D. Kärkäs,[‡,*]✉ Xiang-Shan Wang[†,*]✉

[†] School of Chemistry and Materials Science, Jiangsu Key Laboratory of Green Synthesis for Functional Materials, Jiangsu Normal University, Xuzhou, Jiangsu 221116, China

[‡] Department of Chemistry, KTH Royal Institute of Technology, SE-100 44 Stockholm, Sweden

[∥] Research School of Chemistry & Applied Biomedical Sciences, Tomsk Polytechnic University, 634050 Tomsk, Russia

✉ E-mail: lvll800@126.com (L.L.); karkas@kth.se (M.D.K.); xswang@jsnu.edu.cn (X.-S.W)

Table of Contents

I. General information **S1**

II. Crystallography **S1**

III. Synthesis of compounds 1 and 6 .... **S3**

General procedure for the synthesis of isocyanides (**1**) S3

General procedure for the synthesis of aryl silyl enol ethers (**6**) S3

IV. Synthesis and analytical data of compounds 3, 4, 5 and 7 .... **S5**

(4-Bromophenyl)carbamoyl azide (**3a**) S5

(4-Chlorophenyl)carbamoyl azide (**3b**) S5

p-Tolylcarbamoyl azide (**3c**) S6

(4-Methoxyphenyl)carbamoyl azide (**3d**) S6

(4-Fluorophenyl)carbamoyl azide (**3e**) S7

(4-(Trifluoromethyl)phenyl)carbamoyl azide (**3f**) S7

(4-Nitrophenyl)carbamoyl azide (**3g**) S8

(4-Cyanophenyl)carbamoyl azide (**3h**) S8

(3-Bromophenyl)carbamoyl azide (**3i**) S9

(3-Chlorophenyl)carbamoyl azide (**3j**) S9

m-Tolylcarbamoyl azide (**3k**) S10

(3-Methoxyphenyl)carbamoyl azide (**3l**) S10

(3-Fluorophenyl)carbamoyl azide (**3m**) S11

(2-Fluorophenyl)carbamoyl azide (**3n**) S11

o-Tolylcarbamoyl azide (**3o**) S12

(2,4-Dimethylphenyl)carbamoyl azide (**3p**) S12

(2,4-Dichlorophenyl)carbamoyl azide (**3q**) S13

(3,4-Dichlorophenyl)carbamoyl azide (**3r**) S13

(3,5-Dimethylphenyl)carbamoyl azide (**3s**) S14

Naphthalen-2-ylcarbamoyl azide (**3t**) S14

Cyclohexylcarbamoyl azide (**3u**) S15

Phenethylcarbamoyl azide (**3v**) S15

N-(*p*-Tolyl)morpholine-4-carboxamide (**4**) S16

1-(*p*-Tolyl)-1,4-dihydro-5*H*-tetrazol-5-one (**5**) S16
2-Azido-1-phenylethan-1-one (**7a**) S17
2-Azido-1-(4-methoxyphenyl)ethan-1-one (**7b**) S17
2-Azido-1-(p-tolyl)ethan-1-one (**7c**) S18
2-Azido-1-(4-ethylphenyl)ethan-1-one (**7d**) S18
Methyl-4-(2-azidoacetyl)benzoate (**7e**) S19
2-Azido-1-(4-(methylthio)phenyl)ethan-1-one (**7f**) S19
4-(2-Azidoacetyl)benzonitrile (**7g**) S20
1-([1,1'-Biphenyl]-4-yl)-2-azidoethan-1-one (**7h**) S20
2-Azido-1-(4-nitrophenyl)ethan-1-one (**7i**) S21
2-Azido-1-(4-fluorophenyl)ethan-1-one (**7j**) S21
2-Azido-1-(4-chlorophenyl)ethan-1-one (**7k**) S22
2-Azido-1-(4-bromophenyl)ethan-1-one (**7l**) S22
2-Azido-1-(4-iodophenyl)ethan-1-one (**7m**) S23
2-Azido-1-(3-methoxyphenyl)ethan-1-one (**7n**) S23
2-Azido-1-(*m*-tolyl)ethan-1-one (**7o**) S24
2-Azido-1-(3-fluorophenyl)ethan-1-one (**7p**) S24
2-Azido-1-(3-chlorophenyl)ethan-1-one (**7q**) S25
2-Azido-1-(3-bromophenyl)ethan-1-one (**7r**) S25
2-Azido-1-(3-nitrophenyl)ethan-1-one (**7s**) S26
2-Azido-1-(3-nitrophenyl)ethan-1-one (**7t**) S26
2-Azido-1-(*o*-tolyl)ethan-1-one (**7u**) S27
2-Azido-1-(2-bromophenyl)ethan-1-one (**7v**) S27
2-Azido-1-(3-bromo-4-methylphenyl)ethan-1-one (**7w**) S28
2-Azido-1-(3,4-dichlorophenyl)ethan-1-one (**7x**) S28
2-Azido-1-(2,4-dimethylphenyl)ethan-1-one (**7y**) S29
2-Azido-1-(naphthalen-1-yl)ethan-1-one (**7z**) S29
2-Azido-1-(naphthalen-2-yl)ethan-1-one (**7aa**) S30
2-Azido-1-(furan-2-yl)ethan-1-one (**7ab**) S30
2-Azido-1-(thiophen-2-yl)ethan-1-one (**7ac**) S31

V. NMR spectra **S32**
(4-Bromophenyl)carbamoyl azide (**3a**) S32
(4-Chlorophenyl)carbamoyl azide (**3b**) S33
p-Tolylcarbamoyl azide (**3c**) S34
(4-Methoxyphenyl)carbamoyl azide (**3d**) S35
(4-Fluorophenyl)carbamoyl azide (**3e**) S36
(4-(Trifluoromethyl)phenyl)carbamoyl azide (**3f**) S37
(4-Nitrophenyl)carbamoyl azide (**3g**) S38
(4-Cyanophenyl)carbamoyl azide (**3h**) S39
(3-Bromophenyl)carbamoyl azide (**3i**) S40
(3-Chlorophenyl)carbamoyl azide (**3j**) S41
m-Tolylcarbamoyl azide (**3k**) S42
(3-Methoxyphenyl)carbamoyl azide (**3l**) S43
(3-Fluorophenyl)carbamoyl azide (**3m**) S44
(2-Fluorophenyl)carbamoyl azide (**3n**) S45
o-Tolylcarbamoyl azide (**3o**) S46
(2,4-Dimethylphenyl)carbamoyl azide (**3p**) S47
(2,4-Dichlorophenyl)carbamoyl azide (**3q**) S48
(3,4-Dichlorophenyl)carbamoyl azide (**3r**) S49
(3,5-Dimethylphenyl)carbamoyl azide (**3s**) S50
Naphthalen-2-ylcarbamoyl azide (**3t**) S51
Cyclohexylcarbamoyl azide (**3u**) S52
Phenethylcarbamoyl azide (**3v**) S53
N-(*p*-Tolyl)morpholine-4-carboxamide (**4**) S54
1-(*p*-Tolyl)-1,4-dihydro-5*H*-tetrazol-5-one (**5**) S55
2-Azido-1-phenylethan-1-one (**7a**) S56
2-Azido-1-(4-methoxyphenyl)ethan-1-one (**7b**) S57
2-Azido-1-(*p*-tolyl)ethan-1-one (**7c**) S58
2-Azido-1-(4-ethylphenyl)ethan-1-one (**7d**) S59
Methyl-4-(2-azidoacetyl)benzoate (**7e**) S60

2-Azido-1-(4-(methylthio)phenyl)ethan-1-one (**7f**) S61

4-(2-Azidoacetyl)benzonitrile (**7g**) S62

1-([1,1'-Biphenyl]-4-yl)-2-azidoethan-1-one (**7h**) S63

2-Azido-1-(4-nitrophenyl)ethan-1-one (**7i**) S64

2-Azido-1-(4-fluorophenyl)ethan-1-one (**7j**) S65

2-Azido-1-(4-chlorophenyl)ethan-1-one (**7k**) S66

2-Azido-1-(4-bromophenyl)ethan-1-one (**7l**) S67

2-Azido-1-(4-iodophenyl)ethan-1-one (**7m**) S68

2-Azido-1-(3-methoxyphenyl)ethan-1-one (**7n**) S69

2-Azido-1-(*m*-tolyl)ethan-1-one (**7o**) S70

2-Azido-1-(3-fluorophenyl)ethan-1-one (**7p**) S71

2-Azido-1-(3-chlorophenyl)ethan-1-one (**7q**) S72

2-Azido-1-(3-bromophenyl)ethan-1-one (**7r**) S73

2-Azido-1-(3-nitrophenyl)ethan-1-one (**7s**) S74

2-Azido-1-(3-nitrophenyl)ethan-1-one (**7t**) S75

2-Azido-1-(o-tolyl)ethan-1-one (**7u**) S76

2-Azido-1-(2-bromophenyl)ethan-1-one (**7v**) S77

2-Azido-1-(3-bromo-4-methylphenyl)ethan-1-one (**7w**) S78

2-Azido-1-(3,4-dichlorophenyl)ethan-1-one (**7x**) S79

2-Azido-1-(2,4-dimethylphenyl)ethan-1-one (**7y**) S80

2-Azido-1-(naphthalen-1-yl)ethan-1-one (**7z**) S81

2-Azido-1-(naphthalen-2-yl)ethan-1-one (**7aa**) S82

2-Azido-1-(furan-2-yl)ethan-1-one (**7ab**) S83

2-Azido-1-(thiophen-2-yl)ethan-1-one (**7ac**) S84

VI. Computational details S85

VII. Mechanistic considerations S98

VIII. References S99

I. General information

All reagents were purchased from commercial sources and used without treatment unless otherwise indicated. The products were purified by column chromatography over silica gel. The products were purified by column chromatography over silica gel. ^{1}H nuclear magnetic resonance (NMR) and ^{13}C NMR spectra were recorded at 25 °C on a Varian spectrometer at 400 and 101 MHz, respectively, with TMS as the internal standard. Mass spectra were recorded on a BRUKER AutoflexIII Smartbeam MS-spectrometer. High-resolution mass spectra (HRMS) were recorded on a Bruker microTof using ESI-TOF. Infrared spectroscopy was performed on a ThermoFisher Scientific Nicolet iS10 FTIR spectrometer.

II. Crystallography

Compound **3a** (50 mg) was dissolved in a centrifuge tube in 150 µL $CDCl_3$. Upon standing for several days (seven days), crystals suitable for X-ray diffraction of compound **3a** were obtained. Single-crystal X-ray diffraction data for the reported compound was recorded at a temperature of 296(2) K on an Oxford Diffraction Gemini R Ultra diffractometer using a ω scan technique with Mo-Kα radiation (λ = 0.71073 Å). The structures were solved by the Direct Method of SHELXS-97 and refined by full-matrix least-squares techniques using the SHELXL-97 program. Non-hydrogen atoms were refined with anisotropic temperature parameters, and the hydrogen atoms of the ligands were refined as rigid groups. Basic information pertaining to crystal parameters and structure refinement is summarized in Table S1.

Table S1. Crystal structure and refinement data for compound **3a** (thermal ellipsoids at 30% probability).

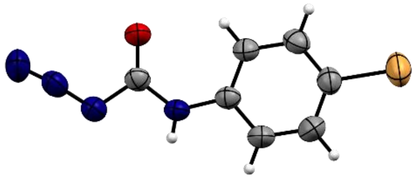

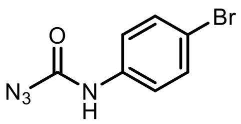

Empirical formula	$C_7H_5BrN_4O$
Temperature	296(2) K
Wavelength	0.71073 Å
Space group	P2(1)/c
Unit cell dimensions	a = 12.105(5) Å<br>b = 9.748(4) Å<br>c = 7.595(3) Å<br>alpha = 90 deg.<br>beta = 96.854(6) deg.<br>gamma = 90 deg.
Volume	889.8(7) $Å^3$
Z	4
Calculated density	1.800 Mg/m^3
Absorption coefficient	4.583 mm^{-1}
F(000)	472
Crystal size	0.146 x 0.123 x 0.098 mm
Theta range for data collection	2.690 to 25.019 deg.
Reflections collected / unique	4383 / 1570 [R(int) = 0.0216]
Data / restraints / parameters	1570 / 0 / 122
Goodness-of-fit on F^2	1.050
Final R indices [I>2sigma(I)]	R1 = 0.0323, wR2 = 0.0793
R indices (all data)	R1 = 0.0413, wR2 = 0.0835

III. Synthesis of compounds 1 and 6

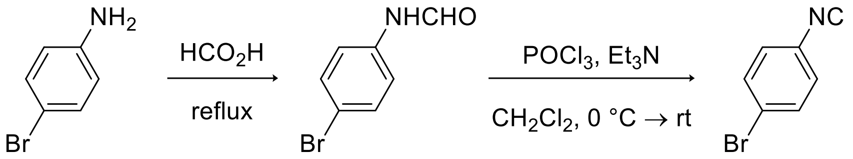

General procedure for the synthesis of isocyanides (1): A solution of the amine (20.0 mmol, 1.0 equiv.) was stirred at 0 °C, formic acid (34.6 mmol, 1.73 equiv.) was dropwise added. Then, the reaction mixture was heated to reflux overnight. Upon completion, the solvents were removed under vacuum and the crude product was directly used for the next step without further purification. To a CH_2Cl_2 solution containing the crude product and Et_3N (100.0 mmol, 5.0 equiv.), $POCl_3$ (0.95 equiv.) was added dropwise over 20 minutes. The resulting mixture was stirred at 0 °C for 1 h and further stirred at ambient temperature for 2 h. A sodium bicarbonate solution was added and the resulting mixture was extracted with CH_2Cl_2. The solvents were removed under vacuum and the crude product was purified by flash chromatography to give the pure isocyanide **1**.

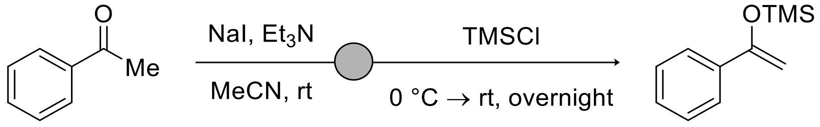

General procedure for the synthesis of aryl silyl enol ethers (6): NaI (210 mg, 1.4 mmol, 1.4 equiv) was placed in a tube and dried under vacuum using a heat gun. Upon cooling to room temperature, the tube was filled with argon. Then, dry CH_3CN (1.0 mL), ketone (1 mmol, 1.0 equiv), and Et_3N (210 µL, 1.5 mmol, 1.5 equiv) were successively added. The mixture was cooled with an ice/water bath, and TMSCl (166 µL, 1.3 mmol, 1.3 equiv) was added at 0 °C. The cooling bath was removed, and the mixture was stirred at room temperature for 12 h. Then, the volatile components were evaporated under vacuum. The solid residue was washed with petroleum ether (3 × 15 mL), the petroleum ether layers were decanted and filtered through a cotton plug. The combined filtrates were concentrated on a rotary evaporator, furnishing the silyl enol ether **6** which was used without further purification.

Note: $TMSN_3$ is commercially available.

CAUTION! Azides are a class of highly reactive and versatile chemicals that have toxic properties and can be potentially explosive and shock-sensitive under certain conditions. Therefore, azides require caution during preparation, storage, handling, and disposal. Organic azides are particularly sensitive to $TMSN_3$ and experience severe decomposition from external energy sources, such as light, heat, friction, and pressure. Therefore, azides should be kept at a temperature below 0 °C and in the absence of light (preferably in amber plastic containers). Given that exposure to azide can occur through inhalation, ingestion, or absorption. Exposure symptoms may include eye and skin irritation, dizziness, blurred vision, weakness/fatigue, low blood pressure, cardiac arrhythmia, renal effects, seizures, and respiratory failure. Therefore, the following guidelines should be followed before working with $TMSN_3$. All personal protective equipment must be worn, including a lab coat, safety glasses, and gloves with adequate chemical resistance. The reaction was conducted behind a blast shield in a fumehood with the sash positioned as low as possible to avoid inhalation exposure to $TMSN_3$ vapors. The container used for this reaction must be completely dry because water and strong acids can lead to the formation of hydrazoic acid, which is very toxic, volatile, and explosive. $TMSN_3$ should be used in an insulated container from light at a temperature below 0 °C. After the reaction components were added, the container lid should be sealed completely. The purification process of the products must be carried out with the mentioned personal protective equipment. It should be noted that in a larger scale reaction, in addition to following the above-mentioned precautions, handling of $TMSN_3$ was done using double gloves (inner-nitrile surgical style, outer-silvershield), a silvershield apron, and a supplied air respirator.[1]

IV. Synthesis and analytical data of compounds 3, 4, 5 and 7

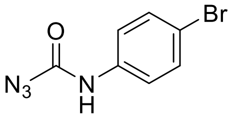

(4-Bromophenyl)carbamoyl azide (3a)[2]

To a 10 mL Schlenk tube equipped with a magnetic stir bar was added $TMSN_3$ **2** (85 μL, 0.65 mmol, 1.3 equiv), isocyanide **1a** (91.0 mg, 0.5 mmol, 1.0 equiv), DMSO (2.0 mL), $PhI(OAc)_2$ (322.1 mg, 1.0 mmol, 2 equiv). The reaction mixture was stirred at 25 °C in an oil bath for about 0.5 h. The resulting mixture was concentrated and the residue was taken up in ethyl acetate. The organic layer was washed with brine, dried over Na_2SO_4 and concentrated. Purification of the crude product by column chromatography (silica gel; petroleum ether/ethyl acetate = 20:1) afforded **3a** in 85% yield (102 mg). Faint white solid; mp 122–123 °C; ^{1}H NMR ($CDCl_3$, 400 MHz): δ_H 7.45 (d, J = 8.8 Hz, 2H), 7.34 (d, J = 8.4 Hz, 2H), 6.91 (s, 1H); ^{13}C{^{1}H} NMR ($CDCl_3$, 101 MHz): δ_C 154.0, 135.9, 133.2, 132.2, 126.4, 120.7, 117.3; IR (KBr, cm^{-1}) ν: 3501, 2124, 1693.

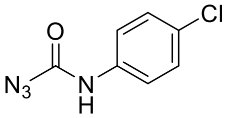

(4-Chlorophenyl)carbamoyl azide (3b)[2]

To a 10 mL Schlenk tube equipped with a magnetic stir bar was added $TMSN_3$ **2** (85 μL, 0.65 mmol, 1.3 equiv), isocyanide **1b** (68.5 mg, 0.5 mmol, 1.0 equiv), DMSO (2.0 mL), $PhI(OAc)_2$ (322.1 mg, 1.0 mmol, 2 equiv). The reaction mixture was stirred at 25 °C in an oil bath for about 0.5 h. The resulting mixture was concentrated and the residue was taken up in ethyl acetate. The organic layer was washed with brine, dried over Na_2SO_4 and concentrated. Purification of the crude product by column chromatography (silica gel; petroleum ether/ethyl acetate = 20:1) afforded **3b** in 77% yield (75 mg). Faint white solid; mp 103–104 °C; ^{1}H NMR ($CDCl_3$, 400 MHz): δ_H 7.39 (d, J = 8.4 Hz, 2H), 7.29 (d, J = 8.4 Hz, 2H), 6.97 (s, 1H); ^{13}C{^{1}H} NMR ($CDCl_3$, 101 MHz): δ_C 154.0, 135.4, 129.7, 129.2, 120.5; IR (KBr, cm^{-1}) ν: 3440, 2130, 1694.

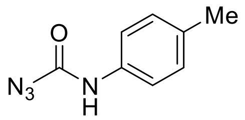

p-Tolylcarbamoyl azide (3c)[2]

To a 10 mL Schlenk tube equipped with a magnetic stir bar was added $TMSN_3$ **2** (85 µL, 0.65 mmol, 1.3 equiv), isocyanide **1c** (58.5 mg, 0.5 mmol, 1.0 equiv), DMSO (2.0 mL), $PhI(OAc)_2$ (322.1 mg, 1.0 mmol, 2 equiv). The reaction mixture was stirred at 25 °C in an oil bath for about 0.5 h. The resulting mixture was concentrated and the residue was taken up in ethyl acetate. The organic layer was washed with brine, dried over Na_2SO_4 and concentrated. Purification of the crude product by column chromatography (silica gel; petroleum ether/ethyl acetate = 20:1) afforded **3c** in 82% yield (72 mg).

Gram-scale reaction: To a 100 mL flask equipped with a magnetic stir bar was added $TMSN_3$ **2** (1710.0 µL, 13.0 mmol, 1.3 equiv), isocyanide **1c** (1170.6 mg, 10.0 mmol, 1.0 equiv), DMSO (40.0 mL), $PhI(OAc)_2$ (6442.0 mg, 20.0 mmol, 2.0 equiv). The reaction mixture was stirred at 25 °C in an oil bath for about 0.5 h. The resulting mixture was concentrated and the residue was taken up in ethyl acetate. The organic layer was washed with brine, dried over Na_2SO_4 and concentrated. Purification of the crude product by column chromatography (silica gel; petroleum ether/ethyl acetate = 20:1) afforded **3c** in 68% yield (1197 mg).

Faint white solid; mp 100–101 °C; ^{1}H NMR ($CDCl_3$, 400 MHz): δ_H 7.31 (d, J = 7.6 Hz, 2H), 7.12 (d, J = 8.0 Hz, 2H), 6.91 (s, 1H), 2.31 (s, 3H); $^{13}C\{^1H\}$ NMR ($CDCl_3$, 101 MHz): δ_C 153.9, 134.33, 134.25, 129.6, 119.3, 20.8; IR (KBr, cm^{-1}) ν: 3321, 2150, 1682.

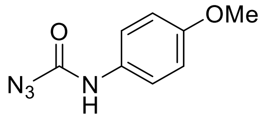

(4-Methoxyphenyl)carbamoyl azide (3d)[2]

To a 10 mL Schlenk tube equipped with a magnetic stir bar was added $TMSN_3$ **2** (85 µL, 0.65 mmol, 1.3 equiv), isocyanide **1d** (66.5 mg, 0.5 mmol, 1.0 equiv), DMSO (2.0 mL), $PhI(OAc)_2$ (322.1 mg, 1.0 mmol, 2 equiv). The reaction mixture was stirred at 25 °C in an oil bath for about 0.5 h. The resulting mixture was concentrated and the residue was taken up in ethyl acetate. The organic layer was washed with brine, dried over Na_2SO_4 and concentrated. Purification of the crude product by column chromatography (silica gel; petroleum ether/ethyl acetate = 15:1) afforded **3d** in 76% yield (73 mg).

Faint white solid; mp 103–104 °C; ^{1}H NMR ($CDCl_3$, 400 MHz): δ_H 7.34 (d, J = 8.4 Hz, 2H), 6.86 (d, J = 8.0 Hz, 2H), 3.79 (s, 3H); $^{13}C\{^1H\}$ NMR ($CDCl_3$, 101 MHz): δ_C 156.7, 154.0, 129.9, 121.2, 114.3, 55.5; IR (KBr, cm^{-1}) ν: 3390, 2160, 1678.

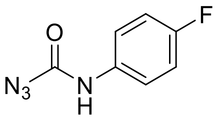

(4-Fluorophenyl)carbamoyl azide (3e)[2]

To a 10 mL Schlenk tube equipped with a magnetic stir bar was added $TMSN_3$ **2** (85 µL, 0.65 mmol, 1.3 equiv), isocyanide **1e** (60.5 mg, 0.5 mmol, 1.0 equiv), DMSO (2.0 mL), $PhI(OAc)_2$ (322.1 mg, 1.0 mmol, 2 equiv). The reaction mixture was stirred at 25 °C in an oil bath for about 0.5 h. The resulting mixture was concentrated and the residue was taken up in ethyl acetate. The organic layer was washed with brine, dried over Na_2SO_4 and concentrated. Purification of the crude product by column chromatography (silica gel; petroleum ether/ethyl acetate = 20:1) afforded **3e** in 74% yield (67 mg).

Faint white solid; mp 105–106 °C; ^{1}H NMR ($CDCl_3$, 400 MHz): δ_H 7.42–7.38 (m, 2H), 7.05–7.00 (m, 2H), 6.98 (s, 1H); $^{13}C\{^1H\}$ NMR ($CDCl_3$, 101 MHz): δ_C 159.6 (d, J_{C-F} = 245.0 Hz), 154.2, 132.9, 121.2 (d, J_{C-F} = 8.0 Hz), 116.0 (d, J_{C-F} = 22.8 Hz); IR (KBr, cm^{-1}) ν: 3461, 2143, 1681.

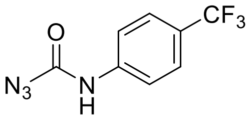

(4-(Trifluoromethyl)phenyl)carbamoyl azide (3f)[3]

To a 10 mL Schlenk tube equipped with a magnetic stir bar was added $TMSN_3$ **2** (85 µL, 0.65 mmol, 1.3 equiv), isocyanide **1f** (85.5 mg, 0.5 mmol, 1.0 equiv), DMSO (2.0 mL), $PhI(OAc)_2$ (322.1 mg, 1.0 mmol, 2 equiv). The reaction mixture was stirred at 25 °C in an oil bath for about 0.5 h. The resulting mixture was concentrated and the residue was taken up in ethyl acetate. The organic layer was washed with brine, dried over Na_2SO_4 and concentrated. Purification of the crude product by column chromatography (silica gel; petroleum ether/ethyl acetate = 20:1) afforded **3f** in 70% yield (81 mg).

Faint white solid; mp 105–106 °C; ^{1}H NMR ($CDCl_3$, 400 MHz): δ_H 7.61–7.55 (m, 4H), 7.09 (s, 1H); $^{13}C\{^1H\}$ NMR ($CDCl_3$, 101 MHz): δ_C 154.2, 140.0, 126.5 (t, J_{C-F} = 3.6 Hz), 125.3, 122.6, 118.8; IR (KBr, cm^{-1}) ν: 3342, 2158, 1675.

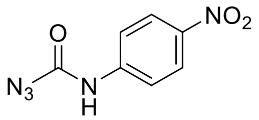

(4-Nitrophenyl)carbamoyl azide (3g)[2]

To a 10 mL Schlenk tube equipped with a magnetic stir bar was added $TMSN_3$ **2** (85 μL, 0.65 mmol, 1.3 equiv), isocyanide **1g** (74.0 mg, 0.5 mmol, 1.0 equiv), DMSO (2.0 mL), $PhI(OAc)_2$ (322.1 mg, 1.0 mmol, 2 equiv). The reaction mixture was stirred at 25 °C in an oil bath for about 0.5 h. The resulting mixture was concentrated and the residue was taken up in ethyl acetate. The organic layer was washed with brine, dried over Na_2SO_4 and concentrated. Purification of the crude product by column chromatography (silica gel; petroleum ether/ethyl acetate = 20:1) afforded **3g** in 56% yield (58 mg). Faint white solid; mp 115–116 °C; ^1H NMR ($CDCl_3$, 400 MHz): δ_H 8.24 (d, *J* = 9.2 Hz, 2H), 7.63 (d, *J* = 8.8 Hz, 2H), 7.23 (s, 1H); $^{13}C\{^1H\}$ NMR ($CDCl_3$, 101 MHz): δ_C154.2, 143.8, 142.7, 125.2, 118.6; IR (KBr, cm^{-1}) ν: 3317, 2135, 1708.

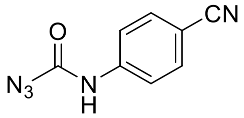

(4-Cyanophenyl)carbamoyl azide (3h)[3]

To a 10 mL Schlenk tube equipped with a magnetic stir bar was added $TMSN_3$ **2** (85 μL, 0.65 mmol, 1.3 equiv), isocyanide **1h** (64.0 mg, 0.5 mmol, 1.0 equiv), DMSO (2.0 mL), $PhI(OAc)_2$ (322.1 mg, 1.0 mmol, 2 equiv). The reaction mixture was stirred at 25 °C in an oil bath for about 0.5 h. The resulting mixture was concentrated and the residue was taken up in ethyl acetate. The organic layer was washed with brine, dried over Na_2SO_4 and concentrated. Purification of the crude product by column chromatography (silica gel; petroleum ether/ethyl acetate = 18:1) afforded **3h** in 67% yield (63 mg). Faint white solid; mp 123–124 °C; ^1H NMR ($CDCl_3$, 400 MHz): δ_H 7.65–7.59 (m, 4H), 7.34 (s, 1H); $^{13}C\{^1H\}$ NMR ($CDCl_3$, 101 MHz): δ_C 154.2, 141.1, 133.4, 119.0, 118.6, 107.5; IR (KBr, cm^{-1}) ν: 3314, 2164, 1682.

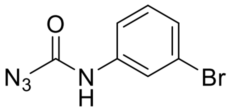

(3-Bromophenyl)carbamoyl azide (3i)[4]

To a 10 mL Schlenk tube equipped with a magnetic stir bar was added $TMSN_3$ **2** (85 µL, 0.65 mmol, 1.3 equiv), isocyanide **1i** (90.5 mg, 0.5 mmol, 1.0 equiv), DMSO (2.0 mL), $PhI(OAc)_2$ (322.1 mg, 1.0 mmol, 2 equiv). The reaction mixture was stirred at 25 °C in an oil bath for about 0.5 h. The resulting mixture was concentrated and the residue was taken up in ethyl acetate. The organic layer was washed with brine, dried over Na_2SO_4 and concentrated. Purification of the crude product by column chromatography (silica gel; petroleum ether/ethyl acetate = 20:1) afforded **3i** in 80% yield (96 mg). Faint white solid; mp 127–128 °C; ^{1}H NMR ($CDCl_3$, 400 MHz): δ_H 7.70 (s, 1H), 7.34 (d, J = 7.6 Hz, 1H), 7.28 (s, 1H), 7.22–7.18 (m, 1H), 6.83 (s, 1H); $^{13}C\{^1H\}$ NMR ($CDCl_3$, 101 MHz): δ_C 138.1, 130.5, 127.7, 122.8, 122.1, 117.6; IR (KBr, cm^{-1}) ν: 3441, 2159, 1693.

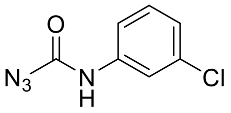

(3-Chlorophenyl)carbamoyl azide (3j)[2]

To a 10 mL Schlenk tube equipped with a magnetic stir bar was added $TMSN_3$ **2** (85 µL, 0.65 mmol, 1.3 equiv), isocyanide **1j** (68.5 mg, 0.5 mmol, 1.0 equiv), DMSO (2.0 mL), $PhI(OAc)_2$ (322.1 mg, 1.0 mmol, 2 equiv). The reaction mixture was stirred at 25 °C in an oil bath for about 0.5 h. The resulting mixture was concentrated and the residue was taken up in ethyl acetate. The organic layer was washed with brine, dried over Na_2SO_4 and concentrated. Purification of the crude product by column chromatography (silica gel; petroleum ether/ethyl acetate = 20:1) afforded **3j** in 71% yield (70 mg). Faint white solid; mp 130–131 °C; ^{1}H NMR ($CDCl_3$, 400 MHz): δ_H 7.55 (s, 1H), 7.29–7.23 (m, 2H), 7.11 (d, J = 7.2 Hz, 1H), 6.97 (s, 1H); $^{13}C\{^1H\}$ NMR ($CDCl_3$, 101 MHz): δ_C 154.0, 138.0, 134.9, 130.1, 124.7, 119.3, 117.2; IR (KBr, cm^{-1}) ν: 3486, 2141, 1697.

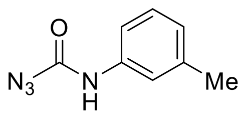

***m*-Tolylcarbamoyl azide (3k)**[5]

To a 10 mL Schlenk tube equipped with a magnetic stir bar was added $TMSN_3$ **2** (85 µL, 0.65 mmol, 1.3 equiv), isocyanide **1k** (58.5 mg, 0.5 mmol, 1.0 equiv), DMSO (2.0 mL), $PhI(OAc)_2$ (322.1 mg, 1.0 mmol, 2 equiv). The reaction mixture was stirred at 25 °C in an oil bath for about 0.5 h. The resulting mixture was concentrated and the residue was taken up in ethyl acetate. The organic layer was washed with brine, dried over Na_2SO_4 and concentrated. Purification of the crude product by column chromatography (silica gel; petroleum ether/ethyl acetate = 20:1) afforded **3k** in 73% yield (64 mg).

Faint white solid; mp 144–145 °C; ^1H NMR ($CDCl_3$, 400 MHz): δ_H 7.27 (s, 1H), 7.22–7.18 (m, 2H), 6.94 (s, 1H), 6.90 (s, 1H); $^{13}C\{^1H\}$ NMR ($CDCl_3$, 101 MHz): δ_C 153.9, 139.2, 136.7, 129.0, 125.4, 119.8, 116.3, 21.4; IR (KBr, cm^{-1}) ν: 3366, 2167, 1688.

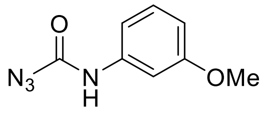

(3-Methoxyphenyl)carbamoyl azide (3l)[5]

To a 10 mL Schlenk tube equipped with a magnetic stir bar was added $TMSN_3$ **2** (85 µL, 0.65 mmol, 1.3 equiv), isocyanide **1l** (66.5 mg, 0.5 mmol, 1.0 equiv), DMSO (2.0 mL), $PhI(OAc)_2$ (322.1 mg, 1.0 mmol, 2 equiv). The reaction mixture was stirred at 25 °C in an oil bath for about 0.5 h. The resulting mixture was concentrated and the residue was taken up in ethyl acetate. The organic layer was washed with brine, dried over Na_2SO_4 and concentrated. Purification of the crude product by column chromatography (silica gel; petroleum ether/ethyl acetate = 15:1) afforded **3l** in 76% yield (73 mg).

Faint white solid; mp 156–157 °C; ^1H NMR ($CDCl_3$, 400 MHz): δ_H 7.24–7.17 (m, 1H), 6.95 (s, 1H), 6.91 (d, J = 8.0 Hz, 1H), 6.68 (d, J = 8.4 Hz, 1H), 3.80 (s, 3H); $^{13}C\{^1H\}$ NMR ($CDCl_3$, 101 MHz): δ_C 160.2, 153.9, 138.0, 129.9, 111.3, 110.4, 105.0, 55.3; IR (KBr, cm^{-1}) ν: 3354, 2136, 1715.

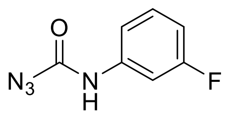

(3-Fluorophenyl)carbamoyl azide (3m)

To a 10 mL Schlenk tube equipped with a magnetic stir bar was added $TMSN_3$ **2** (85 µL, 0.65 mmol, 1.3 equiv), isocyanide **1m** (60.5 mg, 0.5 mmol, 1.0 equiv), DMSO (2.0 mL), $PhI(OAc)_2$ (322.1 mg, 1.0 mmol, 2 equiv). The reaction mixture was stirred at 25 °C in an oil bath for about 0.5 h. The resulting mixture was concentrated and the residue was taken up in ethyl acetate. The organic layer was washed with brine, dried over Na_2SO_4 and concentrated. Purification of the crude product by column chromatography (silica gel; petroleum ether/ethyl acetate = 20:1) afforded **3m** in 65% yield (59 mg). Faint white solid; mp 129–130 °C; ^{1}H NMR ($CDCl_3$, 400 MHz): δ_H 7.38 (d, J = 10.4 Hz, 1H), 7.31–7.25 (m, 1H), 7.08 (d, J = 8.0 Hz, 1H), 6.93 (s, 1H), 6.86–6.82 (m, 1H); ^{13}C{^{1}H} NMR ($CDCl_3$, 101 MHz): δ_C 163.0 (d, $J_{C–F}$ = 244.2 Hz), 154.0, 138.4 (d, $J_{C–F}$ = 10.4 Hz), 130.4 (d, $J_{C–F}$ = 9.4 Hz), 114.4, 111.4 (d, $J_{C–F}$ = 21.3 Hz), 106.7 (d, $J_{C–F}$ = 26.5 Hz); IR (KBr, cm^{-1}) ν: 3322, 2134, 1686.

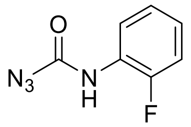

(2-Fluorophenyl)carbamoyl azide (3n)

To a 10 mL Schlenk tube equipped with a magnetic stir bar was added $TMSN_3$ **2** (85 µL, 0.65 mmol, 1.3 equiv), isocyanide **1n** (60.5 mg, 0.5 mmol, 1.0 equiv), DMSO (2.0 mL), $PhI(OAc)_2$ (322.1 mg, 1.0 mmol, 2 equiv). The reaction mixture was stirred at 25 °C in an oil bath for about 0.5 h. The resulting mixture was concentrated and the residue was taken up in ethyl acetate. The organic layer was washed with brine, dried over Na_2SO_4 and concentrated. Purification of the crude product by column chromatography (silica gel; petroleum ether/ethyl acetate = 20:1) afforded **3n** in 61% yield (55 mg). Faint white solid; mp 144–145 °C; ^{1}H NMR ($CDCl_3$, 400 MHz): δ_H 8.18–8.14 (m, 1H), 7.15 (s, 1H), 7.12–7.08 (m, 3H); ^{13}C{^{1}H} NMR ($CDCl_3$, 101 MHz): δ_C 153.7, 153.5, 151.1, 125.5 (d, $J_{C–F}$ = 9.2 Hz), 124.7 (t, $J_{C–F}$ = 2.5 Hz), 120.8, 115.1 (d, $J_{C–F}$ = 18.6 Hz); HRMS (ESI-TOF, *m/z*): calcd for $C_7H_6FN_4O$ [M + H]$^+$, 181.0520; found, 181.0529; IR (KBr, cm^{-1}) ν: 3319, 2151, 1678.

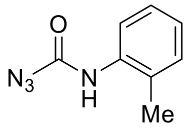

***o*-Tolylcarbamoyl azide (3o)**[6]

To a 10 mL Schlenk tube equipped with a magnetic stir bar was added $TMSN_3$ **2** (85 µL, 0.65 mmol, 1.3 equiv), isocyanide **1o** (58.5 mg, 0.5 mmol, 1.0 equiv), DMSO (2.0 mL), $PhI(OAc)_2$ (322.1 mg, 1.0 mmol, 2 equiv). The reaction mixture was stirred at 25 °C in an oil bath for about 0.5 h. The resulting mixture was concentrated and the residue was taken up in ethyl acetate. The organic layer was washed with brine, dried over Na_2SO_4 and concentrated. Purification of the crude product by column chromatography (silica gel; petroleum ether/ethyl acetate = 20:1) afforded **3o** in 84% yield (74 mg). Faint white solid; mp 169–170 °C; ^{1}H NMR ($CDCl_3$, 400 MHz): δ_H 7.81 (d, J = 7.2 Hz, 2H), 7.26–7.17 (m, 2H), 7.11–7.08 (m, 1H), 6.64 (s, 1H), 2.25 (s, 3H); $^{13}C\{^1H\}$ NMR ($CDCl_3$, 101 MHz): δ_C 154.2, 134.8, 130.6, 128.3, 127.0, 125.3, 121.8, 17.6; IR (KBr, cm^{-1}) ν: 3323, 2152, 1705.

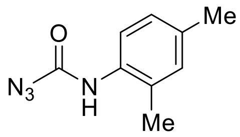

(2,4-Dimethylphenyl)carbamoyl azide (3p)[7]

To a 10 mL Schlenk tube equipped with a magnetic stir bar was added $TMSN_3$ **2** (85 µL, 0.65 mmol, 1.3 equiv), isocyanide **1p** (65.5 mg, 0.5 mmol, 1.0 equiv), DMSO (2.0 mL), $PhI(OAc)_2$ (322.1 mg, 1.0 mmol, 2 equiv). The reaction mixture was stirred at 25 °C in an oil bath for about 0.5 h. The resulting mixture was concentrated and the residue was taken up in ethyl acetate. The organic layer was washed with brine, dried over Na_2SO_4 and concentrated. Purification of the crude product by column chromatography (silica gel; petroleum ether/ethyl acetate = 20:1) afforded **3p** in 73% yield (69 mg). Faint white solid; mp 90–91 °C; ^{1}H NMR ($CDCl_3$, 400 MHz): δ_H 7.61 (d, J = 7.2 Hz, 1H), 7.03–7.00 (m, 2H), 6.56 (s, 1H), 2.30 (s, 3H), 2.21 (s, 3H); $^{13}C\{^1H\}$ NMR ($CDCl_3$, 101 MHz): δ_C 154.3, 135.2, 132.1, 131.2, 128.8, 127.5, 122.3, 20.8, 17.5; IR (KBr, cm^{-1}) ν: 3313, 2134, 1712.

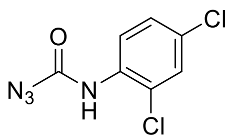

(2,4-Dichlorophenyl)carbamoyl azide (3q)[8]

To a 10 mL Schlenk tube equipped with a magnetic stir bar was added $TMSN_3$ **2** (85 μL, 0.65 mmol, 1.3 equiv), isocyanide **1q** (85.5 mg, 0.5 mmol, 1.0 equiv), DMSO (2.0 mL), $PhI(OAc)_2$ (322.1 mg, 1.0 mmol, 2 equiv). The reaction mixture was stirred at 25 °C in an oil bath for about 0.5 h. The resulting mixture was concentrated and the residue was taken up in ethyl acetate. The organic layer was washed with brine, dried over Na_2SO_4 and concentrated. Purification of the crude product by column chromatography (silica gel; petroleum ether/ethyl acetate = 20:1) afforded **3q** in 77% yield (89 mg). Faint white solid; mp 101–102 °C; ^{1}H NMR ($CDCl_3$, 400 MHz): δ_H 8.19 (d, *J* = 8.0 Hz, 1H), 7.39 (s, 1H), 7.27 (d, *J* = 8.8 Hz, 2H); $^{13}C\{^1H\}$ NMR ($CDCl_3$, 101 MHz): δ_C 154.0, 132.5, 129.6, 128.9, 128.1, 123.2, 121.3; IR (KBr, cm^{-1}) ν: 3270, 2172, 1682.

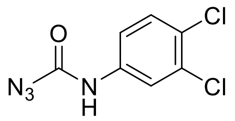

(3,4-Dichlorophenyl)carbamoyl azide (3r)

To a 10 mL Schlenk tube equipped with a magnetic stir bar was added $TMSN_3$ **2** (85 μL, 0.65 mmol, 1.3 equiv), isocyanide **1r** (85.5 mg, 0.5 mmol, 1.0 equiv), DMSO (2.0 mL), $PhI(OAc)_2$ (322.1 mg, 1.0 mmol, 2 equiv). The reaction mixture was stirred at 25 °C in an oil bath for about 0.5 h. The resulting mixture was concentrated and the residue was taken up in ethyl acetate. The organic layer was washed with brine, dried over Na_2SO_4 and concentrated. Purification of the crude product by column chromatography (silica gel; petroleum ether/ethyl acetate = 20:1) afforded **3q** in 79% yield (91 mg). Faint white solid; mp 121–122 °C; ^{1}H NMR ($CDCl_3$, 400 MHz): δ_H 7.67 (s, 1H), 7.39 (d, *J* = 8.8 Hz, 1H), 7.26 (d, *J* = 6.4 Hz, 1H), 6.89 (s, 1H); $^{13}C\{^1H\}$ NMR ($CDCl_3$, 101 MHz): δ_C 154.0, 136.3, 133.1, 130.7, 128.0, 120.9, 118.4; HRMS (ESI-TOF, *m/z*): calcd for $C_7H_5Cl_2N_4O$ $[M + H]^+$, 230.9835; found, 230.9837; IR (KBr, cm^{-1}) ν: 3331, 2134, 1703.

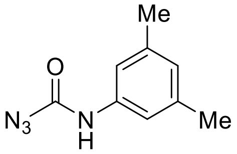

(3,5-Dimethylphenyl)carbamoyl azide (3s)

To a 10 mL Schlenk tube equipped with a magnetic stir bar was added $TMSN_3$ **2** (85 µL, 0.65 mmol, 1.3 equiv), isocyanide **1s** (65.5 mg, 0.5 mmol, 1.0 equiv), DMSO (2.0 mL), $PhI(OAc)_2$ (322.1 mg, 1.0 mmol, 2 equiv). The reaction mixture was stirred at 25 °C in an oil bath for about 0.5 h. The resulting mixture was concentrated and the residue was taken up in ethyl acetate. The organic layer was washed with brine, dried over Na_2SO_4 and concentrated. Purification of the crude product by column chromatography (silica gel; petroleum ether/ethyl acetate = 20:1) afforded **3s** in 82% yield (78 mg). Faint white solid; mp 123–124 °C; ^1H NMR ($CDCl_3$, 400 MHz): δ_H 8.99 (s, 1H), 7.32 (s, 2H), 7.16 (s, 1H), 2.43 (s, 6H); $^{13}C\{^1H\}$ NMR ($CDCl_3$, 101 MHz): δ_C 140.5, 140.3, 133.6, 131.5, 118.8, 21.2; IR (KBr, cm^{-1}) ν : 3402, 2142, 1697.

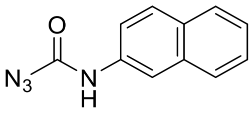

Naphthalen-2-ylcarbamoyl azide (3t)[2]

To a 10 mL Schlenk tube equipped with a magnetic stir bar was added $TMSN_3$ **2** (85 µL, 0.65 mmol, 1.3 equiv), isocyanide **1t** (76.5 mg, 0.5 mmol, 1.0 equiv), DMSO (2.0 mL), $PhI(OAc)_2$ (322.1 mg, 1.0 mmol, 2 equiv). The reaction mixture was stirred at 25 °C in an oil bath for about 0.5 h. The resulting mixture was concentrated and the residue was taken up in ethyl acetate. The organic layer was washed with brine, dried over Na_2SO_4 and concentrated. Purification of the crude product by column chromatography (silica gel; petroleum ether/ethyl acetate = 20:1) afforded **3t** in 67% yield (71 mg). Faint white solid; mp 51–52 °C; ^1H NMR ($CDCl_3$, 400 MHz): δ_H 7.91–7.87 (m, 2H), 7.81 (d, *J* = 7.6 Hz, 1H), 7.72 (d, *J* = 8.4 Hz, 1H), 7.56–7.46 (m, 3H), 4.93 (s, 1H); $^{13}C\{^1H\}$ NMR ($CDCl_3$, 101 MHz): δ_C 154.9, 134.0, 131.4, 128.8, 126.6, 126.2, 125.6, 120.1; IR (KBr, cm^{-1}) ν: 3342, 2112, 1681.

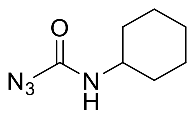

Cyclohexylcarbamoyl azide (3u)[9]

To a 10 mL Schlenk tube equipped with a magnetic stir bar was added $TMSN_3$ **2** (85 µL, 0.65 mmol, 1.3 equiv), isocyanide **1u** (55.1 mg, 0.5 mmol, 1.0 equiv), DMSO (2.0 mL), $PhI(OAc)_2$ (322.1 mg, 1.0 mmol, 2 equiv). The reaction mixture was stirred at 25 °C in an oil bath for about 0.5 h. The resulting mixture was concentrated and the residue was taken up in ethyl acetate. The organic layer was washed with brine, dried over Na_2SO_4 and concentrated. Purification of the crude product by column chromatography (silica gel; petroleum ether/ethyl acetate = 20:1) afforded **3u** in 82% yield (69 mg). Faint white solid; mp 104.3–105.2 °C; ^{1}H NMR (DMSO-d_6, 400 MHz): δ_H 7.96 (s, 1H), 1.75-1.65 (m, 4H), 1.56-1.52 (m, 1H), 1.28-1.02 (m, 6H); ^{13}C{^{1}H} NMR (DMSO-d_6, 101 MHz): δ_C 154.4, 49.8, 32.2, 25.0, 24.5; IR (KBr, cm^{-1}) ν: 3279, 2138, 1677, 1541.

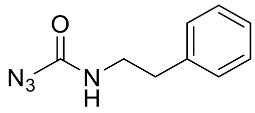

Phenethylcarbamoyl azide (3v)[9]

To a 10 mL Schlenk tube equipped with a magnetic stir bar was added $TMSN_3$ **2** (85 µL, 0.65 mmol, 1.3 equiv), isocyanide **1v** (66.1 mg, 0.5 mmol, 1.0 equiv), DMSO (2.0 mL), $PhI(OAc)_2$ (322.1 mg, 1.0 mmol, 2 equiv). The reaction mixture was stirred at 25 °C in an oil bath for about 0.5 h. The resulting mixture was concentrated and the residue was taken up in ethyl acetate. The organic layer was washed with brine, dried over Na_2SO_4 and concentrated. Purification of the crude product by column chromatography (silica gel; petroleum ether/ethyl acetate = 20:1) afforded **3v** in 74% yield (70 mg). Faint white solid; mp 84.7–85.7 °C; ^{1}H NMR (DMSO-d_6, 400 MHz): δ_H 8.07 (s, 1H), 7.27-7.23 (m, 2H), 7.17-7.15 (m, 3H), 3.24 (q, J = 6.8 Hz, 2H), 2.70 (t, J = 8.4 Hz, 2H); ^{13}C{^{1}H} NMR (DMSO-d_6, 101 MHz): δ_C 155.5, 139.0, 128.7, 128.4, 126.2, 41.9, 34.9; IR (KBr, cm^{-1}) ν: 3281, 3029, 2930, 2142, 1671, 1545.

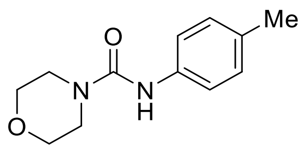

N-(p-Tolyl)morpholine-4-carboxamide (4)

To a 10 mL Schlenk tube equipped with a magnetic stir bar was added **3c** (88.0 mg, 0.5 mmol, 1.0 equiv), morpholine (87.0 mg, 1.0 mmol, 2.0 equiv), EtOAc (2.0 mL). The reaction mixture was stirred at 75 °C in an oil bath for about 12 h. The resulting mixture was concentrated and the residue was taken up in ethyl acetate. The organic layer was washed with brine, dried over Na_2SO_4 and concentrated. Purification of the crude product by column chromatography (silica gel; petroleum ether/ethyl acetate = 16:1) afforded **4** in 67% yield (74 mg).

Faint white solid; mp 135–136 °C; ^{1}H NMR ($CDCl_3$, 400 MHz): δ_H 7.22 (d, J = 8.0 Hz, 2H), 7.09 (d, J = 7.6 Hz, 2H), 6.38 (s, 1H), 3.73–3.69 (m, 4H), 3.447–3.43 (m, 4H), 2.30 (s, 3H); ^{13}C{^{1}H} NMR ($CDCl_3$, 101 MHz): δ_C 155.4, 136.0, 133.0, 129.4, 120.4, 66.5, 44.2, 20.7; HRMS (ESI-TOF, *m/z*): calcd for $C_{12}H_{17}N_2O_2$ $[M + H]^+$, 221.1285; found, 221.1279.

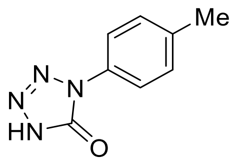

1-(p-Tolyl)-1,4-dihydro-5H-tetrazol-5-one (5)

To a 10 mL Schlenk tube equipped with a magnetic stir bar was added **3c** (88.0 mg, 0.5 mmol, 1.0 equiv), trimethylsilylazide (TMSA) (197.0 µL, 1.5 mmol, 3.0 equiv). The reaction mixture was stirred at 80 °C in an oil bath for about 24 h. The resulting mixture was concentrated and the residue was taken up in ethyl acetate. The organic layer was washed with brine, dried over Na_2SO_4 and concentrated. Purification of the crude product by column chromatography (silica gel; petroleum ether/ethyl acetate = 10:1) afforded **5** in 61% yield (54 mg).

Faint yellow solid; mp 151–152 °C;; ^{1}H NMR (DMSO-d_6, 400 MHz): δ_H 7.71 (d, J = 7.6 Hz, 2H), 7.35 (d, J = 8.0 Hz, 2H), 3.34 (s, 1H), 2.35 (s, 3H); ^{13}C{^{1}H} NMR (DMSO-d_6, 101 MHz): δ_C 150.3, 137.1, 131.8, 129.8, 129.1, 119.6, 118.2, 20.6; HRMS (ESI-TOF, *m/z*): calcd for $C_8H_9N_4O$ $[M + H]^+$, 177.0771; found, 177.0785.

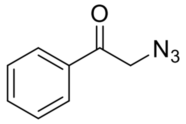

2-Azido-1-phenylethan-1-one (7a)[10]

To a 10 mL Schlenk tube equipped with a magnetic stir bar was added $TMSN_3$ **2** (85 μL, 0.65 mmol, 1.3 equiv), silyl enol ether **6b** (96.0 mg, 0.5 mmol, 1.0 equiv), DMSO (2.0 mL), $PhI(OAc)_2$ (322.1 mg, 1.0 mmol, 2 equiv). The reaction mixture was stirred at 25 °C in an oil bath for about 0.5 h. The resulting mixture was concentrated and the residue was taken up in ethyl acetate. The organic layer was washed with brine, dried over Na_2SO_4 and concentrated. Purification of the crude product by column chromatography (silica gel; petroleum ether/ethyl acetate = 20:1) afforded **7b** in 82% yield (66 mg). Faint colorless oil; ^{1}H NMR ($CDCl_3$, 400 MHz): δ_H 7.92 (d, *J* = 8.0 Hz, 2H), 7.65–7.62 (m, 1H), 7.53–7.48 (m, 2H), 4.58 (s, 2H); ^{13}C{^{1}H} NMR ($CDCl_3$, 101 MHz): δ_C 193.2, 134.3, 134.1, 129.0, 127.9, 54.9; IR (KBr, cm^{-1}) ν: 2105, 1696.

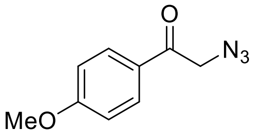

2-Azido-1-(4-methoxyphenyl)ethan-1-one (7b)[11]

To a 10 mL Schlenk tube equipped with a magnetic stir bar was added $TMSN_3$ **2** (85 μL, 0.65 mmol, 1.3 equiv), silyl enol ether **6a** (111.0 mg, 0.5 mmol, 1.0 equiv), DMSO (2.0 mL), $PhI(OAc)_2$ (322.1 mg, 1.0 mmol, 2 equiv). The reaction mixture was stirred at 25 °C in an oil bath for about 0.5 h. The resulting mixture was concentrated and the residue was taken up in ethyl acetate. The organic layer was washed with brine, dried over Na_2SO_4 and concentrated. Purification of the crude product by column chromatography (silica gel; petroleum ether/ethyl acetate = 15:1) afforded **7a** in 88% yield (84 mg). Faint white solid; mp 69–70 °C ; ^{1}H NMR ($CDCl_3$, 400 MHz): δH 7.88 (d, *J* = 8.4 Hz, 2H), 6.96 (d, *J* = 8.4 Hz, 2H), 4.51 (s, 2H), 3.88 (s, 3H); ^{13}C{^{1}H} NMR ($CDCl_3$, 101 MHz): δ_C 191.6, 164.2, 130.2, 127.3, 114.1, 55.5, 54.5; IR (KBr, cm^{-1}) ν: 2124, 1684.

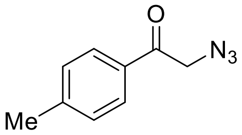

2-Azido-1-(p-tolyl)ethan-1-one (7c)[10]

To a 10 mL Schlenk tube equipped with a magnetic stir bar was added $TMSN_3$ **2** (85 µL, 0.65 mmol, 1.3 equiv), silyl enol ether **6c** (103.0 mg, 0.5 mmol, 1.0 equiv), DMSO (2.0 mL), $PhI(OAc)_2$ (322.1 mg, 1.0 mmol, 2 equiv). The reaction mixture was stirred at 25 °C in an oil bath for about 0.5 h. The resulting mixture was concentrated and the residue was taken up in ethyl acetate. The organic layer was washed with brine, dried over Na_2SO_4 and concentrated. Purification of the crude product by column chromatography (silica gel; petroleum ether/ethyl acetate = 20:1) afforded **7c** in 84% yield (74 mg). Faint white solid; mp 60–61 °C; ^{1}H NMR ($CDCl_3$, 400 MHz): δ_H 7.81 (d, J = 7.6 Hz, 2H), 7.30 (d, J = 8.0 Hz, 2H), 4.54 (s, 2H), 2.43 (s, 3H); ^{13}C{^{1}H} NMR ($CDCl_3$, 101 MHz): δ_C 192.8, 145.2, 131.9, 129.6, 128.0, 54.8, 21.8; IR (KBr, cm^{-1}) ν: 2102, 1600.

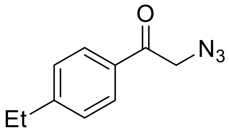

2-Azido-1-(4-ethylphenyl)ethan-1-one (7d)[12]

To a 10 mL Schlenk tube equipped with a magnetic stir bar was added $TMSN_3$ **2** (85 µL, 0.65 mmol, 1.3 equiv), silyl enol ether **6d** (110.0 mg, 0.5 mmol, 1.0 equiv), DMSO (2.0 mL), $PhI(OAc)_2$ (322.1 mg, 1.0 mmol, 2 equiv). The reaction mixture was stirred at 25 °C in an oil bath for about 0.5 h. The resulting mixture was concentrated and the residue was taken up in ethyl acetate. The organic layer was washed with brine, dried over Na_2SO_4 and concentrated. Purification of the crude product by column chromatography (silica gel; petroleum ether/ethyl acetate = 20:1) afforded **7d** in 77% yield (73 mg). Faint colorless oil; ^{1}H NMR ($CDCl_3$, 400 MHz): δ_H 7.83 (d, J = 7.6 Hz, 2H), 7.32 (d, J = 7.6 Hz, 2H), 4.54 (s, 2H), 2.72 (q, J = 7.6 Hz, 2H), 1.26 (t, J = 7.6 Hz, 3H); ^{13}C{^{1}H} NMR ($CDCl_3$, 101 MHz): δ_C 192.8, 151.3, 132.1, 128.4, 128.1, 54.7, 29.0, 15.0; IR (KBr, cm^{-1}) ν: 2105, 1607.

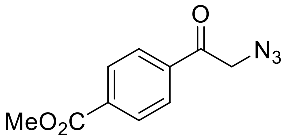

Methyl-4-(2-azidoacetyl)benzoate (7e)[13]

To a 10 mL Schlenk tube equipped with a magnetic stir bar was added $TMSN_3$ **2** (85 μL, 0.65 mmol, 1.3 equiv), silyl enol ether **6e** (125.0 mg, 0.5 mmol, 1.0 equiv), DMSO (2.0 mL), $PhI(OAc)_2$ (322.1 mg, 1.0 mmol, 2 equiv). The reaction mixture was stirred at 25 °C in an oil bath for about 0.5 h. The resulting mixture was concentrated and the residue was taken up in ethyl acetate. The organic layer was washed with brine, dried over Na_2SO_4 and concentrated. Purification of the crude product by column chromatography (silica gel; petroleum ether/ethyl acetate = 16:1) afforded **7e** in 73% yield (80 mg). Faint white solid; mp 87–88 °C; ^{1}H NMR ($CDCl_3$, 400 MHz): δ_H 8.16 (d, *J* = 7.6 Hz, 2H), 7.97 (d, *J* = 7.6 Hz, 2H), 4.59 (s, 2H), 3.97 (s, 3H); ^{13}C{^{1}H} NMR ($CDCl_3$, 101 MHz): δ_C 192.8, 165.9, 137.5, 134.8, 130.1, 127.9, 55.1, 52.6; IR (KBr, cm^{-1}) ν: 2110, 1596.

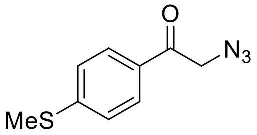

2-Azido-1-(4-(methylthio)phenyl)ethan-1-one (7f)[14]

To a 10 mL Schlenk tube equipped with a magnetic stir bar was added $TMSN_3$ **2** (85 μL, 0.65 mmol, 1.3 equiv), silyl enol ether **6f** (119.0 mg, 0.5 mmol, 1.0 equiv), DMSO (2.0 mL), $PhI(OAc)_2$ (322.1 mg, 1.0 mmol, 2 equiv). The reaction mixture was stirred at 25 °C in an oil bath for about 0.5 h. The resulting mixture was concentrated and the residue was taken up in ethyl acetate. The organic layer was washed with brine, dried over Na_2SO_4 and concentrated. Purification of the crude product by column chromatography (silica gel; petroleum ether/ethyl acetate = 18:1) afforded **7f** in 82% yield (85 mg). Faint white solid; mp 76–77 °C ; ^{1}H NMR ($CDCl_3$, 400 MHz): δ_H 7.81 (d, *J* = 8.0 Hz, 2H), 7.28 (d, *J* = 8.4 Hz, 2H), 4.52 (s, 2H), 2.53 (s, 3H); ^{13}C{^{1}H} NMR ($CDCl_3$, 101 MHz): δ_C 192.1, 147.5, 130.4, 128.2, 125.1, 54.6, 14.6; IR (KBr, cm^{-1}) ν: 2095, 1682.

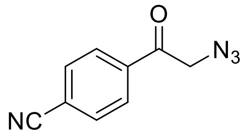

4-(2-Azidoacetyl)benzonitrile (7g)[15]

To a 10 mL Schlenk tube equipped with a magnetic stir bar was added $TMSN_3$ **2** (85 μL, 0.65 mmol, 1.3 equiv), silyl enol ether **6g** (119.0 mg, 0.5 mmol, 1.0 equiv), DMSO (2.0 mL), $PhI(OAc)_2$ (322.1 mg, 1.0 mmol, 2 equiv). The reaction mixture was stirred at 25 °C in an oil bath for about 0.5 h. The resulting mixture was concentrated and the residue was taken up in ethyl acetate. The organic layer was washed with brine, dried over Na_2SO_4 and concentrated. Purification of the crude product by column chromatography (silica gel; petroleum ether/ethyl acetate = 20:1) afforded **7g** in 74% yield (69 mg). Faint white solid; mp 82–83 °C; ^1H NMR ($CDCl_3$, 400 MHz): δ_H 8.02 (d, *J* = 8.0 Hz, 2H), 7.82 (d, *J* = 8.0 Hz, 2H), 4.57 (s, 2H); $^{13}C\{^1H\}$ NMR ($CDCl_3$, 101 MHz): δ_C 192.1, 137.2, 132.8, 128.4, 117.5, 55.1; IR (KBr, cm^{-1}) ν: 2106, 1597.

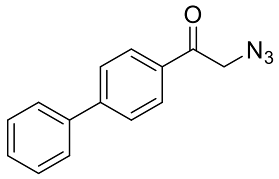

1-([1,1'-Biphenyl]-4-yl)-2-azidoethan-1-one (7h)[16]

To a 10 mL Schlenk tube equipped with a magnetic stir bar was added $TMSN_3$ **2** (85 μL, 0.65 mmol, 1.3 equiv), silyl enol ether **6h** (134.0 mg, 0.5 mmol, 1.0 equiv), DMSO (2.0 mL), $PhI(OAc)_2$ (322.1 mg, 1.0 mmol, 2 equiv). The reaction mixture was stirred at 25 °C in an oil bath for about 0.5 h. The resulting mixture was concentrated and the residue was taken up in ethyl acetate. The organic layer was washed with brine, dried over Na_2SO_4 and concentrated. Purification of the crude product by column chromatography (silica gel; petroleum ether/ethyl acetate = 20:1) afforded **7g** in 78% yield (92 mg). Faint white solid; mp 88–89 °C ; ^1H NMR ($CDCl_3$, 400 MHz): δ_H 7.97 (d, *J* = 8.0 Hz, 2H), 7.71 (d, *J* = 8.0 Hz, 2H), 7.62 (d, *J* = 8.0 Hz, 2H), 7.50–7.40 (m, 3H), 4.58 (s, 2H); $^{13}C\{^1H\}$ NMR ($CDCl_3$, 101 MHz): δ_C 192.8, 146.8, 139.4, 133.0, 129.0, 128.5, 127.5, 127.2, 54.9; IR (KBr, cm^{-1}) ν: 2098, 1683.

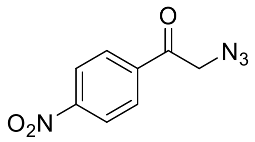

2-Azido-1-(4-nitrophenyl)ethan-1-one (7i)[17]

To a 10 mL Schlenk tube equipped with a magnetic stir bar was added $TMSN_3$ **2** (85 μL, 0.65 mmol, 1.3 equiv), silyl enol ether **6i** (118.5 mg, 0.5 mmol, 1.0 equiv), DMSO (2.0 mL), $PhI(OAc)_2$ (322.1 mg, 1.0 mmol, 2 equiv). The reaction mixture was stirred at 25 °C in an oil bath for about 0.5 h. The resulting mixture was concentrated and the residue was taken up in ethyl acetate. The organic layer was washed with brine, dried over Na_2SO_4 and concentrated. Purification of the crude product by column chromatography (silica gel; petroleum ether/ethyl acetate = 20:1) afforded **7i** in 71% yield (73 mg). Faint yellow solid; mp 78–79 °C; ^{1}H NMR ($CDCl_3$, 400 MHz): δ_H 8.36 (d, J = 8.0 Hz, 2H), 8.10 (d, J = 8.0 Hz, 2H), 4.63 (s, 2H); $^{13}C\{^1H\}$ NMR ($CDCl_3$, 101 MHz): δ_C 192.0, 150.8, 138.7, 129.1, 124.2, 55.2; IR (KBr, cm^{-1}) ν: 2113, 1704.

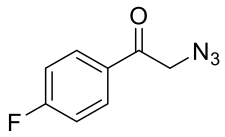

2-Azido-1-(4-fluorophenyl)ethan-1-one (7j)[10]

To a 10 mL Schlenk tube equipped with a magnetic stir bar was added $TMSN_3$ **2** (85 μL, 0.65 mmol, 1.3 equiv), silyl enol ether **6j** (105.0 mg, 0.5 mmol, 1.0 equiv), DMSO (2.0 mL), $PhI(OAc)_2$ (322.1 mg, 1.0 mmol, 2 equiv). The reaction mixture was stirred at 25 °C in an oil bath for about 0.5 h. The resulting mixture was concentrated and the residue was taken up in ethyl acetate. The organic layer was washed with brine, dried over Na_2SO_4 and concentrated. Purification of the crude product by column chromatography (silica gel; petroleum ether/ethyl acetate = 20:1) afforded **7j** in 75% yield (67 mg). Faint white solid; mp 49–50 °C; ^{1}H NMR ($CDCl_3$, 400 MHz): δ_H 7.97–7.94 (m, 2H), 7.20–7.16 (m, 2H), 4.54 (s, 2H); $^{13}C\{^1H\}$ NMR ($CDCl_3$, 101 MHz): δ_C 191.7, 166.2 (d, J_{C-F} = 258.1 Hz), 130.8 (d, J_{C-F} = 3.0 Hz), 130.6 (d, J_{C-F} = 9.6 Hz), 116.2 (d, J_{C-F} = 22.2 Hz), 54.7; IR (KBr, cm^{-1}) ν: 2106, 1602.

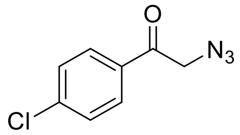

2-Azido-1-(4-chlorophenyl)ethan-1-one (7k)[10]

To a 10 mL Schlenk tube equipped with a magnetic stir bar was added $TMSN_3$ **2** (85 μL, 0.65 mmol, 1.3 equiv), silyl enol ether **6k** (113.0 mg, 0.5 mmol, 1.0 equiv), DMSO (2.0 mL), $PhI(OAc)_2$ (322.1 mg, 1.0 mmol, 2 equiv). The reaction mixture was stirred at 25 °C in an oil bath for about 0.5 h. The resulting mixture was concentrated and the residue was taken up in ethyl acetate. The organic layer was washed with brine, dried over Na_2SO_4 and concentrated. Purification of the crude product by column chromatography (silica gel; petroleum ether/ethyl acetate = 20:1) afforded **7k** in 72% yield (70 mg). Faint white solid; mp 67–68 °C; ^1H NMR ($CDCl_3$, 400 MHz): δ_H 7.86 (d, J = 7.2 Hz, 2H), 7.48 (d, J = 7.6 Hz, 2H), 4.53 (s, 2H); $^{13}C\{^1H\}$ NMR ($CDCl_3$, 101 MHz): δ_C 192.1, 140.7, 132.6, 129.34, 129.32, 54.8; IR (KBr, cm^{-1}) ν: 2107, 1601.

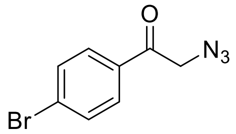

2-Azido-1-(4-bromophenyl)ethan-1-one (7l)[10]

To a 10 mL Schlenk tube equipped with a magnetic stir bar was added $TMSN_3$ **2** (85 μL, 0.65 mmol, 1.3 equiv), silyl enol ether **6l** (135.0 mg, 0.5 mmol, 1.0 equiv), DMSO (2.0 mL), $PhI(OAc)_2$ (322.1 mg, 1.0 mmol, 2 equiv). The reaction mixture was stirred at 25 °C in an oil bath for about 0.5 h. The resulting mixture was concentrated and the residue was taken up in ethyl acetate. The organic layer was washed with brine, dried over Na_2SO_4 and concentrated. Purification of the crude product by column chromatography (silica gel; petroleum ether/ethyl acetate = 20:1) afforded **7l** in 73% yield (87 mg). Faint white solid; mp 80–81 °C; ^1H NMR ($CDCl_3$, 400 MHz): δ_H 7.78 (d, J = 8.4 Hz, 2H), 7.65 (d, J = 8.4 Hz, 2H), 4.53 (s, 2H); $^{13}C\{^1H\}$ NMR ($CDCl_3$, 101 MHz): δ_C 192.3, 133.0, 132.3, 129.5, 129.4, 54.8; IR (KBr, cm^{-1}) ν: 2105, 1600.

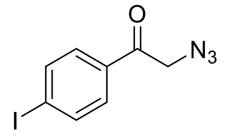

2-Azido-1-(4-iodophenyl)ethan-1-one (7m)[17]

To a 10 mL Schlenk tube equipped with a magnetic stir bar was added $TMSN_3$ **2** (85 µL, 0.65 mmol, 1.3 equiv), silyl enol ether **6m** (159.0 mg, 0.5 mmol, 1.0 equiv), DMSO (2.0 mL), $PhI(OAc)_2$ (322.1 mg, 1.0 mmol, 2 equiv). The reaction mixture was stirred at 25 °C in an oil bath for about 0.5 h. The resulting mixture was concentrated and the residue was taken up in ethyl acetate. The organic layer was washed with brine, dried over Na_2SO_4 and concentrated. Purification of the crude product by column chromatography (silica gel; petroleum ether/ethyl acetate = 20:1) afforded **7m** in 70% yield (100 mg). Faint white solid; mp 89–90 °C; ^1H NMR ($CDCl_3$, 400 MHz): δ_H 7.88 (d, J = 7.6 Hz, 2H), 7.62 (d, J = 7.6 Hz, 2H), 4.52 (s, 2H); $^{13}C\{^1H\}$ NMR ($CDCl_3$, 101 MHz): δ_C 192.6, 138.3, 133.6, 129.2, 102.3, 54.7; IR (KBr, cm^{-1}) ν: 2103, 1602.

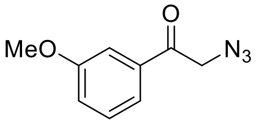

2-Azido-1-(3-methoxyphenyl)ethan-1-one (7n)[18]

To a 10 mL Schlenk tube equipped with a magnetic stir bar was added $TMSN_3$ **2** (85 µL, 0.65 mmol, 1.3 equiv), silyl enol ether **6n** (111.0 mg, 0.5 mmol, 1.0 equiv), DMSO (2.0 mL), $PhI(OAc)_2$ (322.1 mg, 1.0 mmol, 2 equiv). The reaction mixture was stirred at 25 °C in an oil bath for about 0.5 h. The resulting mixture was concentrated and the residue was taken up in ethyl acetate. The organic layer was washed with brine, dried over Na_2SO_4 and concentrated. Purification of the crude product by column chromatography (silica gel; petroleum ether/ethyl acetate = 15:1) afforded **7n** in 86% yield (82 mg). Faint colorless oil; ^1H NMR ($CDCl_3$, 400 MHz): δ_H 7.46–7.38 (m, 3H), 7.18–7.16 (m, 1H), 4.55 (s, 2H), 3.87 (s, 3H); $^{13}C\{^1H\}$ NMR ($CDCl_3$, 101 MHz): δ_C 193.1, 160.0, 135.7, 130.0, 120.6, 120.3, 112.2, 55.5, 54.9; IR (KBr, cm^{-1}) ν: 2105, 1632.

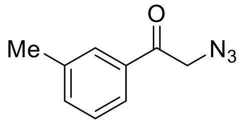

2-Azido-1-(*m*-tolyl)ethan-1-one (7o)[15]

To a 10 mL Schlenk tube equipped with a magnetic stir bar was added $TMSN_3$ **2** (85 µL, 0.65 mmol, 1.3 equiv), silyl enol ether **6o** (103.0 mg, 0.5 mmol, 1.0 equiv), DMSO (2.0 mL), $PhI(OAc)_2$ (322.1 mg, 1.0 mmol, 2 equiv). The reaction mixture was stirred at 25 °C in an oil bath for about 0.5 h. The resulting mixture was concentrated and the residue was taken up in ethyl acetate. The organic layer was washed with brine, dried over Na_2SO_4 and concentrated. Purification of the crude product by column chromatography (silica gel; petroleum ether/ethyl acetate = 20:1) afforded **7o** in 83% yield (73 mg). Faint colorless oil; ^{1}H NMR ($CDCl_3$, 400 MHz): δ_H 7.73–7.68 (m, 2H), 7.45–7.36 (m, 2H), 4.56 (s, 2H), 2.43 (s, 3H); ^{13}C{^{1}H} NMR ($CDCl_3$, 101 MHz): δ_C 193.3, 138.9, 134.9, 134.4, 128.8, 128.4, 125.1, 54.9, 21.3; IR (KBr, cm^{-1}) ν: 2109, 1594.

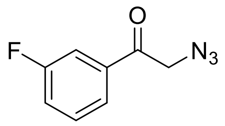

2-Azido-1-(3-fluorophenyl)ethan-1-one (7p)[15]

To a 10 mL Schlenk tube equipped with a magnetic stir bar was added $TMSN_3$ **2** (85 µL, 0.65 mmol, 1.3 equiv), silyl enol ether **6p** (105.0 mg, 0.5 mmol, 1.0 equiv), DMSO (2.0 mL), $PhI(OAc)_2$ (322.1 mg, 1.0 mmol, 2 equiv). The reaction mixture was stirred at 25 °C in an oil bath for about 0.5 h. The resulting mixture was concentrated and the residue was taken up in ethyl acetate. The organic layer was washed with brine, dried over Na_2SO_4 and concentrated. Purification of the crude product by column chromatography (silica gel; petroleum ether/ethyl acetate = 20:1) afforded **7p** in 73% yield (65 mg). Faint colorless oil; ^{1}H NMR ($CDCl_3$, 400 MHz): δ_H 7.69 (d, J = 7.2 Hz, 1H), 7.62 (d, J = 8.8 Hz, 1H), 7.51–7.50 (m, 1H), 7.36–7.34 (m, 1H), 4.55 (s, 2H); ^{13}C{^{1}H} NMR ($CDCl_3$, 101 MHz): δ_C 192.1(d, J_{C-F} = 1.51 Hz), 162.9 (d, J_{C-F} = 250.5 Hz), 136.3 (d, J_{C-F} = 6.3 Hz), 130.7 (d, J_{C-F} = 7.7 Hz), 123.6 (d, J_{C-F} = 2.9 Hz), 54.7; IR (KBr, cm^{-1}) ν: 2109, 1593.

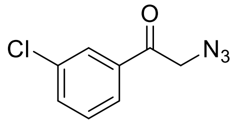

2-Azido-1-(3-chlorophenyl)ethan-1-one (7q)[15]

To a 10 mL Schlenk tube equipped with a magnetic stir bar was added $TMSN_3$ **2** (85 µL, 0.65 mmol, 1.3 equiv), silyl enol ether **6q** (113.0 mg, 0.5 mmol, 1.0 equiv), DMSO (2.0 mL), $PhI(OAc)_2$ (322.1 mg, 1.0 mmol, 2 equiv). The reaction mixture was stirred at 25 °C in an oil bath for about 0.5 h. The resulting mixture was concentrated and the residue was taken up in ethyl acetate. The organic layer was washed with brine, dried over Na_2SO_4 and concentrated. Purification of the crude product by column chromatography (silica gel; petroleum ether/ethyl acetate = 20:1) afforded **7q** in 71% yield (69 mg). Faint white solid; mp 65–66 °C; ^{1}H NMR ($CDCl_3$, 400 MHz): δ_H 7.89 (s, 1H), 7.78 (d, J = 7.6 Hz, 1H), 7.61 (d, J = 8.0 Hz, 1H), 7.48–7.44 (m, 1H), 4.54 (s, 2H); ^{13}C{^{1}H} NMR ($CDCl_3$, 101 MHz): δ_C 192.1, 135.8, 135.4, 134.1, 130.3, 128.1, 126.0, 54.9; IR (KBr, cm^{-1}) ν: 2106, 1594.

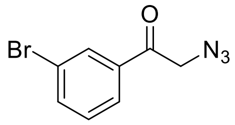

2-Azido-1-(3-bromophenyl)ethan-1-one (7r)[19]

To a 10 mL Schlenk tube equipped with a magnetic stir bar was added $TMSN_3$ **2** (85 µL, 0.65 mmol, 1.3 equiv), silyl enol ether **6r** (135.0 mg, 0.5 mmol, 1.0 equiv), DMSO (2.0 mL), $PhI(OAc)_2$ (322.1 mg, 1.0 mmol, 2 equiv). The reaction mixture was stirred at 25 °C in an oil bath for about 0.5 h. The resulting mixture was concentrated and the residue was taken up in ethyl acetate. The organic layer was washed with brine, dried over Na_2SO_4 and concentrated. Purification of the crude product by column chromatography (silica gel; petroleum ether/ethyl acetate = 20:1) afforded **7r** in 72% yield (86 mg). Faint white solid; mp 51–52 °C; ^{1}H NMR ($CDCl_3$, 400 MHz): δ_H 8.05 (s, 1H), 7.83 (d, J = 7.6 Hz, 1H), 7.76 (d, J = 8.0 Hz, 1H), 7.41–7.37 (m, 1H), 4.54 (s, 2H); ^{13}C{^{1}H} NMR ($CDCl_3$, 101 MHz): δ_C 192.0, 137.0, 136.0, 131.0, 130.5, 126.4, 123.3, 54.9; IR (KBr, cm^{-1}) ν: 2104, 1632.

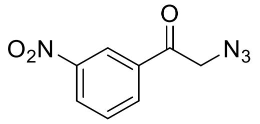

2-Azido-1-(3-nitrophenyl)ethan-1-one (7s)[17]

To a 10 mL Schlenk tube equipped with a magnetic stir bar was added $TMSN_3$ **2** (85 µL, 0.65 mmol, 1.3 equiv), silyl enol ether **6s** (118.5 mg, 0.5 mmol, 1.0 equiv), DMSO (2.0 mL), $PhI(OAc)_2$ (322.1 mg, 1.0 mmol, 2 equiv). The reaction mixture was stirred at 25 °C in an oil bath for about 0.5 h. The resulting mixture was concentrated and the residue was taken up in ethyl acetate. The organic layer was washed with brine, dried over Na_2SO_4 and concentrated. Purification of the crude product by column chromatography (silica gel; petroleum ether/ethyl acetate = 20:1) afforded **7s** in 68% yield (70 mg). Faint white solid; mp 52–53 °C; ^{1}H NMR ($CDCl_3$, 400 MHz): δ_H 8.77 (s, 1H), 8.53 (d, J = 8.4 Hz, 1H), 8.31 (d, J = 7.6 Hz, 1H), 7.81–7.77 (m, 1H), 4.68 (s, 2H); $^{13}C\{^1H\}$ NMR ($CDCl_3$, 101 MHz): δ_C 191.4, 148.5, 135.5, 133.5, 130.4, 128.3, 122.9, 55.1; IR (KBr, cm^{-1}) ν: 2108, 1613.

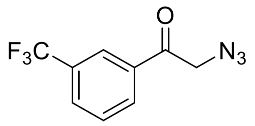

2-Azido-1-(3-nitrophenyl)ethan-1-one (7t)[20]

To a 10 mL Schlenk tube equipped with a magnetic stir bar was added $TMSN_3$ **2** (85 µL, 0.65 mmol, 1.3 equiv), silyl enol ether **6t** (130.0 mg, 0.5 mmol, 1.0 equiv), DMSO (2.0 mL), $PhI(OAc)_2$ (322.1 mg, 1.0 mmol, 2 equiv). The reaction mixture was stirred at 25 °C in an oil bath for about 0.5 h. The resulting mixture was concentrated and the residue was taken up in ethyl acetate. The organic layer was washed with brine, dried over Na_2SO_4 and concentrated. Purification of the crude product by column chromatography (silica gel; petroleum ether/ethyl acetate = 20:1) afforded **7t** in 60% yield (69 mg). Faint colorless oil; ^{1}H NMR ($CDCl_3$, 400 MHz): δ_H 8.17 (s, 1H), 8.10 (d, J = 7.6 Hz, 1H), 7.89 (d, J = 8.0 Hz, 1H), 7.69–7.67 (m, 1H), 4.60 (s, 2H); $^{13}C\{^1H\}$ NMR ($CDCl_3$, 101 MHz): δ_C 192.1, 134.9, 131.7 (d, $J_{C–F}$ = 33.2 Hz), 131.1, 130.5 (q, $J_{C–F}$ = 3.4 Hz), 129.7, 124.8 (t, $J_{C–F}$ = 3.7 Hz), 122.1, 55.0; IR (KBr, cm^{-1}) ν: 2108, 1704.

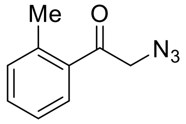

2-Azido-1-(*o*-tolyl)ethan-1-one (7u)[15]

To a 10 mL Schlenk tube equipped with a magnetic stir bar was added $TMSN_3$ **2** (85 µL, 0.65 mmol, 1.3 equiv), silyl enol ether **6u** (103.0 mg, 0.5 mmol, 1.0 equiv), DMSO (2.0 mL), $PhI(OAc)_2$ (322.1 mg, 1.0 mmol, 2 equiv). The reaction mixture was stirred at 25 °C in an oil bath for about 0.5 h. The resulting mixture was concentrated and the residue was taken up in ethyl acetate. The organic layer was washed with brine, dried over Na_2SO_4 and concentrated. Purification of the crude product by column chromatography (silica gel; petroleum ether/ethyl acetate = 20:1) afforded **7u** in 58% yield (51 mg).

Faint colorless oil; ^1H NMR ($CDCl_3$, 400 MHz): δ_H 7.57 (d, J = 7.6 Hz, 1H), 7.47–7.43 (m, 1H), 7.31 (d, J = 8.8 Hz, 2H), 4.46 (s, 2H), 2.56 (s, 3H); $^{13}C\{^1H\}$ NMR ($CDCl_3$, 101 MHz): δ_C 196.3, 139.5, 134.4, 132.52, 132.50, 128.3, 125.9, 56.4, 21.5; IR (KBr, cm^{-1}) ν: 2105, 1632.

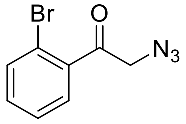

2-Azido-1-(2-bromophenyl)ethan-1-one (7v)[21]

To a 10 mL Schlenk tube equipped with a magnetic stir bar was added $TMSN_3$ **2** (85 µL, 0.65 mmol, 1.3 equiv), silyl enol ether **6v** (135.0 mg, 0.5 mmol, 1.0 equiv), DMSO (2.0 mL), $PhI(OAc)_2$ (322.1 mg, 1.0 mmol, 2 equiv). The reaction mixture was stirred at 25 °C in an oil bath for about 0.5 h. The resulting mixture was concentrated and the residue was taken up in ethyl acetate. The organic layer was washed with brine, dried over Na_2SO_4 and concentrated. Purification of the crude product by column chromatography (silica gel; petroleum ether/ethyl acetate = 20:1) afforded **7v** in 55% yield (66 mg).

Faint colorless oil; ^1H NMR ($CDCl_3$, 400 MHz): δ_H 7.65 (d, J = 7.6 Hz, 1H), 7.46–7.36 (m, 3H), 4.48 (s, 2H); $^{13}C\{^1H\}$ NMR ($CDCl_3$, 101 MHz): δ_C 197.3, 138.5, 133.9, 132.7, 129.1, 127.7, 119.2, 57.3; IR (KBr, cm^{-1}) ν: 2105, 1640.

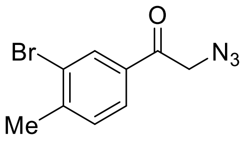

2-Azido-1-(3-bromo-4-methylphenyl)ethan-1-one (7w)

To a 10 mL Schlenk tube equipped with a magnetic stir bar was added $TMSN_3$ **2** (85 µL, 0.65 mmol, 1.3 equiv), silyl enol ether **6w** (142.0 mg, 0.5 mmol, 1.0 equiv), DMSO (2.0 mL), $PhI(OAc)_2$ (322.1 mg, 1.0 mmol, 2 equiv). The reaction mixture was stirred at 25 °C in an oil bath for about 0.5 h. The resulting mixture was concentrated and the residue was taken up in ethyl acetate. The organic layer was washed with brine, dried over Na_2SO_4 and concentrated. Purification of the crude product by column chromatography (silica gel; petroleum ether/ethyl acetate = 20:1) afforded **7w** in 69% yield (87 mg). Faint white solid; mp 80–81 °C; ^{1}H NMR ($CDCl_3$, 400 MHz): δ_H 8.07 (s, 1H), 7.74 (d, *J* = 8.0 Hz, 1H), 7.36 (d, *J* = 8.0 Hz, 1H), 4.52 (s, 2H), 2.47 (s, 3H); $^{13}C\{^1H\}$ NMR ($CDCl_3$, 101 MHz): δ_C 191.7, 144.9, 133.6, 131.9, 131.2, 126.6, 125.6, 54.8, 23.3; IR (KBr, cm^{-1}) ν: 2110, 1688.

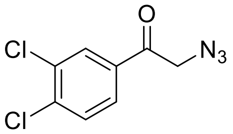

2-Azido-1-(3,4-dichlorophenyl)ethan-1-one (7x)[20]

To a 10 mL Schlenk tube equipped with a magnetic stir bar was added $TMSN_3$ **2** (85 µL, 0.65 mmol, 1.3 equiv), silyl enol ether **6x** (130.0 mg, 0.5 mmol, 1.0 equiv), DMSO (2.0 mL), $PhI(OAc)_2$ (322.1 mg, 1.0 mmol, 2 equiv). The reaction mixture was stirred at 25 °C in an oil bath for about 0.5 h. The resulting mixture was concentrated and the residue was taken up in ethyl acetate. The organic layer was washed with brine, dried over Na_2SO_4 and concentrated. Purification of the crude product by column chromatography (silica gel; petroleum ether/ethyl acetate = 20:1) afforded **7x** in 65% yield (74 mg). Faint colorless oil; ^{1}H NMR ($CDCl_3$, 400 MHz): δ_H 8.00 (s, 1H), 7.74 (d, *J* = 8.4 Hz, 1H), 7.60 (d, *J* = 8.4 Hz, 1H), 4.52 (s, 2H); $^{13}C\{^1H\}$ NMR ($CDCl_3$, 101 MHz): δ_C 191.2, 138.9, 133.9, 133.8, 131.1, 130.0, 126.9, 54.9; IR (KBr, cm^{-1}) ν: 2114, 1683.

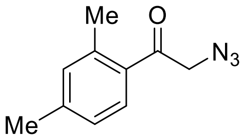

2-Azido-1-(2,4-dimethylphenyl)ethan-1-one (7y)

To a 10 mL Schlenk tube equipped with a magnetic stir bar was added $TMSN_3$ **2** (85 µL, 0.65 mmol, 1.3 equiv), silyl enol ether **6y** (110.0 mg, 0.5 mmol, 1.0 equiv), DMSO (2.0 mL), $PhI(OAc)_2$ (322.1 mg, 1.0 mmol, 2 equiv). The reaction mixture was stirred at 25 °C in an oil bath for about 0.5 h. The resulting mixture was concentrated and the residue was taken up in ethyl acetate. The organic layer was washed with brine, dried over Na_2SO_4 and concentrated. Purification of the crude product by column chromatography (silica gel; petroleum ether/ethyl acetate = 20:1) afforded **7y** in 67% yield (63 mg).

Faint colorless oil; ^1H NMR ($CDCl_3$, 400 MHz): δ_H 7.50 (d, J = 8.0 Hz, 2H), 7.11–7.08 (m, 2H), 4.44 (s, 2H), 2.54 (s, 3H), 2.37 (s, 3H); $^{13}C\{^1H\}$ NMR ($CDCl_3$, 101 MHz): δ_C 195.4, 143.4, 140.0, 133.3, 131.3, 128.8, 126.5, 56.1, 21.7, 21.4; HRMS (ESI-TOF, *m/z*): calcd for $C_{10}H_{12}N_3O$ $[M + H]^+$, 190.0975; found, 190.0986; IR (KBr, cm^{-1}) ν: 2105, 1690.

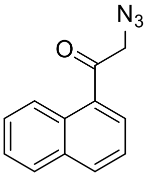

2-Azido-1-(naphthalen-1-yl)ethan-1-one (7z)[10]

To a 10 mL Schlenk tube equipped with a magnetic stir bar was added $TMSN_3$ **2** (85 µL, 0.65 mmol, 1.3 equiv), silyl enol ether **6z** (121.0 mg, 0.5 mmol, 1.0 equiv), DMSO (2.0 mL), $PhI(OAc)_2$ (322.1 mg, 1.0 mmol, 2 equiv). The reaction mixture was stirred at 25 °C in an oil bath for about 0.5 h. The resulting mixture was concentrated and the residue was taken up in ethyl acetate. The organic layer was washed with brine, dried over Na_2SO_4 and concentrated. Purification of the crude product by column chromatography (silica gel; petroleum ether/ethyl acetate = 20:1) afforded **7z** in 75% yield (79 mg).

Faint colorless oil; ^1H NMR ($CDCl_3$, 400 MHz): δ_H 8.73 (d, J = 8.4 Hz, 1H), 8.05 (d, J = 8.4 Hz, 1H), 7.89 (d, J = 8.0 Hz, 1H), 7.81 (d, J = 7.2 Hz, 1H), 7.66–7.48 (m, 3H), 4.58 (s, 2H); $^{13}C\{^1H\}$ NMR ($CDCl_3$, 101 MHz): δ_C 196.6, 134.1, 134.0, 132.2, 130.2, 128.62, 128.57, 128.0, 126.9, 125.5, 124.2, 56.7; IR (KBr, cm^{-1}) ν: 2105, 1605.

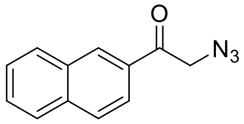

2-Azido-1-(naphthalen-2-yl)ethan-1-one (7aa)[16]

To a 10 mL Schlenk tube equipped with a magnetic stir bar was added $TMSN_3$ **2** (85 µL, 0.65 mmol, 1.3 equiv), silyl enol ether **6aa** (121.0 mg, 0.5 mmol, 1.0 equiv), DMSO (2.0 mL), $PhI(OAc)_2$ (322.1 mg, 1.0 mmol, 2 equiv). The reaction mixture was stirred at 25 °C in an oil bath for about 0.5 h. The resulting mixture was concentrated and the residue was taken up in ethyl acetate. The organic layer was washed with brine, dried over Na_2SO_4 and concentrated. Purification of the crude product by column chromatography (silica gel; petroleum ether/ethyl acetate = 20:1) afforded **7aa** in 78% yield (82 mg). Faint white solid; mp 66–67 °C; ^{1}H NMR ($CDCl_3$, 400 MHz): δ_H 8.37 (s, 1H), 7.96–7.87 (m, 4H), 7.65–7.56 (m, 2H), 4.67 (s, 2H); ^{13}C{^{1}H} NMR ($CDCl_3$, 101 MHz): δ_C 193.1, 135.9, 132.3, 131.6, 129.8, 129.6, 129.0, 128.9, 127.9, 127.2, 123.3, 54.9; IR (KBr, cm^{-1}) ν: 2103, 1677.

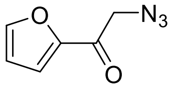

2-Azido-1-(furan-2-yl)ethan-1-one (7ab)[22]

To a 10 mL Schlenk tube equipped with a magnetic stir bar was added $TMSN_3$ **2** (85 µL, 0.65 mmol, 1.3 equiv), silyl enol ether **6ab** (91.0 mg, 0.5 mmol, 1.0 equiv), DMSO (2.0 mL), $PhI(OAc)_2$ (322.1 mg, 1.0 mmol, 2 equiv). The reaction mixture was stirred at 25 °C in an oil bath for about 0.5 h. The resulting mixture was concentrated and the residue was taken up in ethyl acetate. The organic layer was washed with brine, dried over Na_2SO_4 and concentrated. Purification of the crude product by column chromatography (silica gel; petroleum ether/ethyl acetate = 20:1) afforded **7ab** in 81% yield (61 mg). Faint white solid; mp 32–33 °C; ^{1}H NMR ($CDCl_3$, 400 MHz): δ_H 7.75–7.74 (m, 2H), 7.19–7.17 (m, 1H), 4.47 (s, 2H); ^{13}C{^{1}H} NMR ($CDCl_3$, 101 MHz): δ_C 186.2, 140.6, 134.9, 132.4, 128.4, 54.8; IR (KBr, cm^{-1}) ν: 2109, 1659.

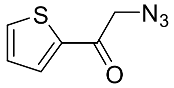

2-Azido-1-(thiophen-2-yl)ethan-1-one (7ac)[16]

To a 10 mL Schlenk tube equipped with a magnetic stir bar was added $TMSN_3$ **2** (85 μL, 0.65 mmol, 1.3 equiv), silyl enol ether **6ac** (99.0 mg, 0.5 mmol, 1.0 equiv), DMSO (2.0 mL), $PhI(OAc)_2$ (322.1 mg, 1.0 mmol, 2 equiv). The reaction mixture was stirred at 25 °C in an oil bath for about 0.5 h. The resulting mixture was concentrated and the residue was taken up in ethyl acetate. The organic layer was washed with brine, dried over Na_2SO_4 and concentrated. Purification of the crude product by column chromatography (silica gel; petroleum ether/ethyl acetate = 20:1) afforded **7ac** in 79% yield (66 mg). Faint white solid; mp 62–63 °C; ^1H NMR ($CDCl_3$, 400 MHz): δ_H 7.74–7.73 (m, 2H), 7.19–7.17 (m, 1H), 4.46 (s, 2H); $^{13}C\{^1H\}$ NMR ($CDCl_3$, 101 MHz): δ_C 186.2, 140.6, 134.9, 132.4, 128.4, 54.8; IR (KBr, cm^{-1}) ν: 2105, 1640.

V. NMR spectra

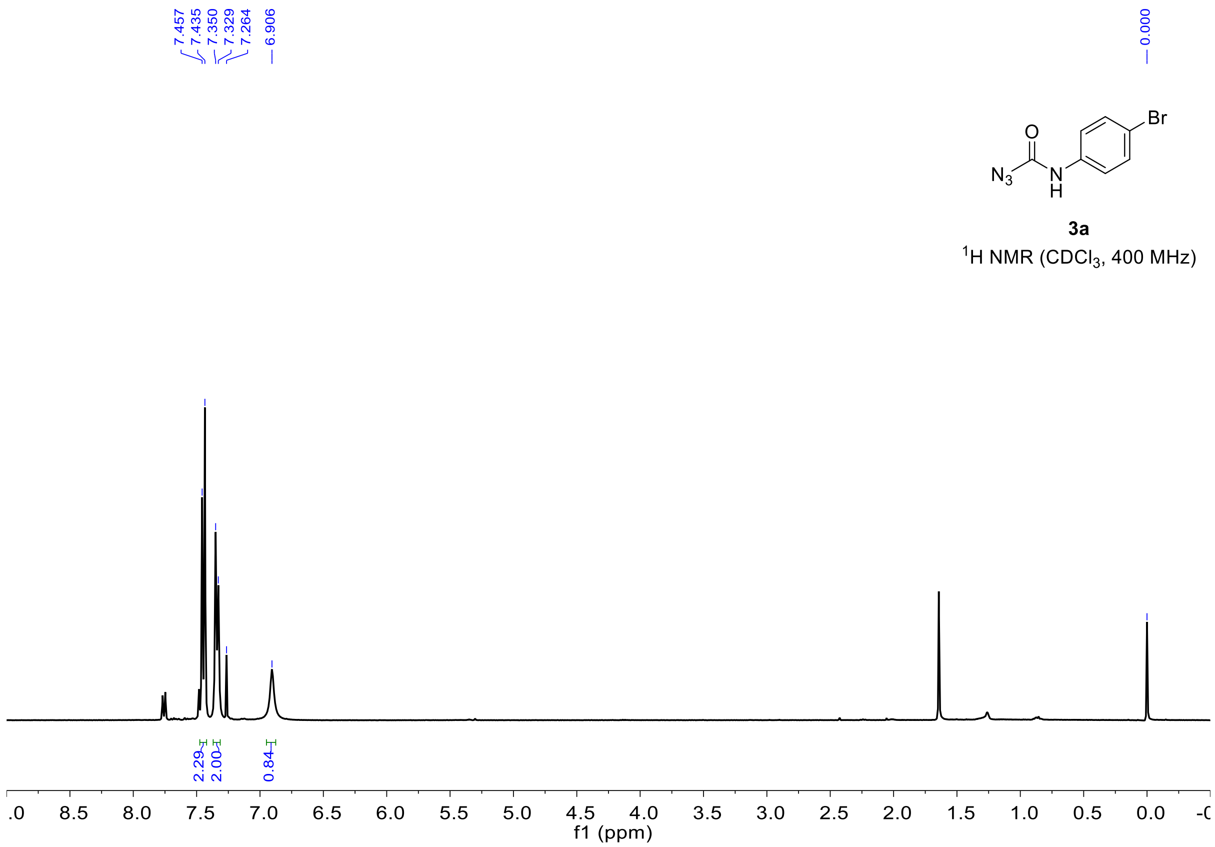

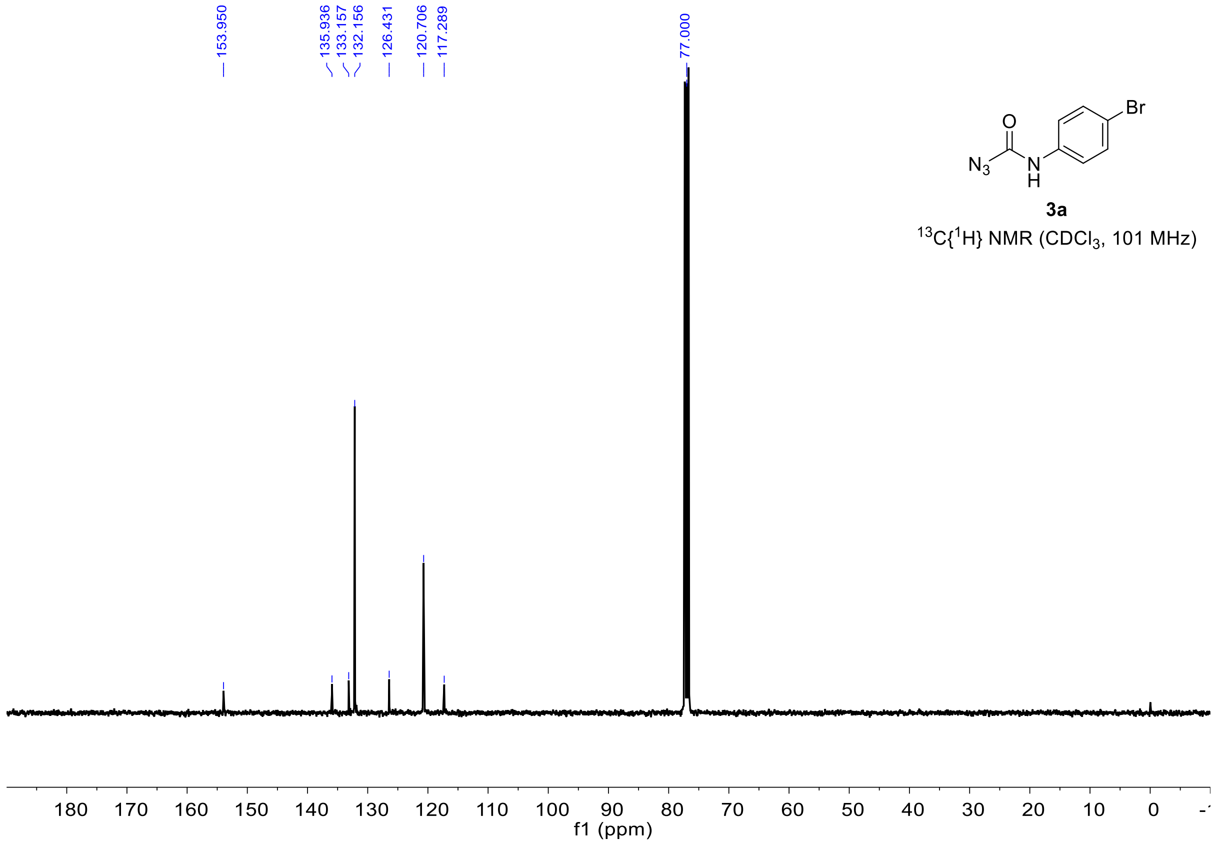

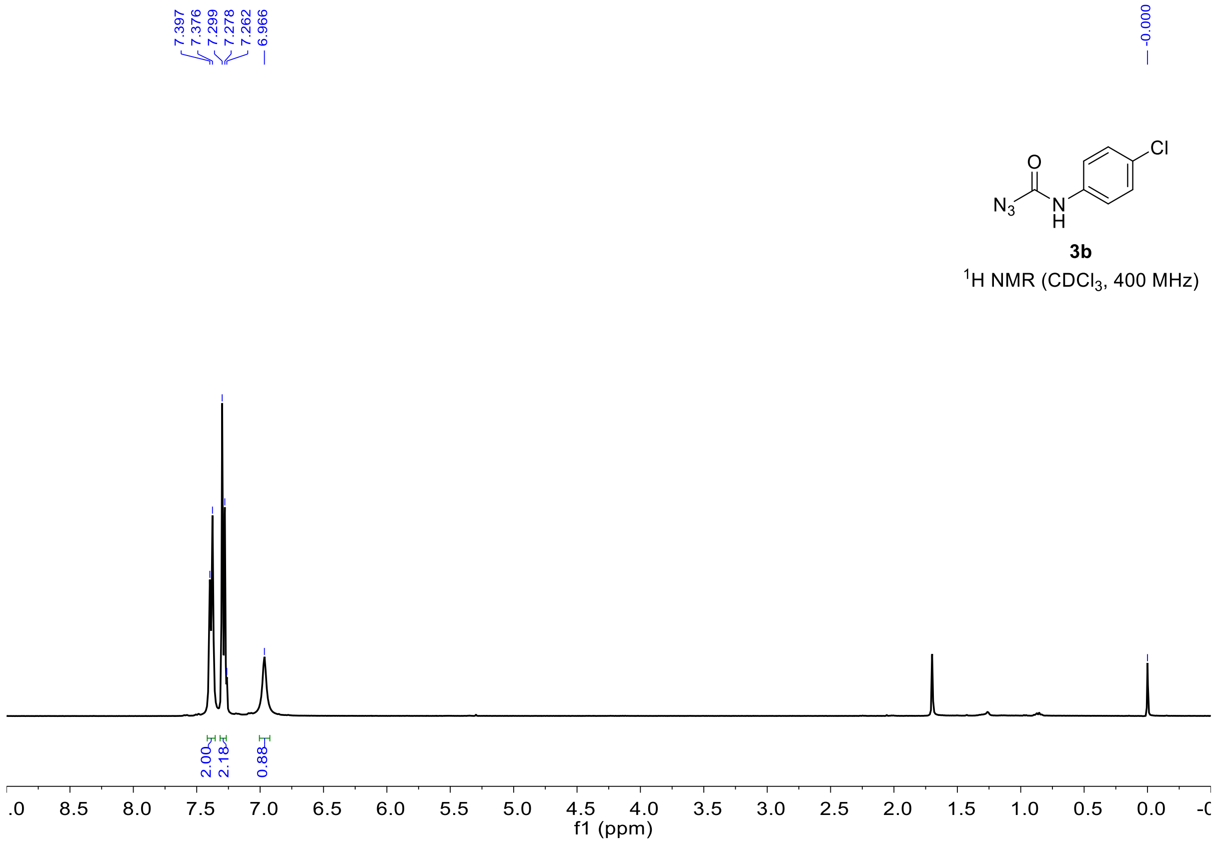
7.397
7.376
7.299
7.278
7.262
6.966
-0.000
O
Cl
N3
N
H
3b
1H NMR (CDCl3, 400 MHz)
2.00
2.18
0.88
.0 8.5 8.0 7.5 7.0 6.5 6.0 5.5 5.0 4.5 4.0 3.5 3.0 2.5 2.0 1.5 1.0 0.5 0.0
f1 (ppm)

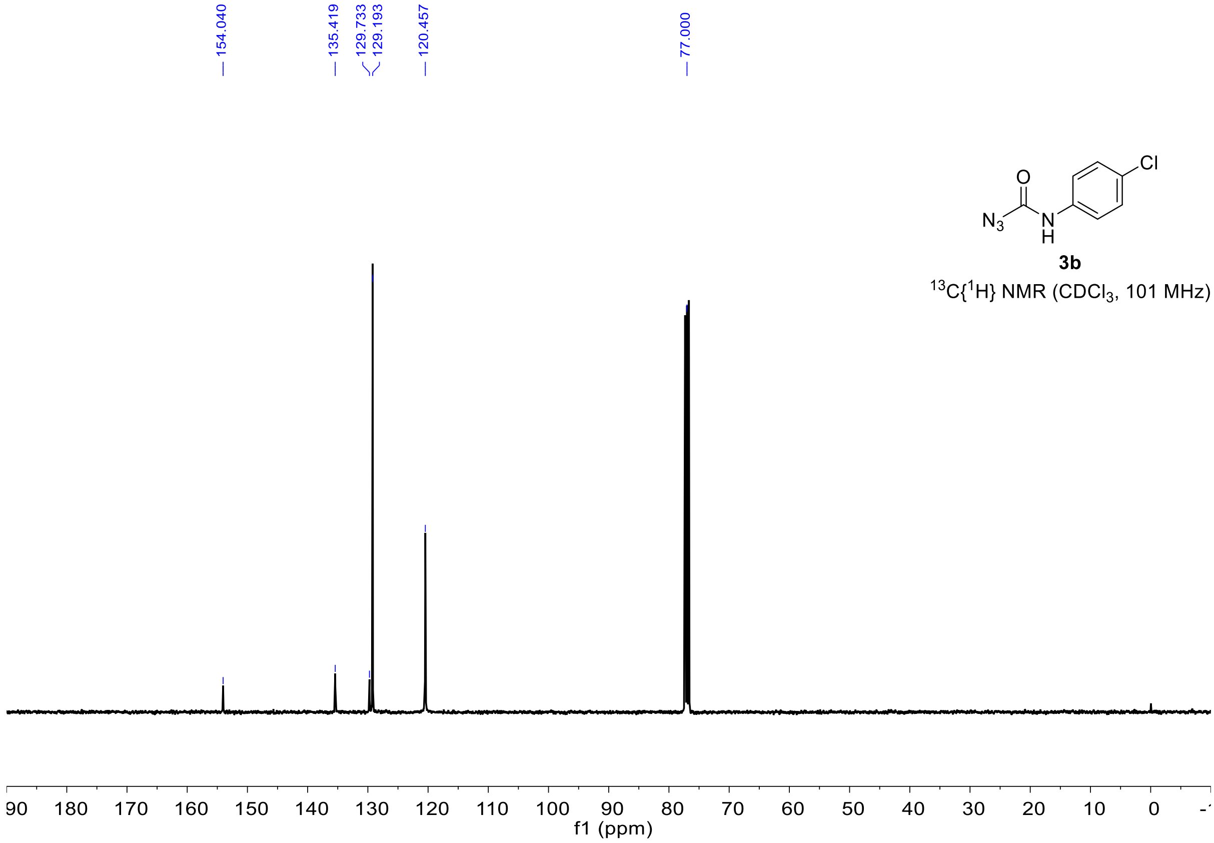
154.040
135.419
129.733
129.193
120.457
77.000
O
Cl
N3
N
H
3b
13C{1H} NMR (CDCl3, 101 MHz)
90 180 170 160 150 140 130 120 110 100 90 80 70 60 50 40 30 20 10 0
f1 (ppm)

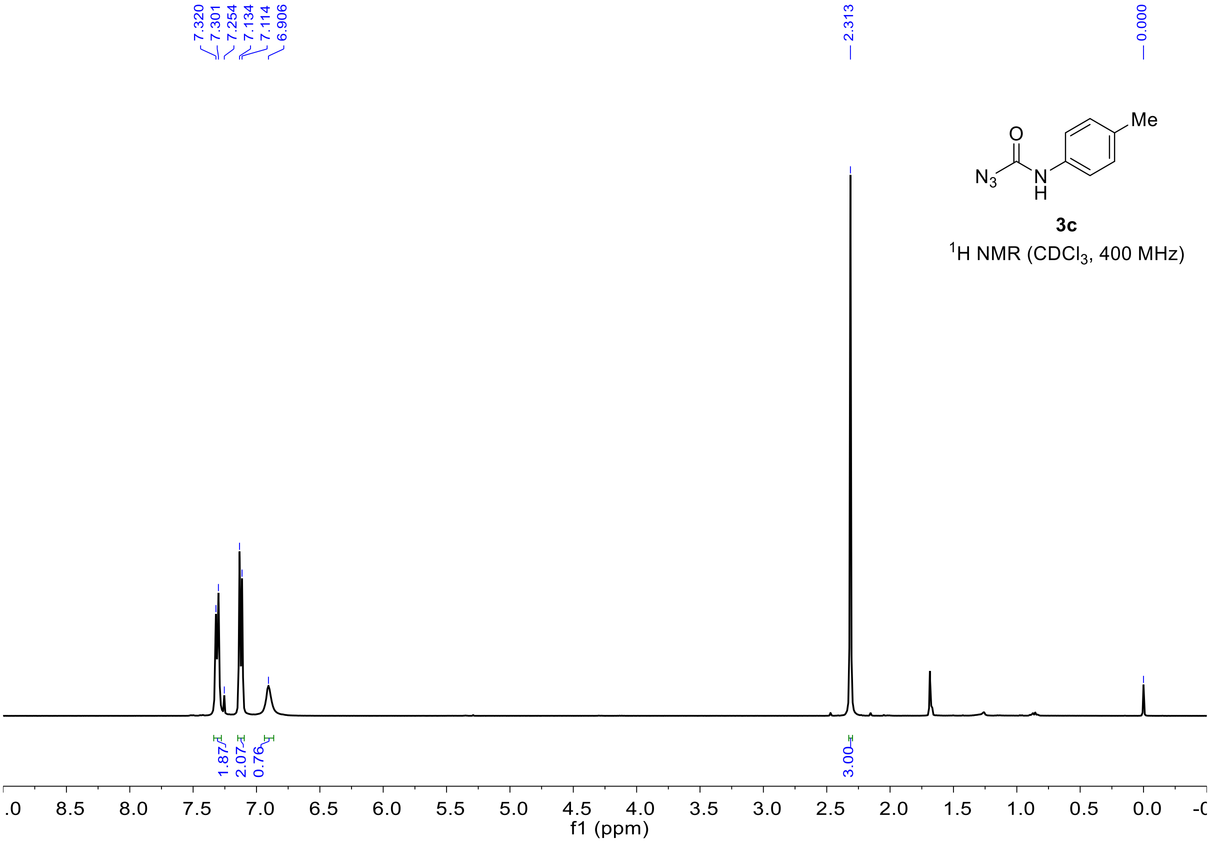
7.320
7.301
7.254
7.134
7.114
6.906
2.313
0.000
Me
O
N_3
N
H
3c
^1H NMR ($CDCl_3$, 400 MHz)
1.87
2.07
0.76
3.00
f1 (ppm)

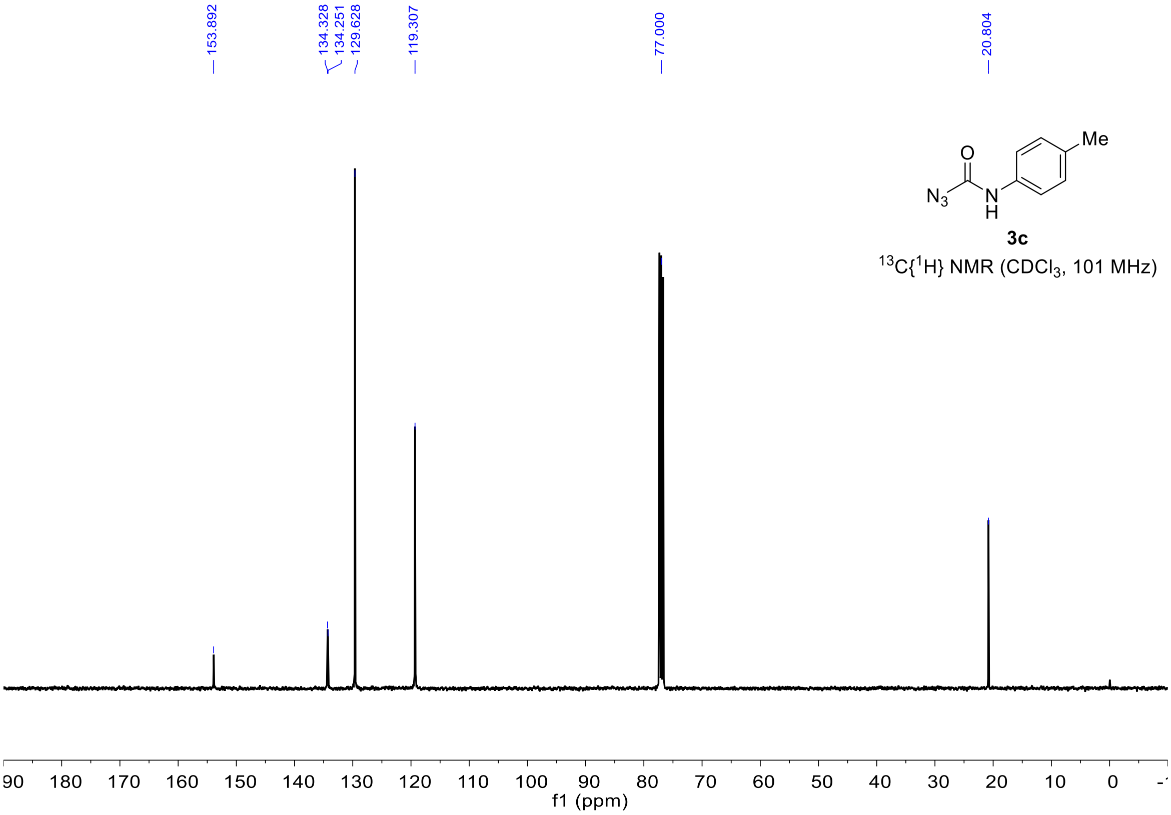
153.892
134.328
134.251
129.628
119.307
77.000
20.804
Me
O
N_3
N
H
3c
$^{13}C\{^1H\}$ NMR ($CDCl_3$, 101 MHz)
f1 (ppm)

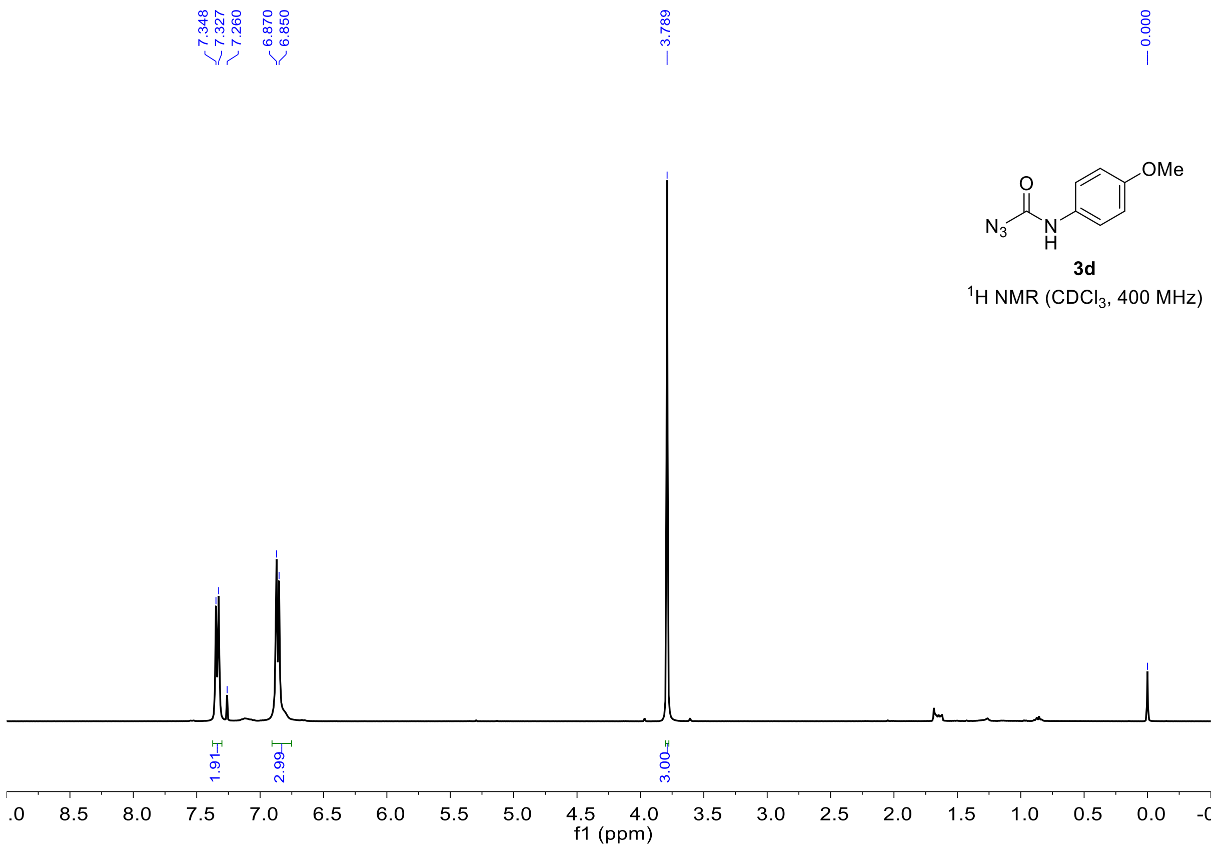
7.348
7.327
7.260
6.870
6.850
3.789
0.000
OMe
O
N3
N
H
3d
1H NMR (CDCl3, 400 MHz)
1.91
2.99
3.00
f1 (ppm)

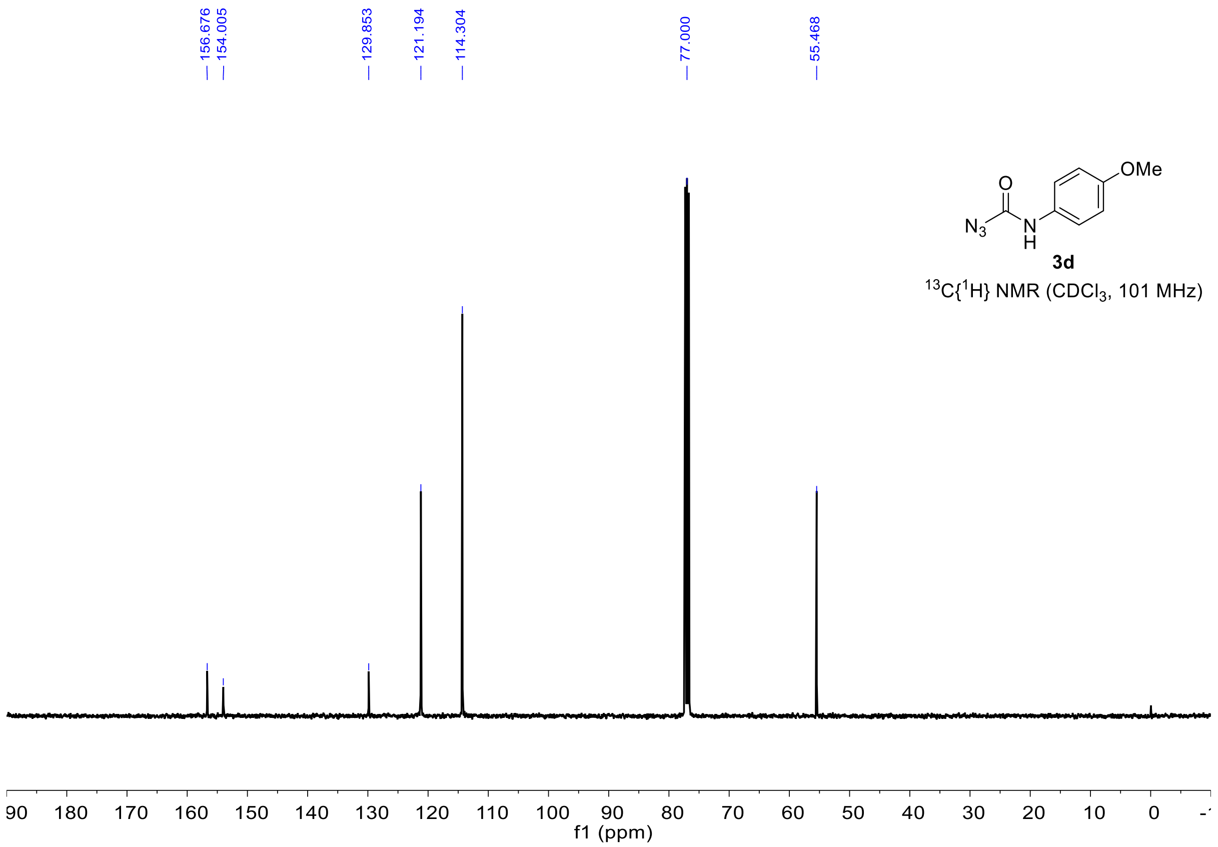
156.676
154.005
129.853
121.194
114.304
77.000
55.468
OMe
O
N3
N
H
3d
13C{1H} NMR (CDCl3, 101 MHz)
f1 (ppm)

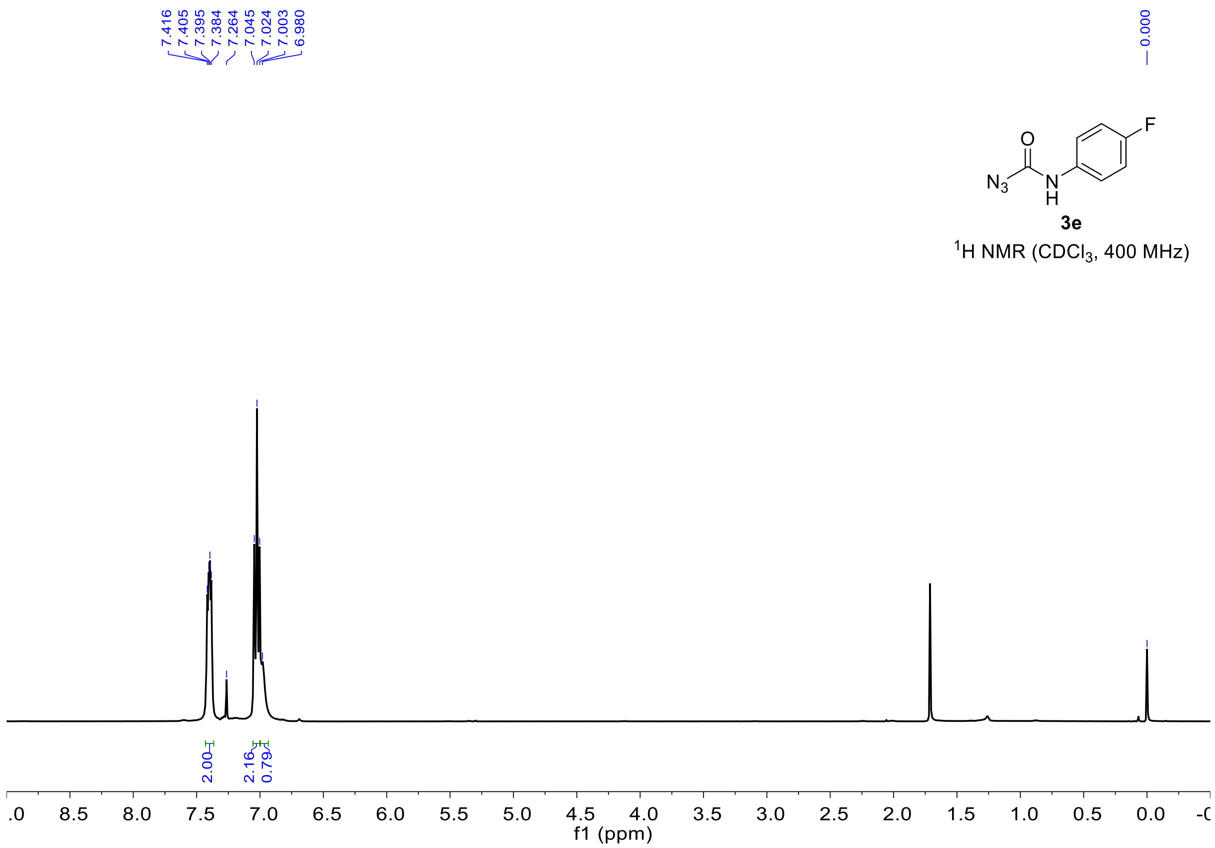
7.416
7.405
7.395
7.384
7.264
7.045
7.024
7.003
6.980
0.000
3e
1H NMR (CDCl3, 400 MHz)
2.00
2.16
0.79
f1 (ppm)

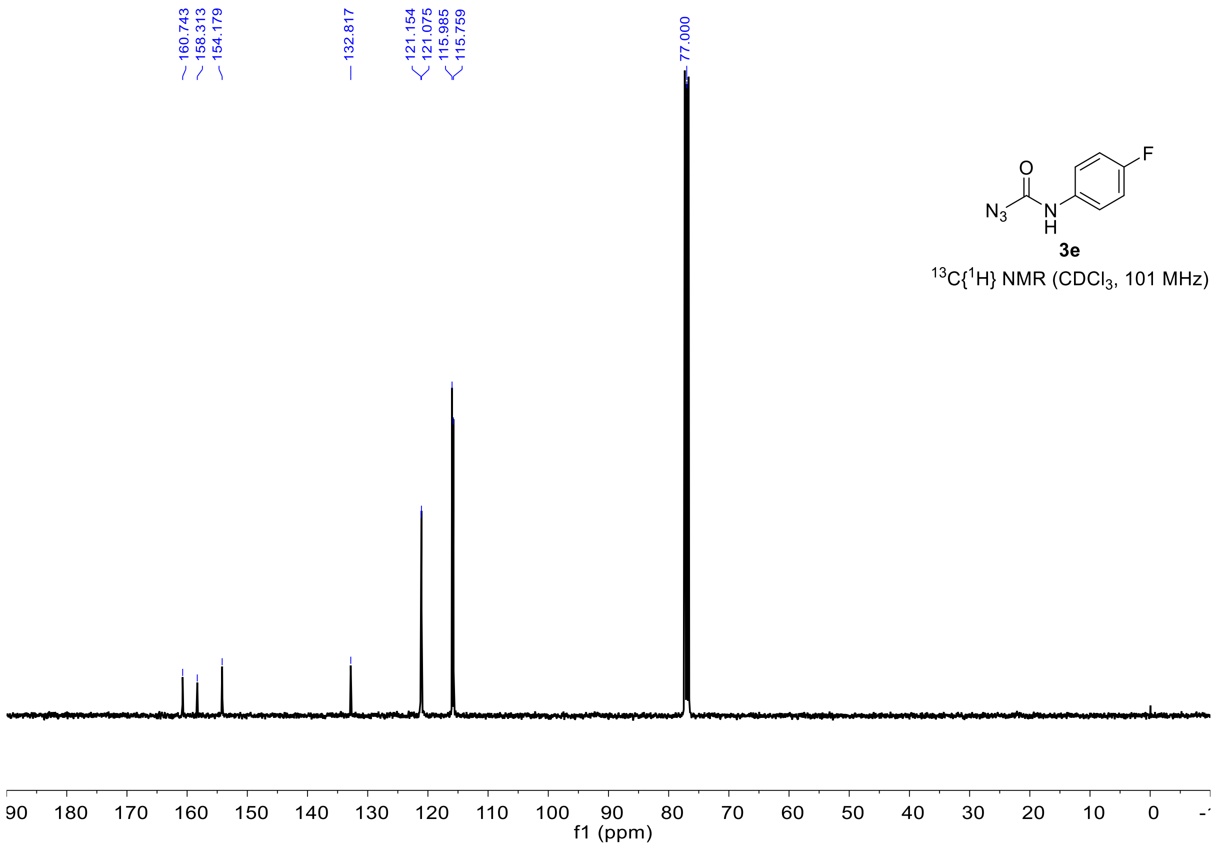
160.743
158.313
154.179
132.817
121.154
121.075
115.985
115.759
77.000
3e
13C{1H} NMR (CDCl3, 101 MHz)
f1 (ppm)

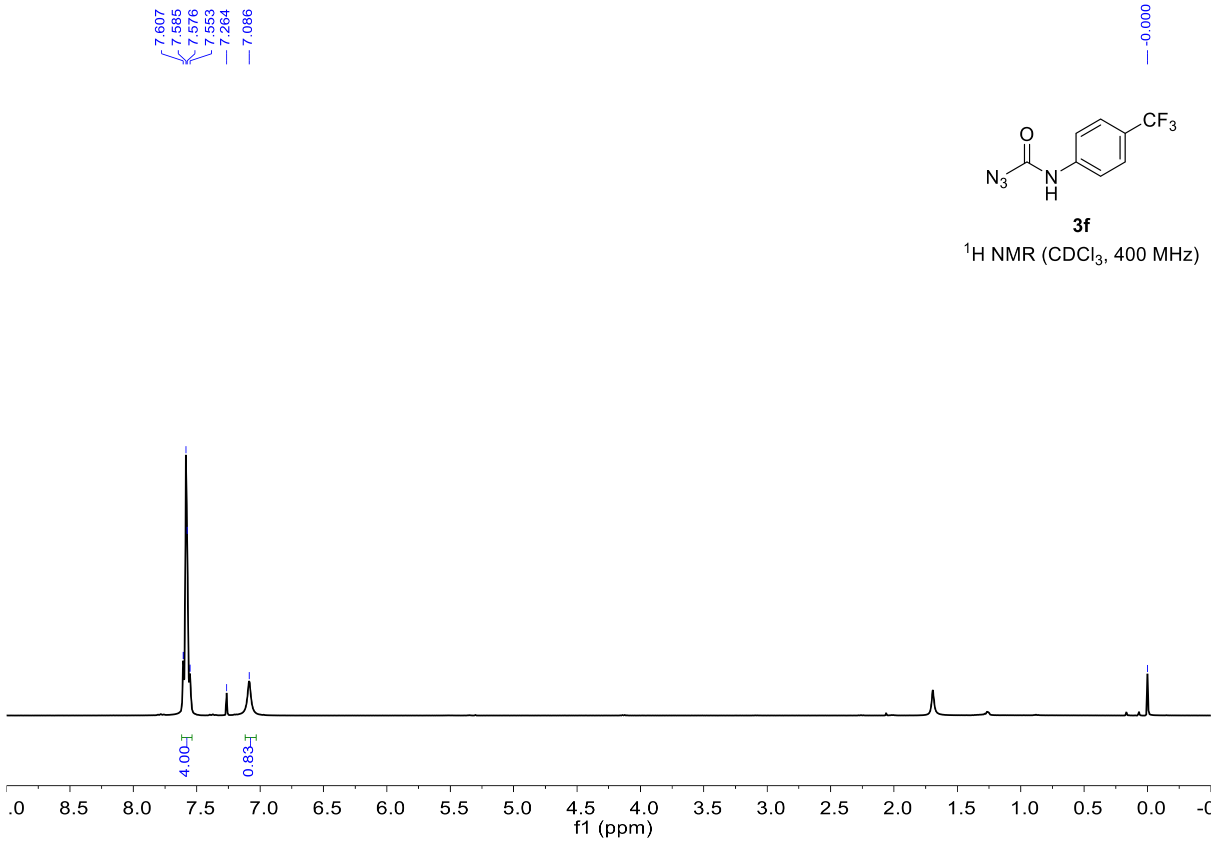
7.607
7.585
7.576
7.553
7.264
7.086
-0.000
3f
^{1}H NMR ($CDCl_3$, 400 MHz)
4.00
0.83
f1 (ppm)

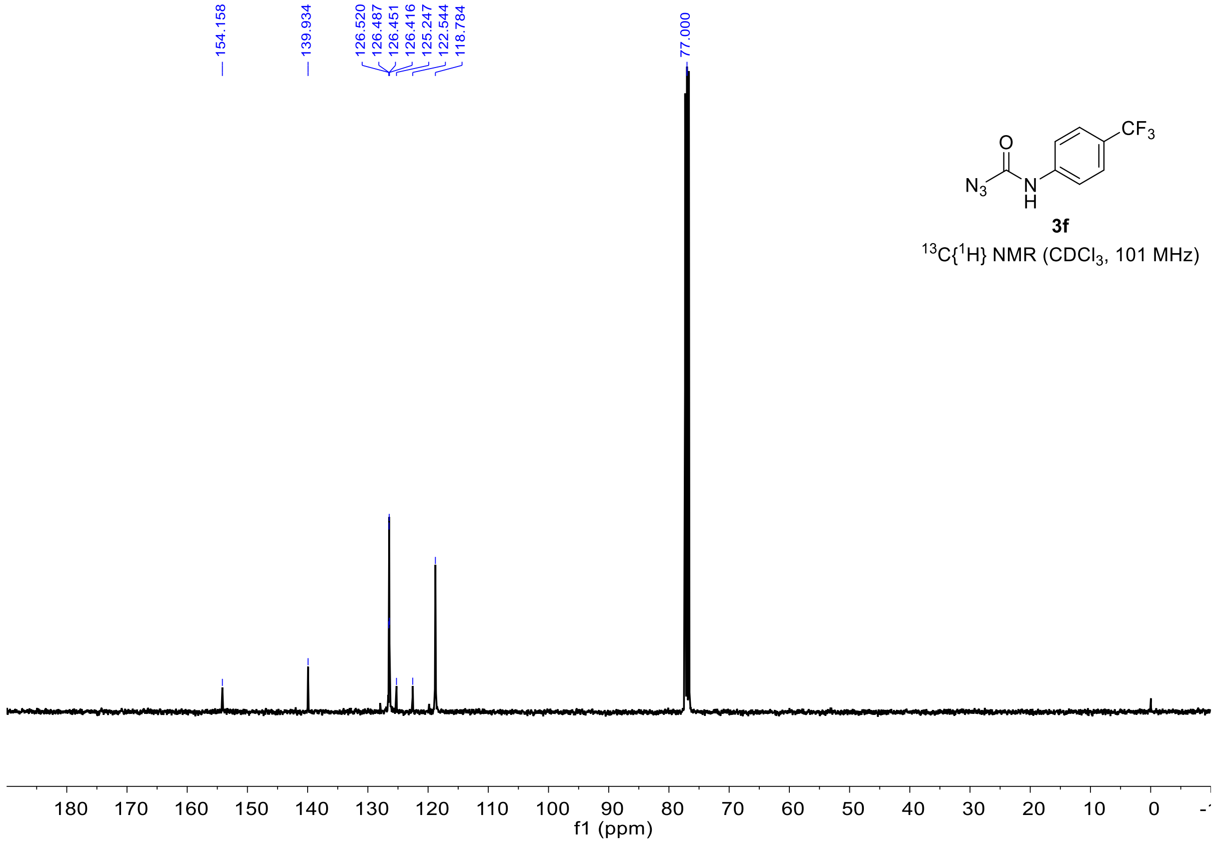
154.158
139.934
126.520
126.487
126.451
126.416
125.247
122.544
118.784
77.000
3f
$^{13}C\{^1H\}$ NMR ($CDCl_3$, 101 MHz)
f1 (ppm)

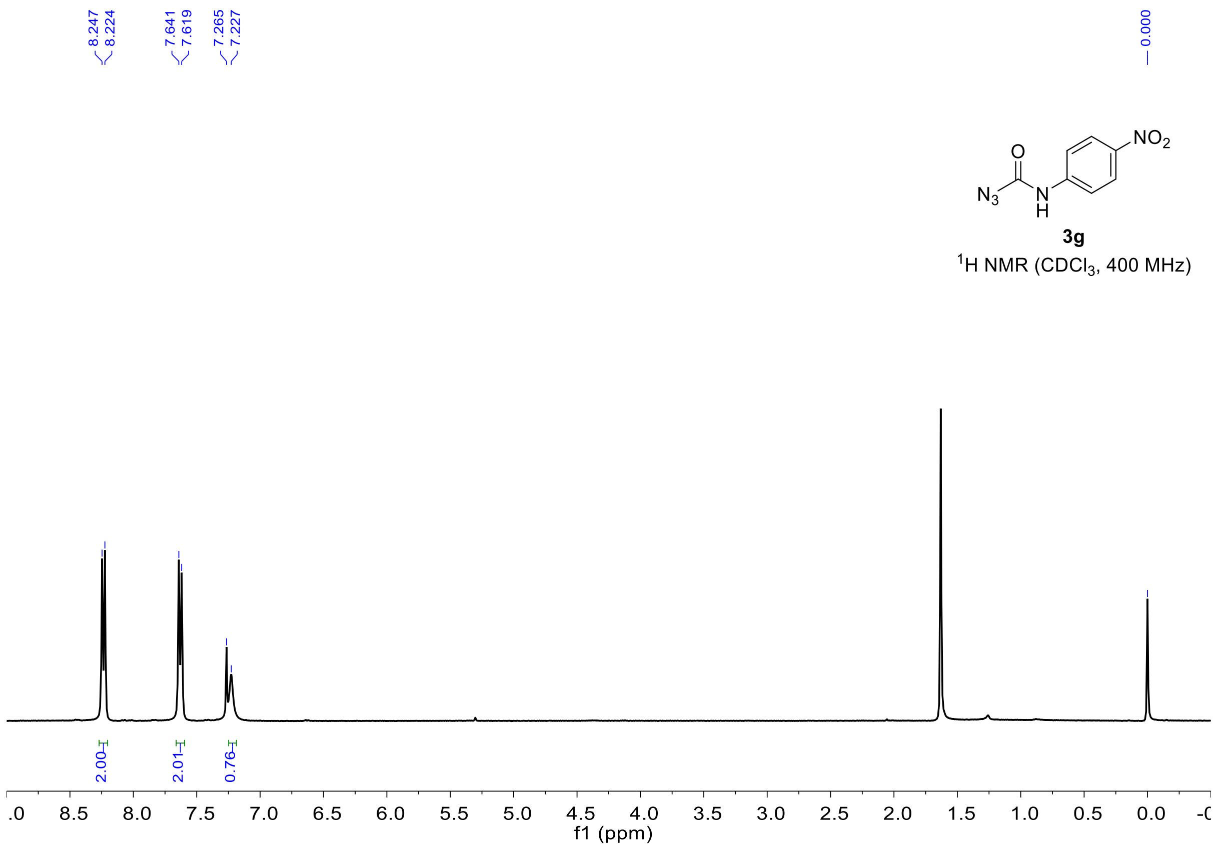
8.247
8.224
7.641
7.619
7.265
7.227
0.000
NO2
O
N3
N
H
3g
1H NMR (CDCl3, 400 MHz)
2.00
2.01
0.76
f1 (ppm)

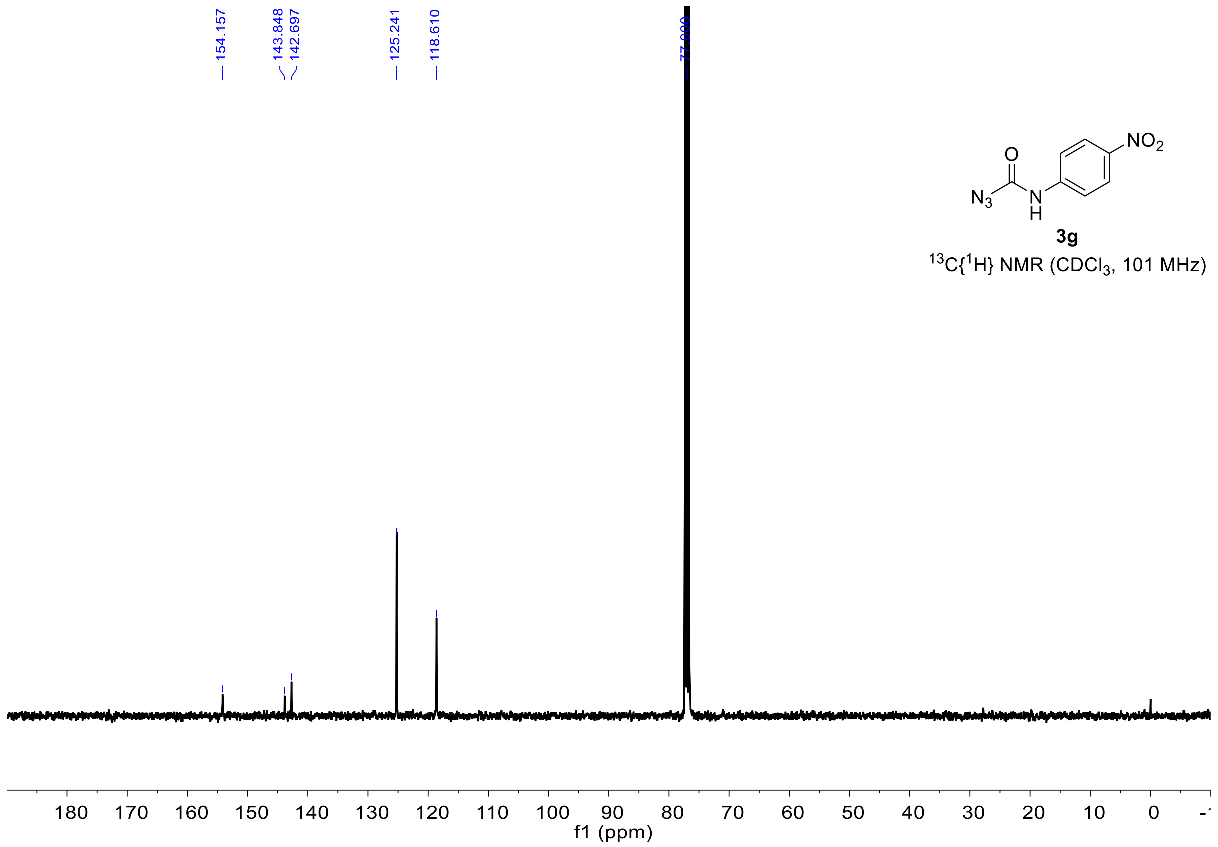
154.157
143.848
142.697
125.241
118.610
NO2
O
N3
N
H
3g
13C{1H} NMR (CDCl3, 101 MHz)
f1 (ppm)

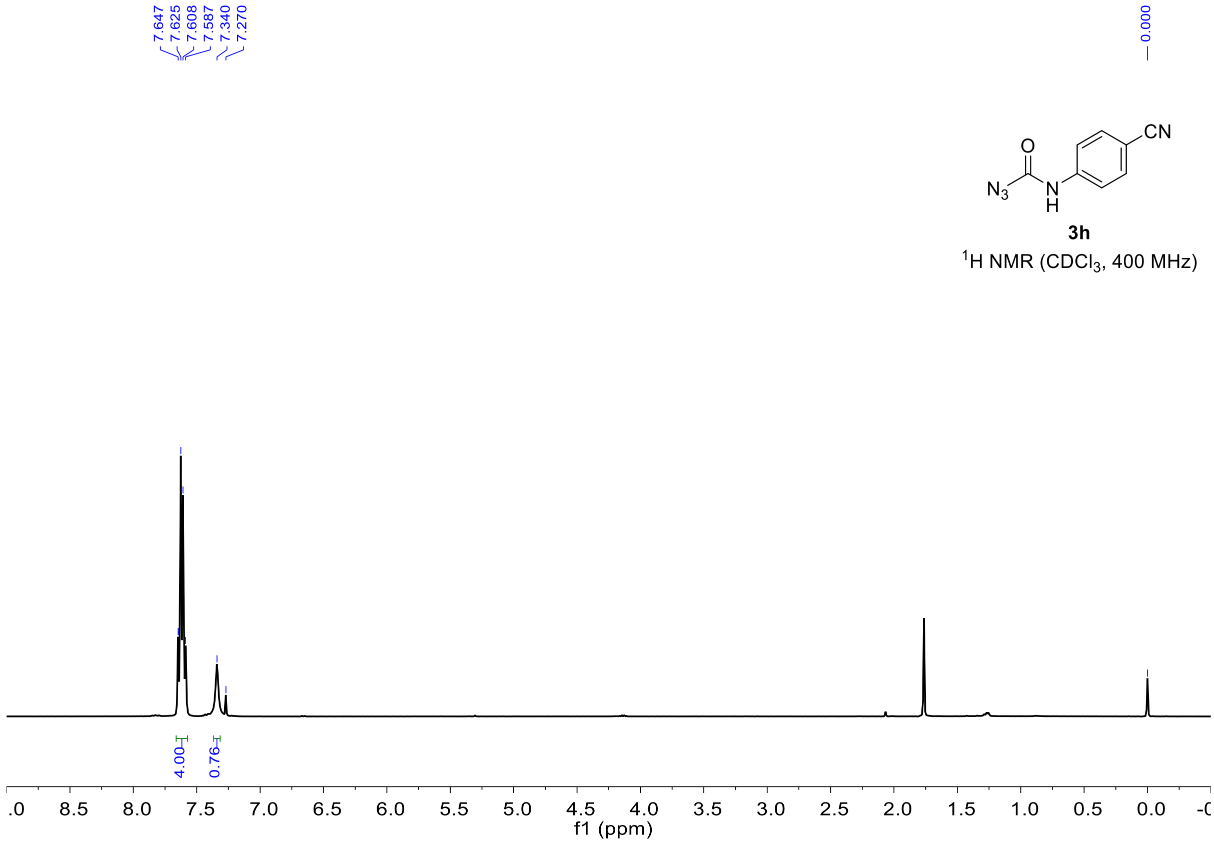
7.647
7.625
7.608
7.587
7.340
7.270
0.000
CN
O
N3
N
H
3h
1H NMR (CDCl3, 400 MHz)
4.00
0.76
f1 (ppm)

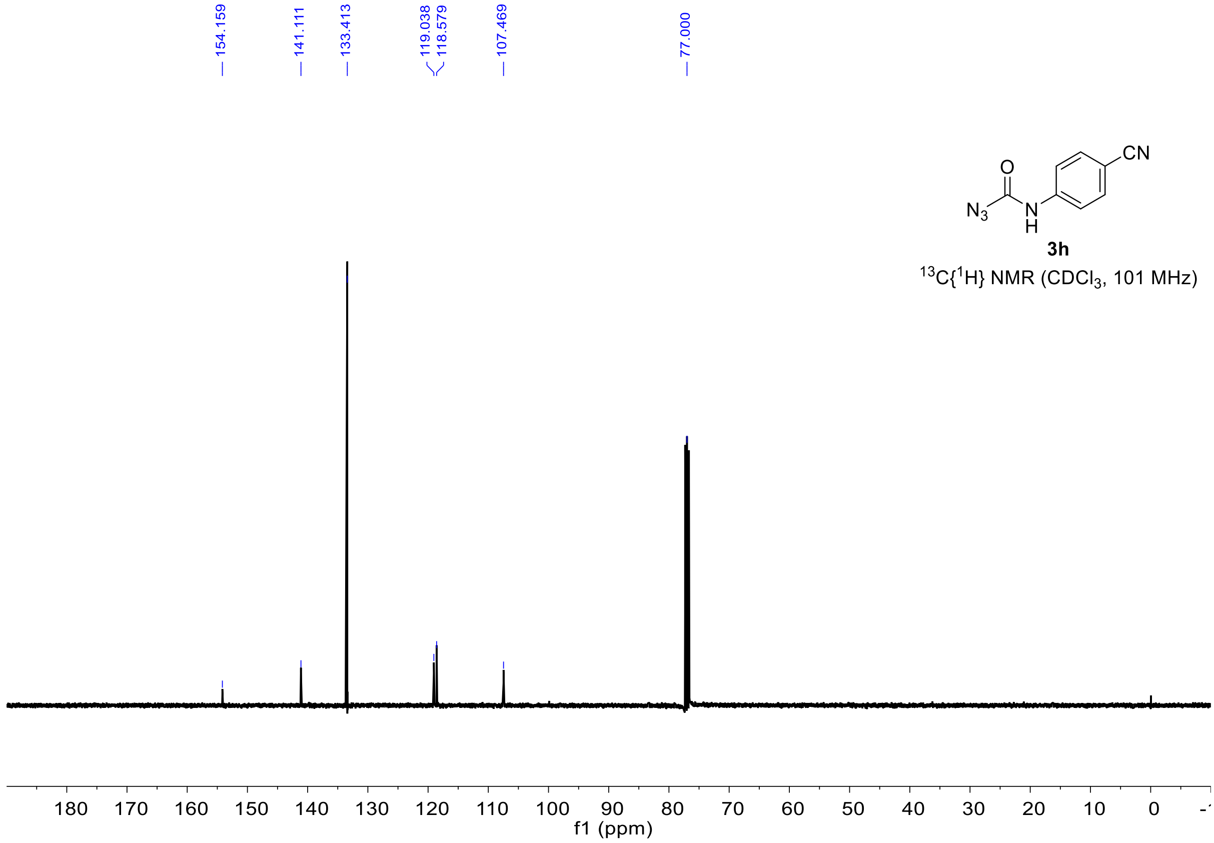
154.159
141.111
133.413
119.038
118.579
107.469
77.000
CN
O
N3
N
H
3h
13C{1H} NMR (CDCl3, 101 MHz)
f1 (ppm)

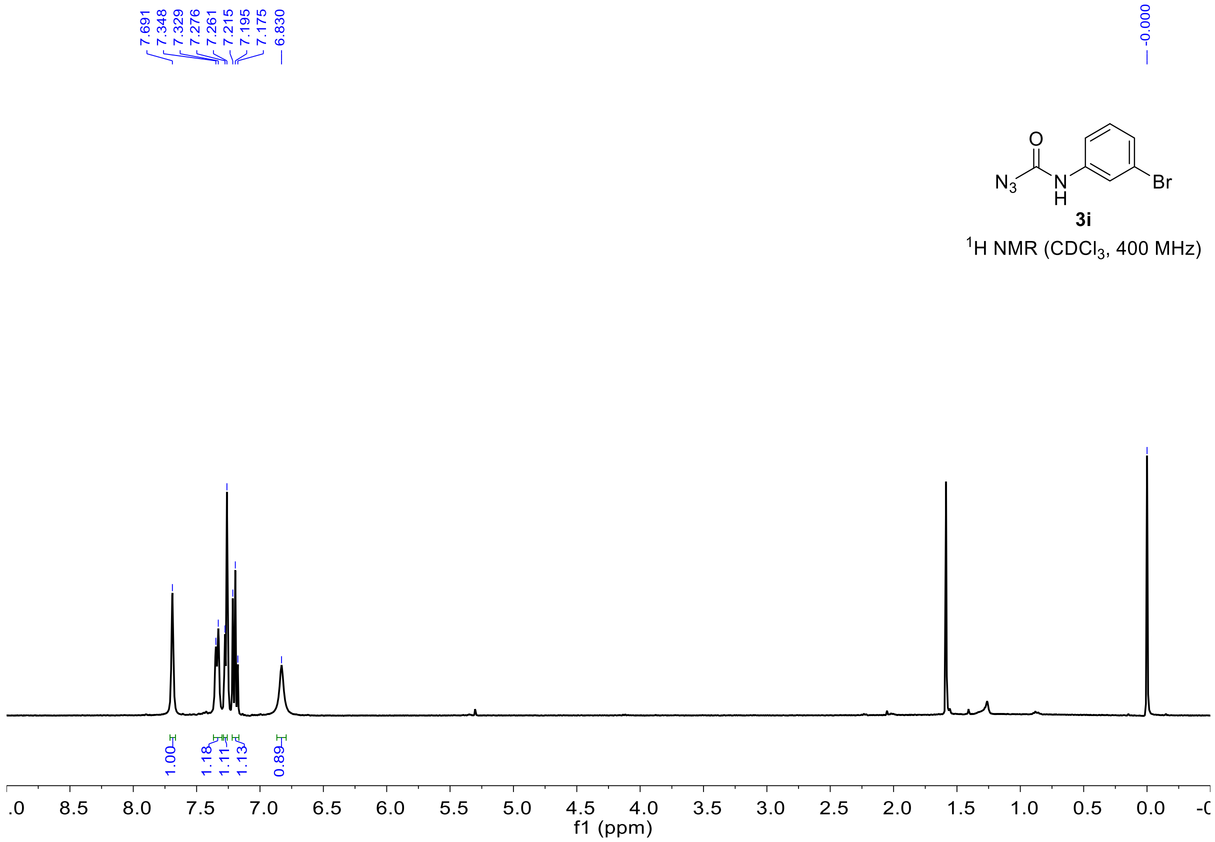
7.691
7.348
7.329
7.276
7.261
7.215
7.195
7.175
6.830
-0.000
3i
^{1}H NMR (CDCl$_3$, 400 MHz)
1.00
1.18
1.11
1.13
0.89
f1 (ppm)

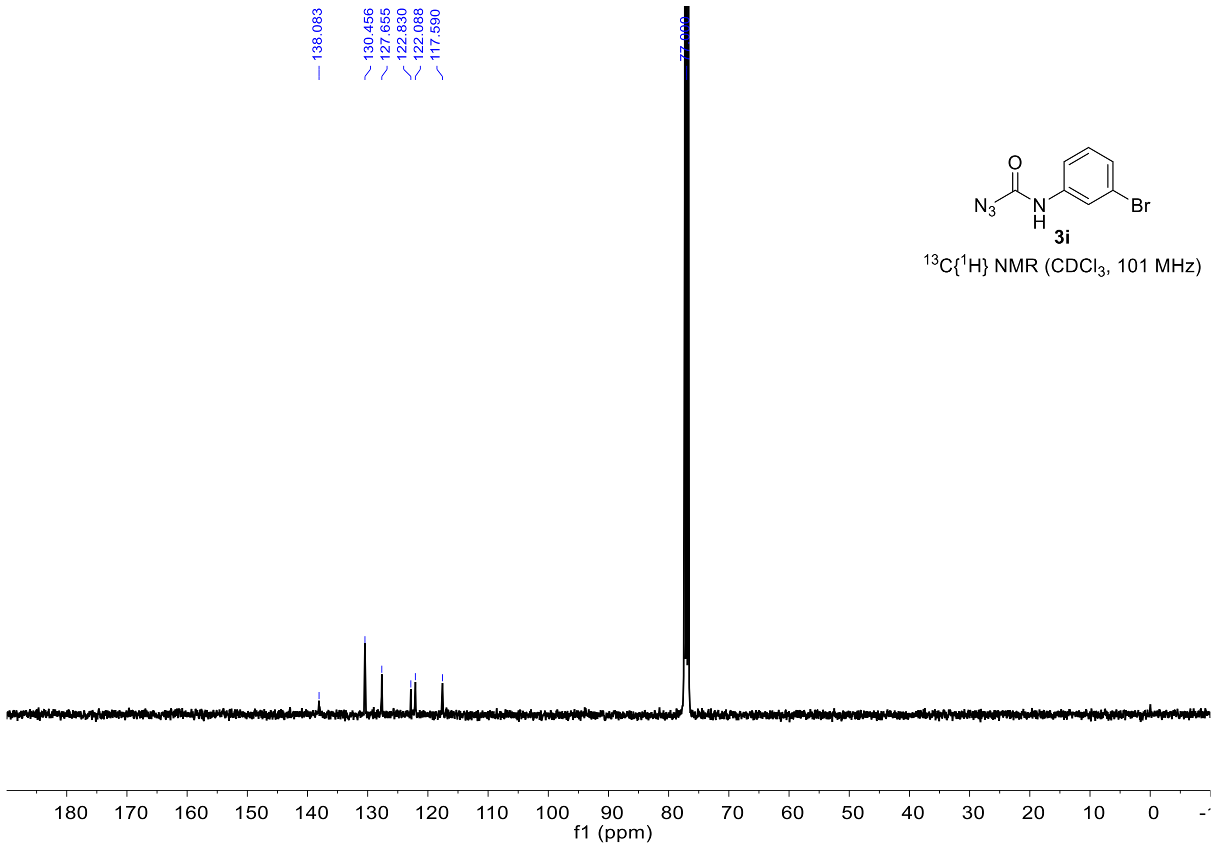
138.083
130.456
127.655
122.830
122.088
117.590
3i
^{13}C{^{1}H} NMR (CDCl$_3$, 101 MHz)
f1 (ppm)

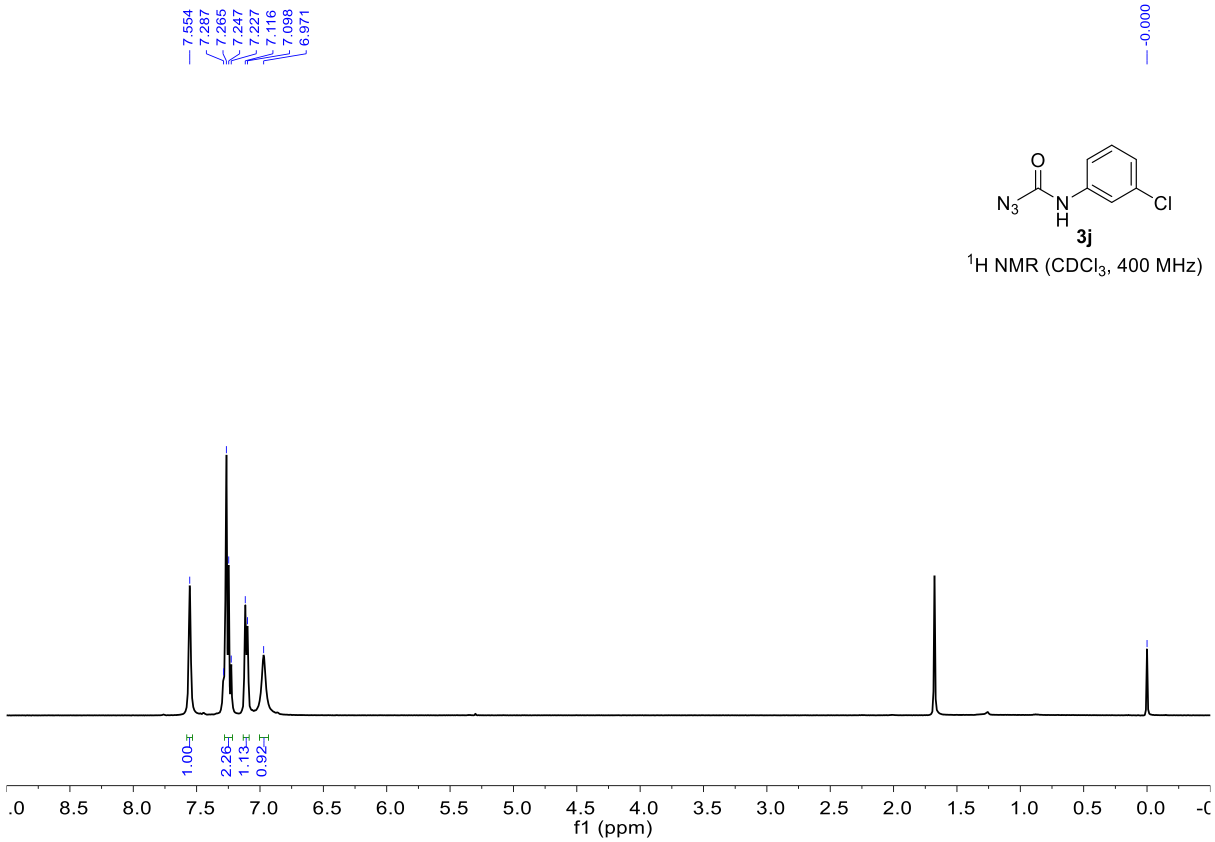
7.554
7.287
7.265
7.247
7.227
7.116
7.098
6.971
0.000
3j
^{1}H NMR (CDCl$_3$, 400 MHz)
1.00
2.26
1.13
0.92
f1 (ppm)

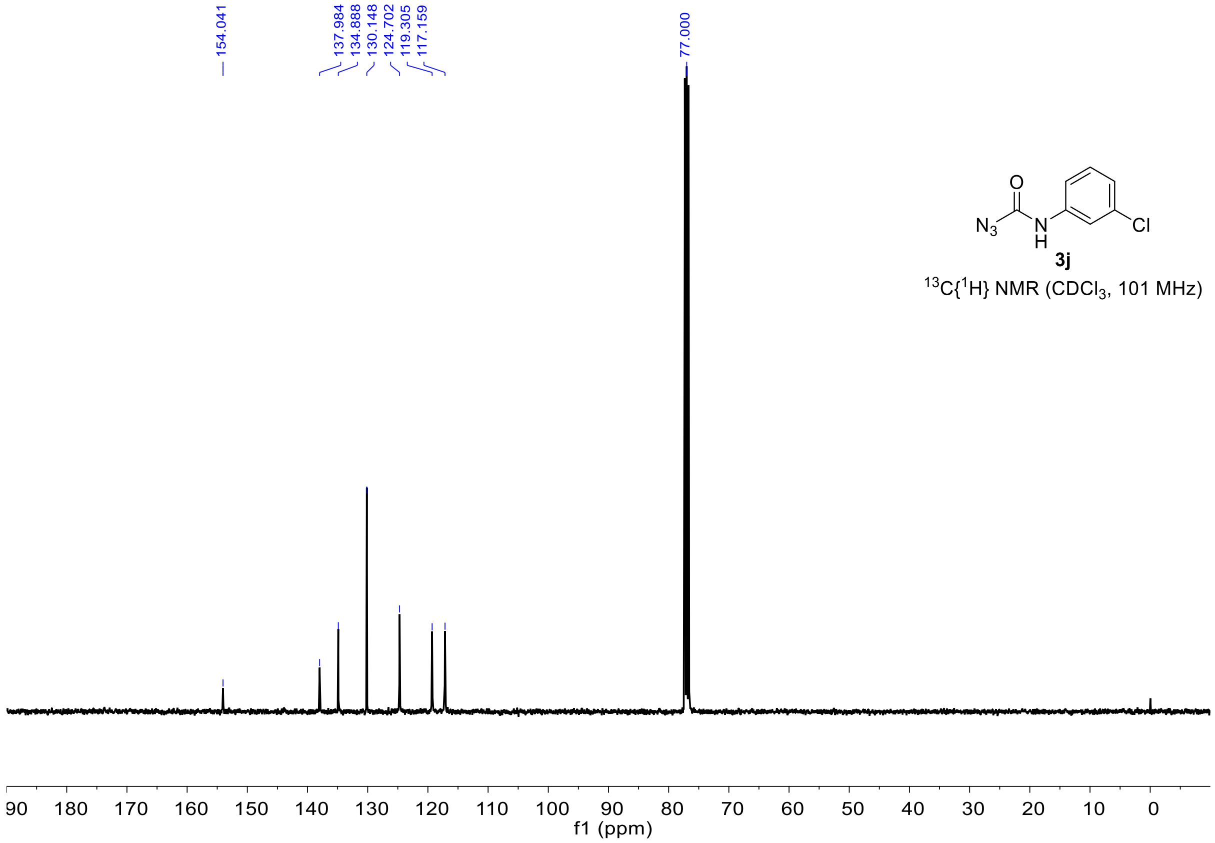
154.041
137.984
134.888
130.148
124.702
119.305
117.159
77.000
3j
^{13}C{^{1}H} NMR (CDCl$_3$, 101 MHz)
f1 (ppm)

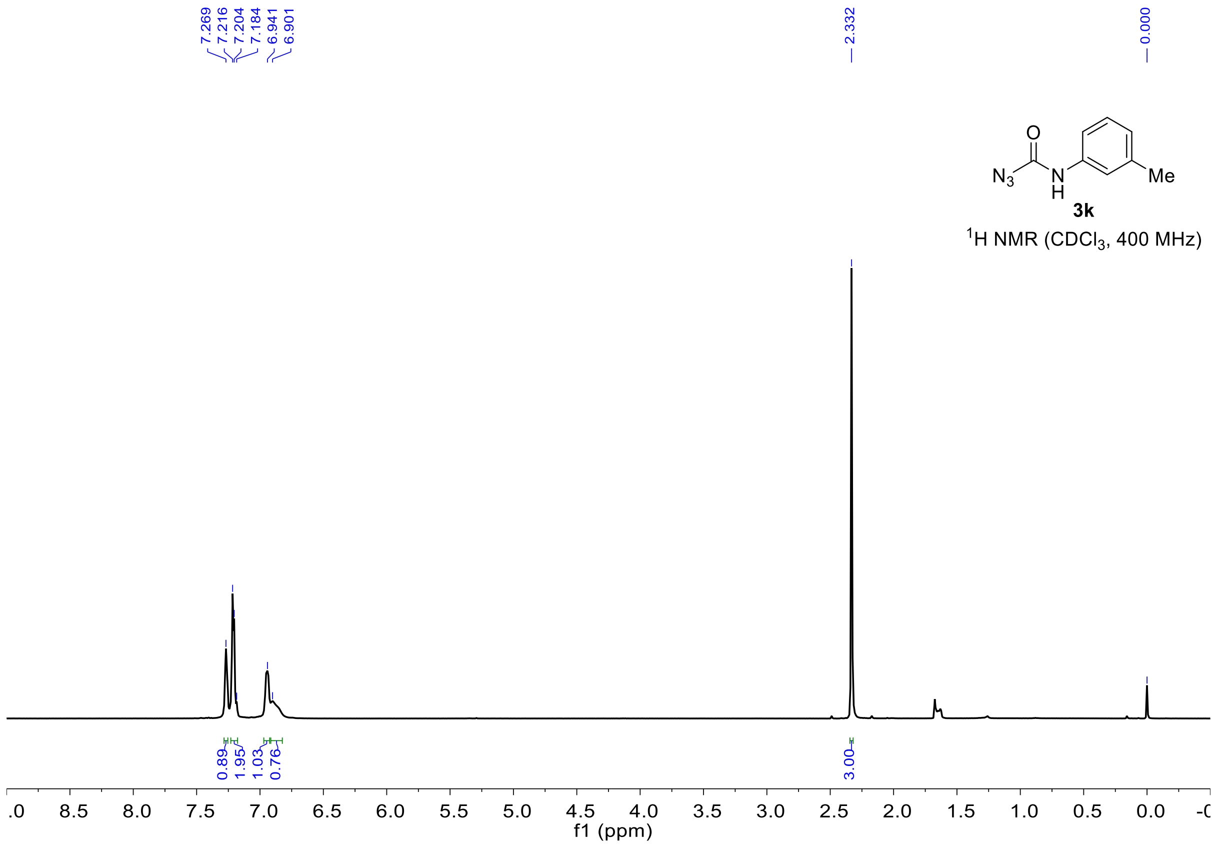
7.269
7.216
7.204
7.184
6.941
6.901
2.332
0.000
N3
O
N
H
Me
3k
1H NMR (CDCl3, 400 MHz)
0.89
1.95
1.03
0.76
3.00
f1 (ppm)

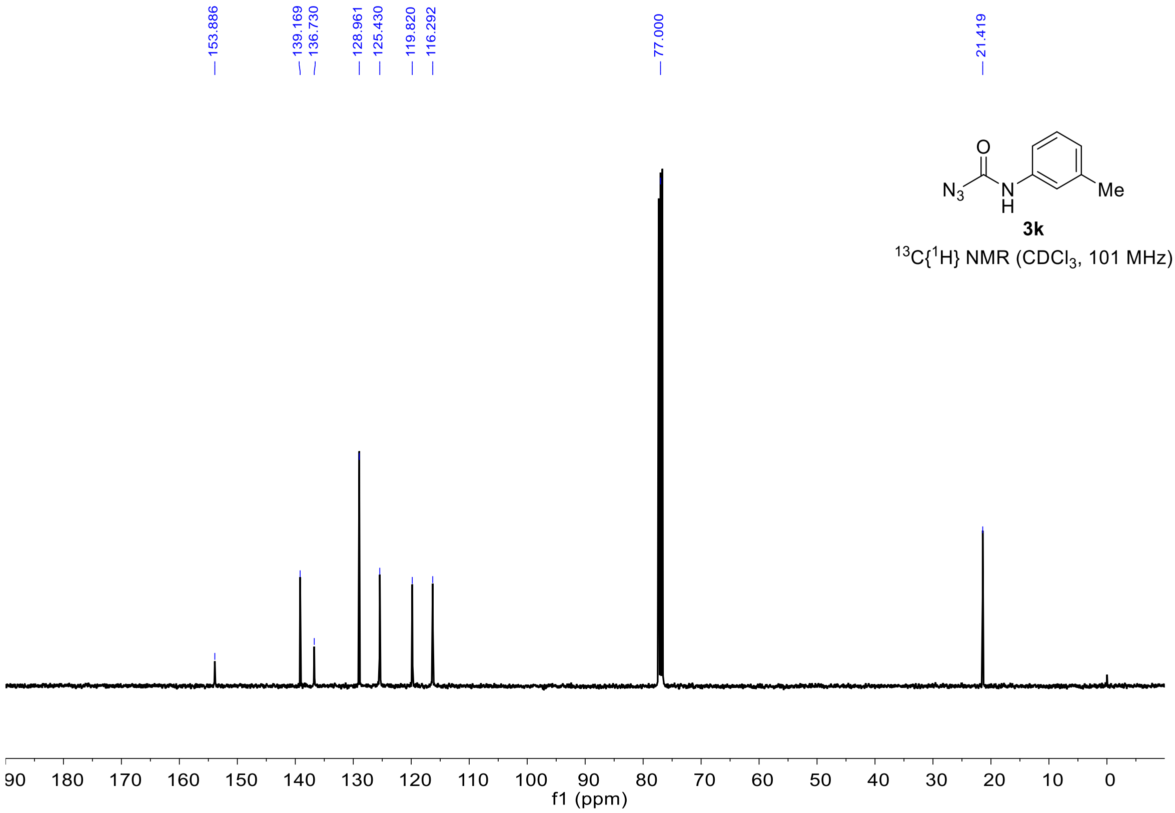
153.886
139.169
136.730
128.961
125.430
119.820
116.292
77.000
21.419
N3
O
N
H
Me
3k
13C{1H} NMR (CDCl3, 101 MHz)
f1 (ppm)

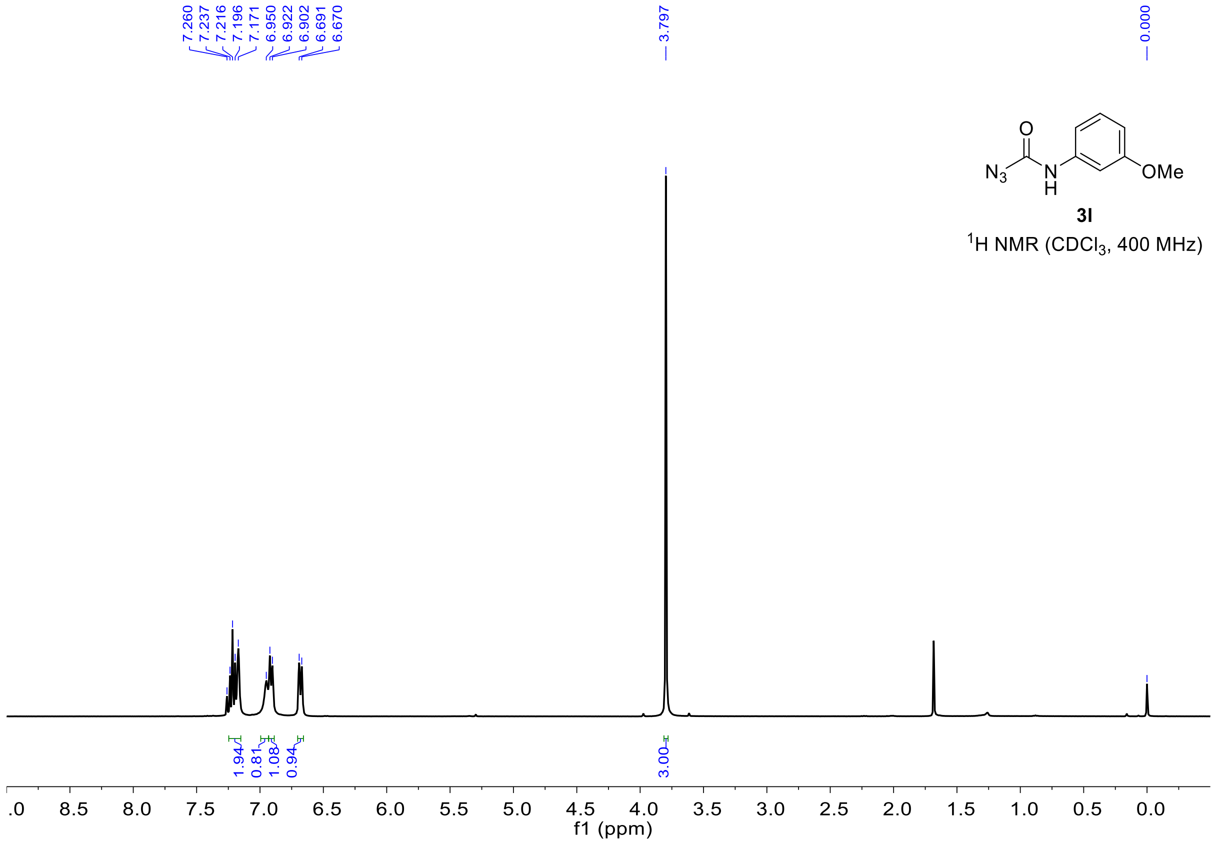

3l

^{1}H NMR ($CDCl_3$, 400 MHz)

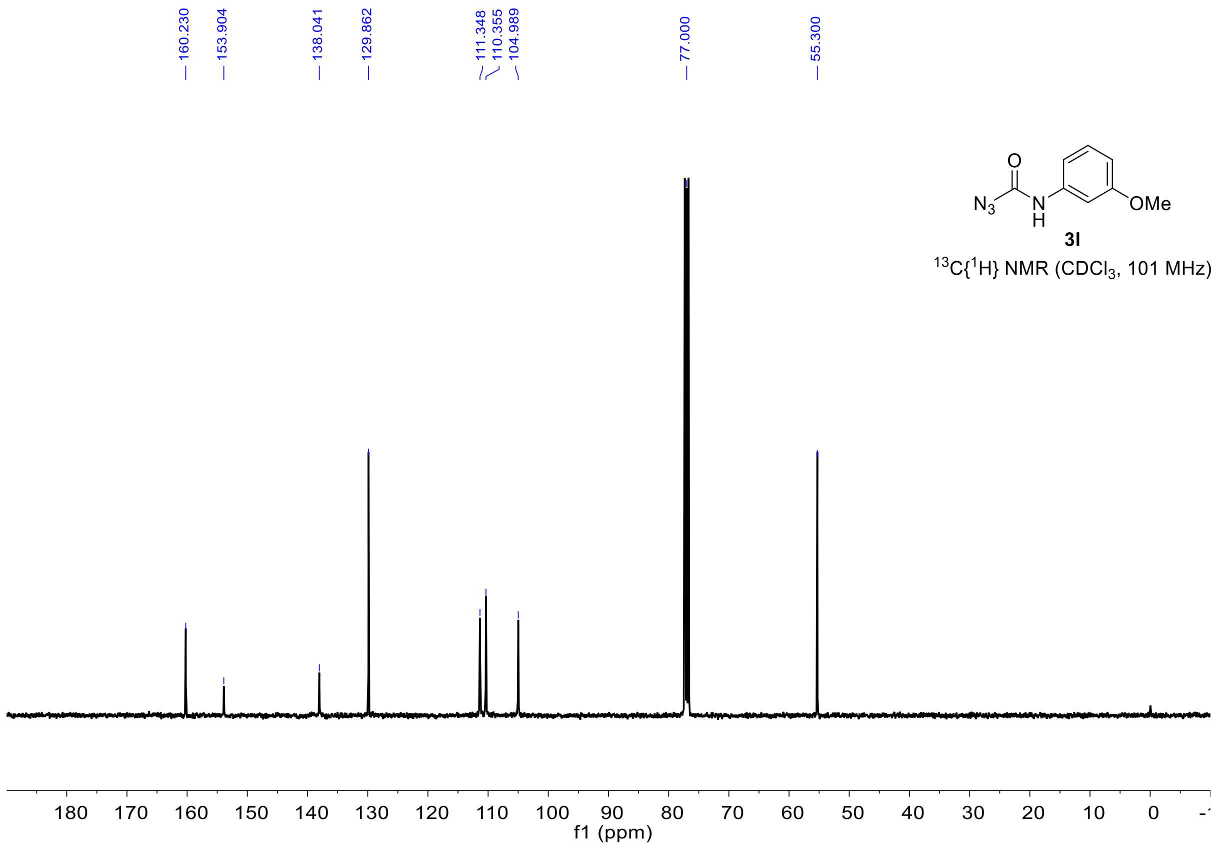

3l

$^{13}C\{^1H\}$ NMR ($CDCl_3$, 101 MHz)

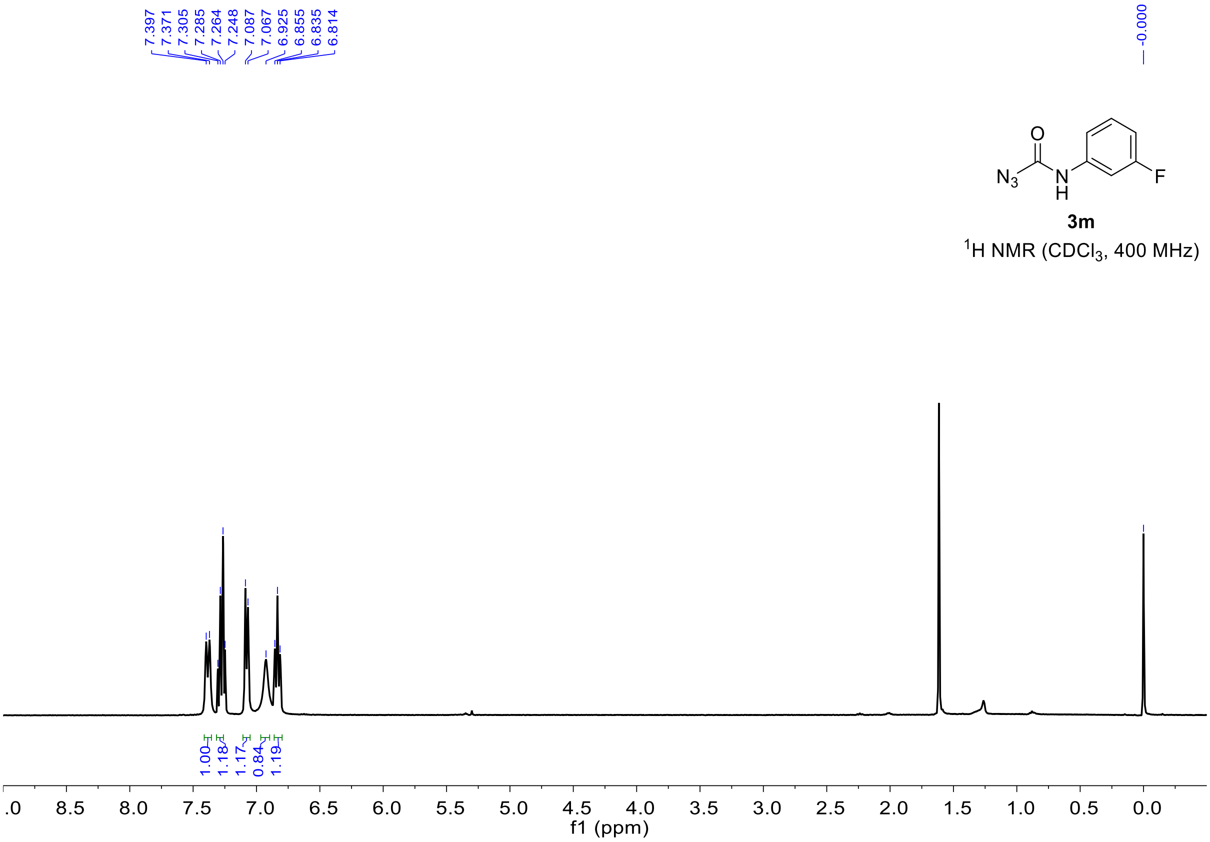

3m

^{1}H NMR ($CDCl_3$, 400 MHz)

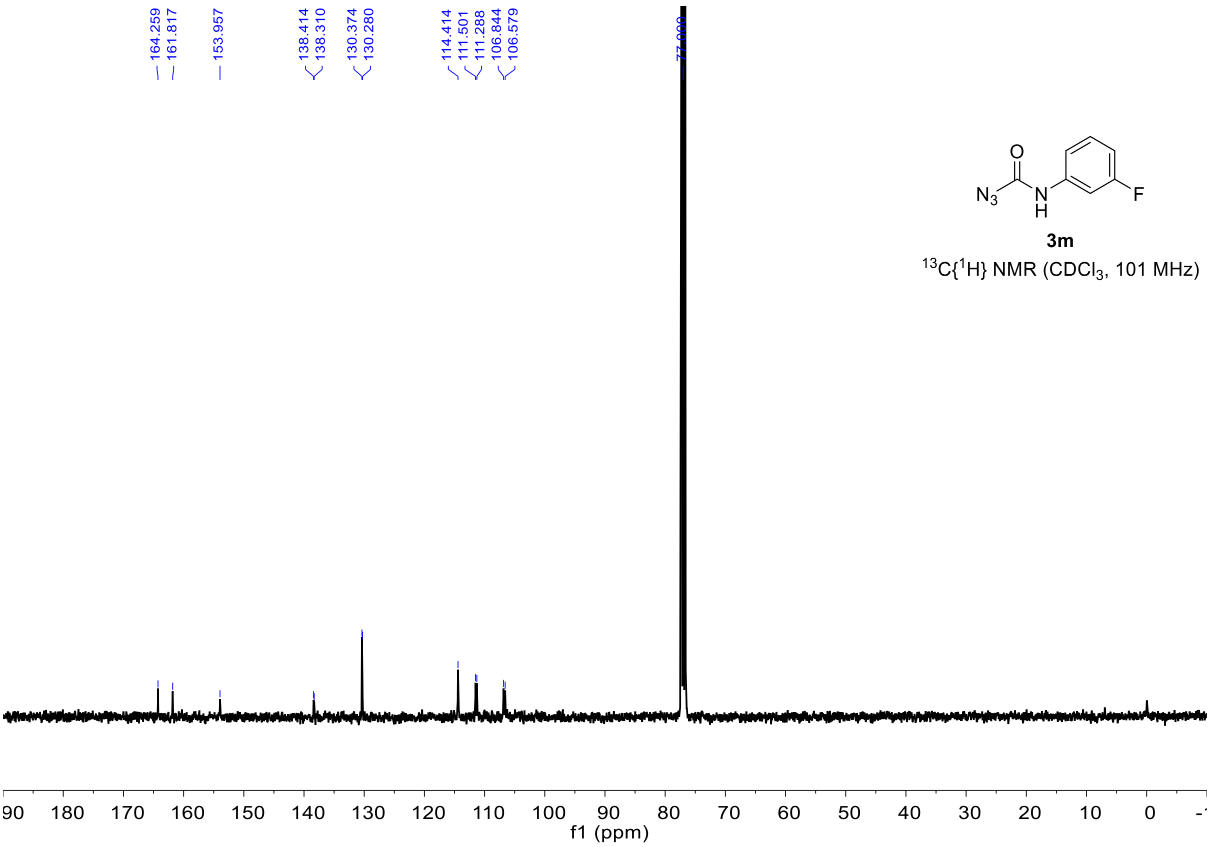

3m

^{13}C{^{1}H} NMR ($CDCl_3$, 101 MHz)

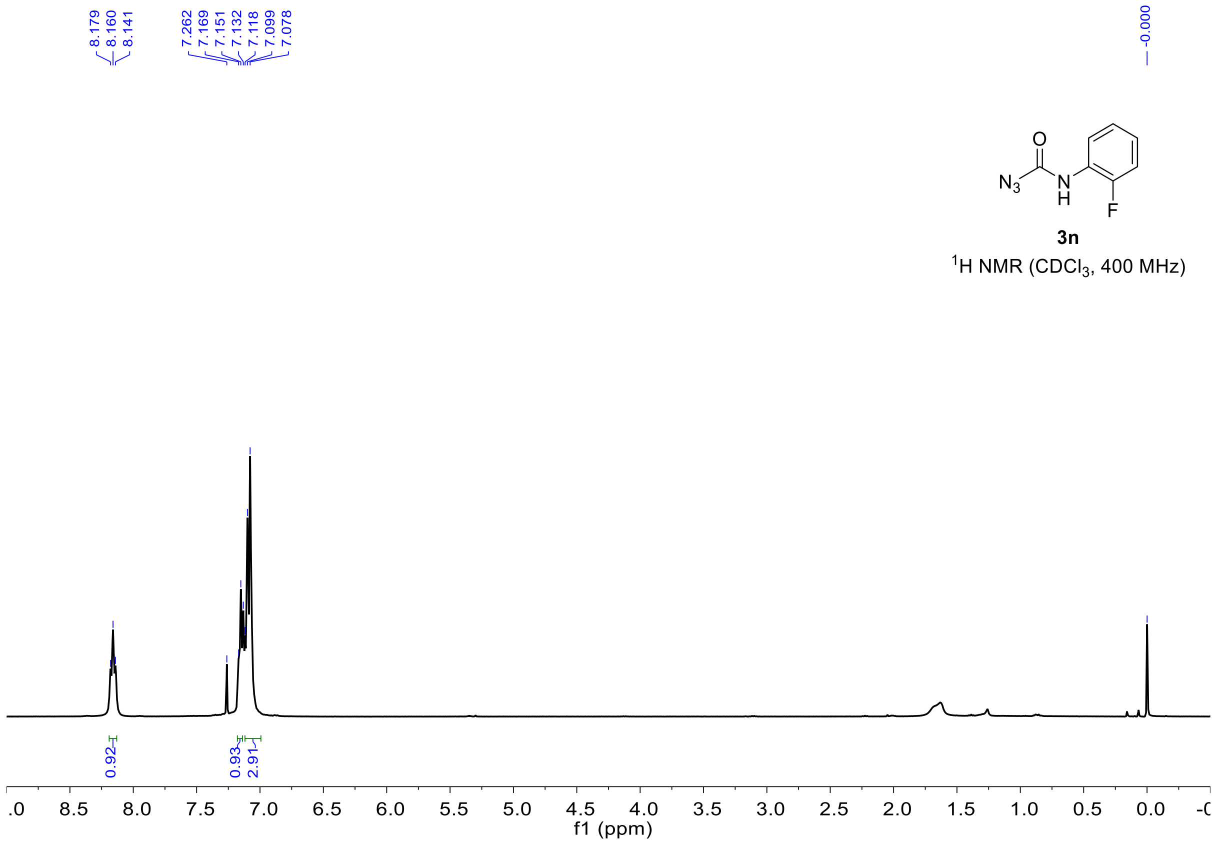
8.179
8.160
8.141
7.262
7.169
7.151
7.132
7.118
7.099
7.078
0.000
N3
O
N
H
F
3n
1H NMR (CDCl3, 400 MHz)
0.92
0.93
2.91
8.5 8.0 7.5 7.0 6.5 6.0 5.5 5.0 4.5 4.0 3.5 3.0 2.5 2.0 1.5 1.0 0.5 0.0
f1 (ppm)

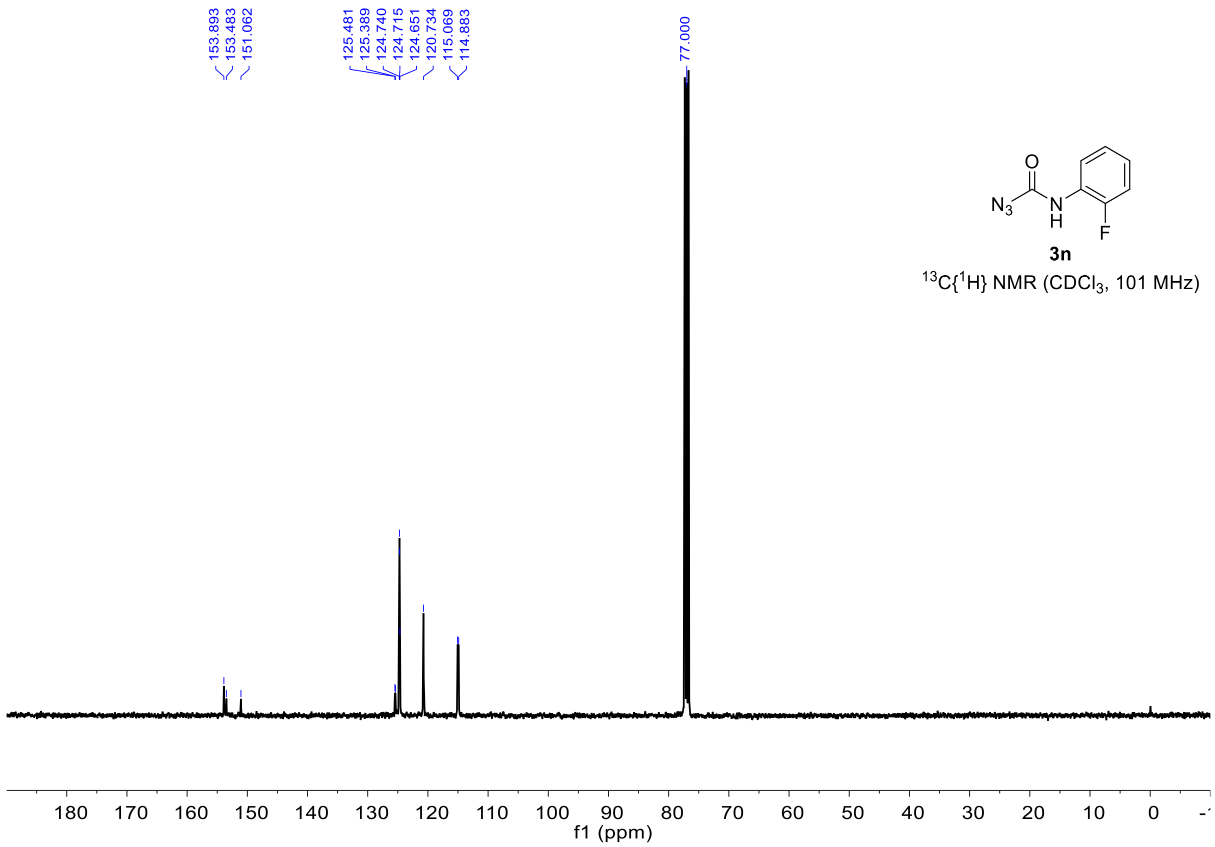
153.893
153.483
151.062
125.481
125.389
124.740
124.715
124.651
120.734
115.069
114.883
77.000
N3
O
N
H
F
3n
13C{1H} NMR (CDCl3, 101 MHz)
180 170 160 150 140 130 120 110 100 90 80 70 60 50 40 30 20 10 0
f1 (ppm)

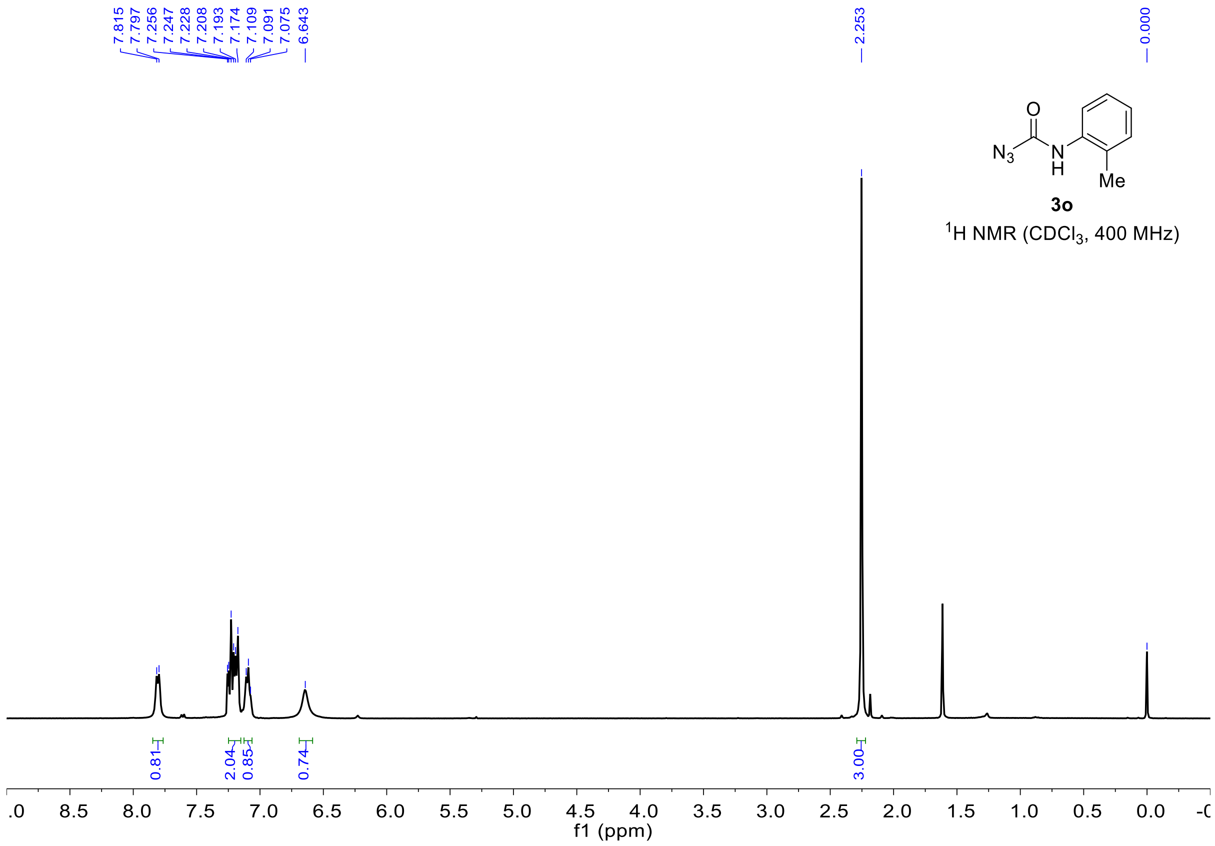
7.815
7.797
7.256
7.247
7.228
7.208
7.193
7.174
7.109
7.091
7.075
6.643
2.253
0.000
O
N3
N
H
Me
3o
1H NMR (CDCl3, 400 MHz)
0.81
2.04
0.85
0.74
3.00
f1 (ppm)

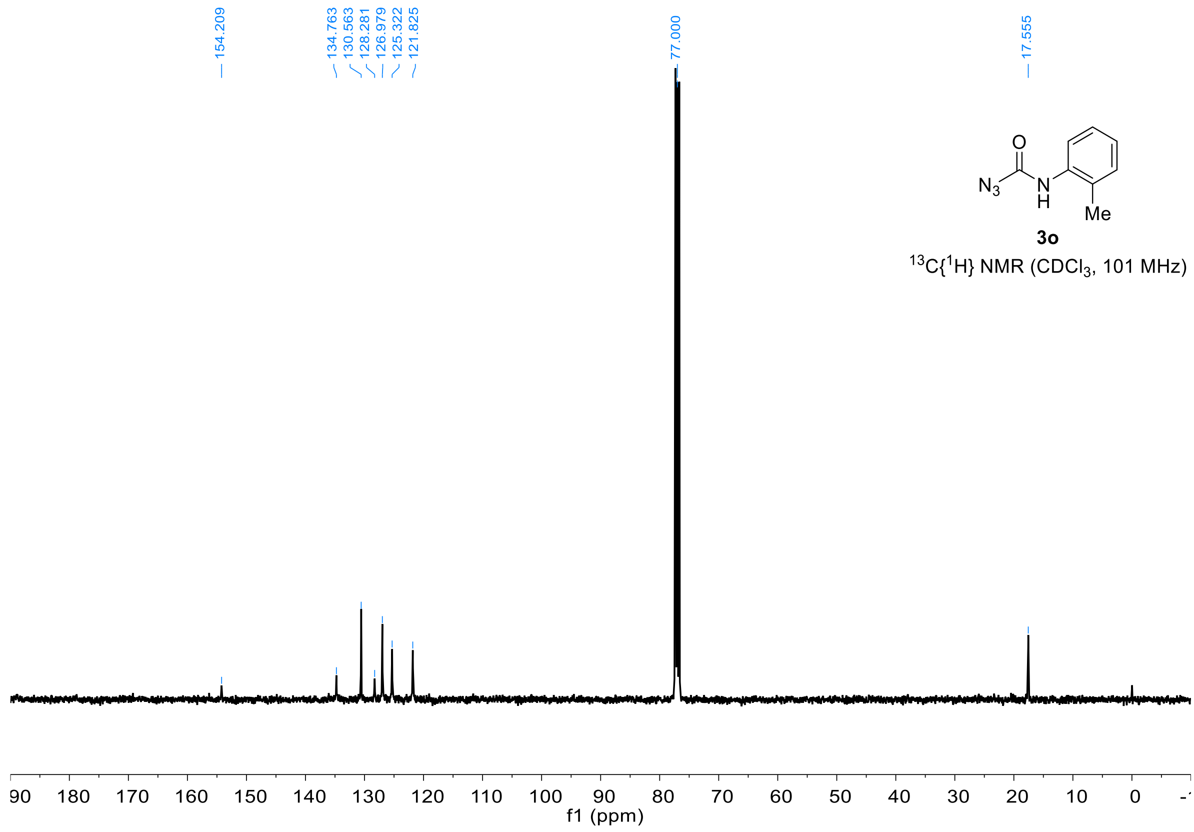
154.209
134.763
130.563
128.281
126.979
125.322
121.825
77.000
17.555
O
N3
N
H
Me
3o
13C{1H} NMR (CDCl3, 101 MHz)
f1 (ppm)

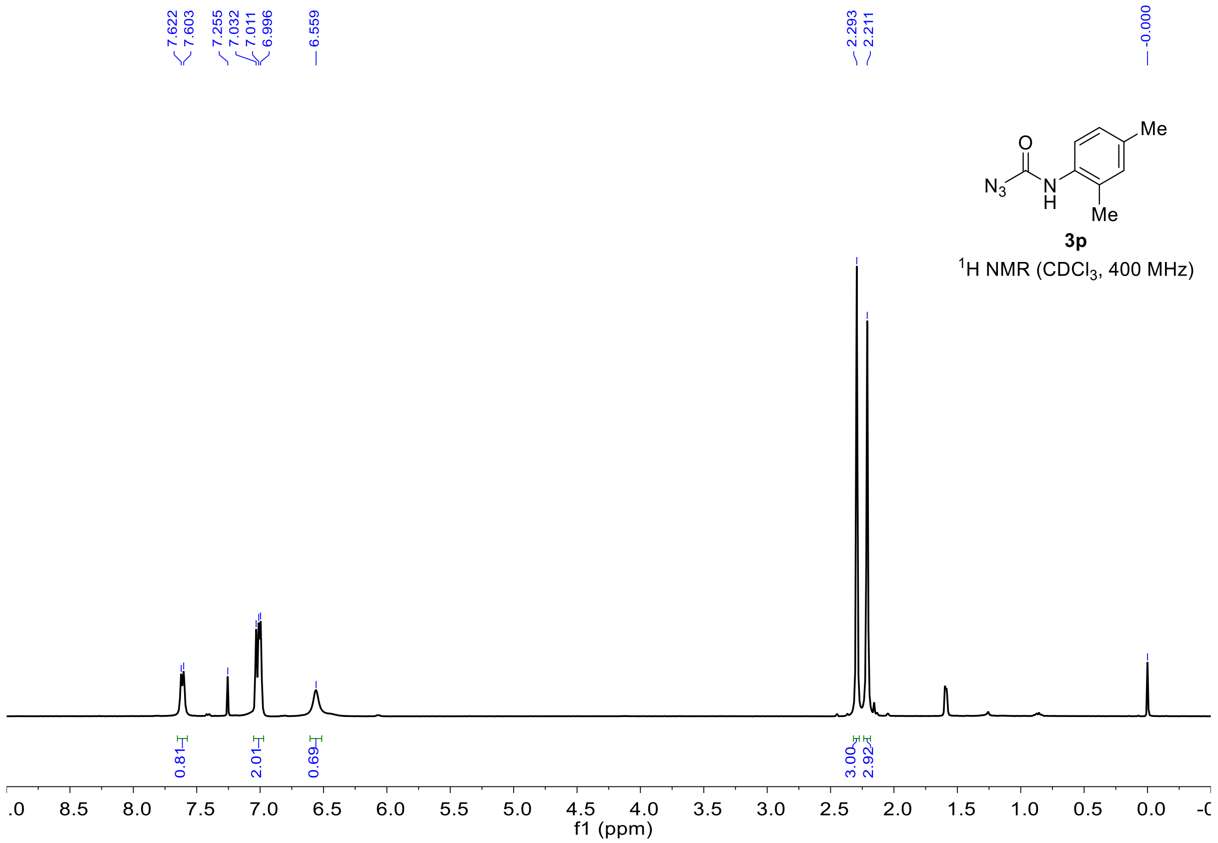

7.622
7.603
7.255
7.032
7.011
6.996
6.559
2.293
2.211
-0.000
O
Me
N3
N
H
Me
3p
1H NMR (CDCl3, 400 MHz)
0.81
2.01
0.69
3.00
2.92
f1 (ppm)

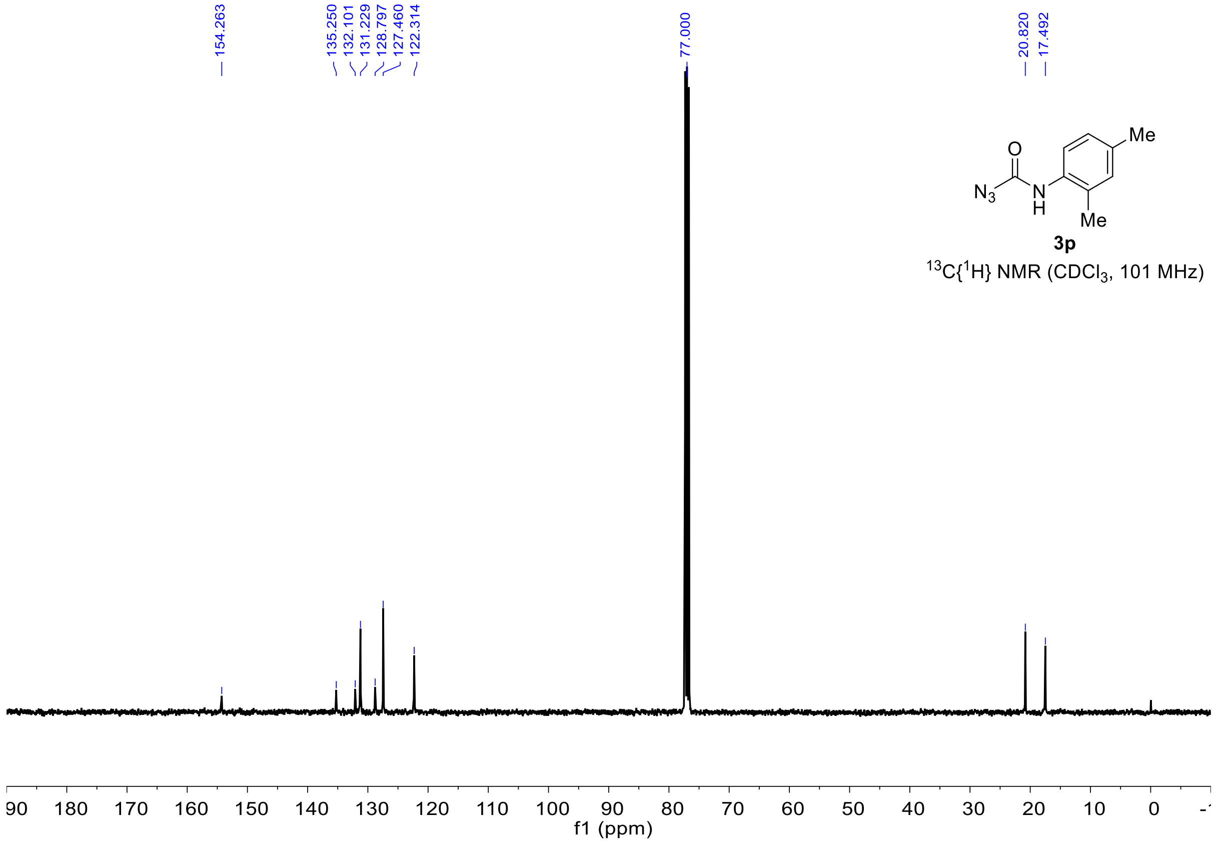

154.263
135.250
132.101
131.229
128.797
127.460
122.314
77.000
20.820
17.492
O
Me
N3
N
H
Me
3p
13C{1H} NMR (CDCl3, 101 MHz)
f1 (ppm)

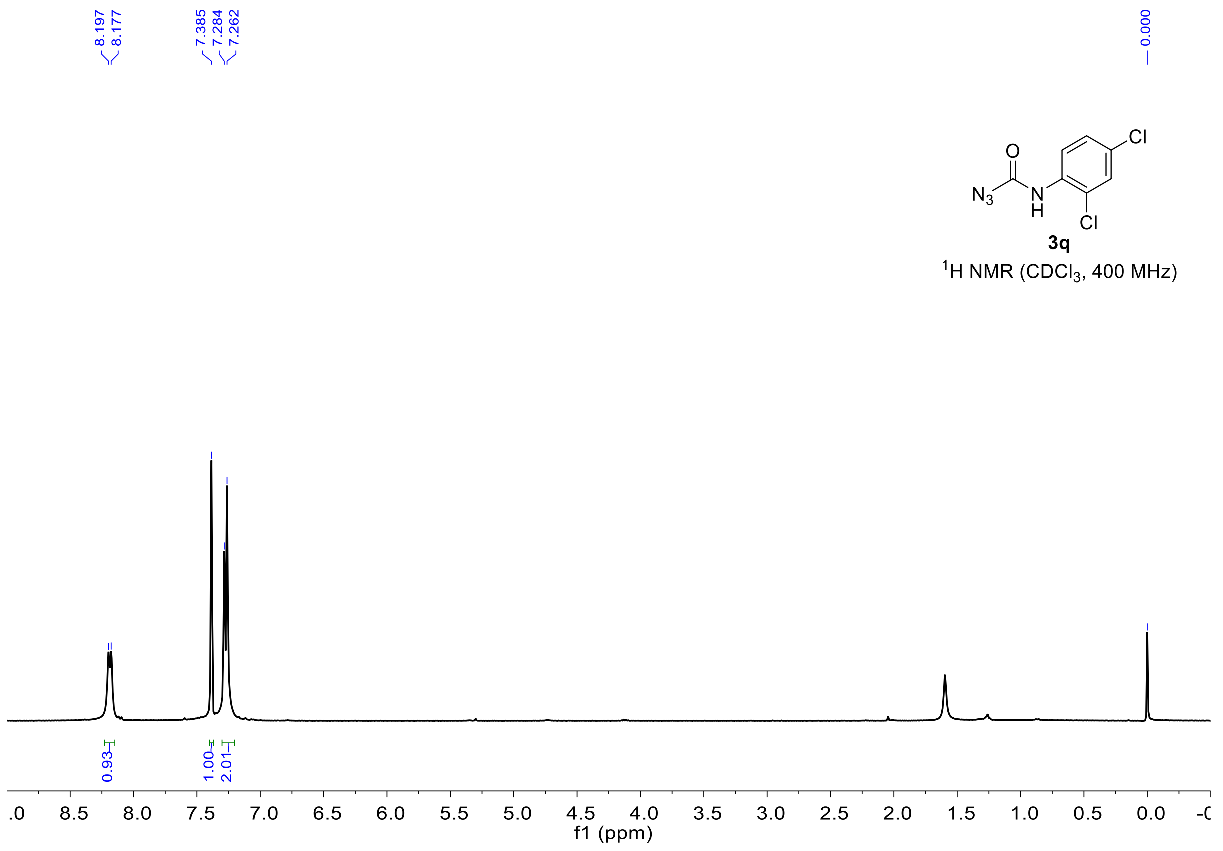
8.197
8.177
7.385
7.284
7.262
0.000
O
N3
N
H
Cl
Cl
3q
1H NMR (CDCl3, 400 MHz)
0.93
1.00
2.01
f1 (ppm)

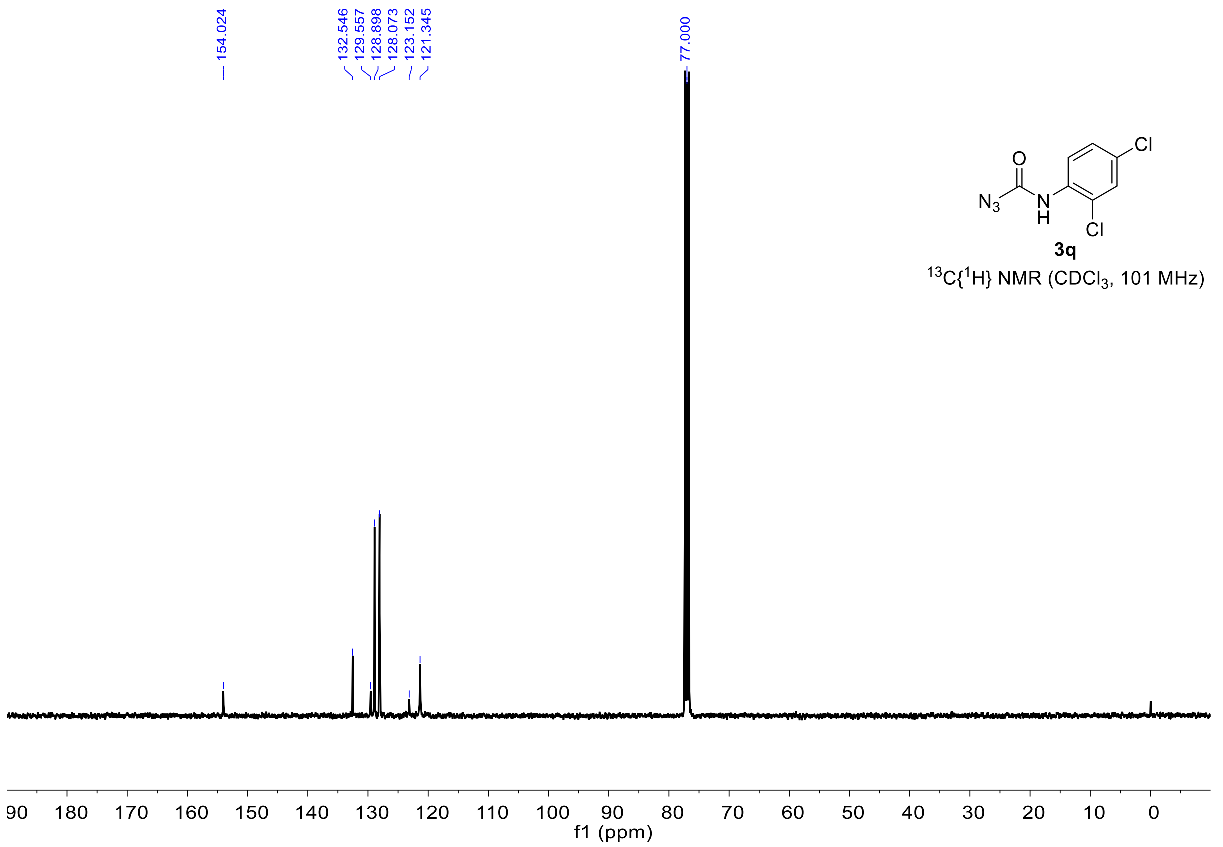
154.024
132.546
129.557
128.898
128.073
123.152
121.345
77.000
O
N3
N
H
Cl
Cl
3q
13C{1H} NMR (CDCl3, 101 MHz)
f1 (ppm)

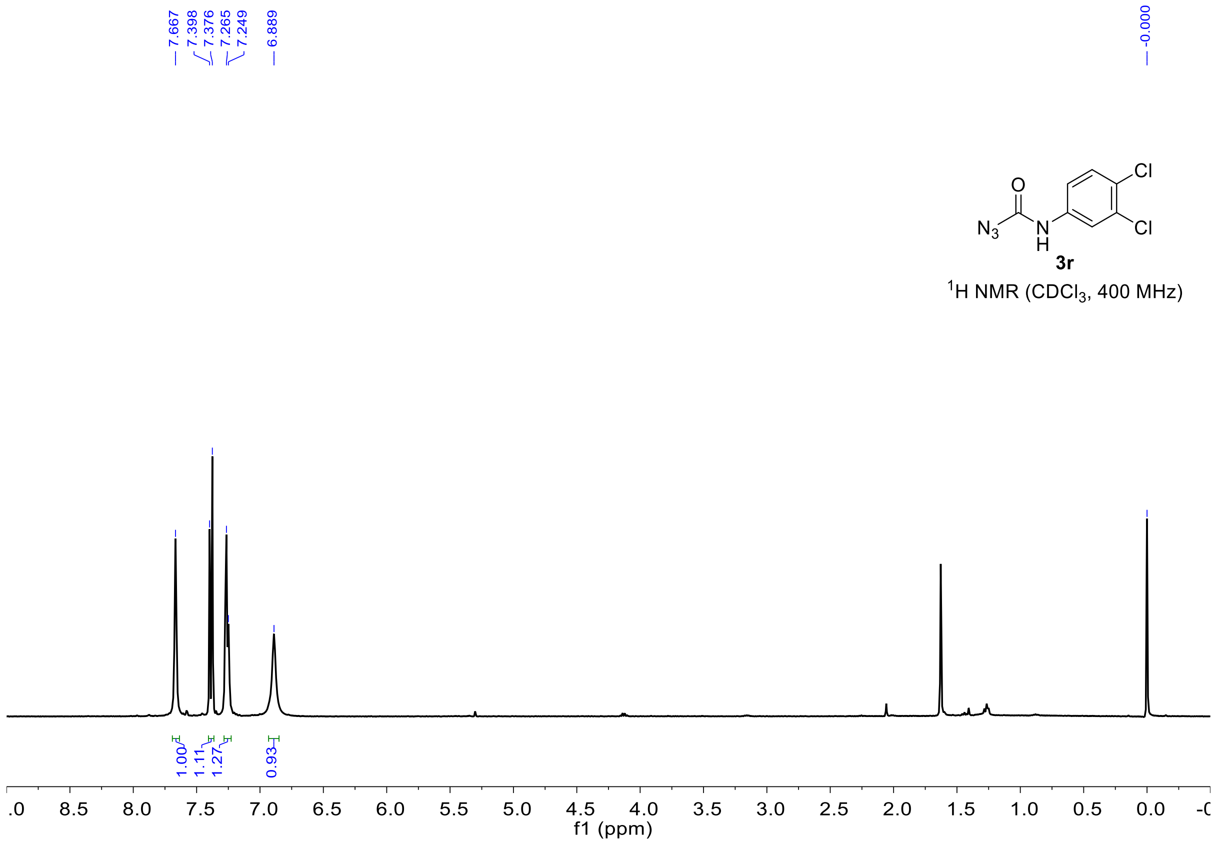
7.667
7.398
7.376
7.265
7.249
6.889
0.000
O
N3
N
H
Cl
Cl
3r
1H NMR (CDCl3, 400 MHz)
1.00
1.11
1.27
0.93
f1 (ppm)

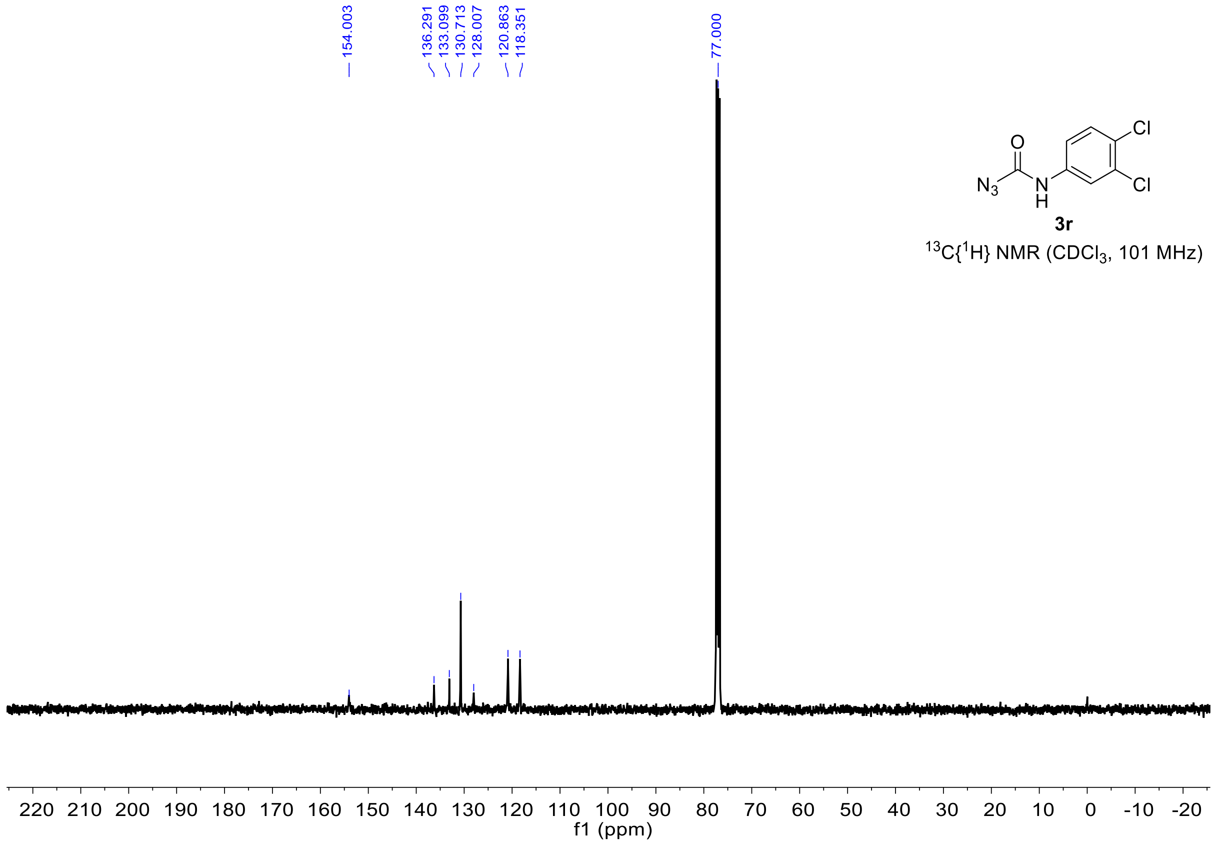
154.003
136.291
133.099
130.713
128.007
120.863
118.351
77.000
O
N3
N
H
Cl
Cl
3r
13C{1H} NMR (CDCl3, 101 MHz)
f1 (ppm)

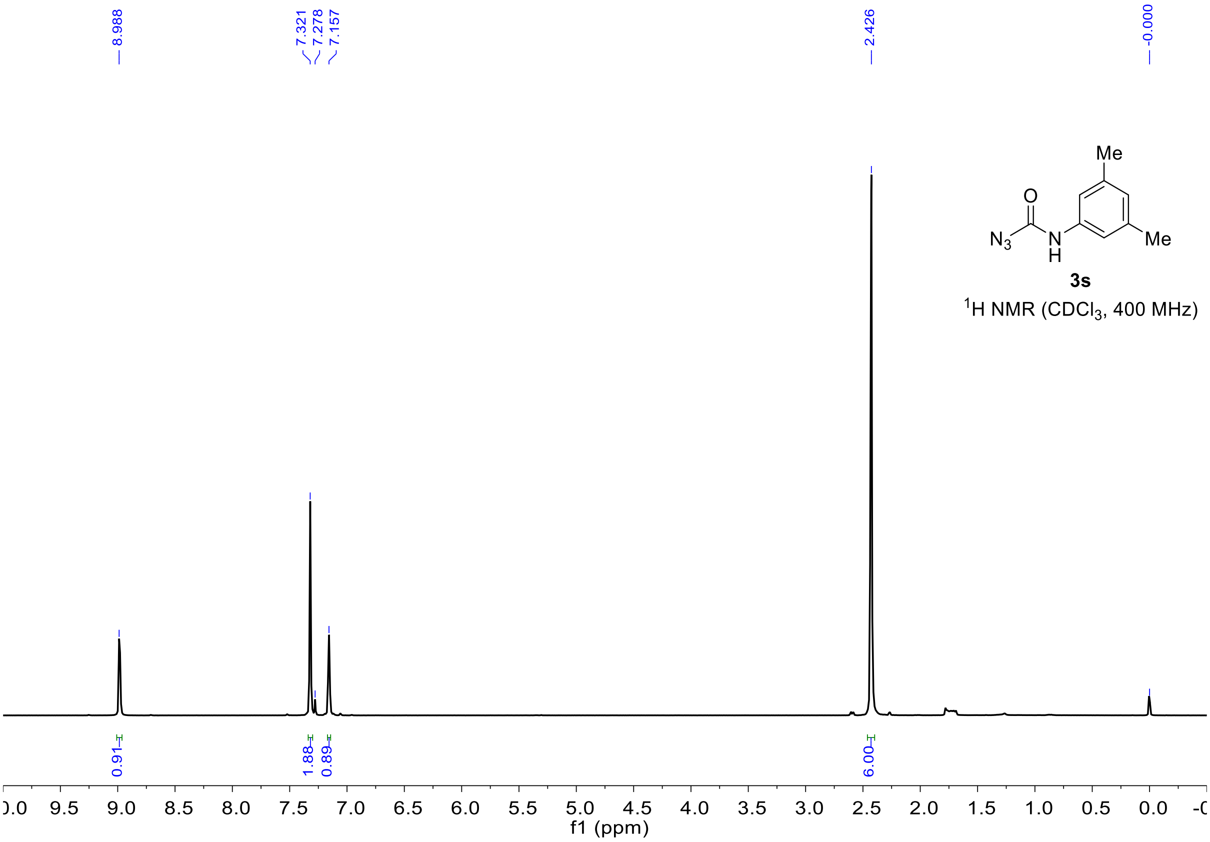
8.988
7.321
7.278
7.157
2.426
-0.000
Me
O
N3
N
H
Me
3s
1H NMR (CDCl3, 400 MHz)
0.91
1.88
0.89
6.00
f1 (ppm)

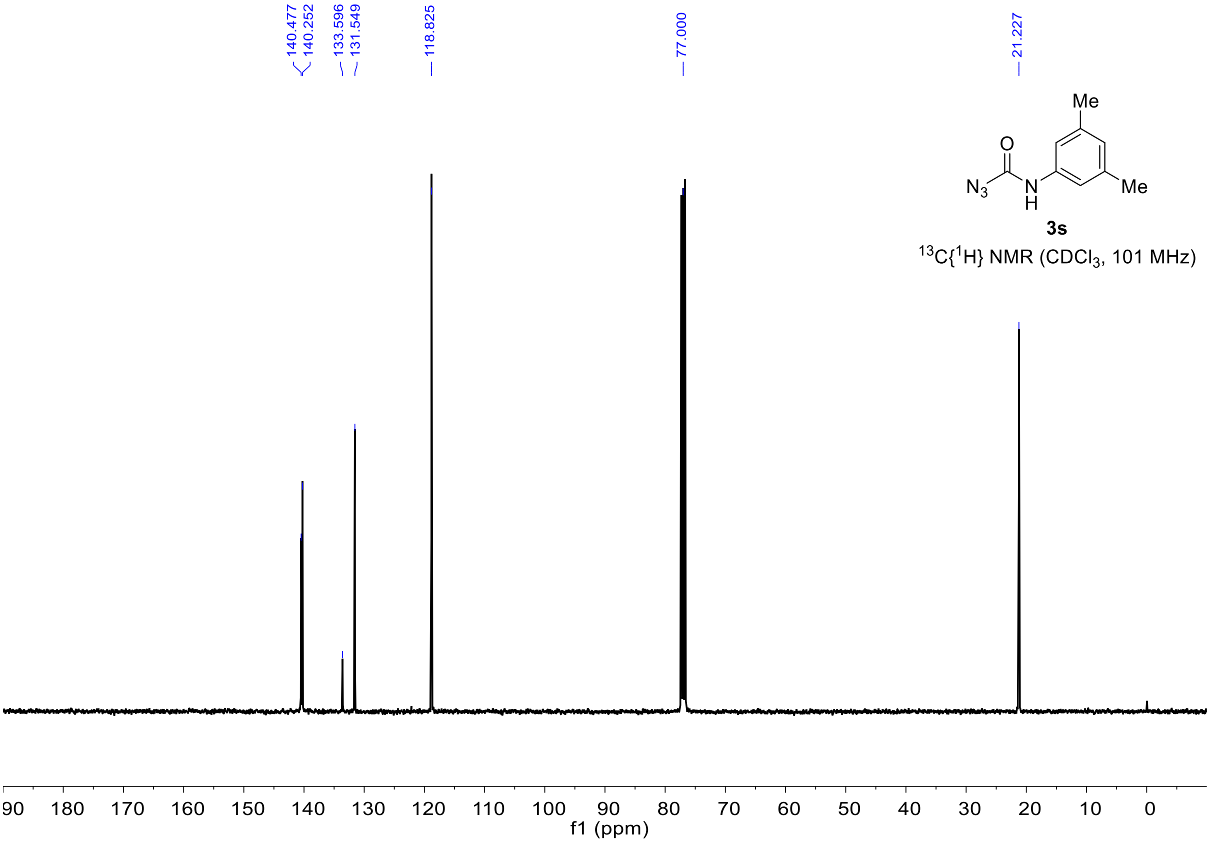
140.477
140.252
133.596
131.549
118.825
77.000
21.227
Me
O
N3
N
H
Me
3s
13C{1H} NMR (CDCl3, 101 MHz)
f1 (ppm)

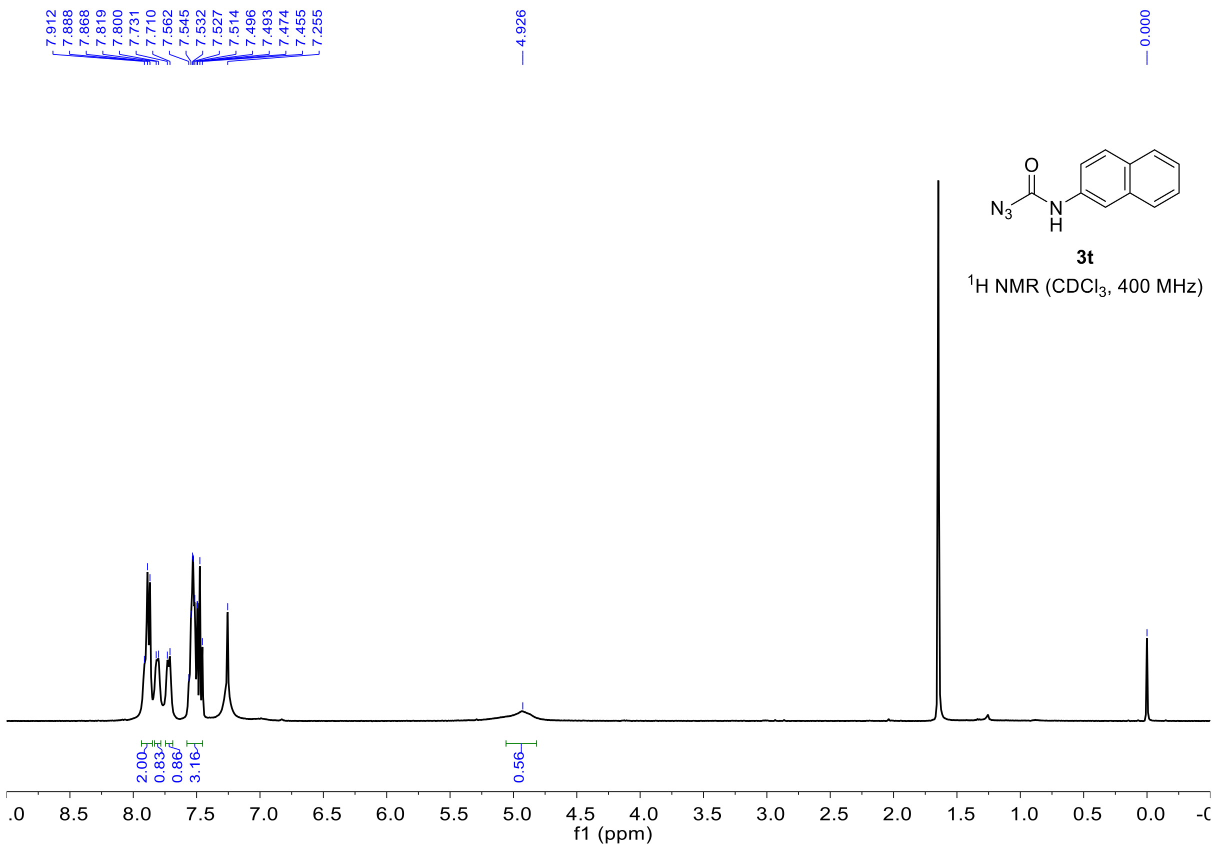

7.912
7.888
7.868
7.819
7.800
7.731
7.710
7.562
7.545
7.532
7.527
7.514
7.496
7.493
7.474
7.455
7.255
4.926
0.000
N3
O
N
H
3t
1H NMR (CDCl3, 400 MHz)
2.00
0.83
0.86
3.16
0.56
f1 (ppm)

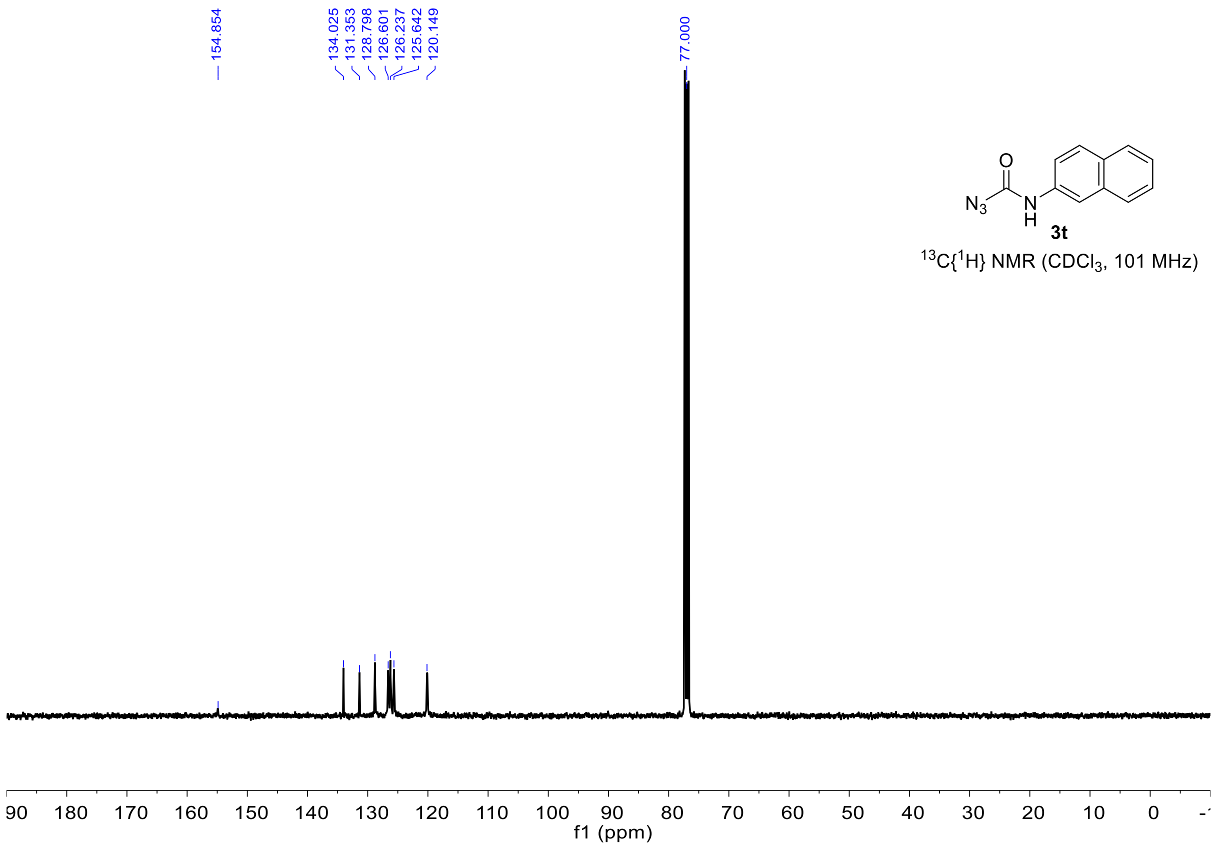

154.854
134.025
131.353
128.798
126.601
126.237
125.642
120.149
77.000
N3
O
N
H
3t
13C{1H} NMR (CDCl3, 101 MHz)
f1 (ppm)

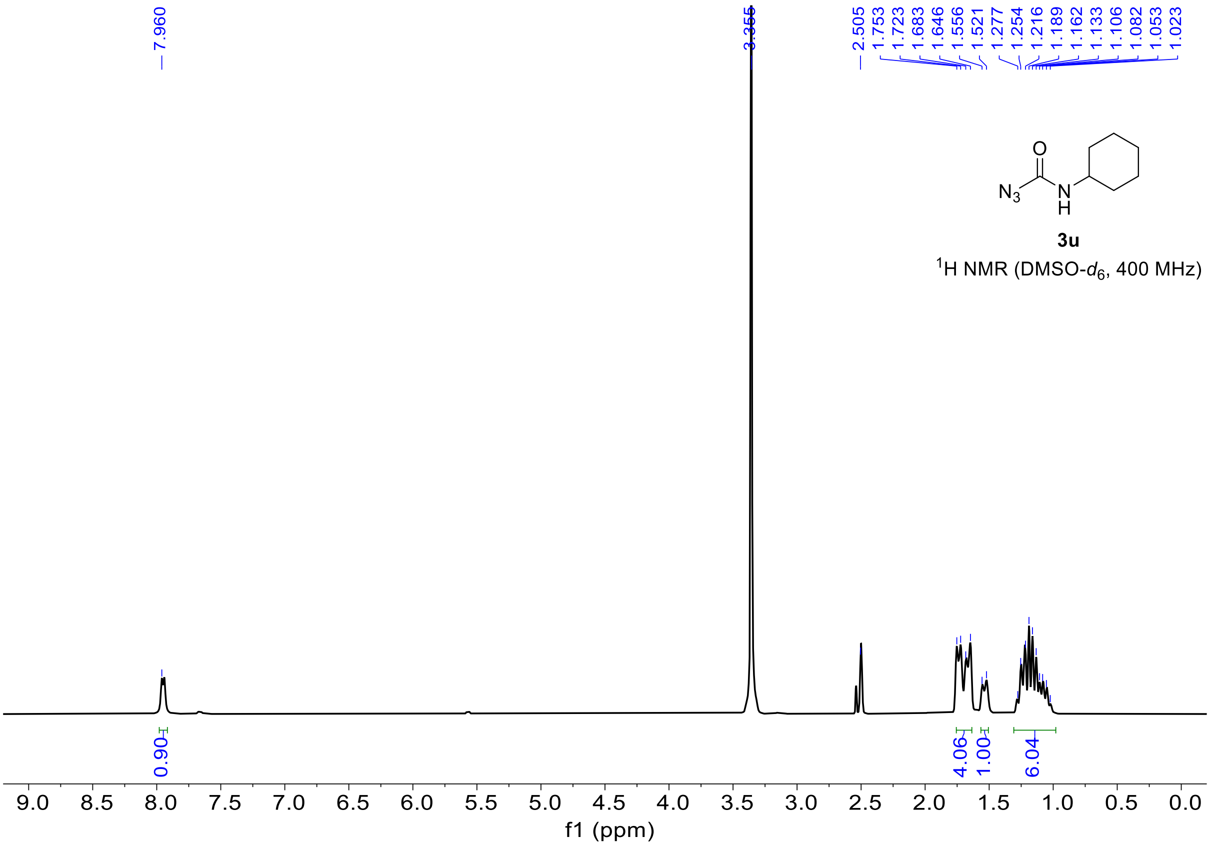

3u

^{1}H NMR (DMSO-d_6, 400 MHz)

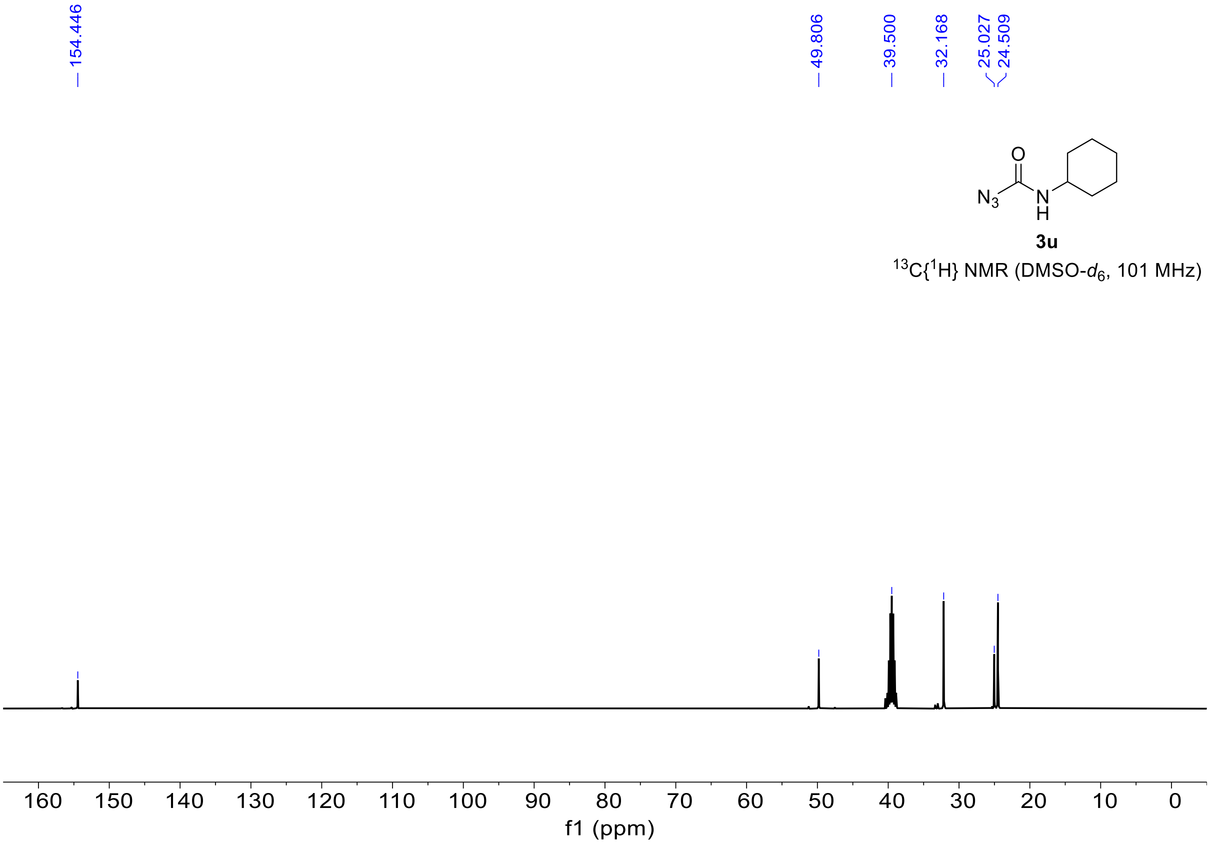

3u

^{13}C{^{1}H} NMR (DMSO-d_6, 101 MHz)

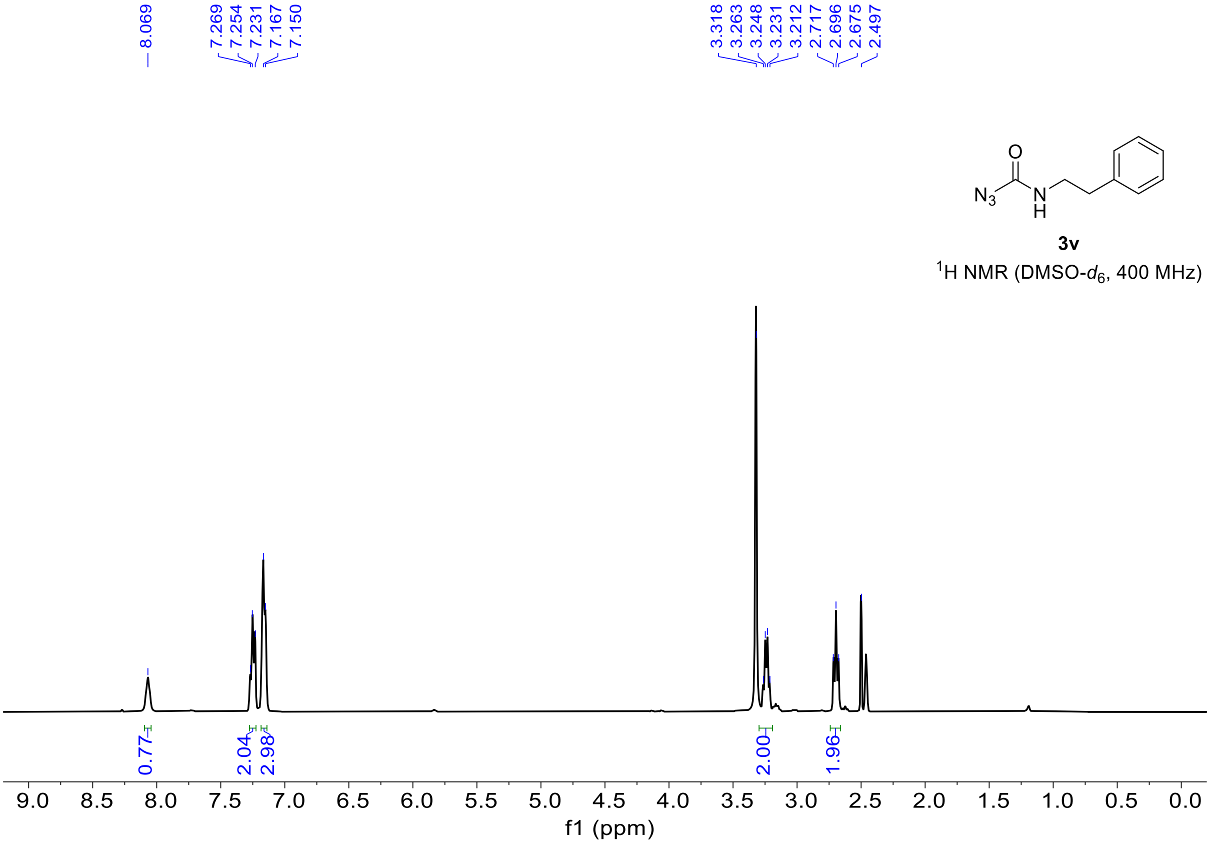
8.069
7.269
7.254
7.231
7.167
7.150
3.318
3.263
3.248
3.231
3.212
2.717
2.696
2.675
2.497
3v
^{1}H NMR (DMSO-d_6, 400 MHz)
0.77
2.04
2.98
2.00
1.96
f1 (ppm)

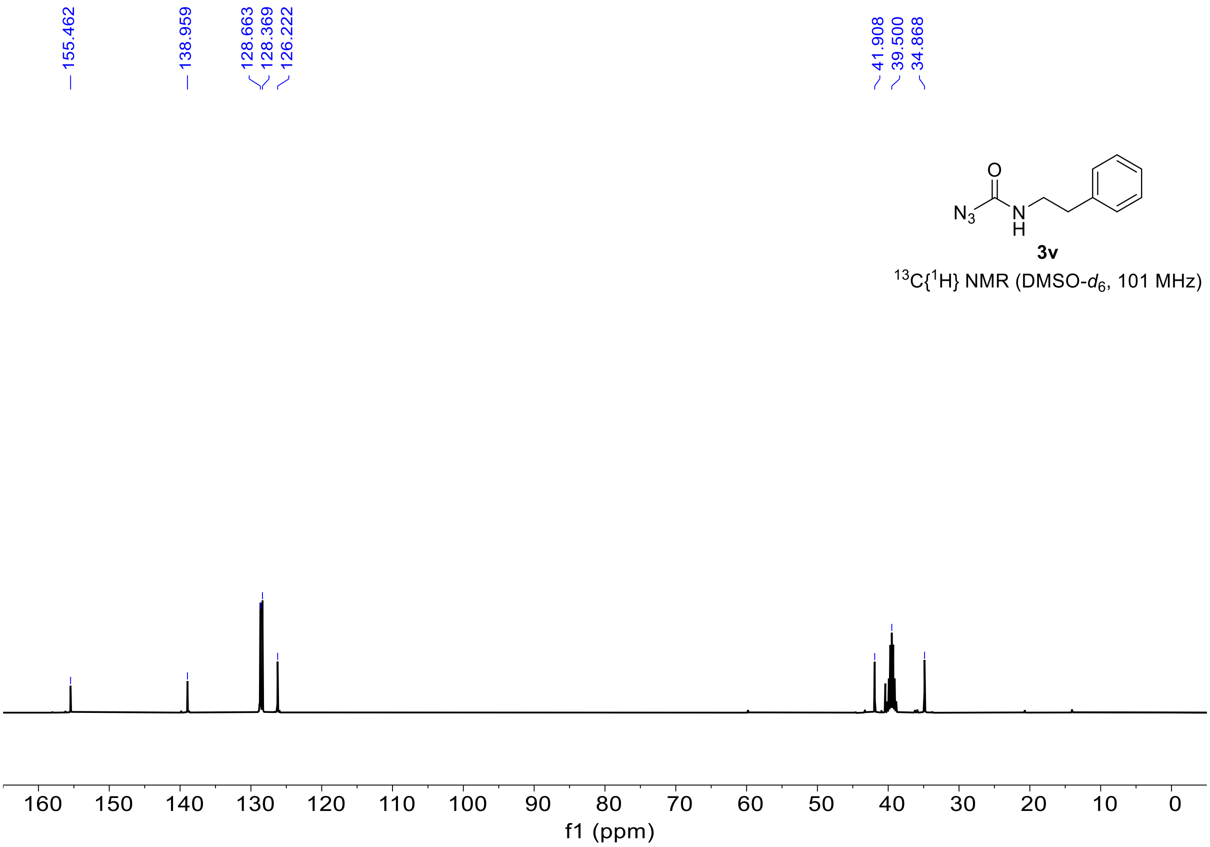
155.462
138.959
128.663
128.369
126.222
41.908
39.500
34.868
3v
^{13}C{^{1}H} NMR (DMSO-d_6, 101 MHz)
f1 (ppm)

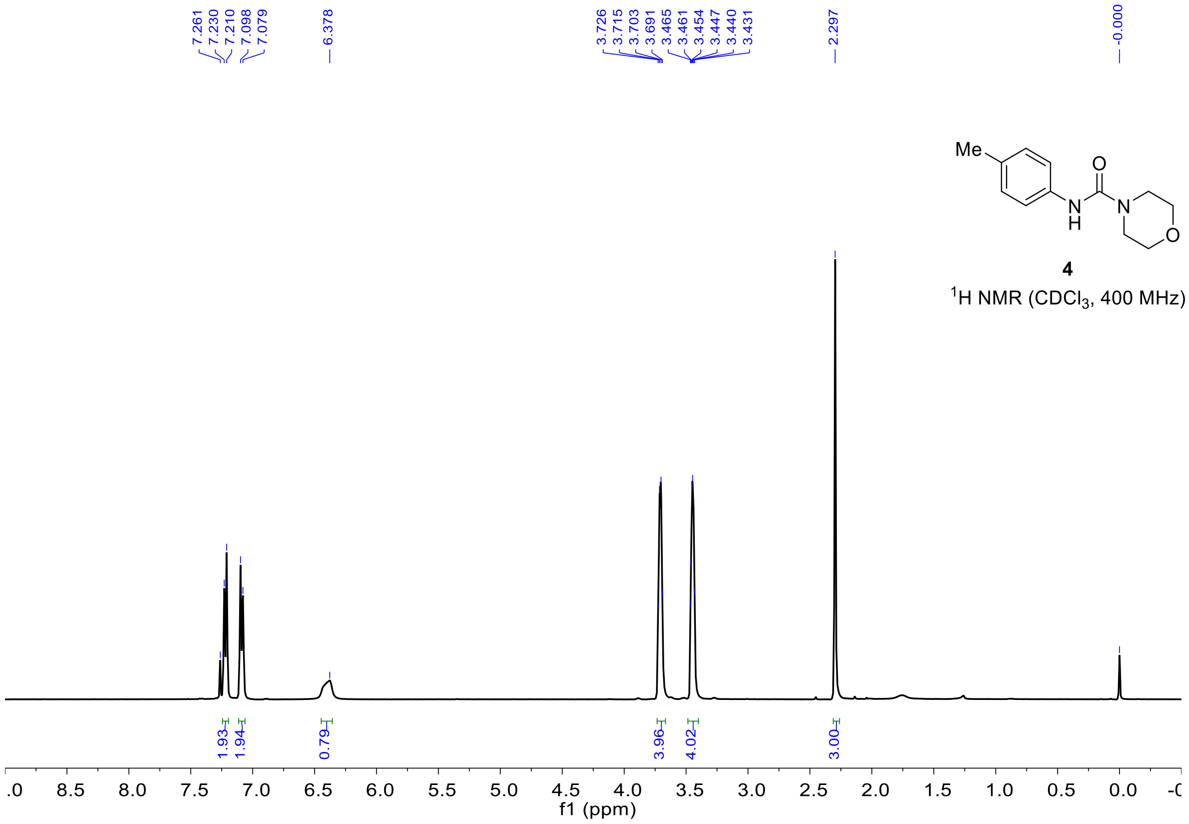
7.261
7.230
7.210
7.098
7.079
6.378
3.726
3.715
3.703
3.691
3.465
3.461
3.454
3.447
3.440
3.431
2.297
-0.000
Me
O
N
H
N
O
4
^{1}H NMR ($CDCl_3$, 400 MHz)
1.93
1.94
0.79
3.96
4.02
3.00
f1 (ppm)

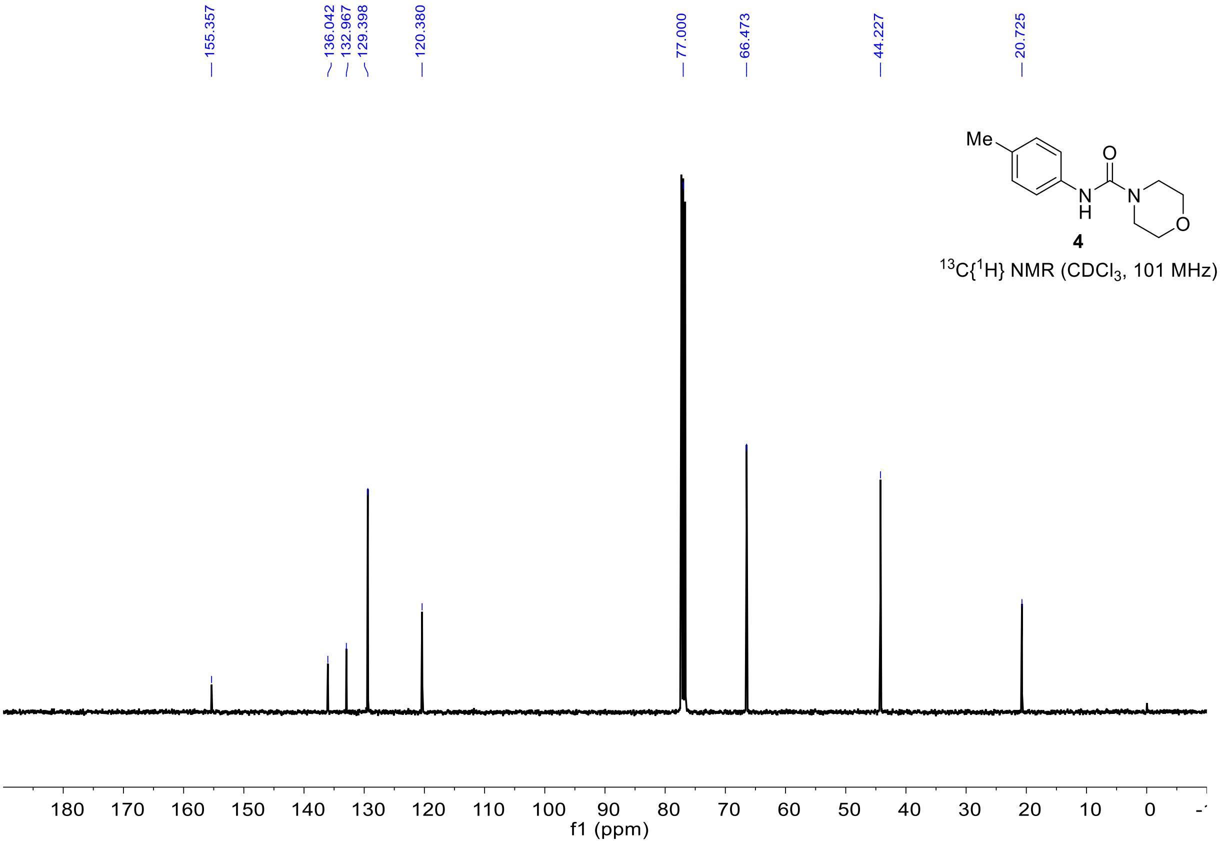
155.357
136.042
132.967
129.398
120.380
77.000
66.473
44.227
20.725
Me
O
N
H
N
O
4
^{13}C{^{1}H} NMR ($CDCl_3$, 101 MHz)
f1 (ppm)

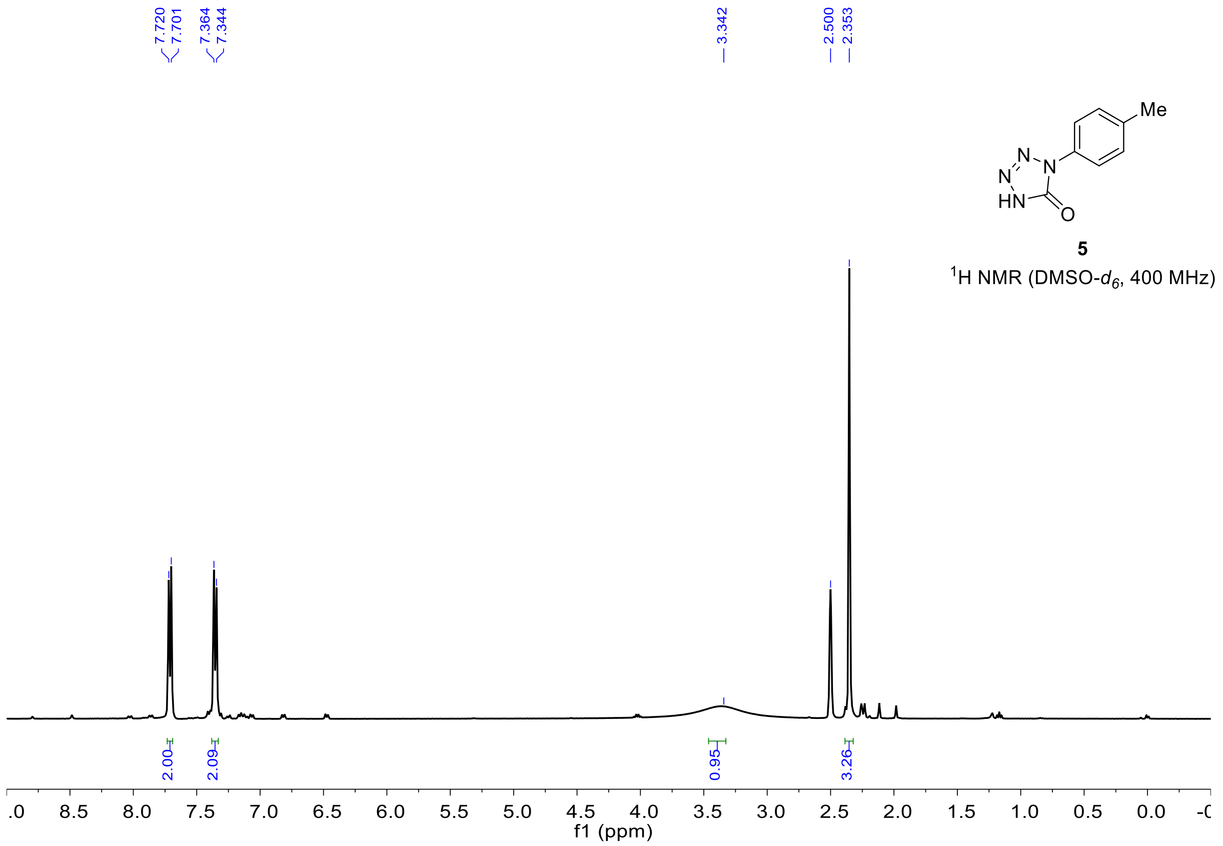
7.720
7.701
7.364
7.344
3.342
2.500
2.353
Me
N
N
N
HN
O
5
^{1}H NMR (DMSO-d_6, 400 MHz)
2.00
2.09
0.95
3.26
f1 (ppm)

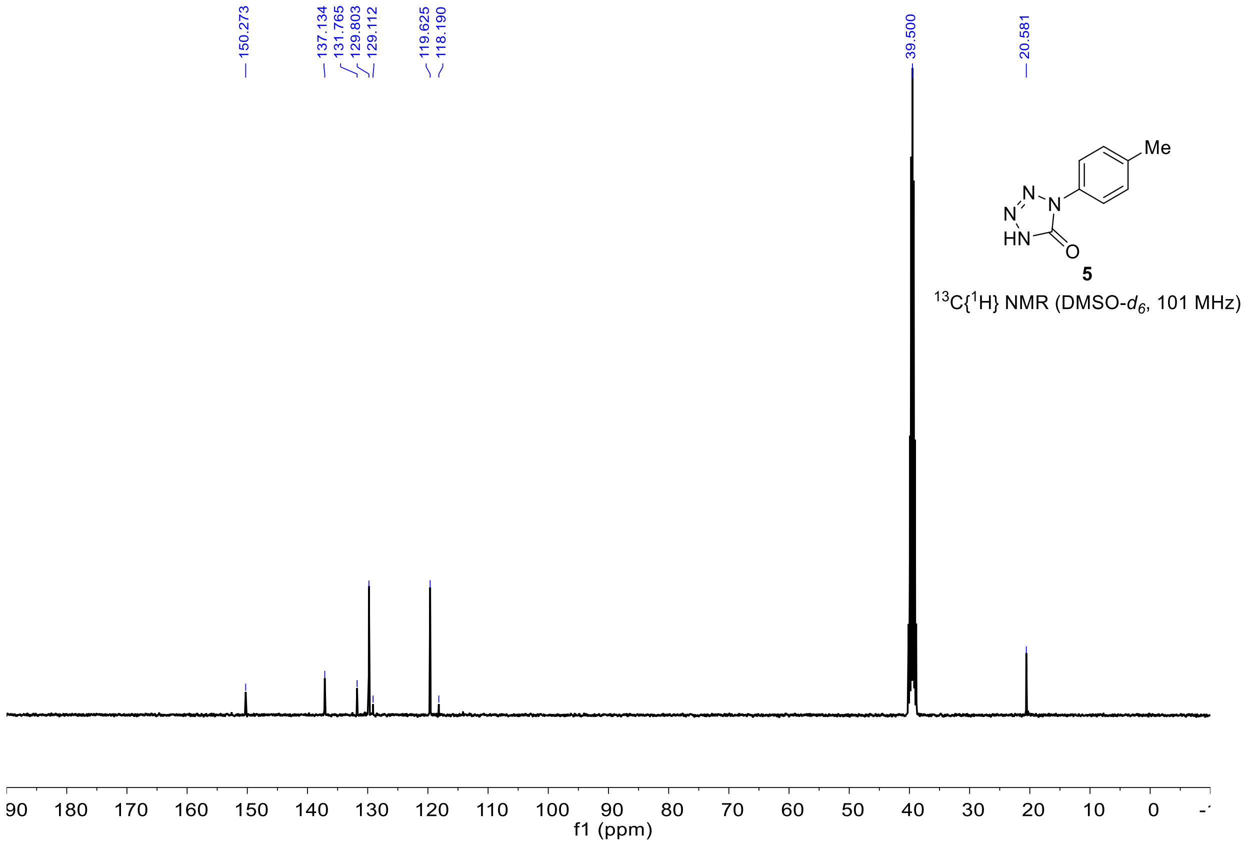
150.273
137.134
131.765
129.803
129.112
119.625
118.190
39.500
20.581
Me
N
N
N
HN
O
5
^{13}C{^{1}H} NMR (DMSO-d_6, 101 MHz)
f1 (ppm)

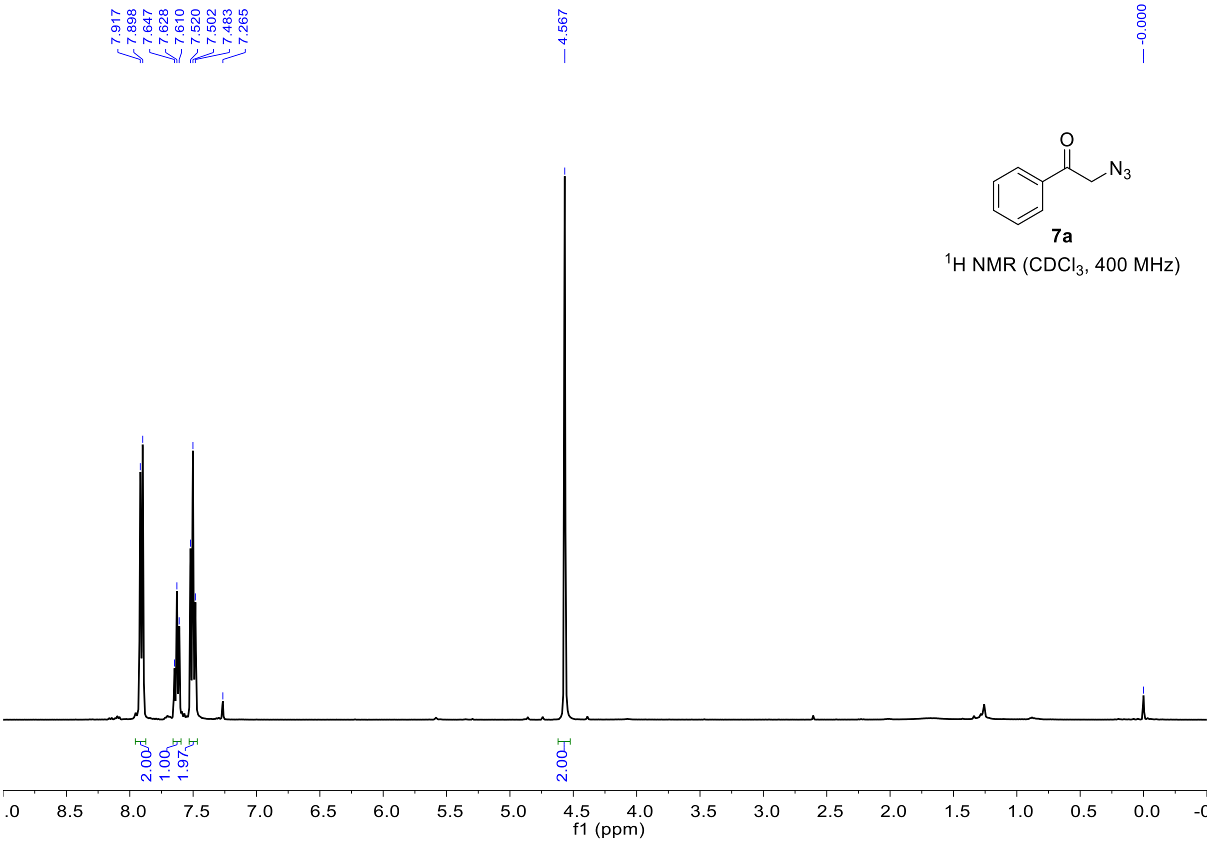
7.917
7.898
7.647
7.628
7.610
7.520
7.502
7.483
7.265
4.567
-0.000
O
N3
7a
1H NMR (CDCl3, 400 MHz)
2.00
1.00
1.97
2.00
f1 (ppm)

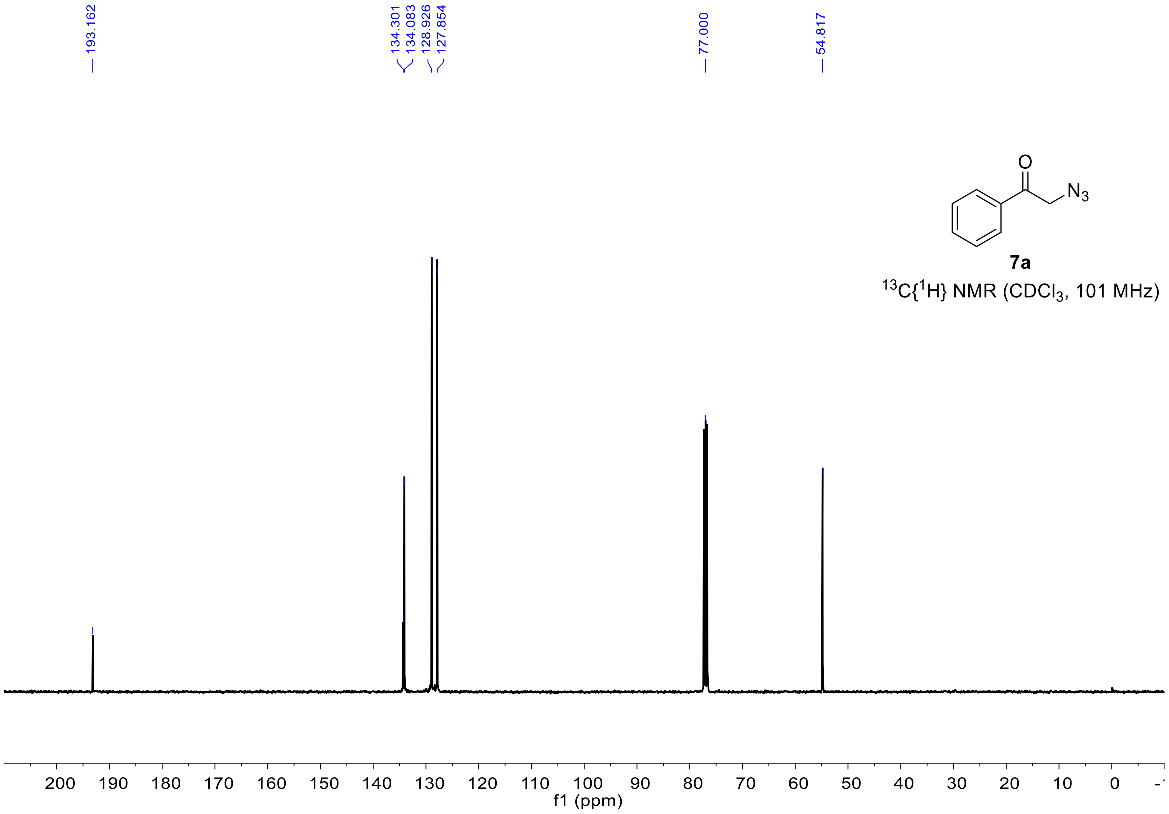
193.162
134.301
134.083
128.926
127.854
77.000
54.817
O
N3
7a
13C{1H} NMR (CDCl3, 101 MHz)
f1 (ppm)

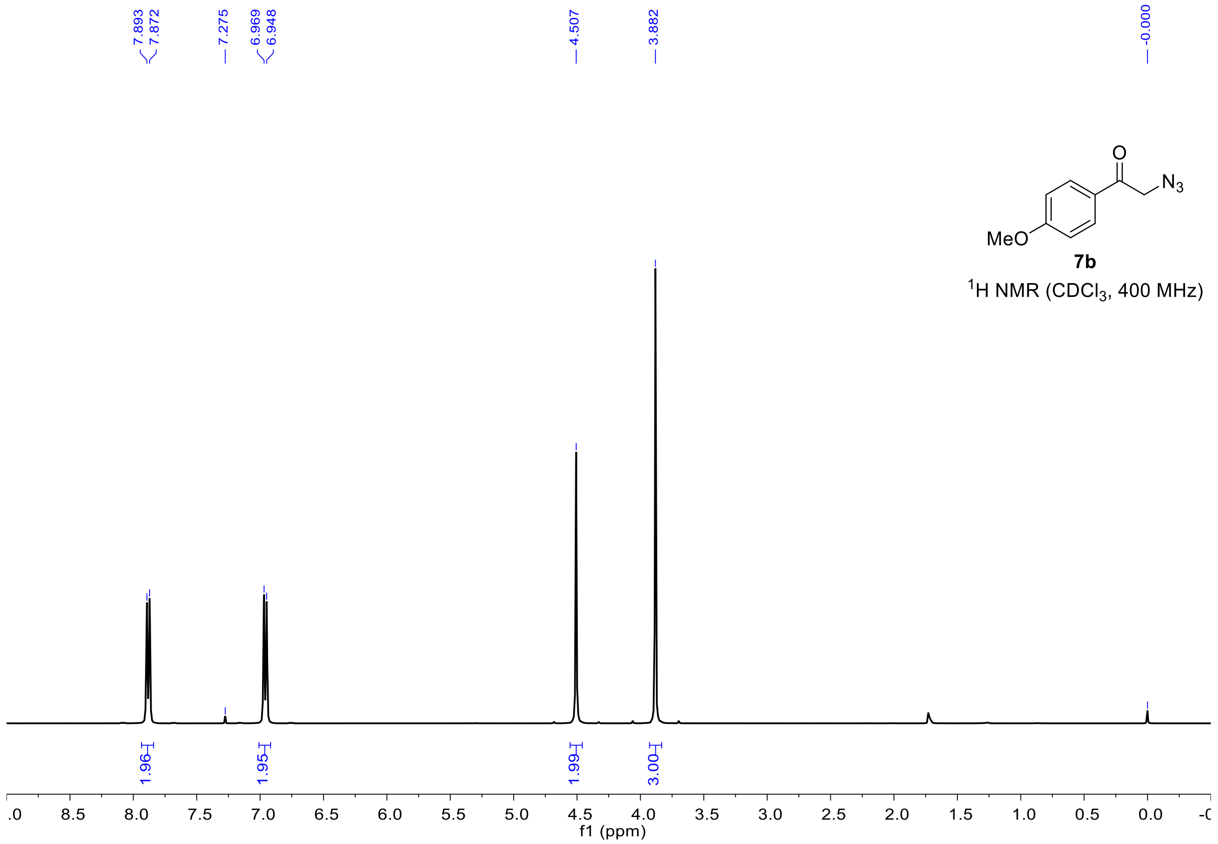
7.893
7.872
7.275
6.969
6.948
4.507
3.882
-0.000
O
N3
MeO
7b
1H NMR (CDCl3, 400 MHz)
1.96
1.95
1.99
3.00
f1 (ppm)

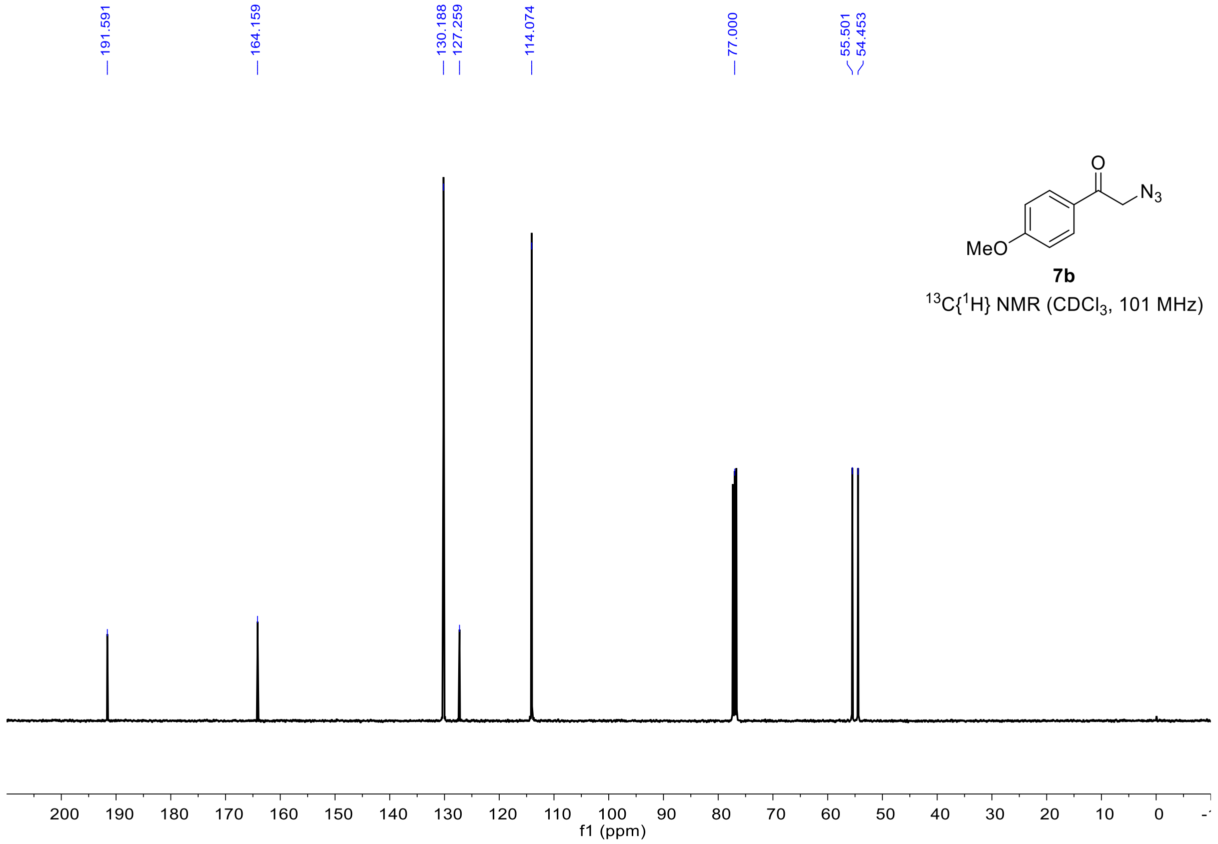
191.591
164.159
130.188
127.259
114.074
77.000
55.501
54.453
O
N3
MeO
7b
13C{1H} NMR (CDCl3, 101 MHz)
f1 (ppm)

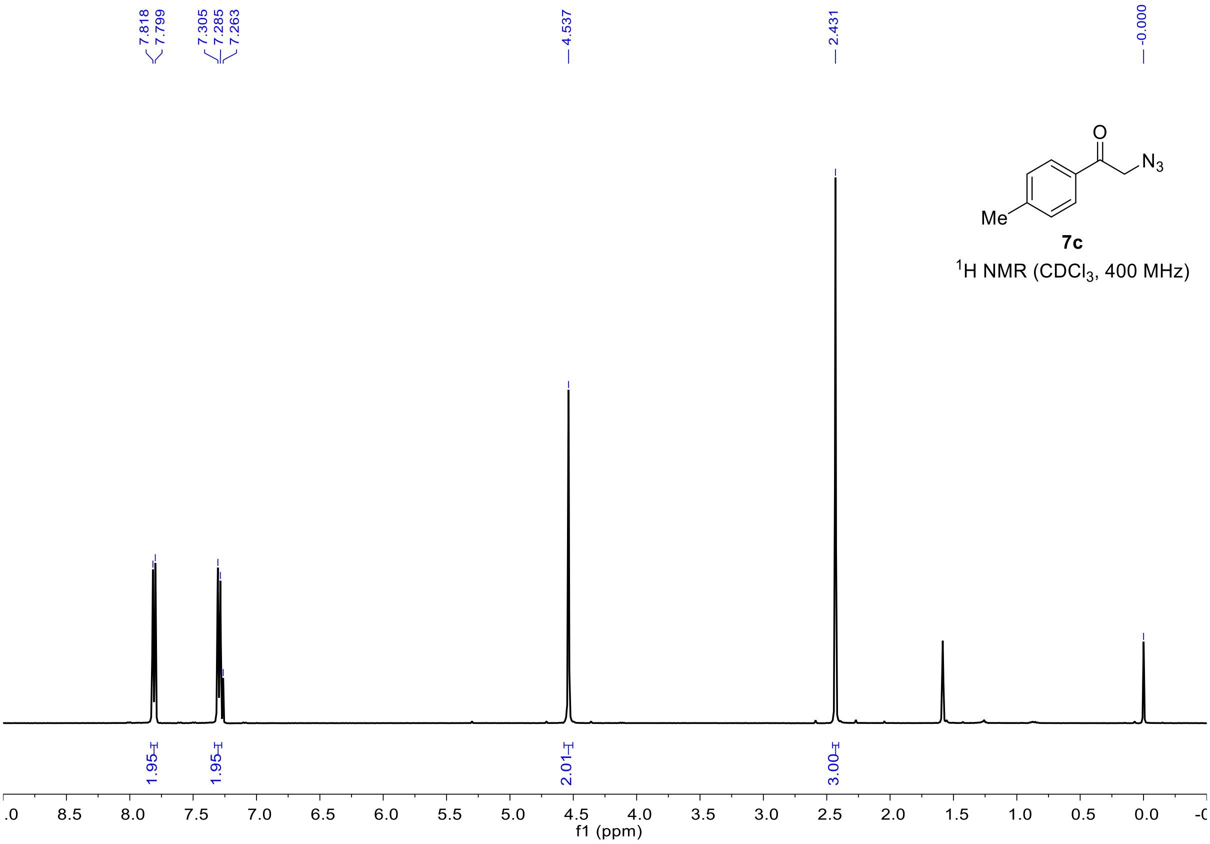
7.818
7.799
7.305
7.285
7.263
4.537
2.431
-0.000
O
N3
Me
7c
1H NMR (CDCl3, 400 MHz)
1.95
1.95
2.01
3.00
f1 (ppm)

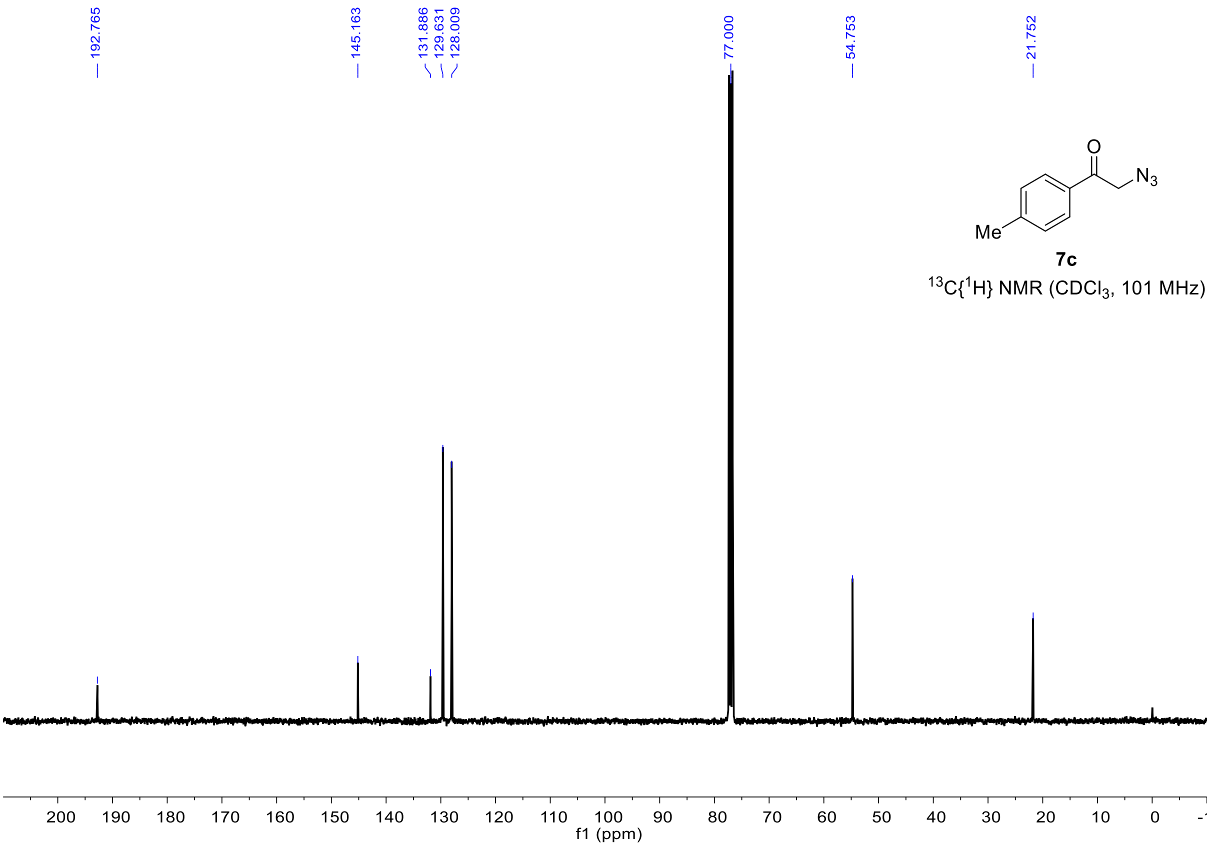
192.765
145.163
131.886
129.631
128.009
77.000
54.753
21.752
O
N3
Me
7c
13C{1H} NMR (CDCl3, 101 MHz)
f1 (ppm)

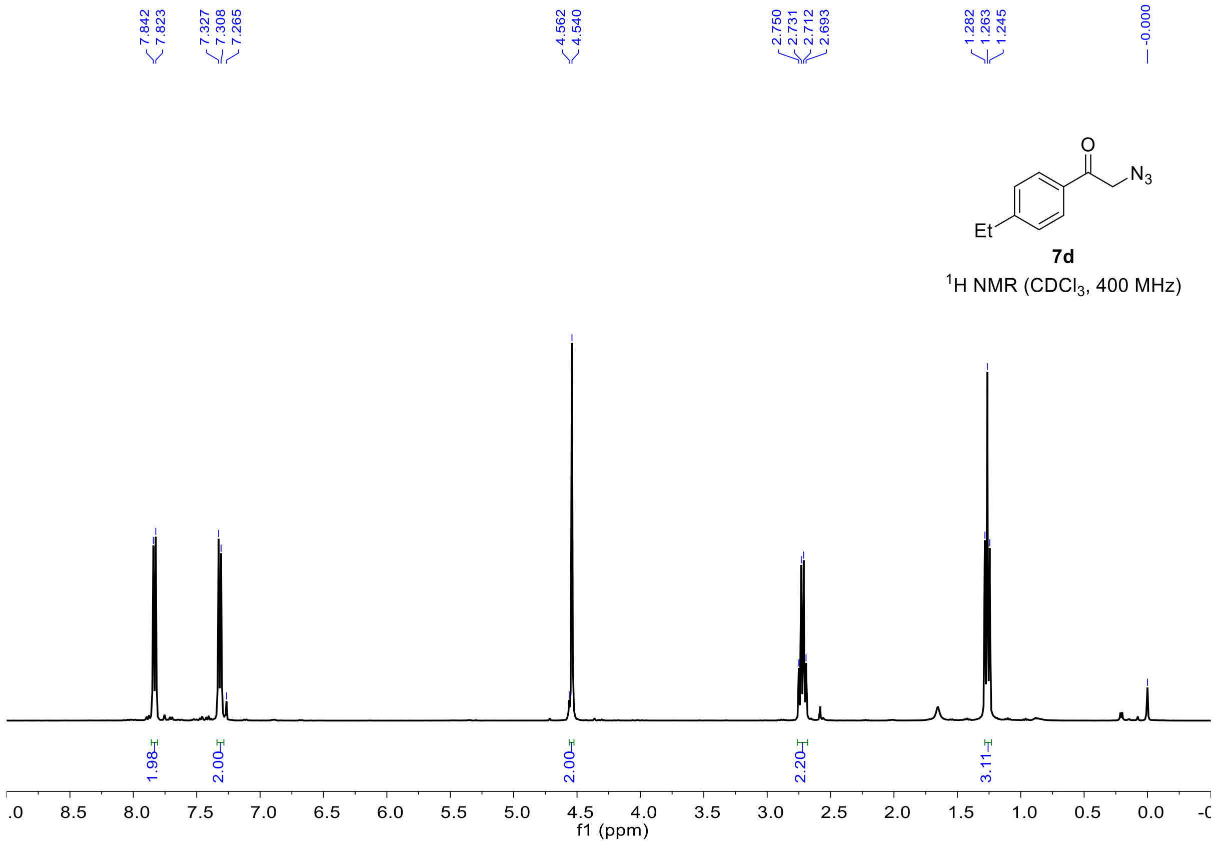

7d

^{1}H NMR ($CDCl_3$, 400 MHz)

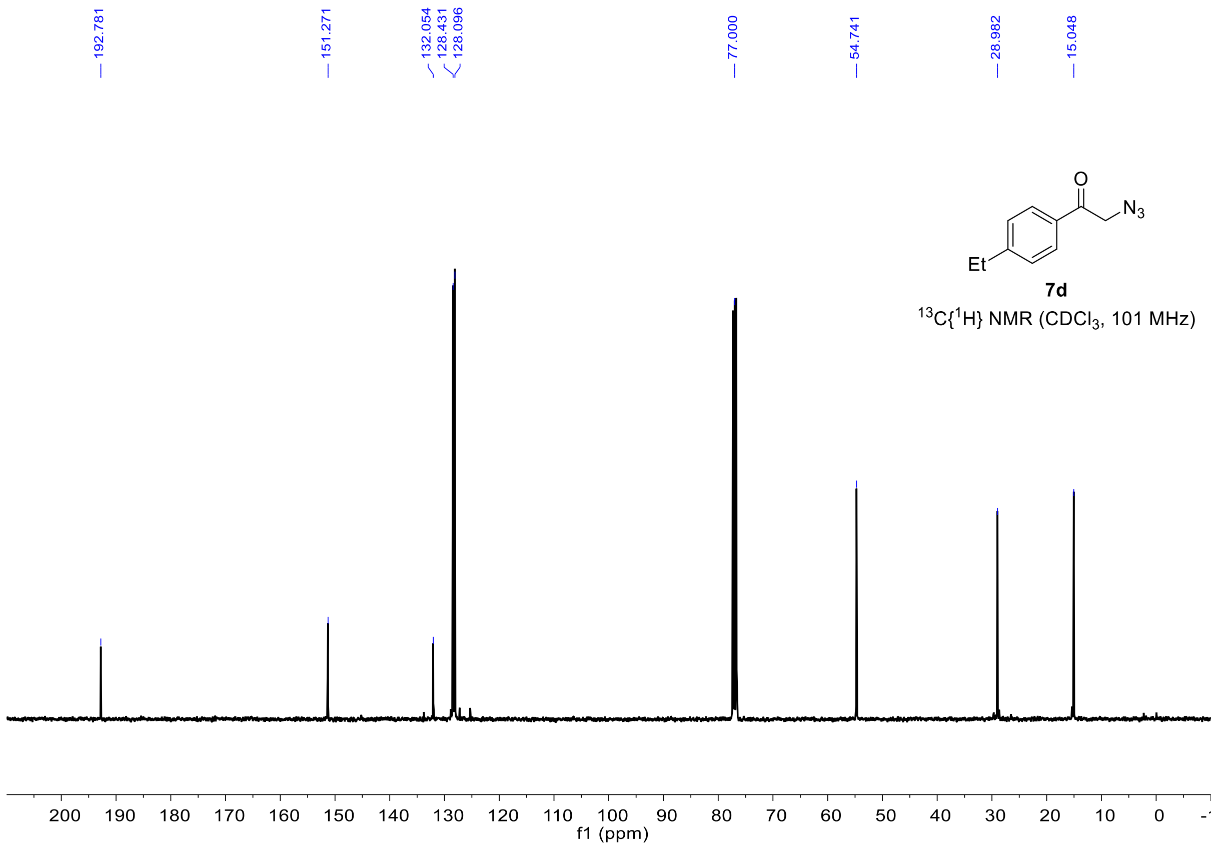

7d

^{13}C{^{1}H} NMR ($CDCl_3$, 101 MHz)

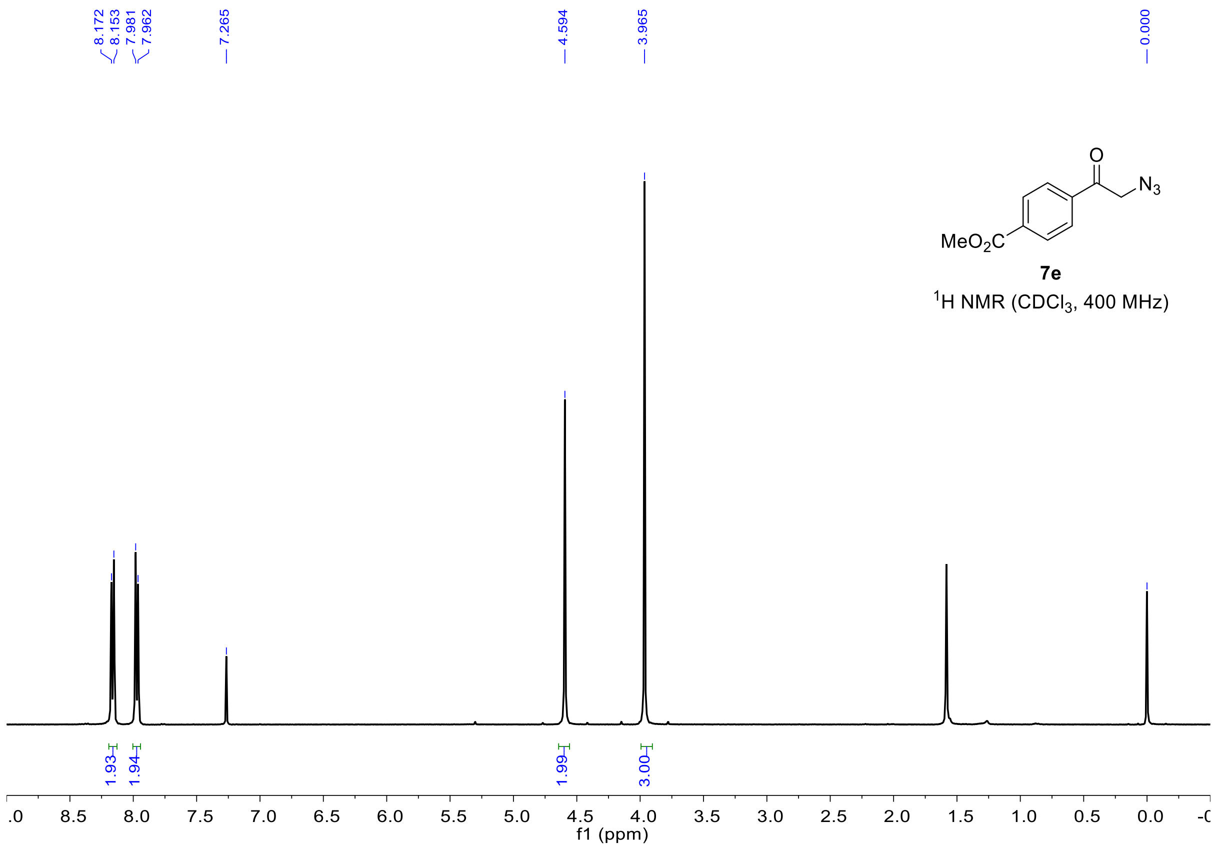

7e

^{1}H NMR (CDCl$_3$, 400 MHz)

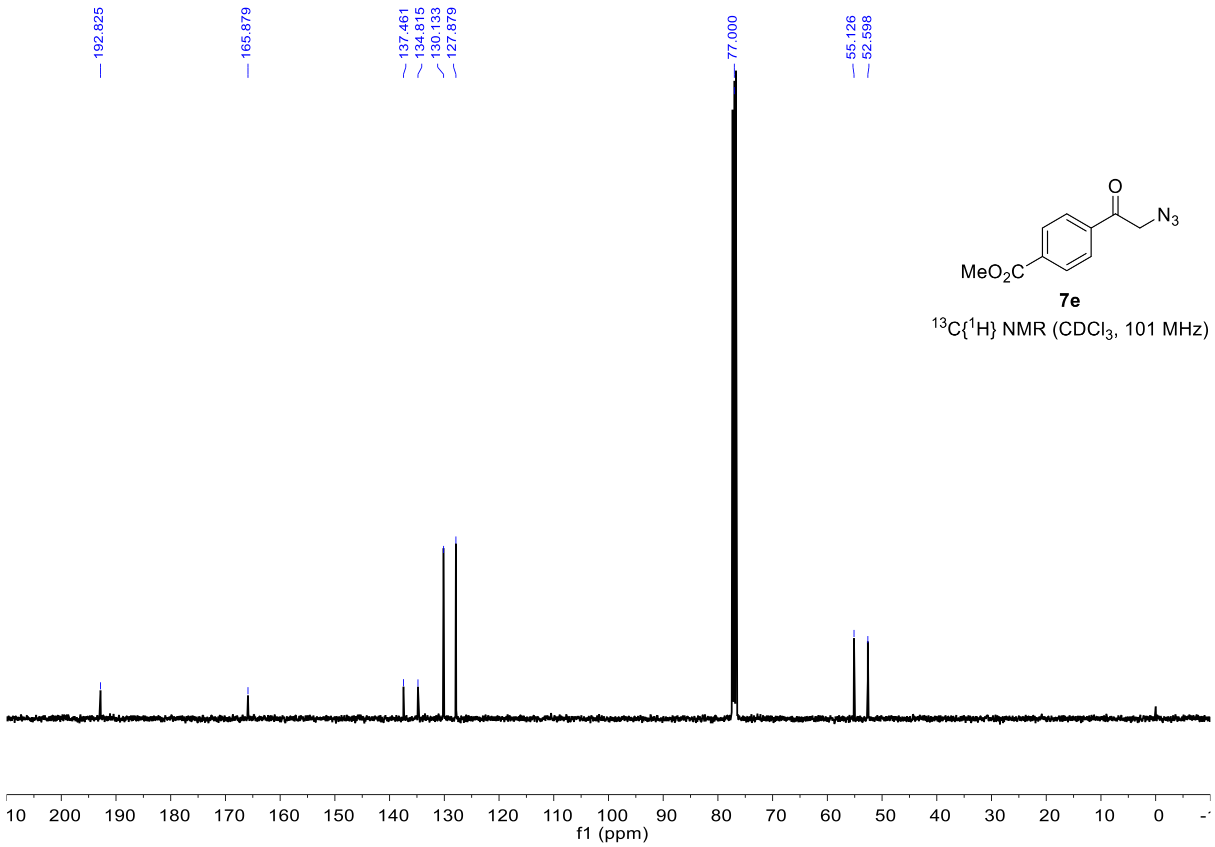

7e

^{13}C{^{1}H} NMR (CDCl$_3$, 101 MHz)

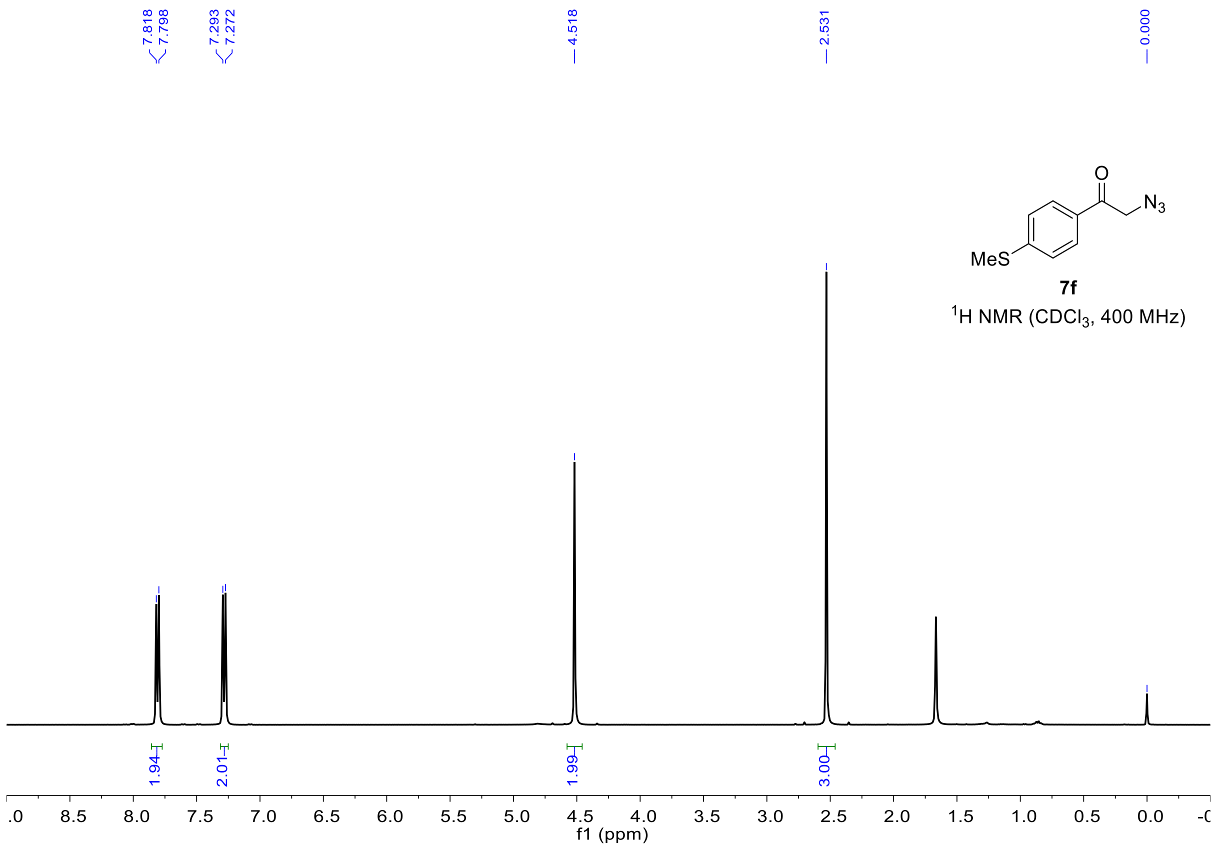
7.818
7.798
7.293
7.272
4.518
2.531
0.000
O
N3
MeS
7f
1H NMR (CDCl3, 400 MHz)
1.94
2.01
1.99
3.00
f1 (ppm)

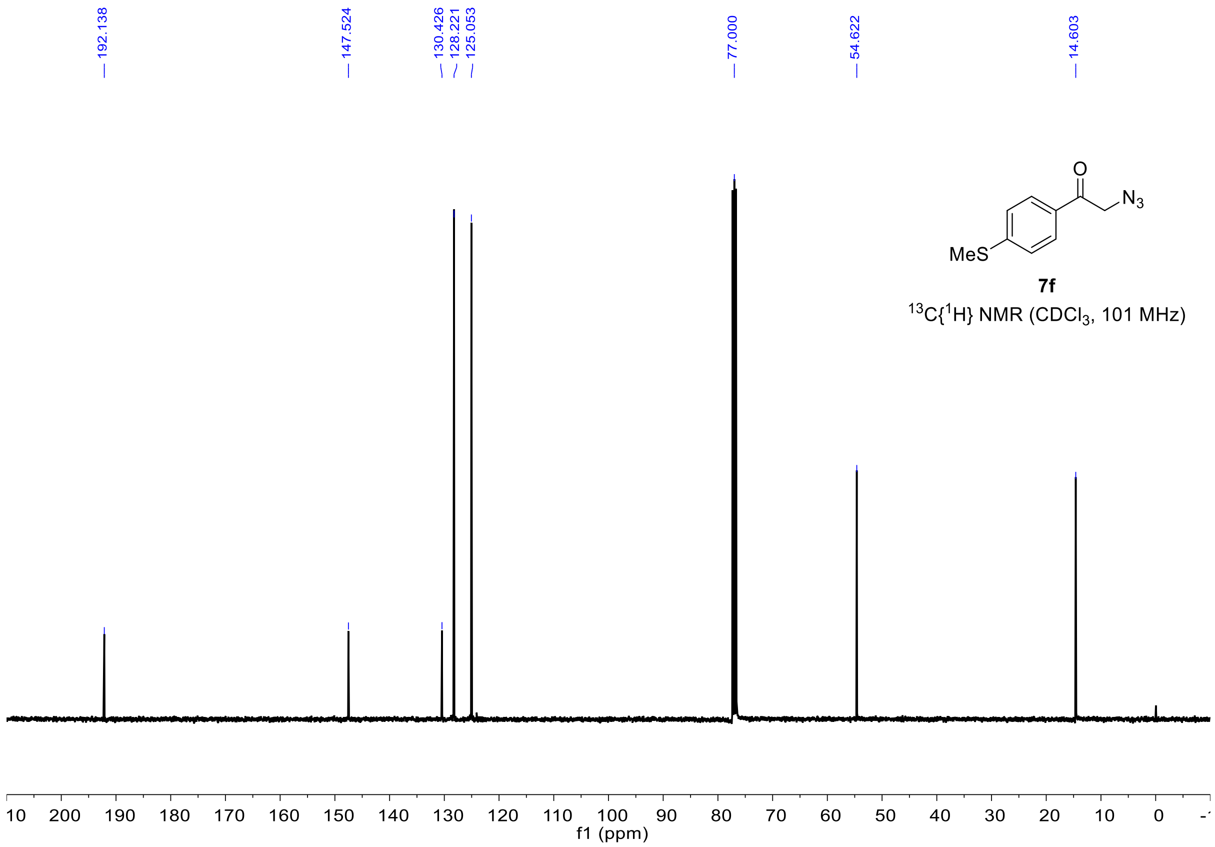
192.138
147.524
130.426
128.221
125.053
77.000
54.622
14.603
O
N3
MeS
7f
13C{1H} NMR (CDCl3, 101 MHz)
f1 (ppm)

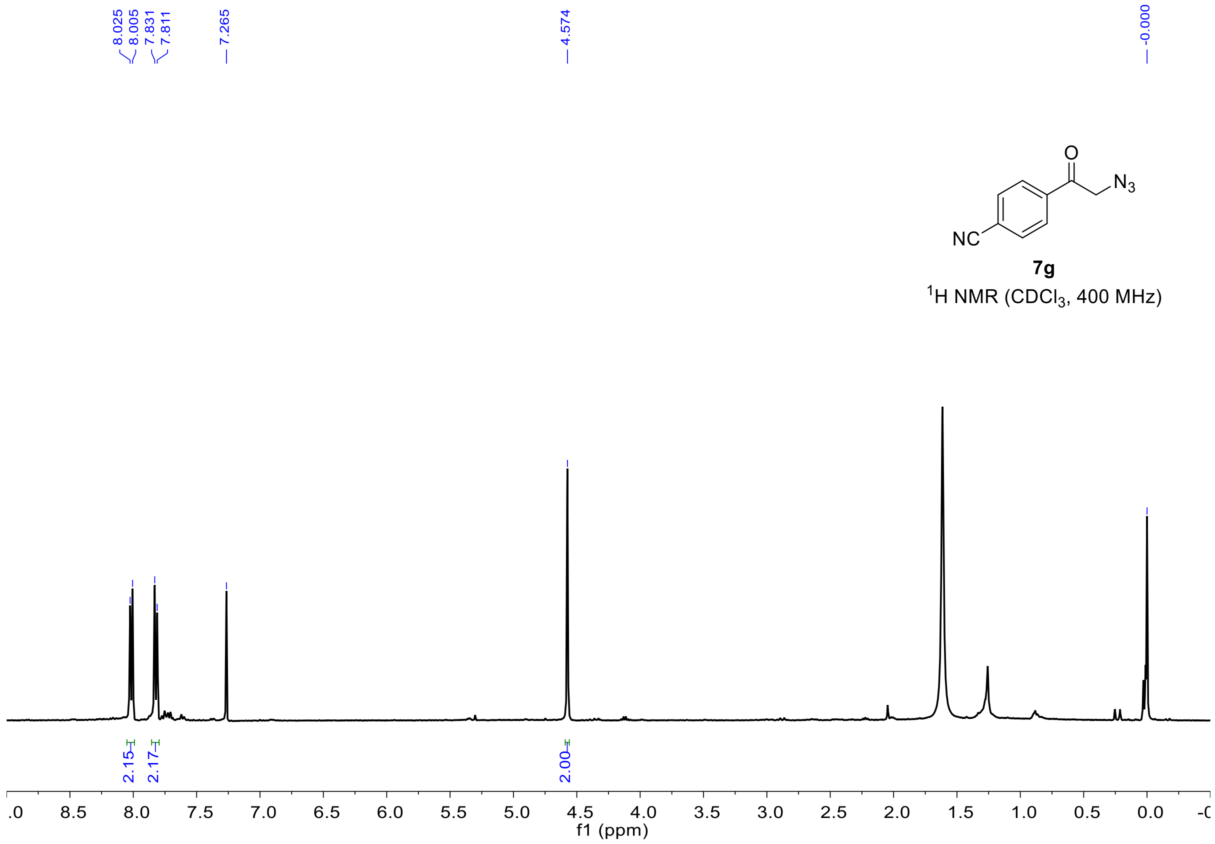

8.025
8.005
7.831
7.811
7.265
4.574
-0.000
O
N3
NC
7g
1H NMR (CDCl3, 400 MHz)
2.15
2.17
2.00
f1 (ppm)

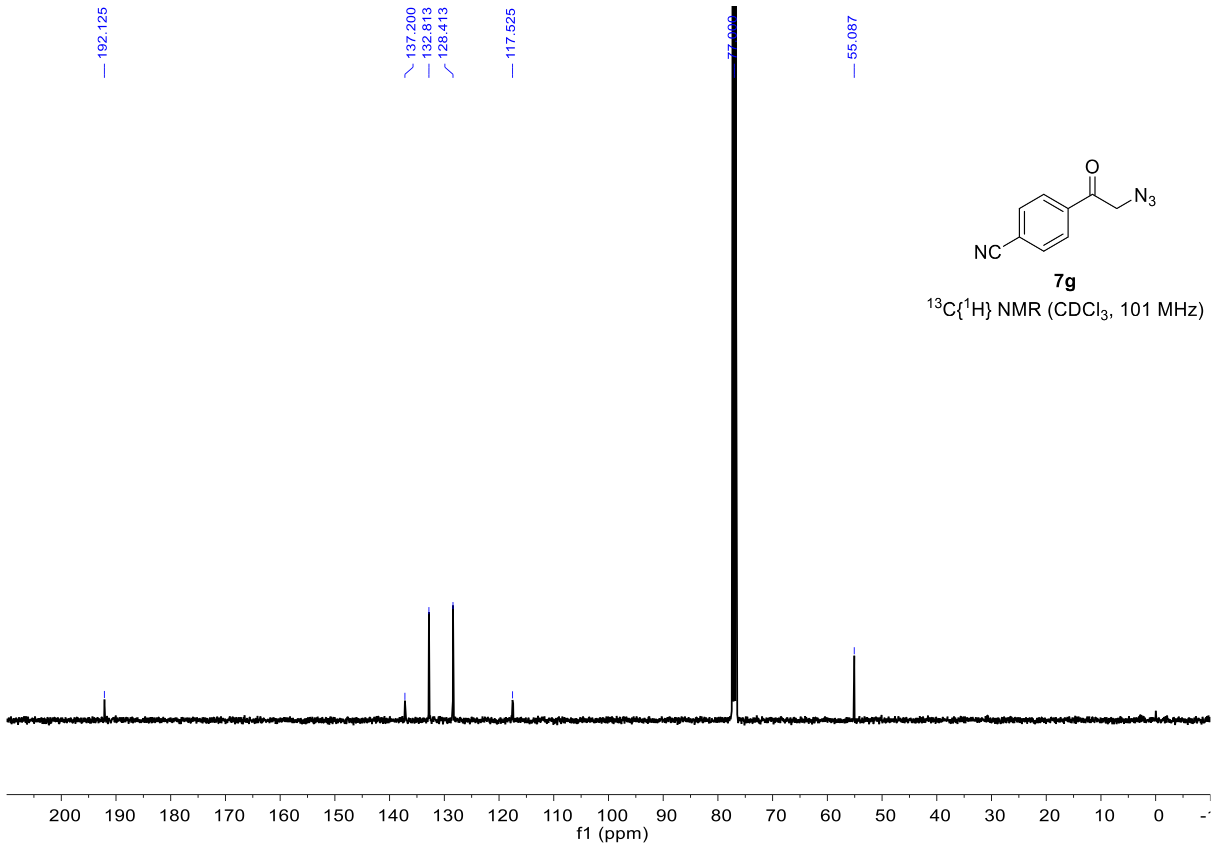

192.125
137.200
132.813
128.413
117.525
55.087
O
N3
NC
7g
13C{1H} NMR (CDCl3, 101 MHz)
f1 (ppm)

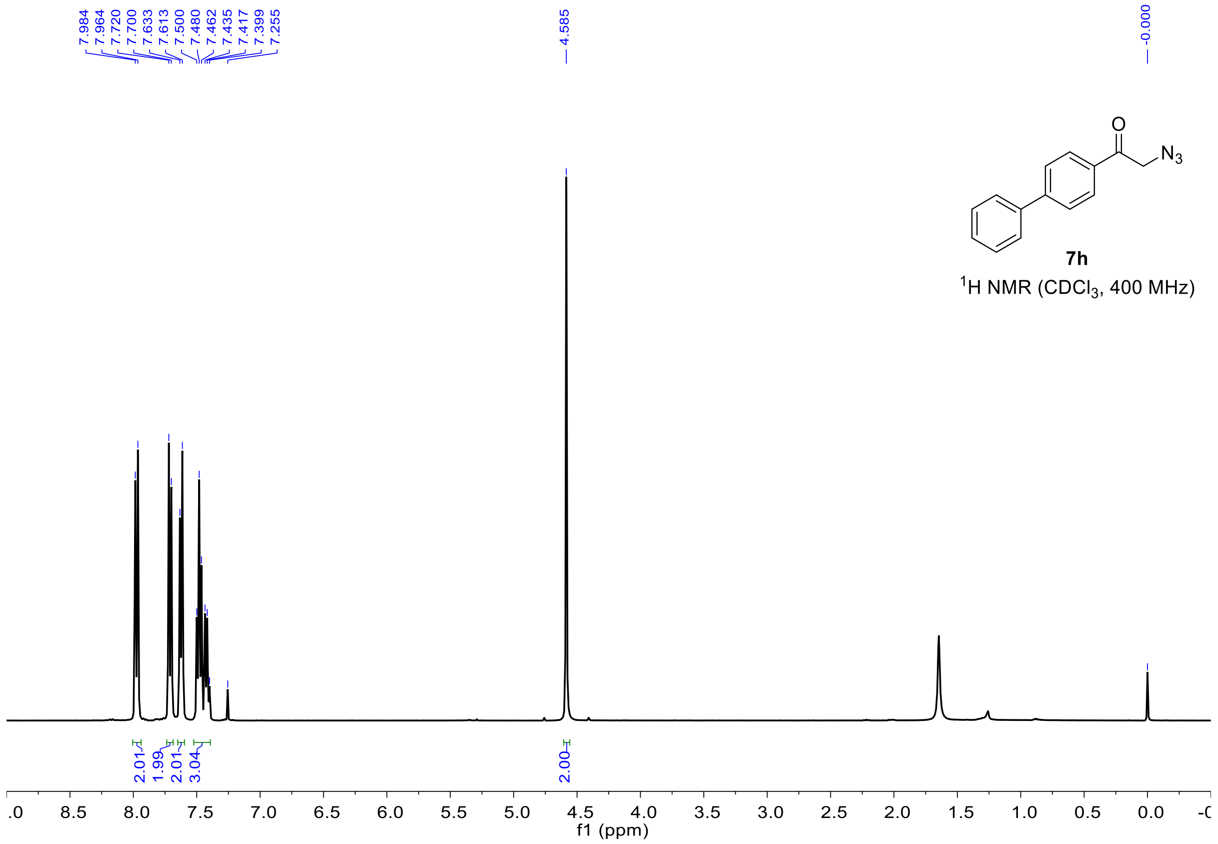
7.984
7.964
7.720
7.700
7.633
7.613
7.500
7.480
7.462
7.435
7.417
7.399
7.255
4.585
-0.000
O
N3
7h
1H NMR (CDCl3, 400 MHz)
2.01
1.99
2.01
3.04
2.00
f1 (ppm)

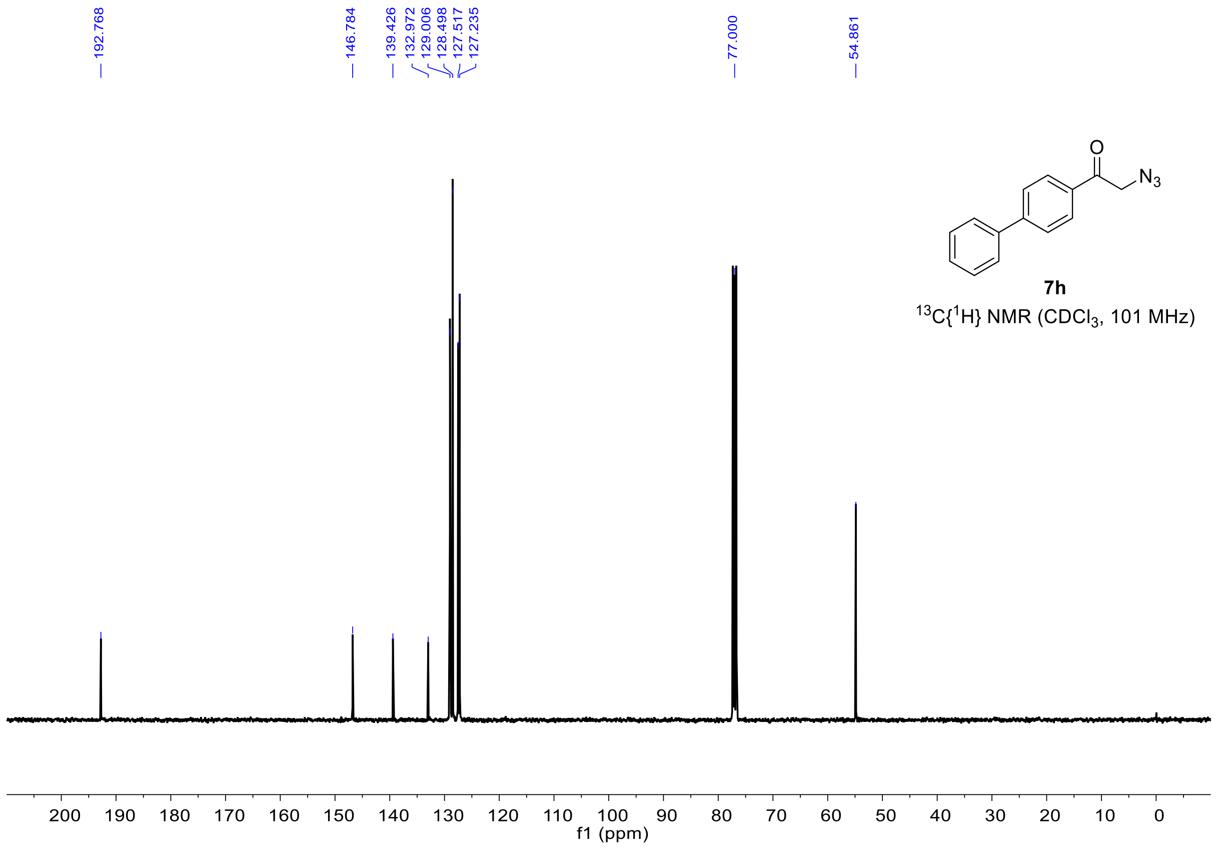
192.768
146.784
139.426
132.972
129.006
128.498
127.517
127.235
77.000
54.861
O
N3
7h
13C{1H} NMR (CDCl3, 101 MHz)
f1 (ppm)

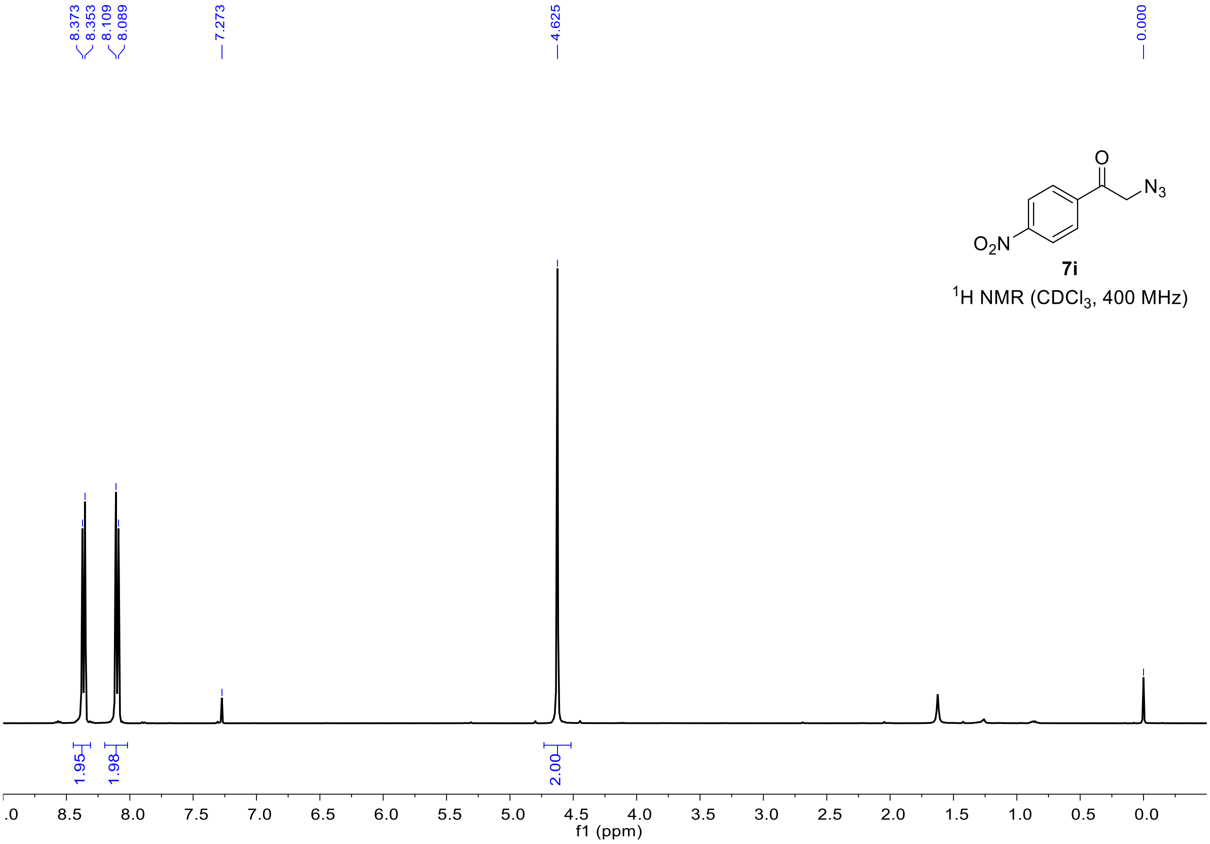
8.373
8.353
8.109
8.089
7.273
4.625
0.000
O
N3
O2N
7i
1H NMR (CDCl3, 400 MHz)
1.95
1.98
2.00
f1 (ppm)

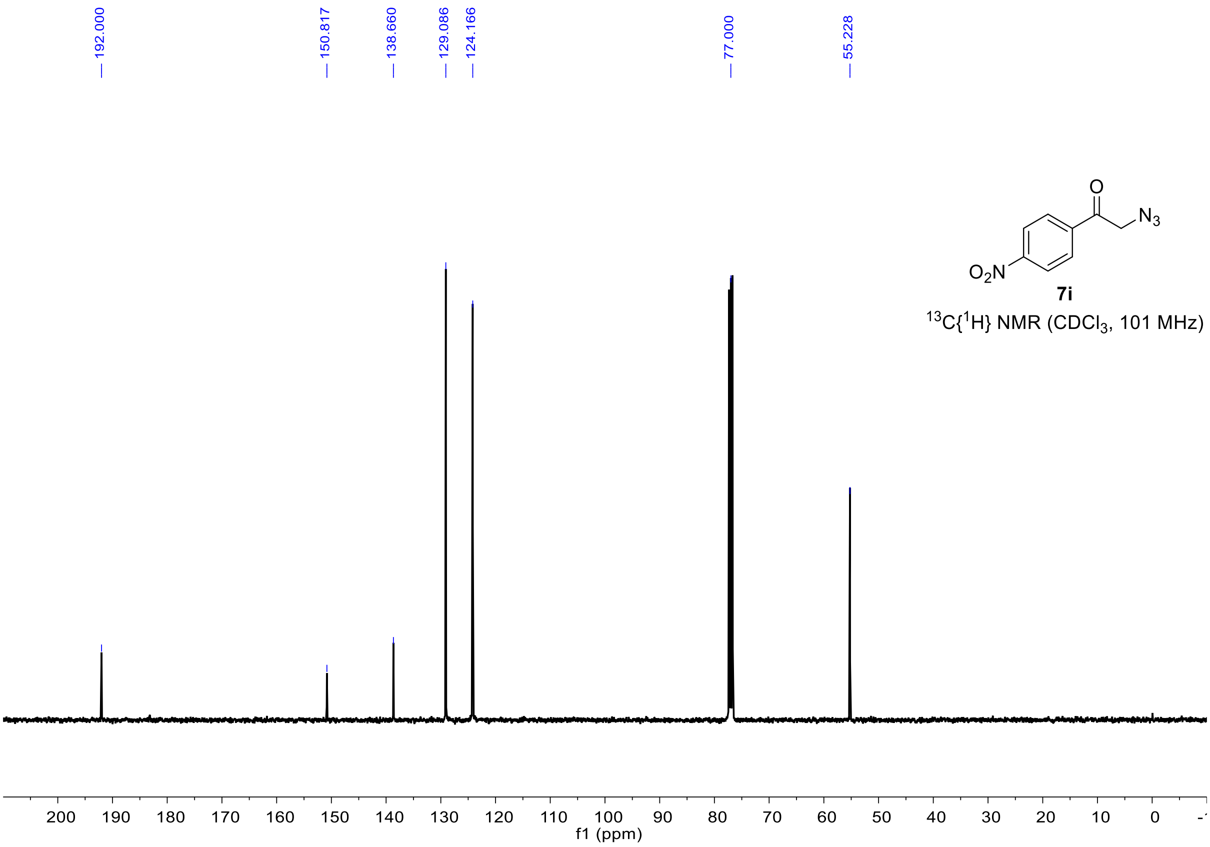
192.000
150.817
138.660
129.086
124.166
77.000
55.228
O
N3
O2N
7i
13C{1H} NMR (CDCl3, 101 MHz)
f1 (ppm)

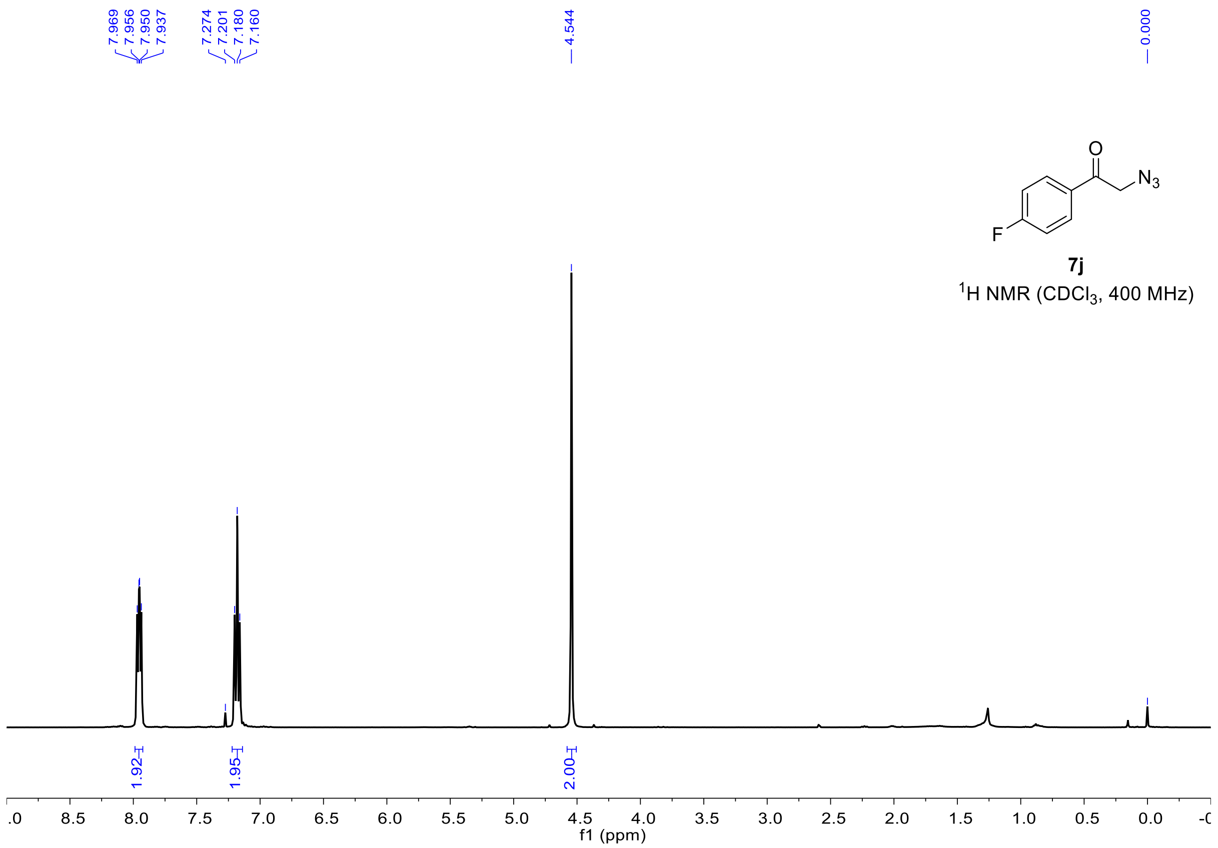
7.969
7.956
7.950
7.937
7.274
7.201
7.180
7.160
4.544
0.000
O
N3
F
7j
1H NMR (CDCl3, 400 MHz)
1.92
1.95
2.00
f1 (ppm)

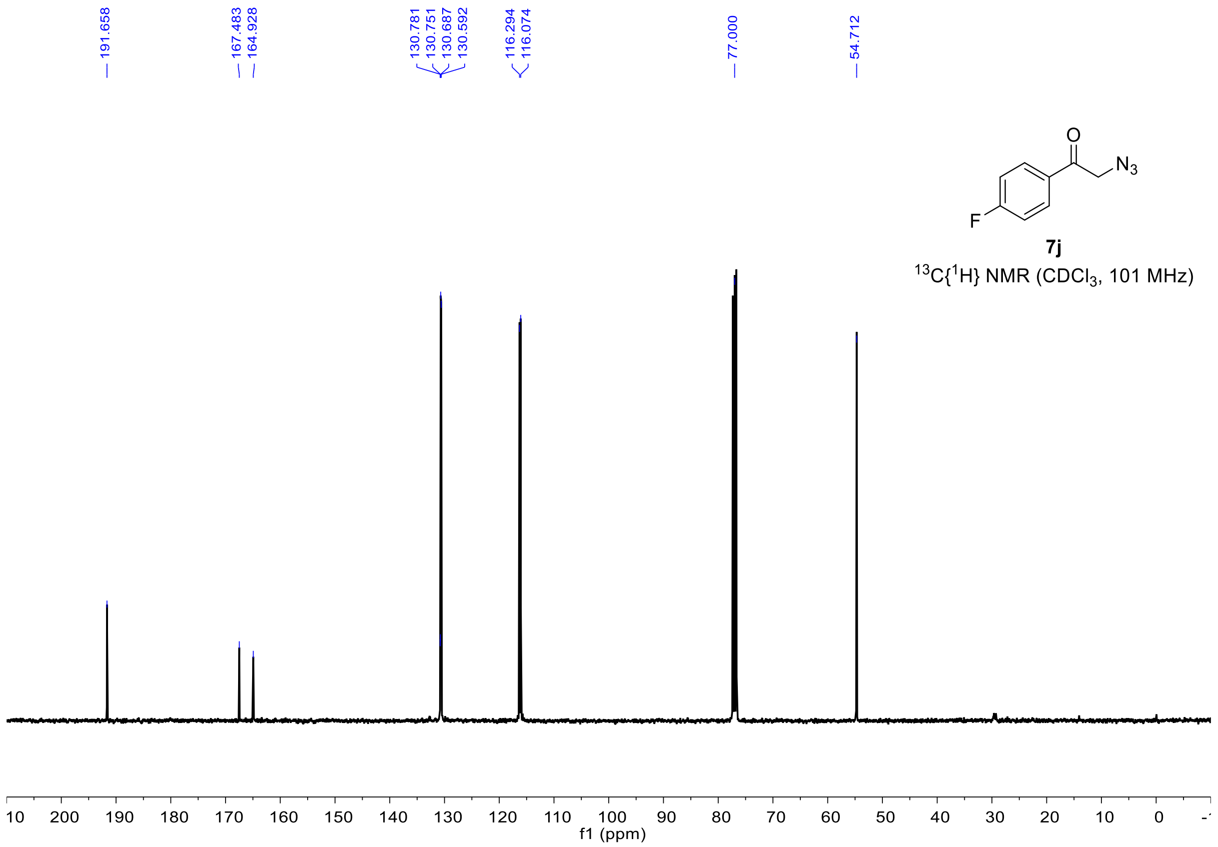
191.658
167.483
164.928
130.781
130.751
130.687
130.592
116.294
116.074
77.000
54.712
O
N3
F
7j
13C{1H} NMR (CDCl3, 101 MHz)
f1 (ppm)

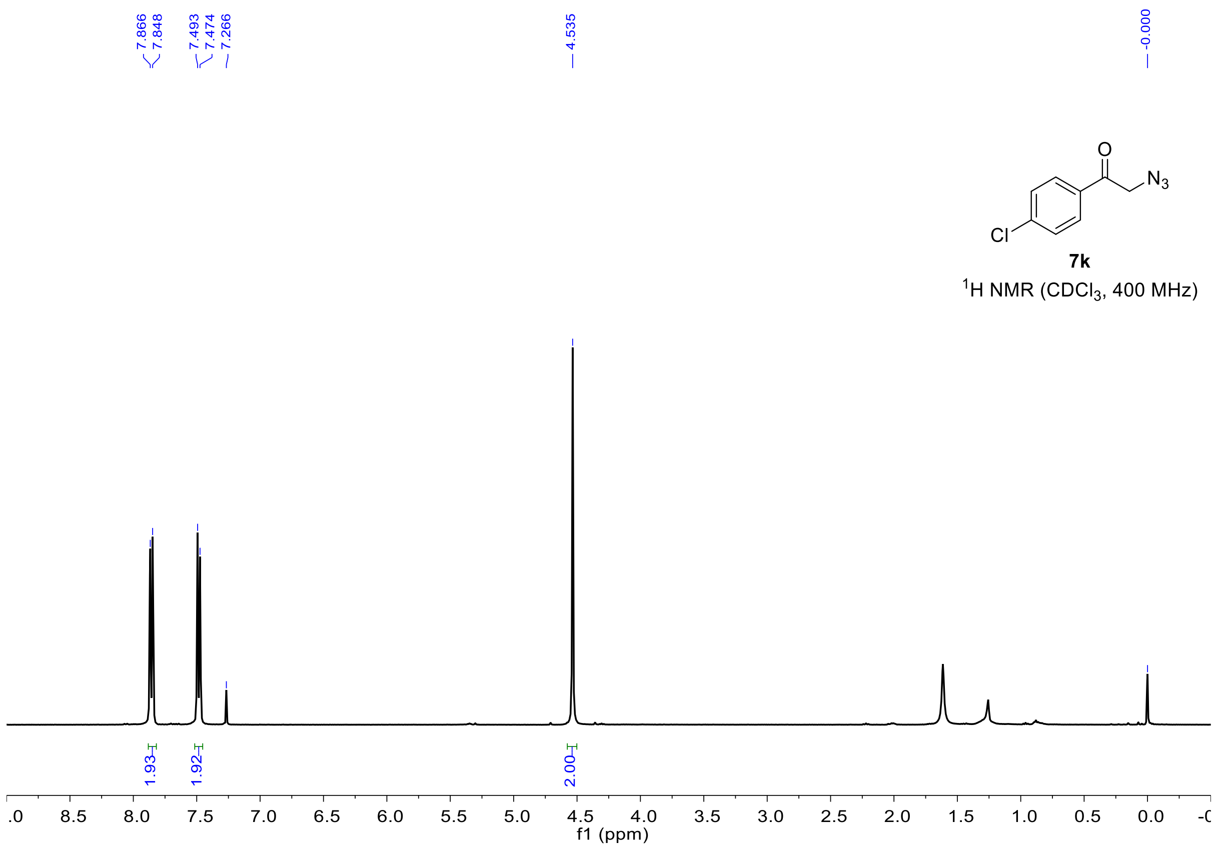
7.866
7.848
7.493
7.474
7.266
4.535
-0.000
O
N3
Cl
7k
1H NMR (CDCl3, 400 MHz)
1.93
1.92
2.00
f1 (ppm)

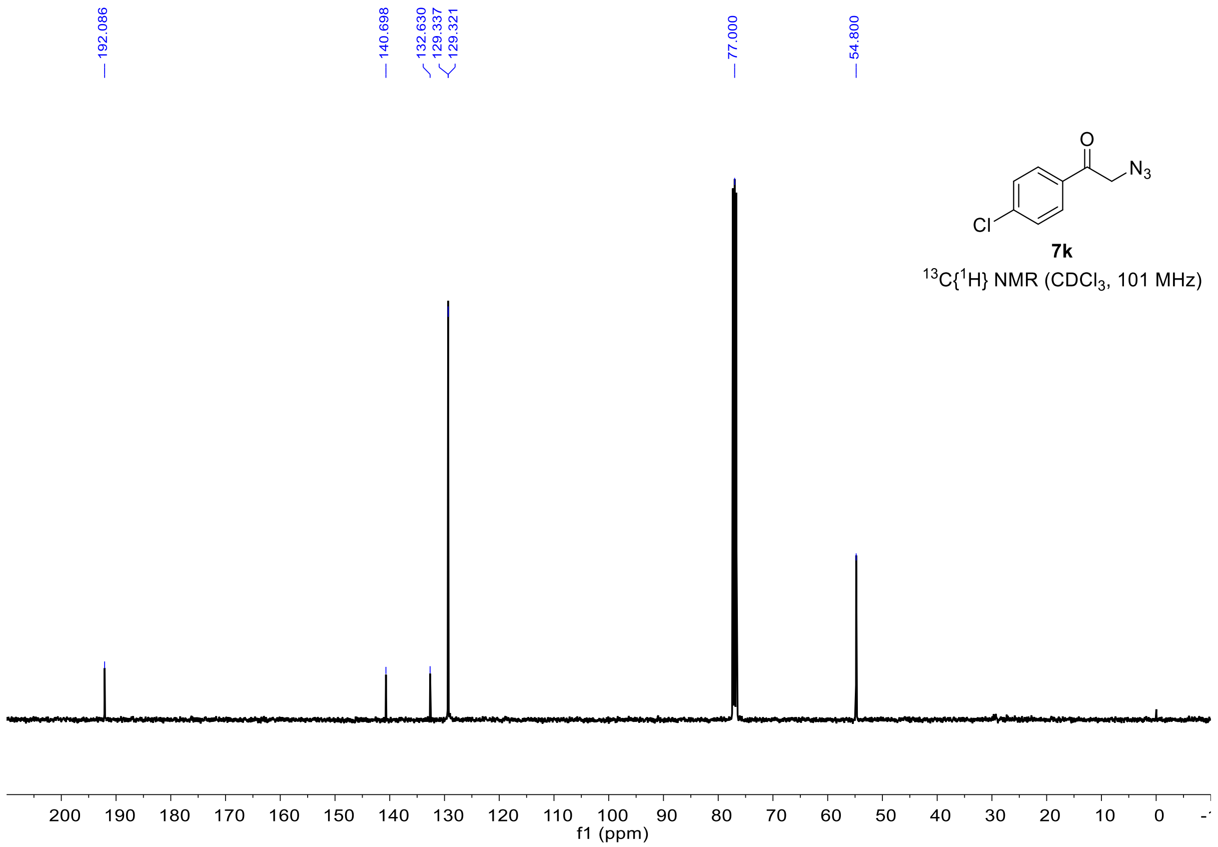
192.086
140.698
132.630
129.337
129.321
77.000
54.800
O
N3
Cl
7k
13C{1H} NMR (CDCl3, 101 MHz)
f1 (ppm)

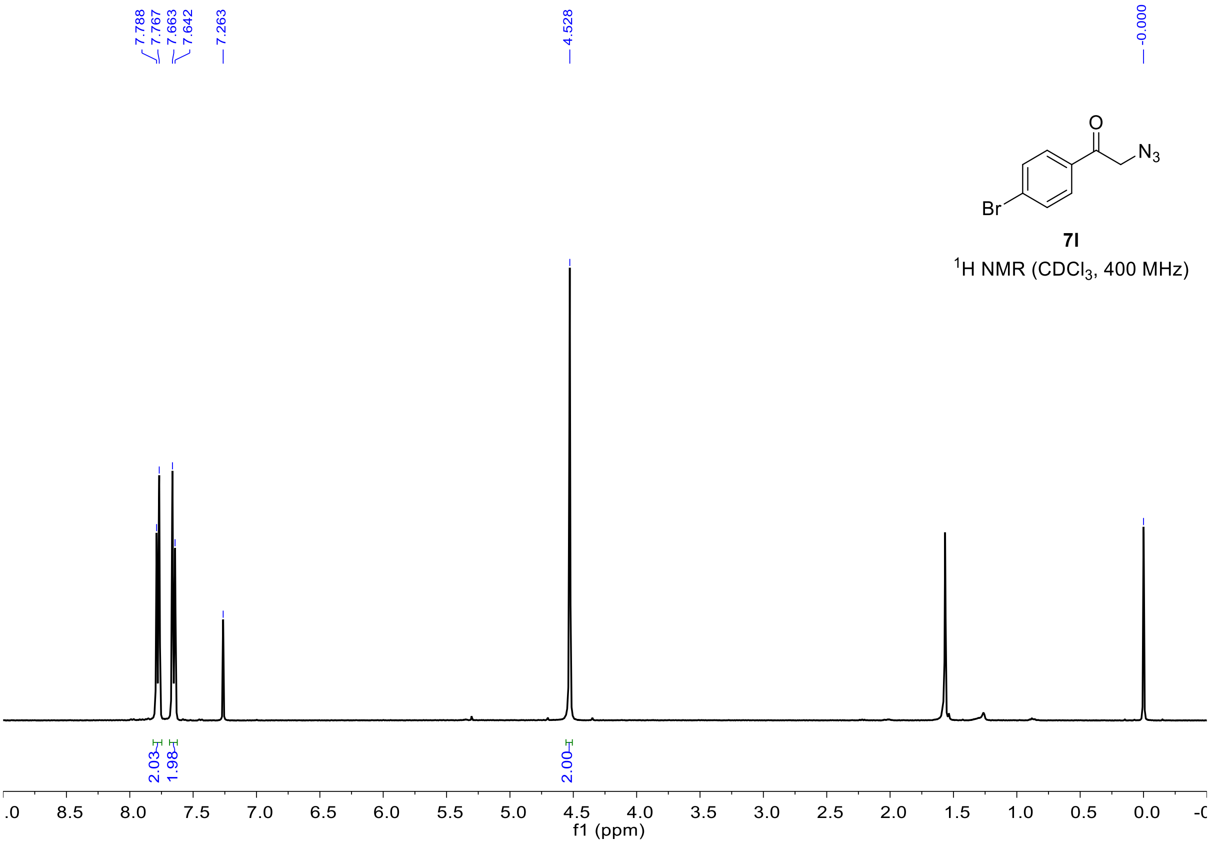
7.788
7.767
7.663
7.642
7.263
4.528
-0.000
O
N3
Br
7l
1H NMR (CDCl3, 400 MHz)
2.03
1.98
2.00
f1 (ppm)

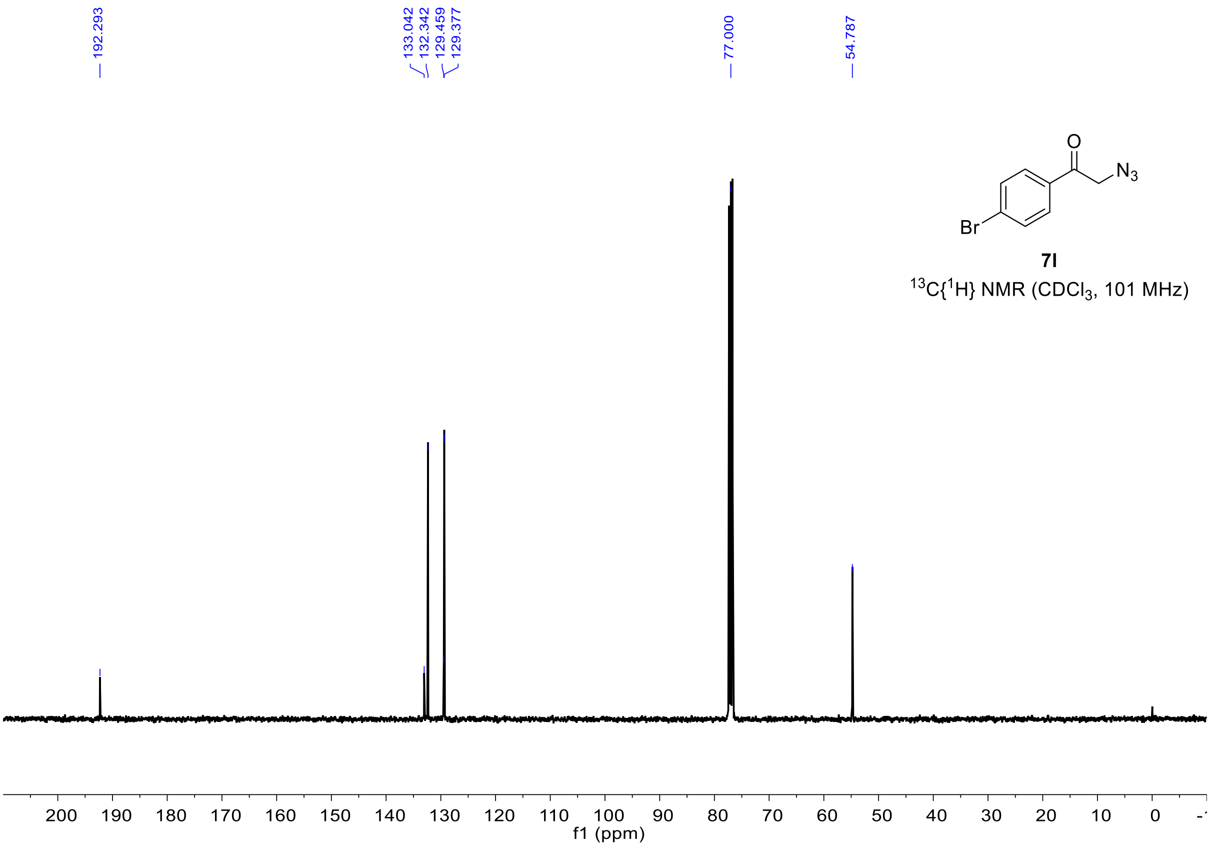
192.293
133.042
132.342
129.459
129.377
77.000
54.787
O
N3
Br
7l
13C{1H} NMR (CDCl3, 101 MHz)
f1 (ppm)

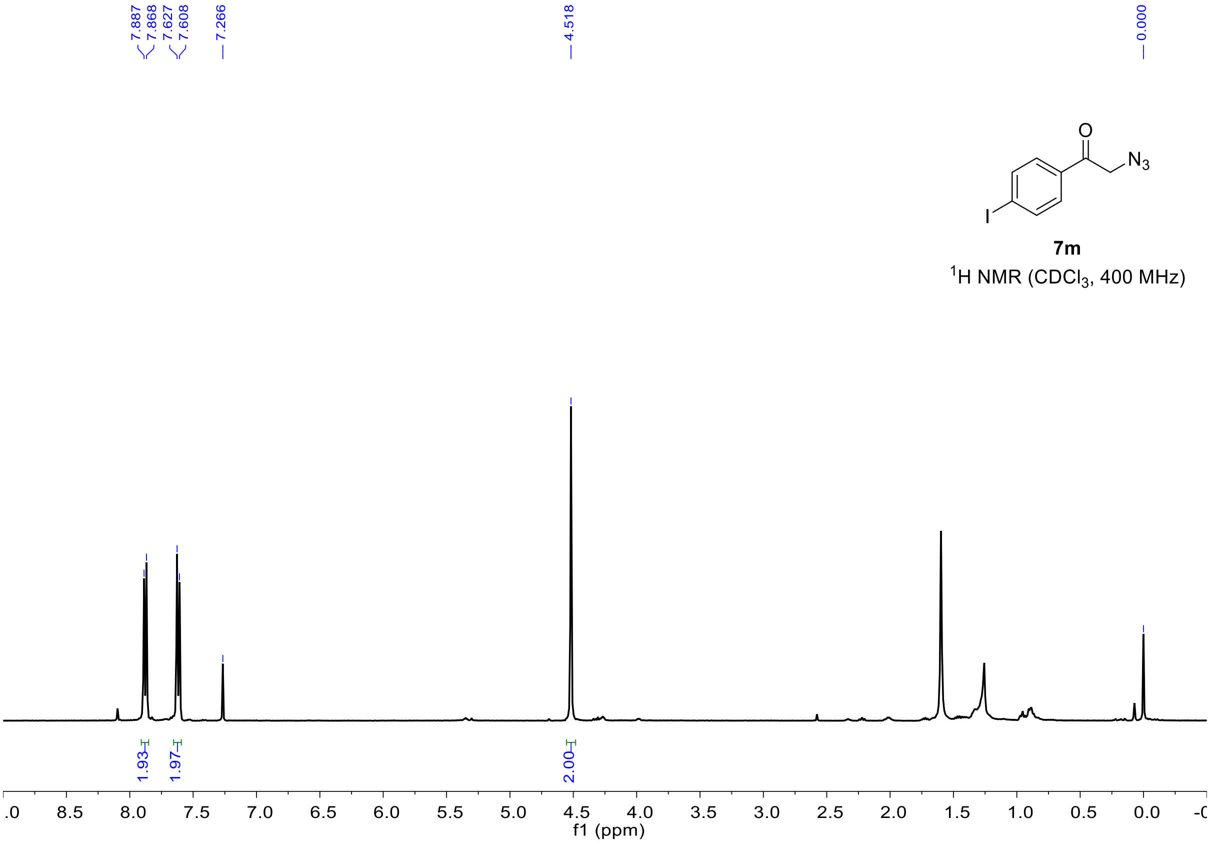
7.887
7.868
7.627
7.608
7.266
4.518
0.000
7m
^{1}H NMR ($CDCl_3$, 400 MHz)
1.93
1.97
2.00
f1 (ppm)

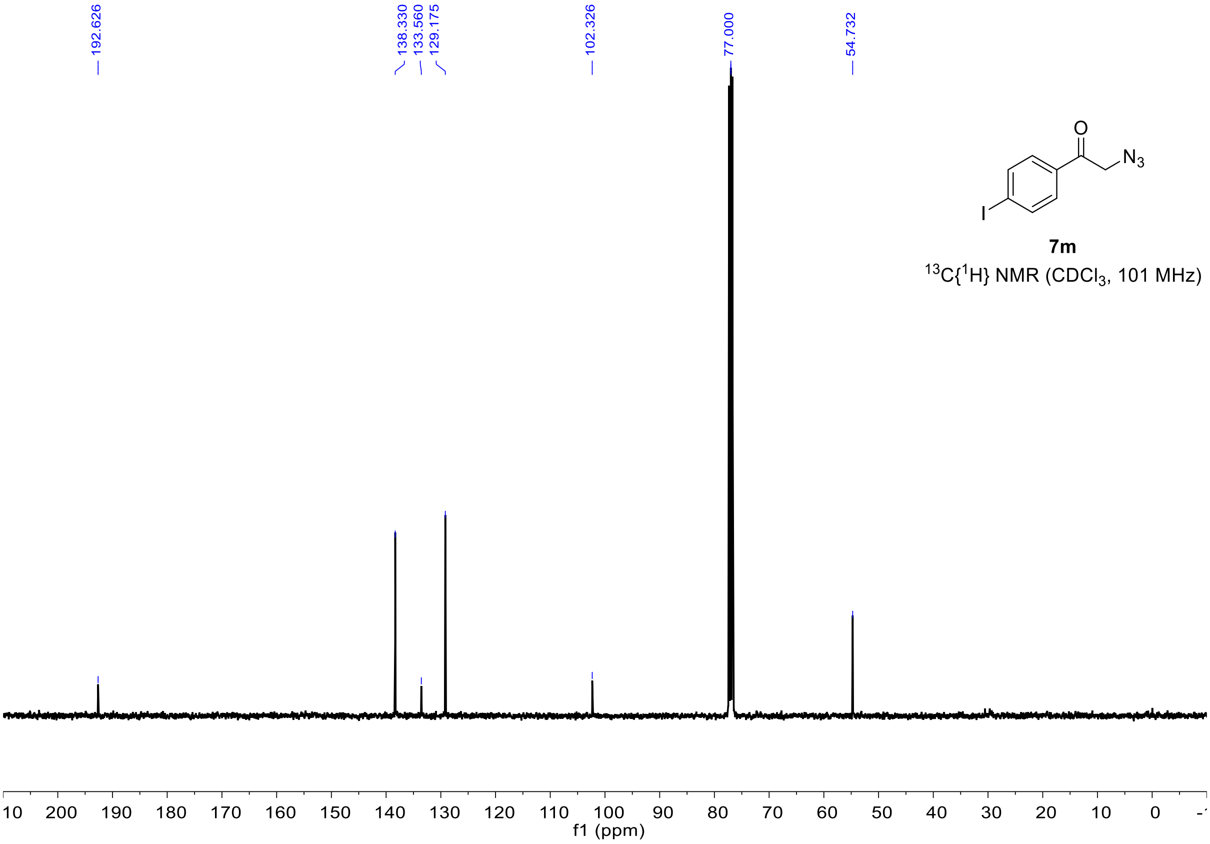
192.626
138.330
133.560
129.175
102.326
77.000
54.732
7m
^{13}C{^{1}H} NMR ($CDCl_3$, 101 MHz)
f1 (ppm)

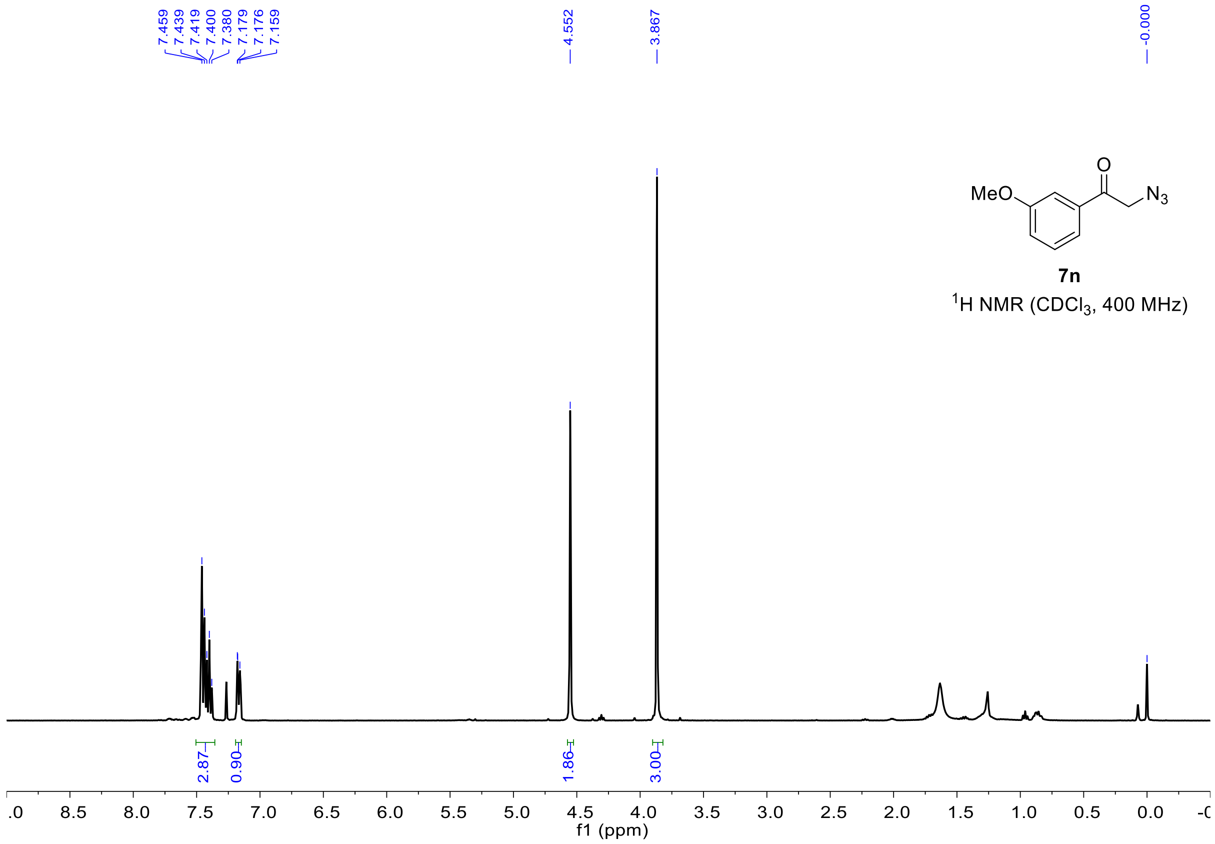
7.459
7.439
7.419
7.400
7.380
7.179
7.176
7.159
4.552
3.867
-0.000
MeO
O
N3
7n
1H NMR (CDCl3, 400 MHz)
2.87
0.90
1.86
3.00
f1 (ppm)

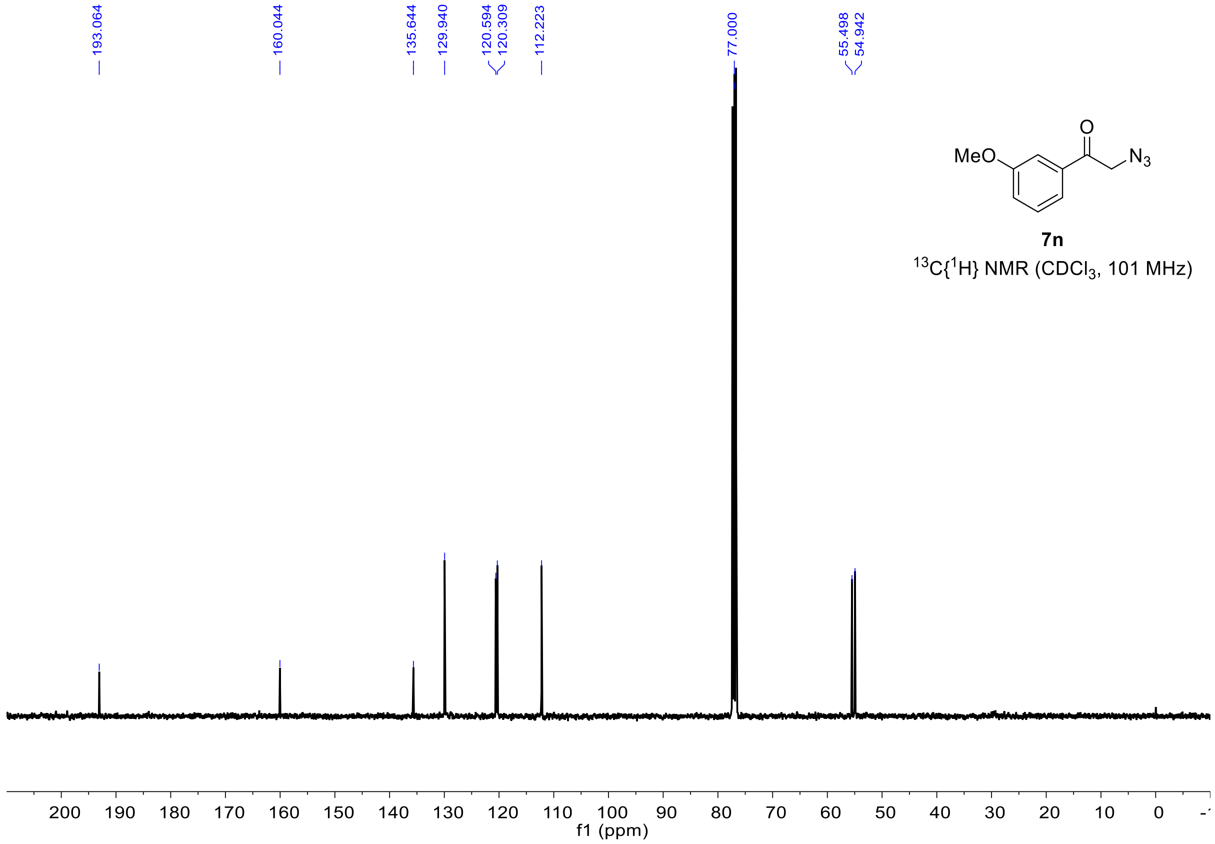
193.064
160.044
135.644
129.940
120.594
120.309
112.223
77.000
55.498
54.942
MeO
O
N3
7n
13C{1H} NMR (CDCl3, 101 MHz)
f1 (ppm)

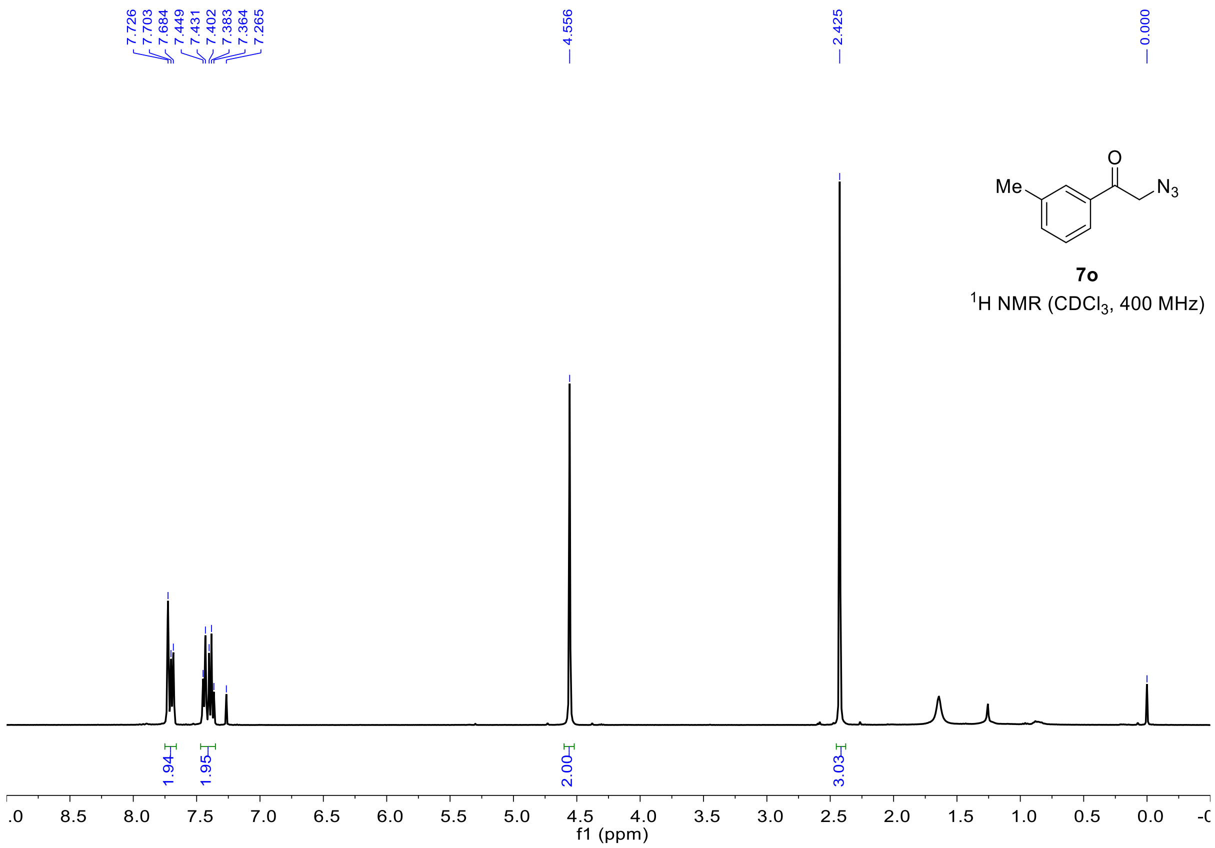
7.726
7.703
7.684
7.449
7.431
7.402
7.383
7.364
7.265
4.556
2.425
0.000
Me
O
N3
7o
1H NMR (CDCl3, 400 MHz)
1.94
1.95
2.00
3.03
f1 (ppm)

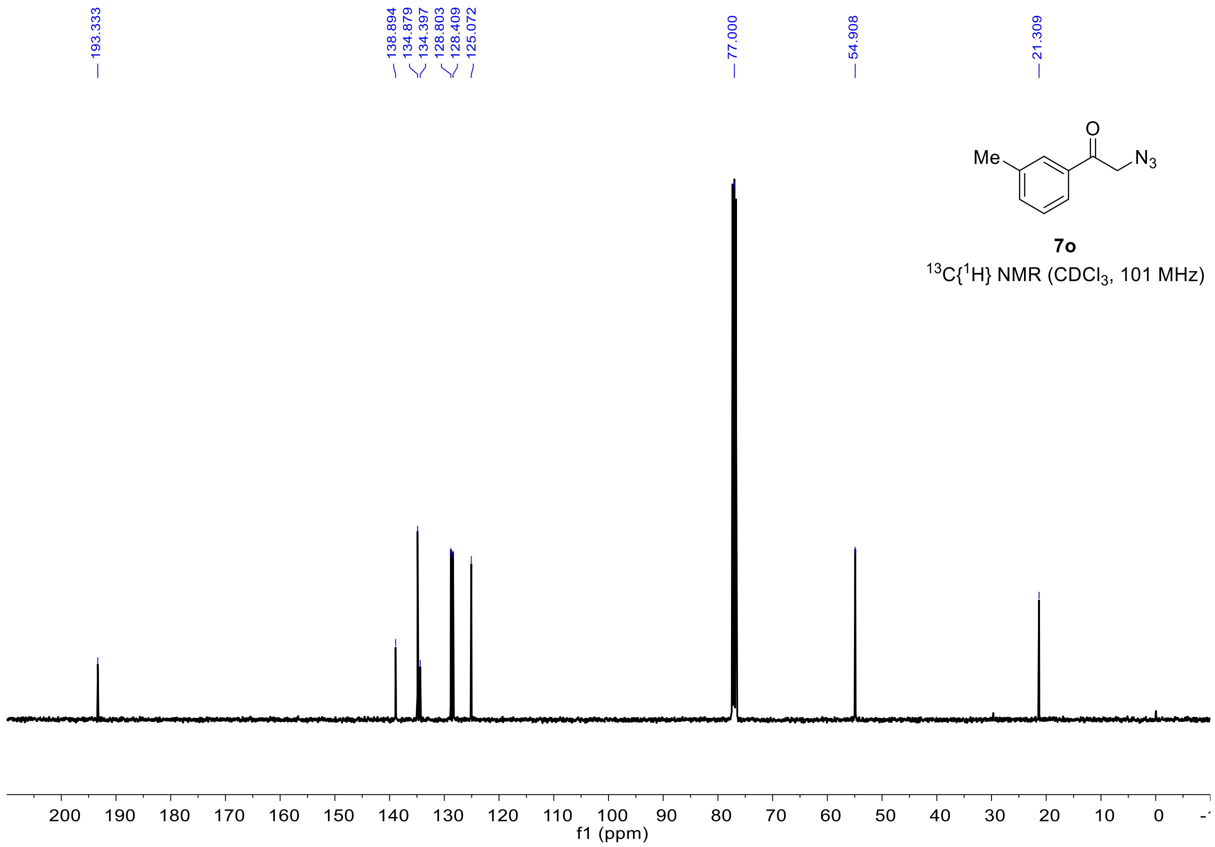
193.333
138.894
134.879
134.397
128.803
128.409
125.072
77.000
54.908
21.309
Me
O
N3
7o
13C{1H} NMR (CDCl3, 101 MHz)
f1 (ppm)

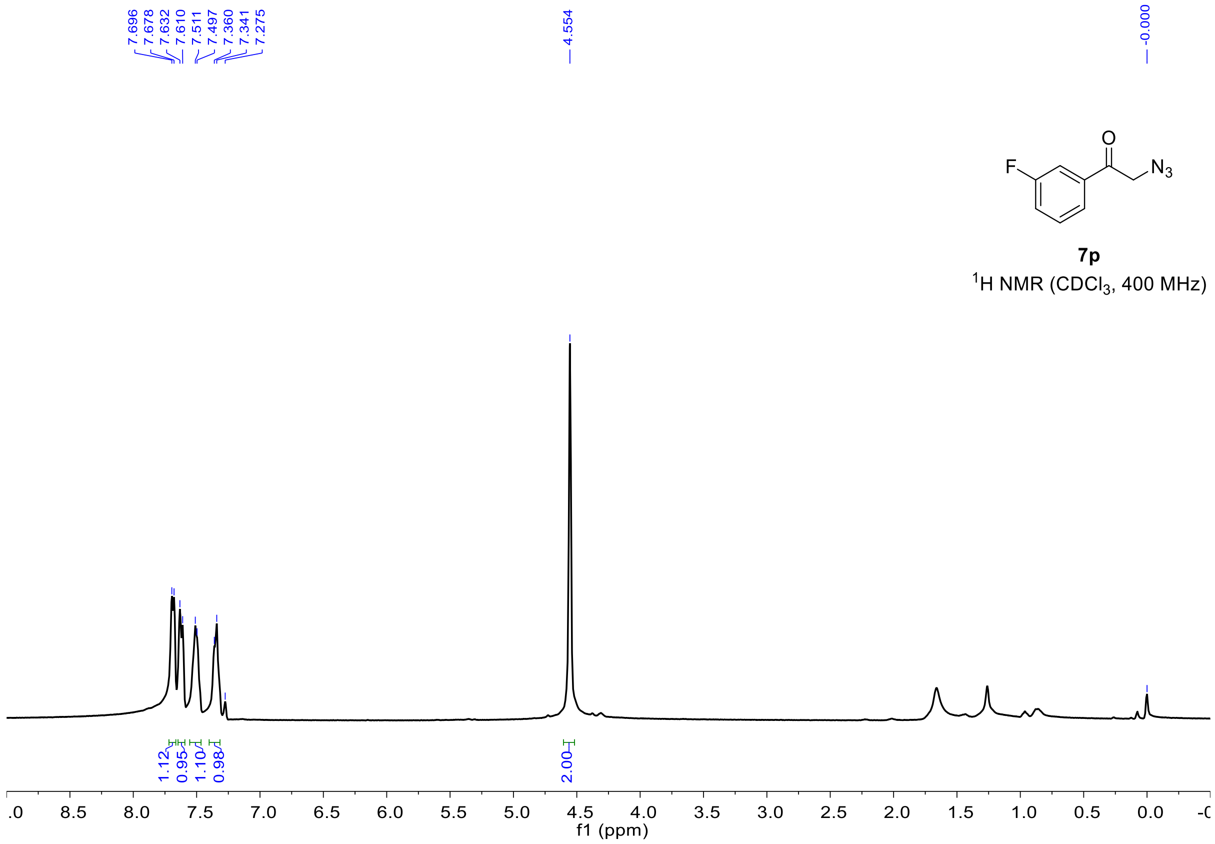
7.696
7.678
7.632
7.610
7.511
7.497
7.360
7.341
7.275
4.554
-0.000
F
O
N3
7p
1H NMR (CDCl3, 400 MHz)
1.12
0.95
1.10
0.98
2.00
f1 (ppm)

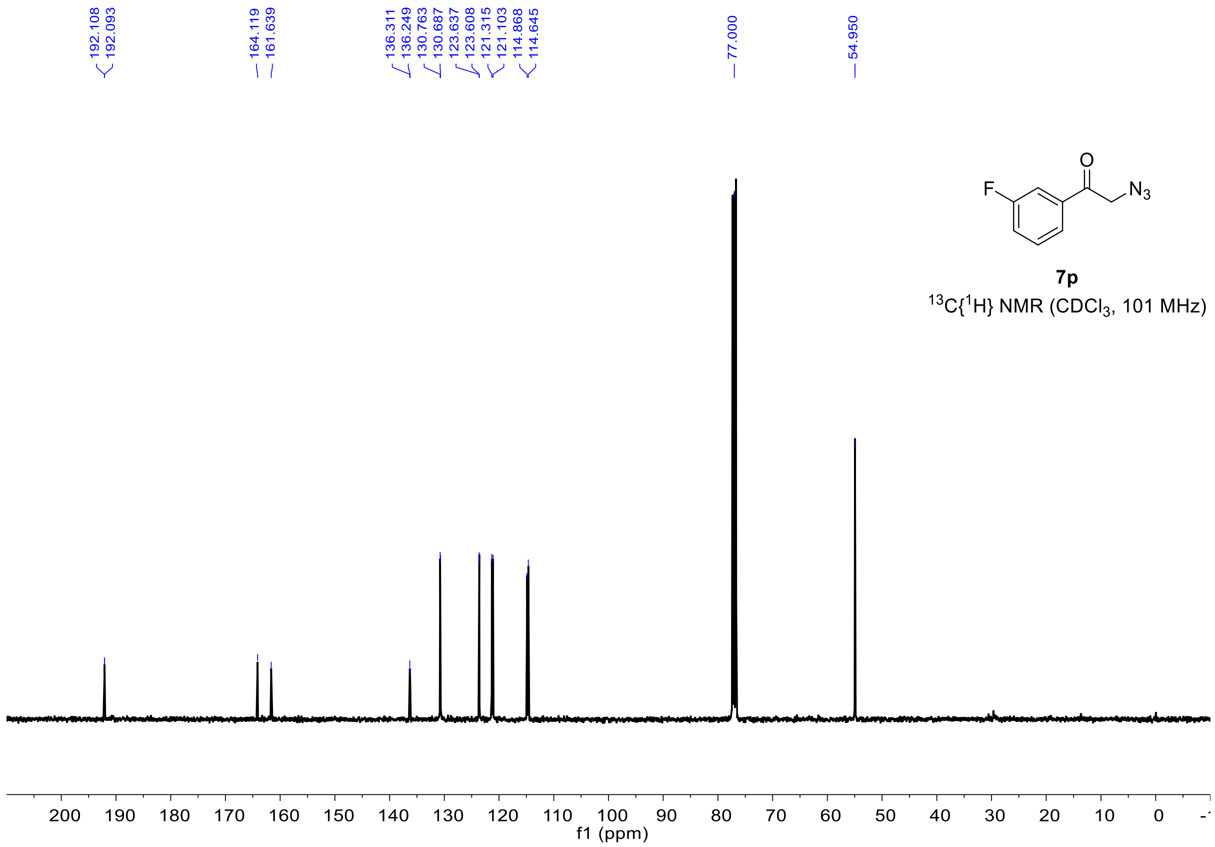
192.108
192.093
164.119
161.639
136.311
136.249
130.763
130.687
123.637
123.608
121.315
121.103
114.868
114.645
77.000
54.950
F
O
N3
7p
13C{1H} NMR (CDCl3, 101 MHz)
f1 (ppm)

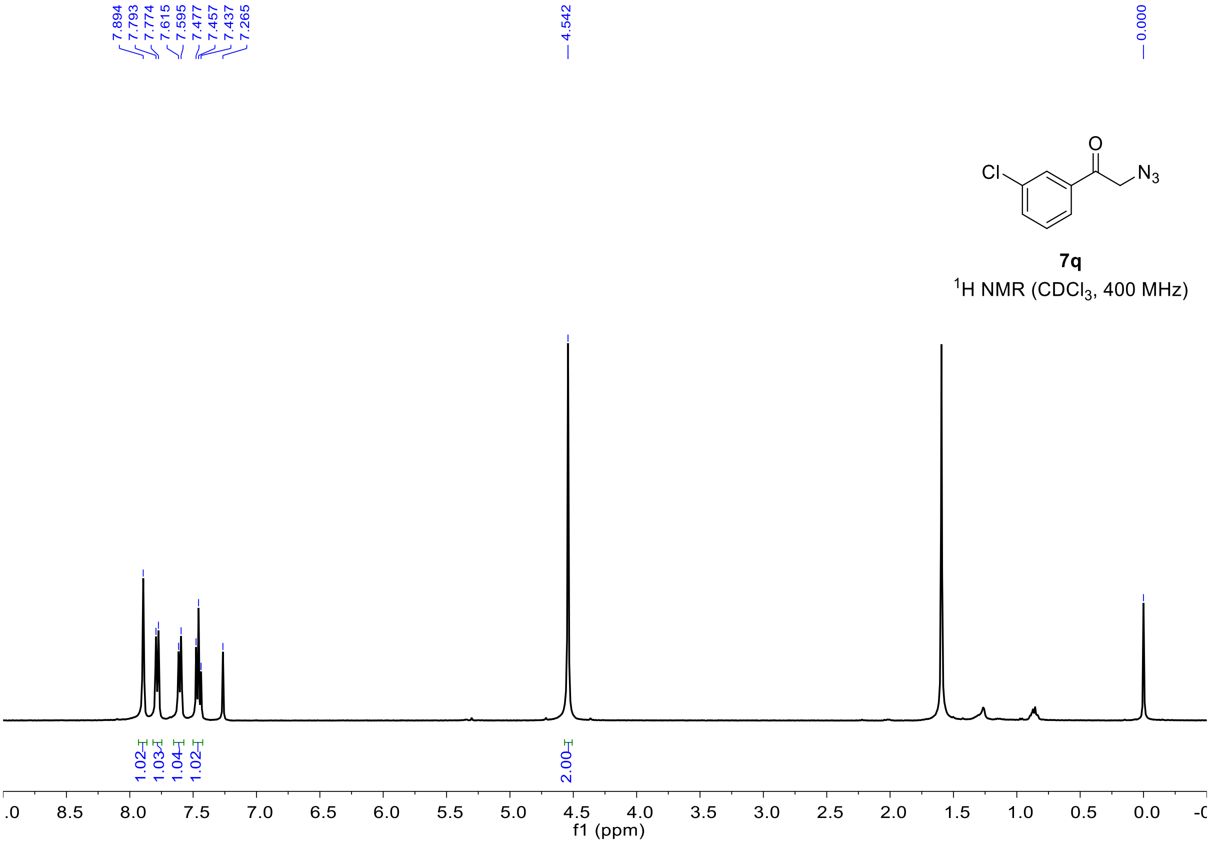
7.894
7.793
7.774
7.615
7.595
7.477
7.457
7.437
7.265
4.542
0.000
O
Cl
N3
7q
1H NMR (CDCl3, 400 MHz)
1.02
1.03
1.04
1.02
2.00
f1 (ppm)

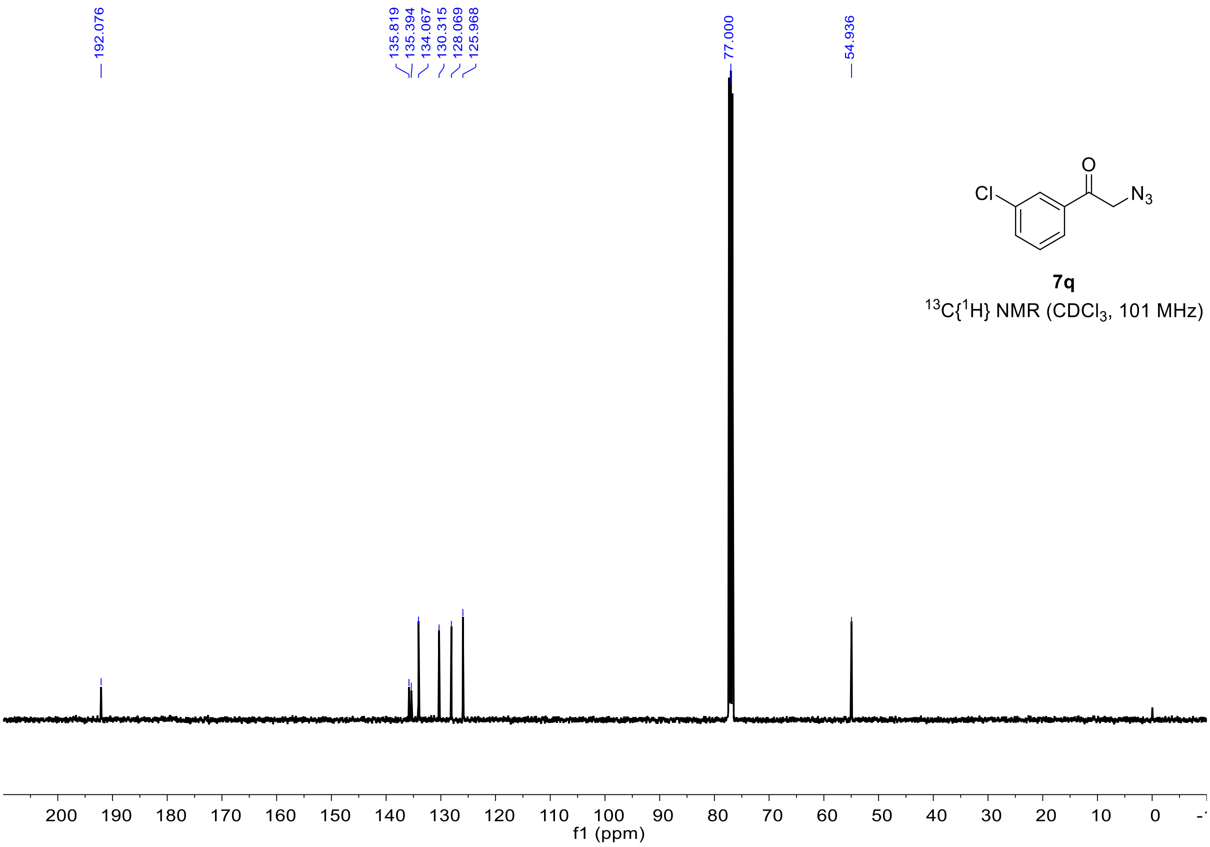
192.076
135.819
135.394
134.067
130.315
128.069
125.968
77.000
54.936
O
Cl
N3
7q
13C{1H} NMR (CDCl3, 101 MHz)
f1 (ppm)

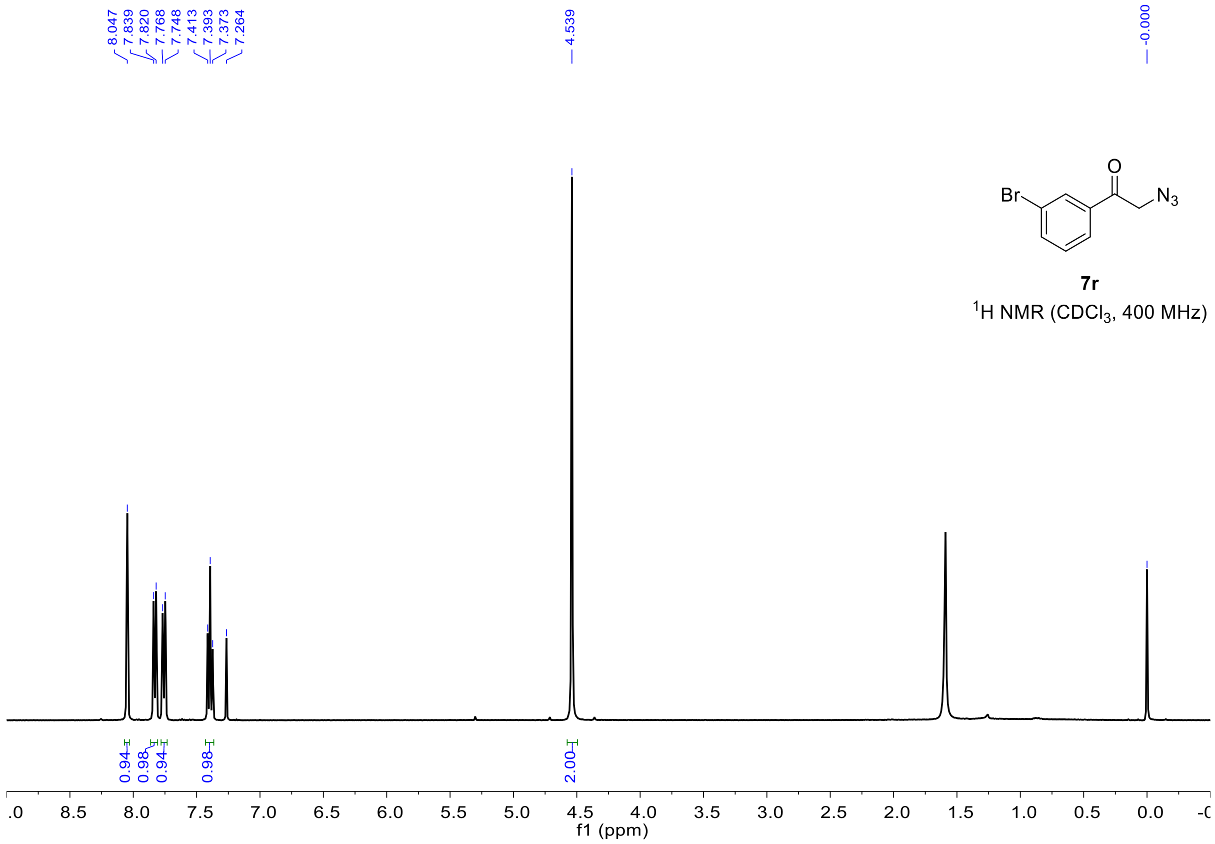
8.047
7.839
7.820
7.768
7.748
7.413
7.393
7.373
7.264
4.539
-0.000
Br
O
N3
7r
1H NMR (CDCl3, 400 MHz)
0.94
0.98
0.94
0.98
2.00
f1 (ppm)

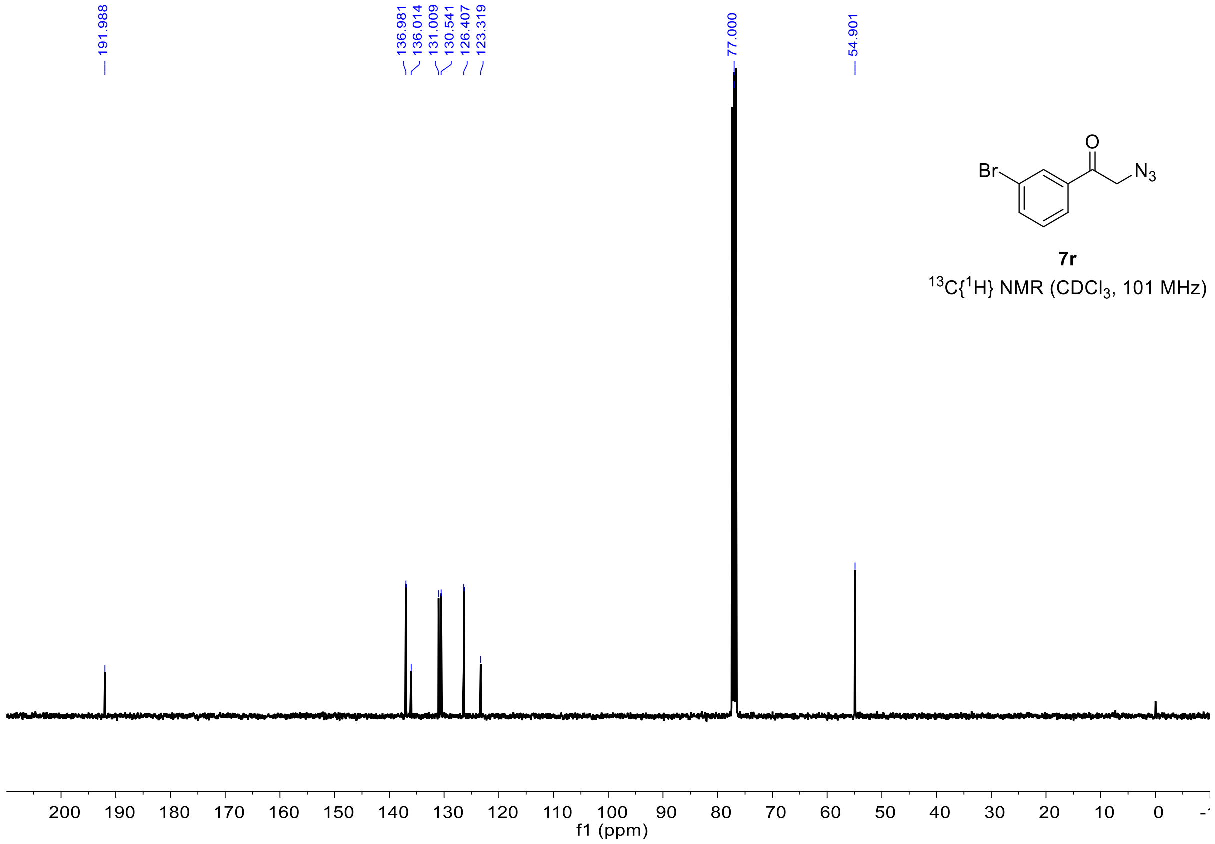
191.988
136.981
136.014
131.009
130.541
126.407
123.319
77.000
54.901
Br
O
N3
7r
13C{1H} NMR (CDCl3, 101 MHz)
f1 (ppm)

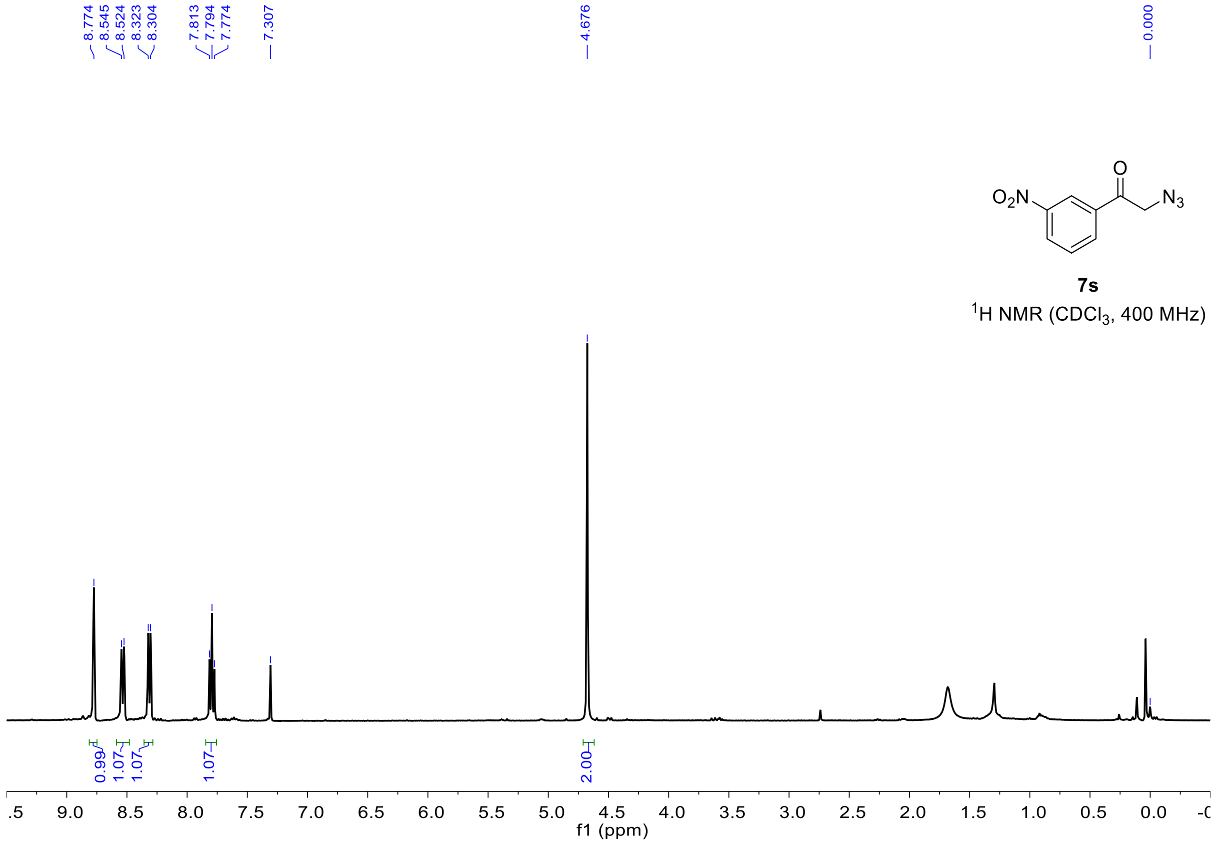
8.774
8.545
8.524
8.323
8.304
7.813
7.794
7.774
7.307
4.676
0.000
O2N
O
N3
7s
1H NMR (CDCl3, 400 MHz)
0.99
1.07
1.07
1.07
2.00
f1 (ppm)

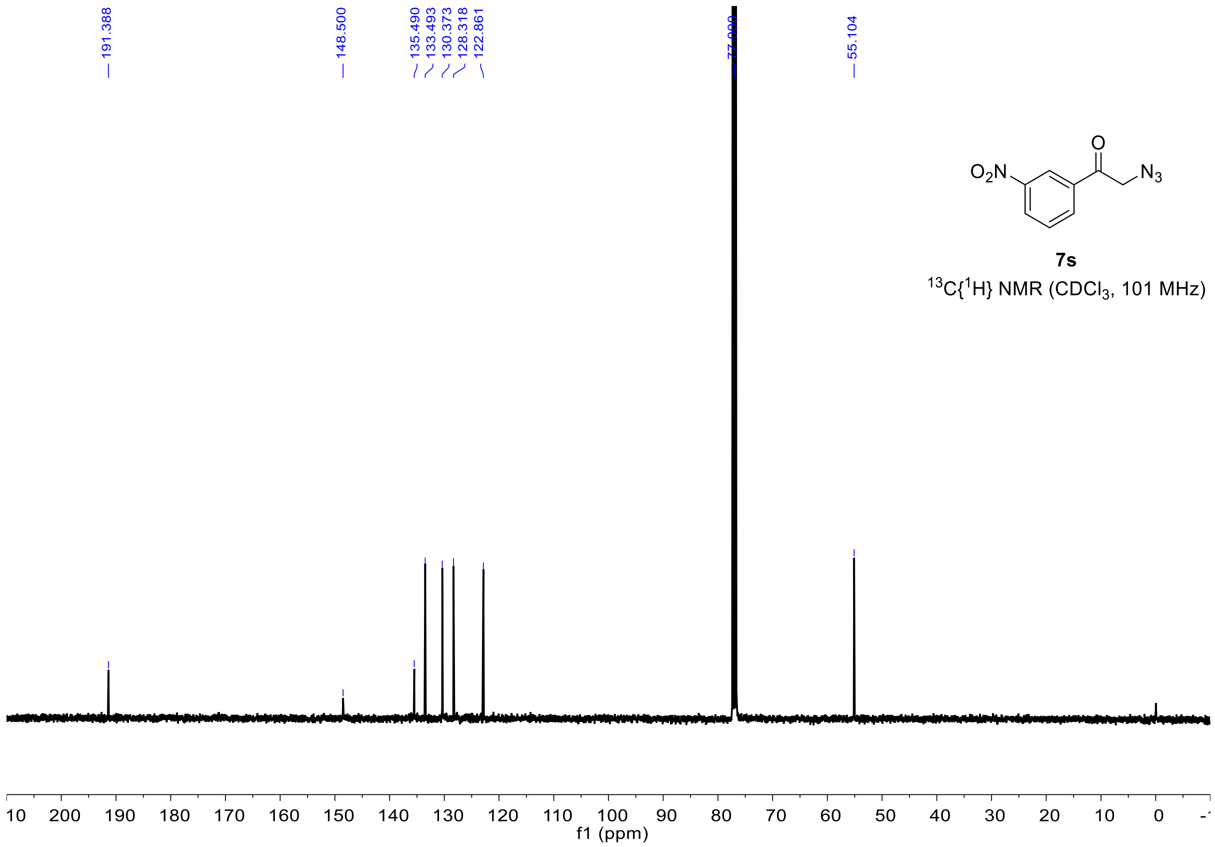
191.388
148.500
135.490
133.493
130.373
128.318
122.861
55.104
O2N
O
N3
7s
13C{1H} NMR (CDCl3, 101 MHz)
f1 (ppm)

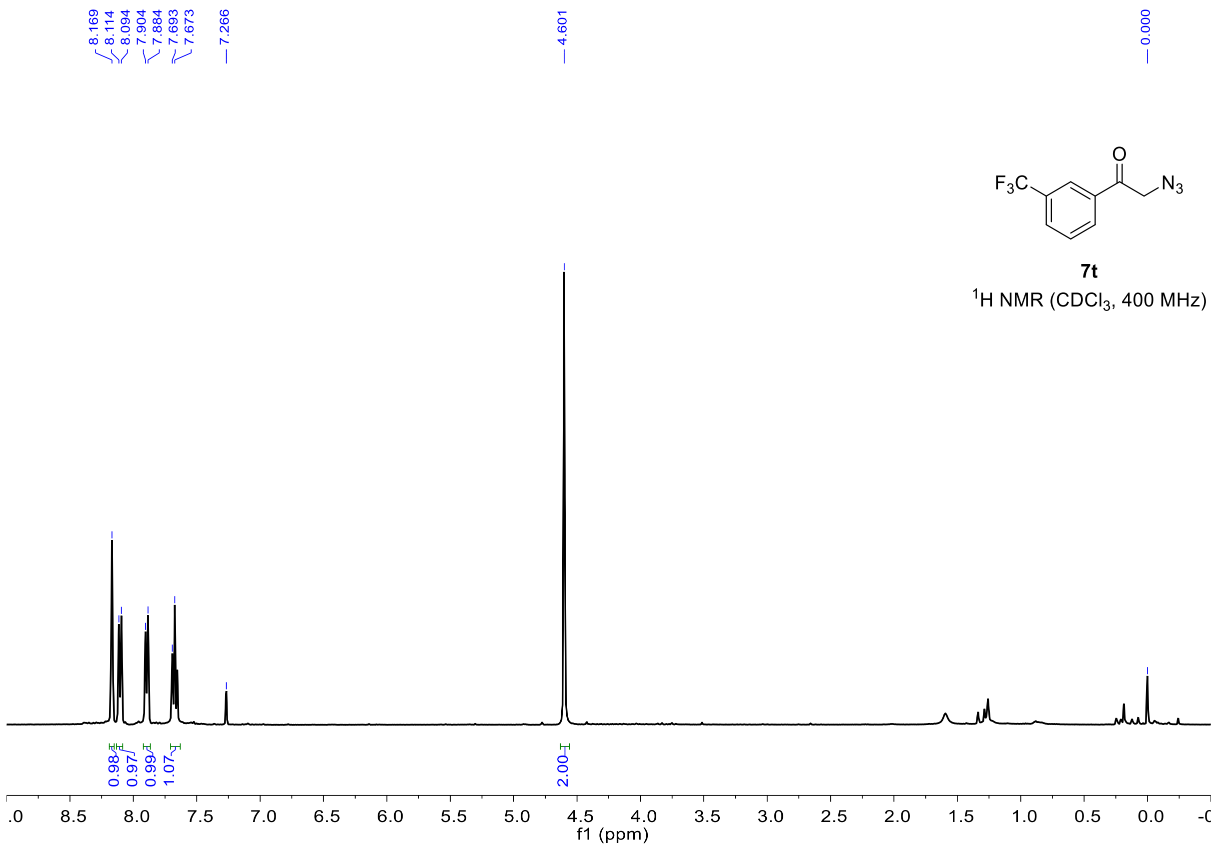
8.169
8.114
8.094
7.904
7.884
7.693
7.673
7.266
4.601
0.000
0.98
0.97
0.99
1.07
2.00
f1 (ppm)
F3C
O
N3
7t
1H NMR (CDCl3, 400 MHz)

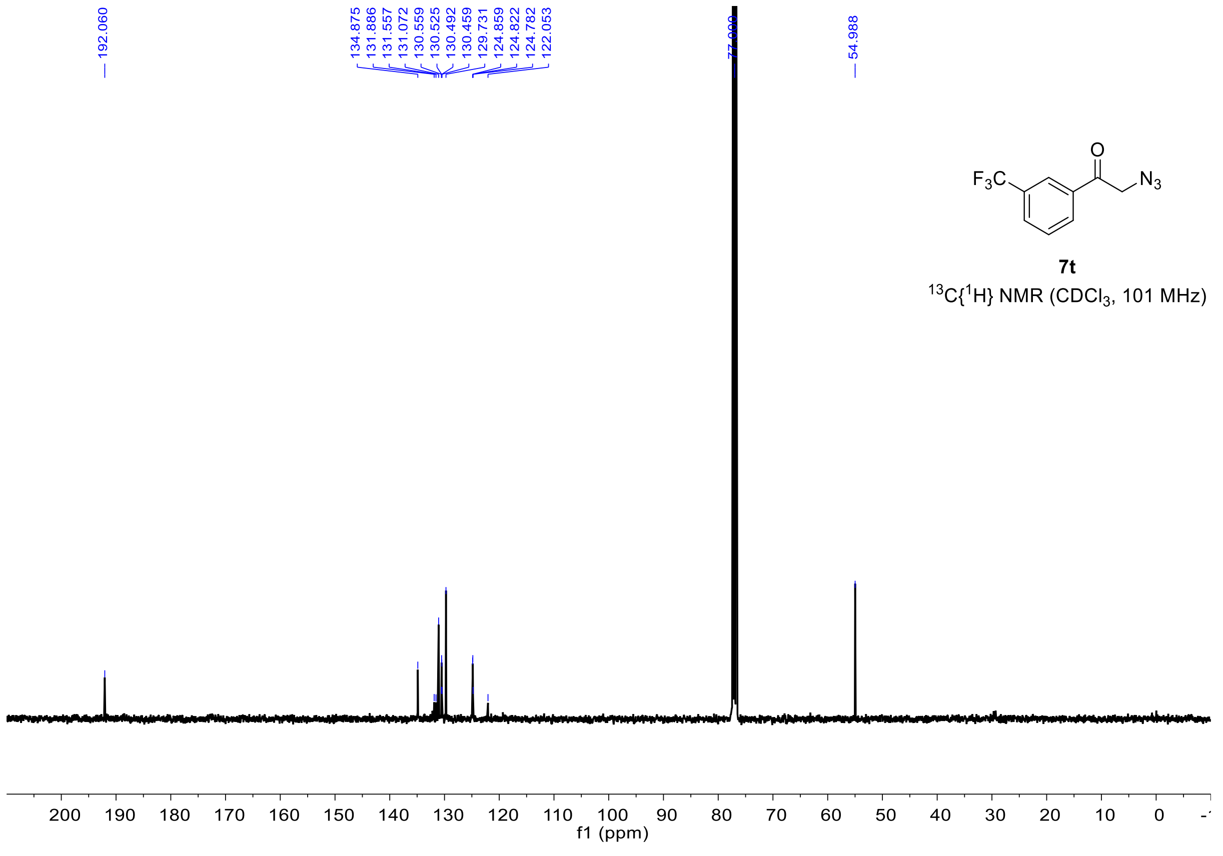
192.060
134.875
131.886
131.557
131.072
130.559
130.525
130.492
130.459
129.731
124.859
124.822
124.782
122.053
54.988
f1 (ppm)
F3C
O
N3
7t
13C{1H} NMR (CDCl3, 101 MHz)

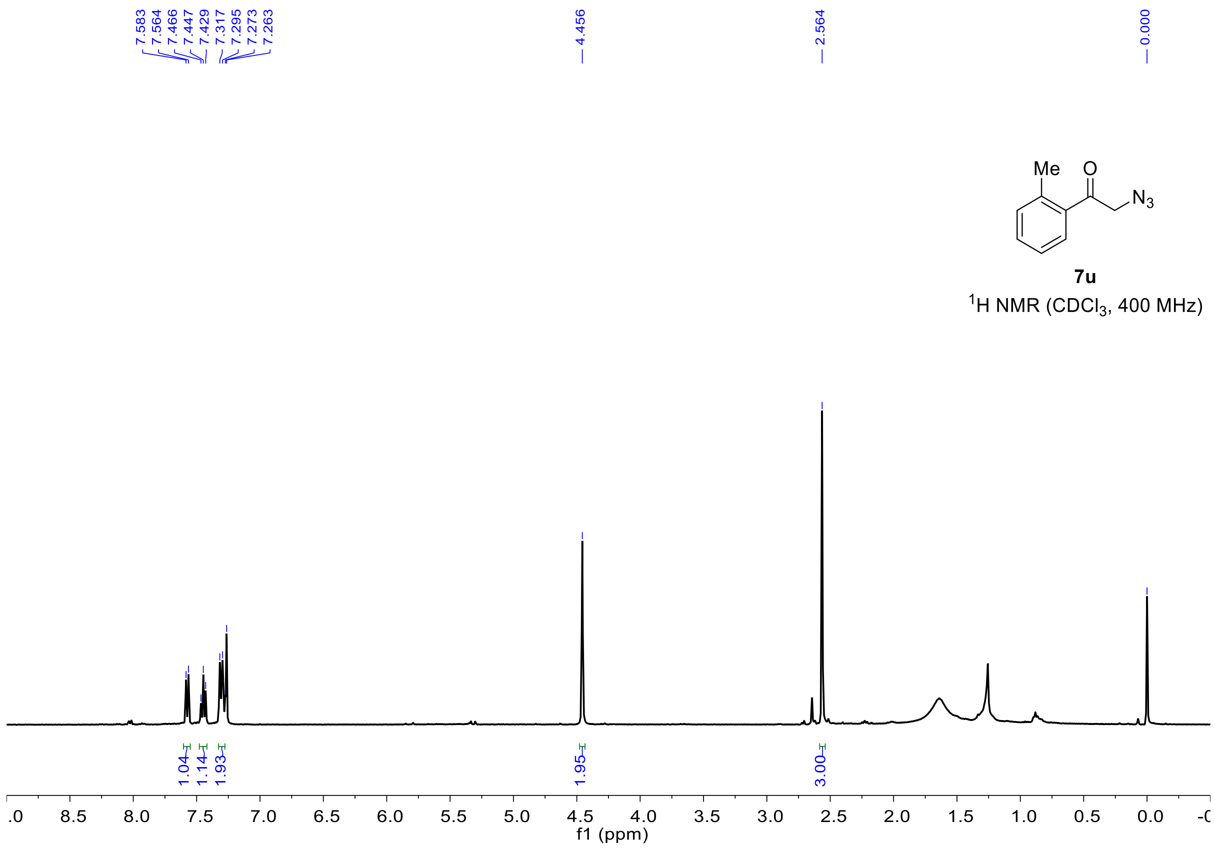
7.583
7.564
7.466
7.447
7.429
7.317
7.295
7.273
7.263
4.456
2.564
0.000
Me
O
N3
7u
1H NMR (CDCl3, 400 MHz)
1.04
1.14
1.93
1.95
3.00
f1 (ppm)

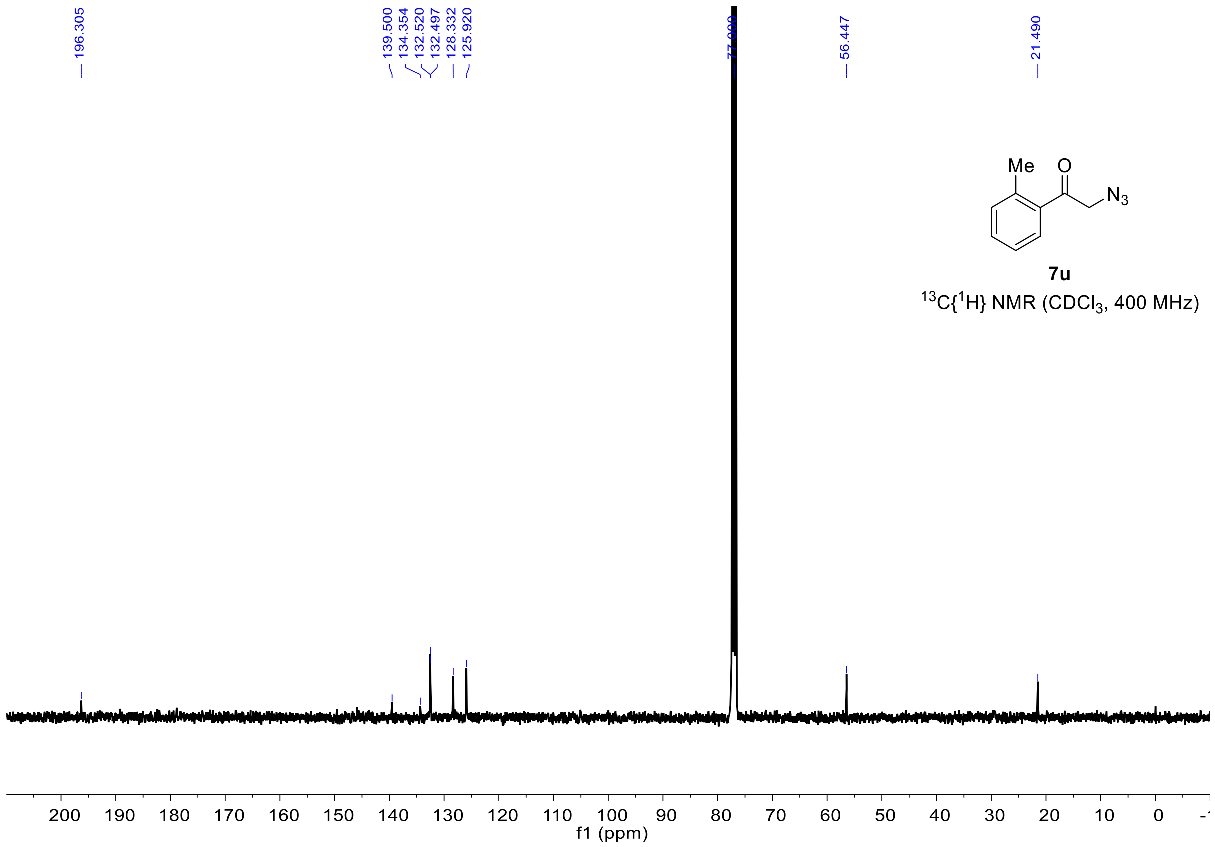
196.305
139.500
134.354
132.520
132.497
128.332
125.920
56.447
21.490
Me
O
N3
7u
13C{1H} NMR (CDCl3, 400 MHz)
f1 (ppm)

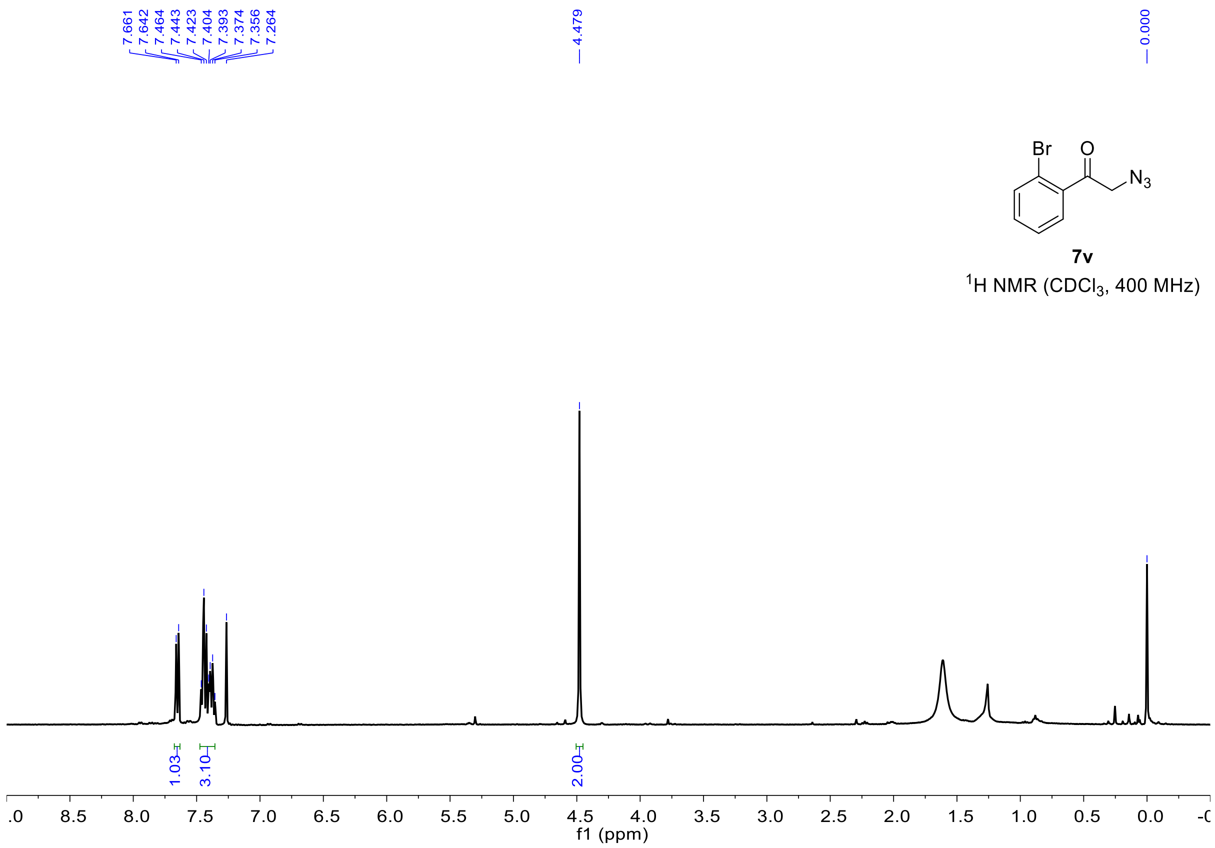

7v

^1H NMR ($CDCl_3$, 400 MHz)

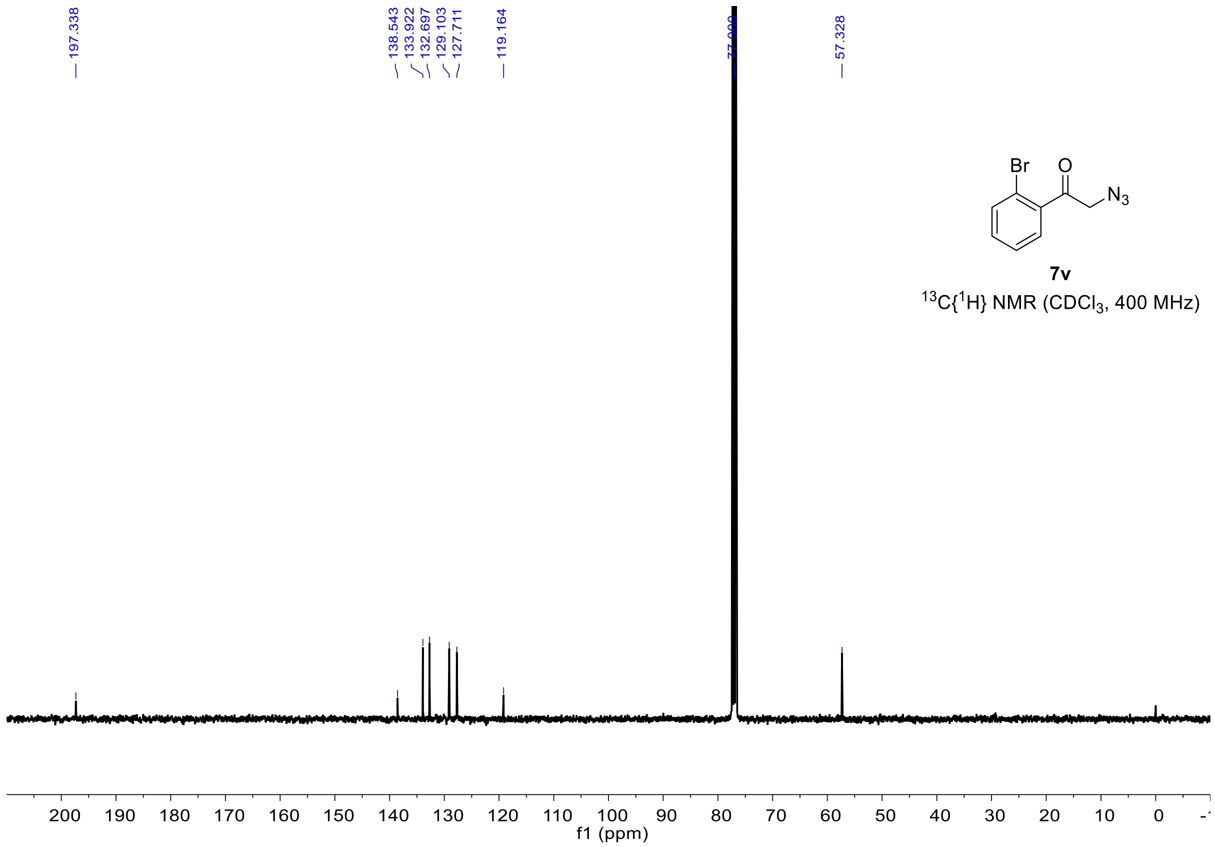

7v

$^{13}C\{^1H\}$ NMR ($CDCl_3$, 400 MHz)

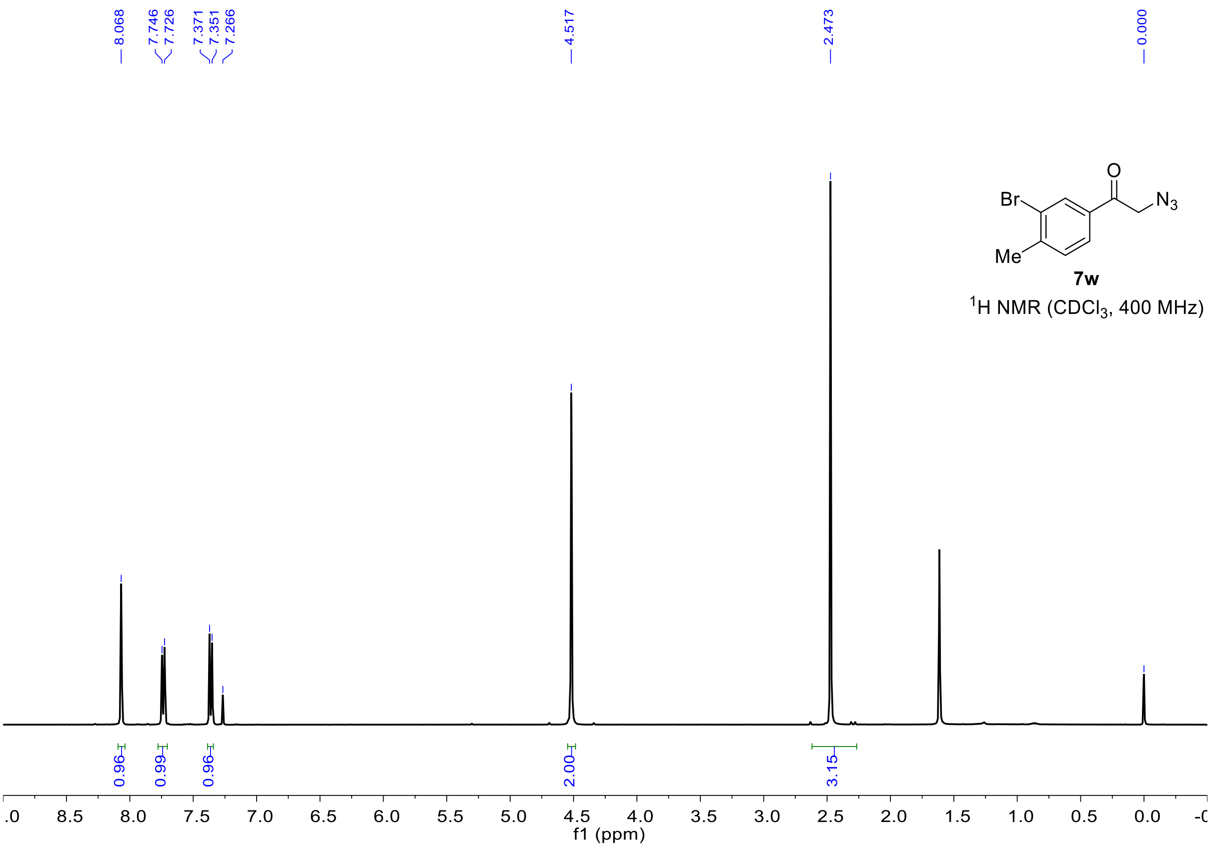
8.068
7.746
7.726
7.371
7.351
7.266
4.517
2.473
0.000
Br
Me
O
N3
7w
1H NMR (CDCl3, 400 MHz)
0.96
0.99
0.96
2.00
3.15
f1 (ppm)

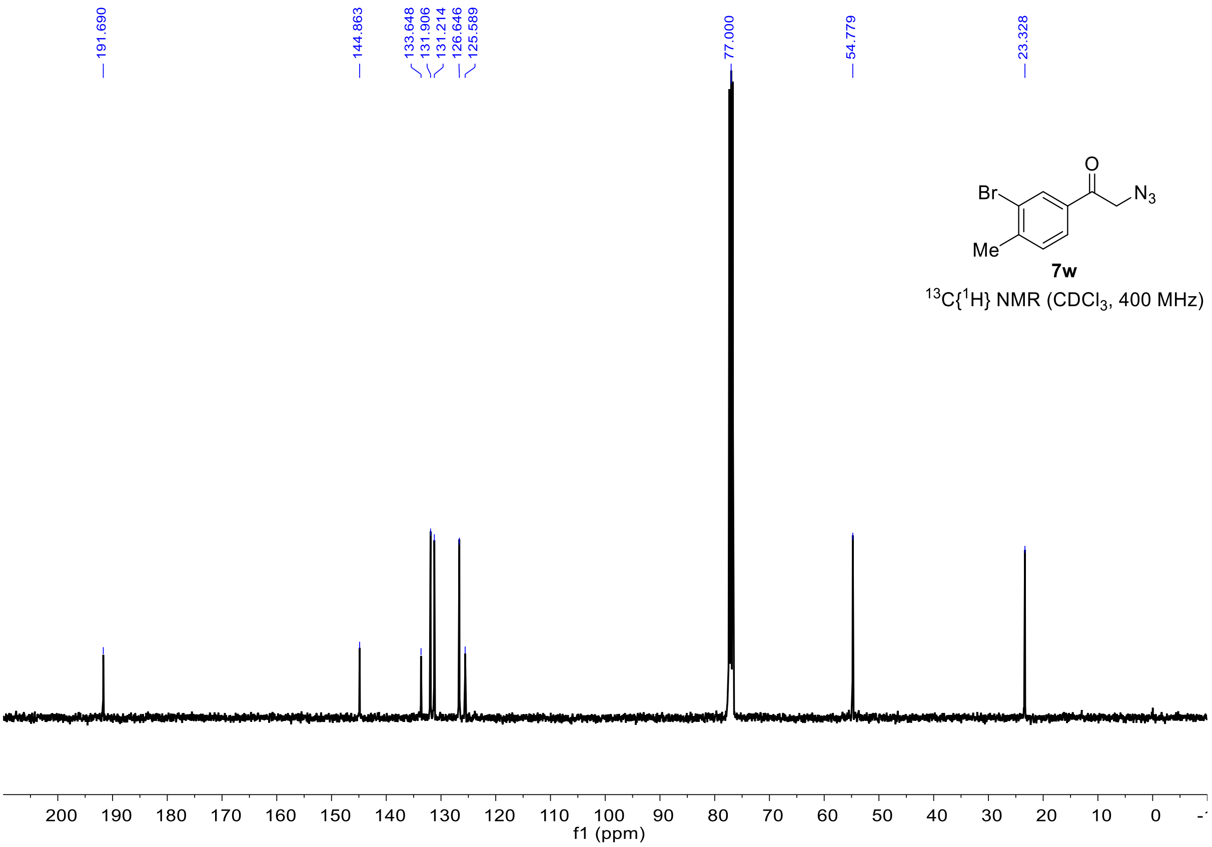
191.690
144.863
133.648
131.906
131.214
126.646
125.589
77.000
54.779
23.328
Br
Me
O
N3
7w
13C{1H} NMR (CDCl3, 400 MHz)
f1 (ppm)

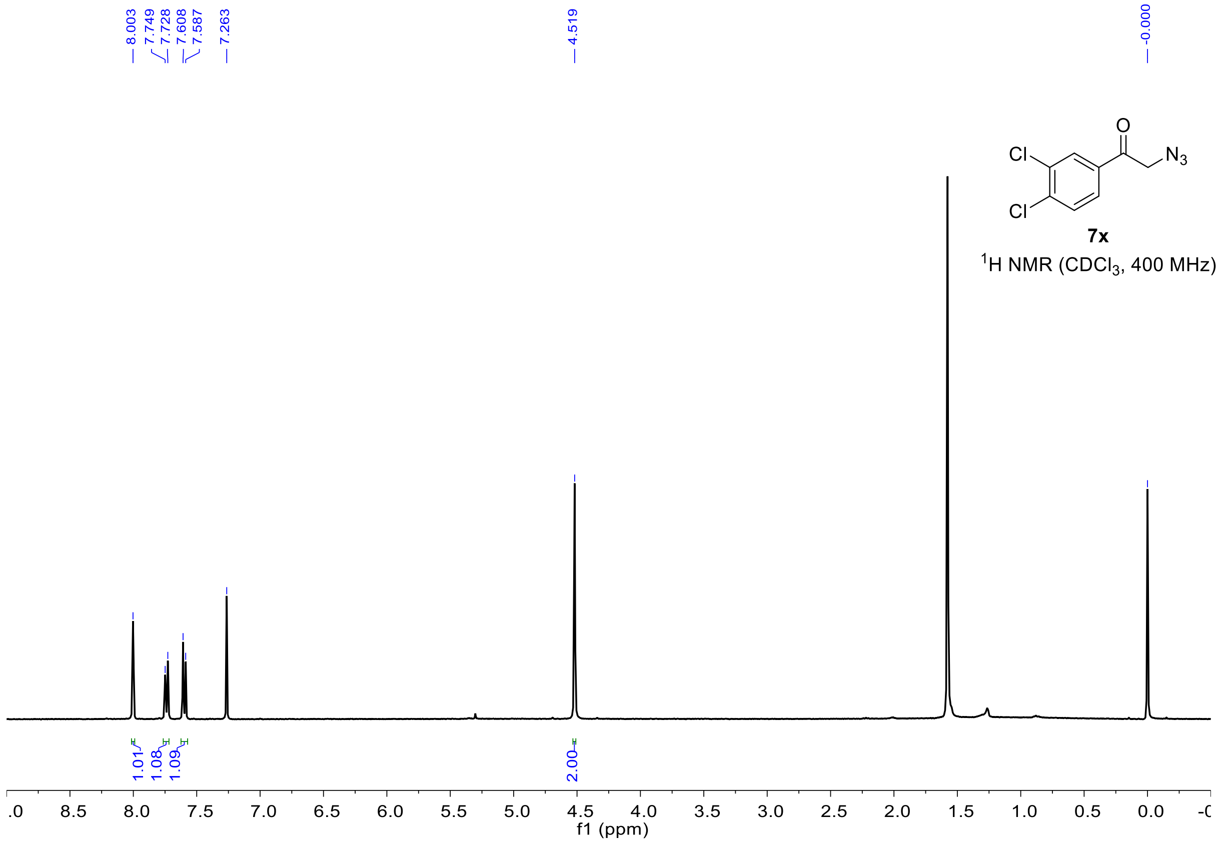
8.003
7.749
7.728
7.608
7.587
7.263
4.519
-0.000
Cl
Cl
O
N3
7x
¹H NMR (CDCl3, 400 MHz)
1.01
1.08
1.09
2.00
f1 (ppm)

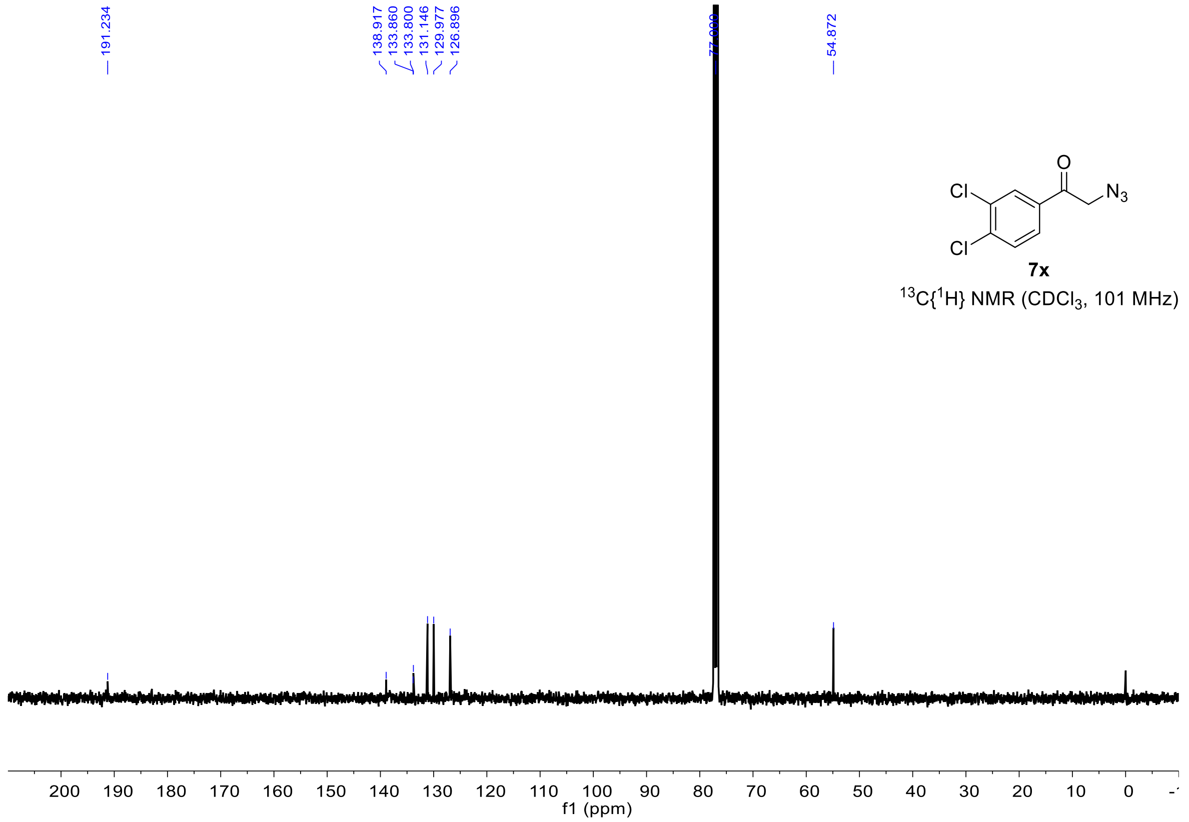
191.234
138.917
133.860
133.800
131.146
129.977
126.896
54.872
Cl
Cl
O
N3
7x
¹³C{¹H} NMR (CDCl3, 101 MHz)
f1 (ppm)

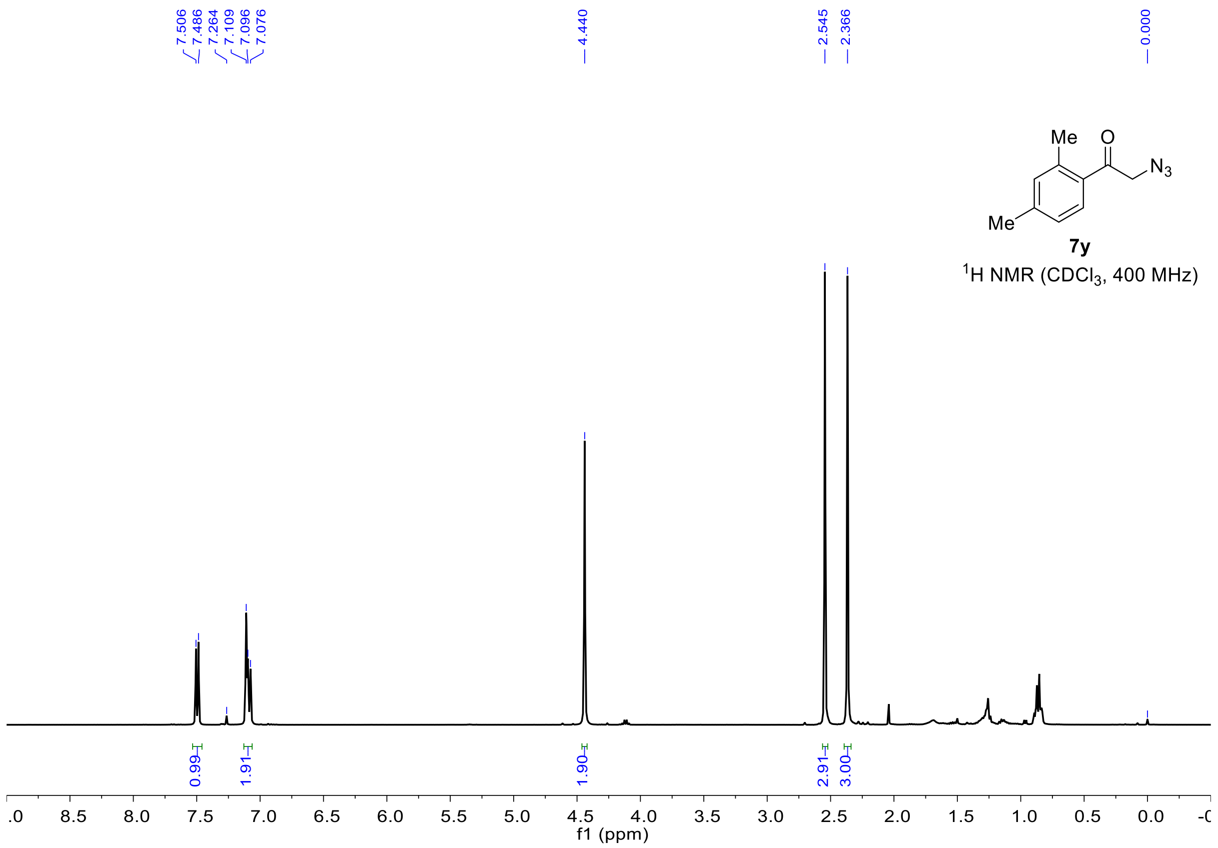
7.506
7.486
7.264
7.109
7.096
7.076
4.440
2.545
2.366
0.000
Me
O
N3
Me
7y
1H NMR (CDCl3, 400 MHz)
0.99
1.91
1.90
2.91
3.00
f1 (ppm)

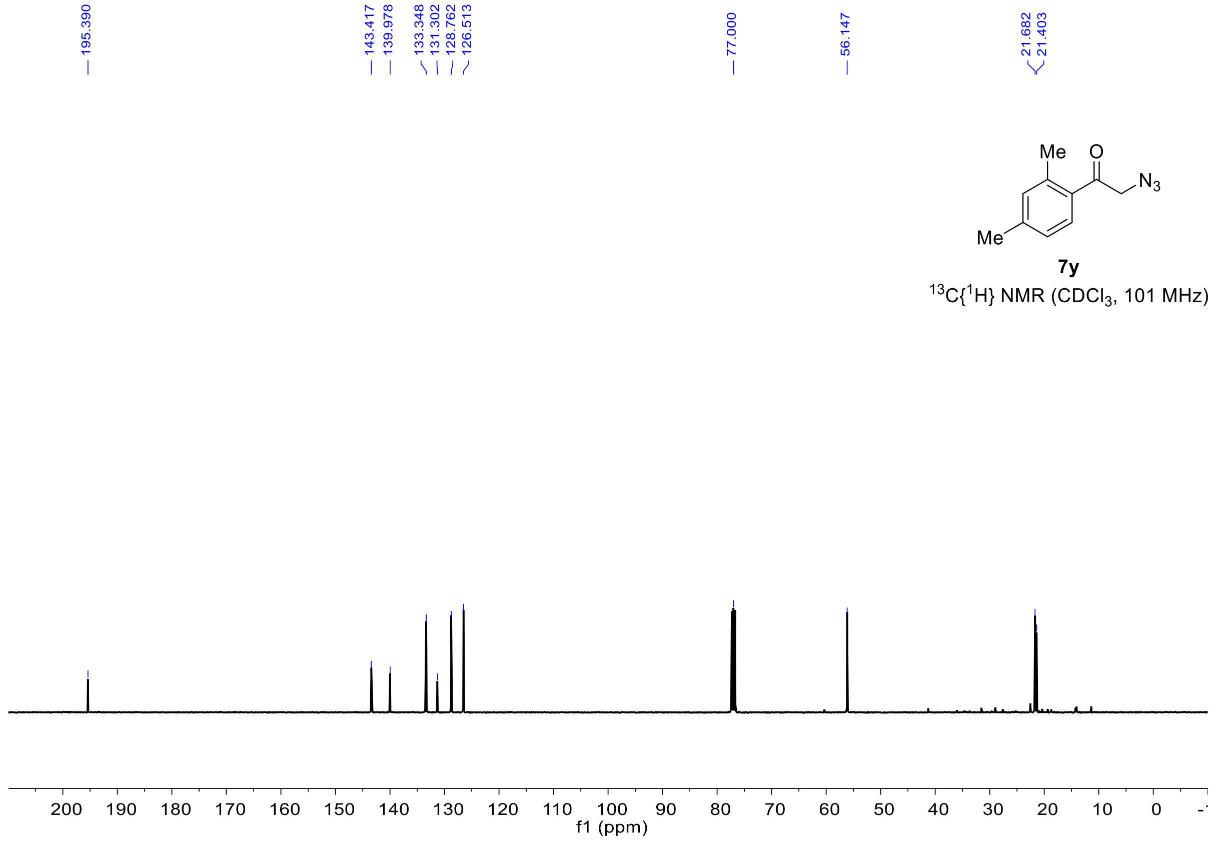
195.390
143.417
139.978
133.348
131.302
128.762
126.513
77.000
56.147
21.682
21.403
Me
O
N3
Me
7y
13C{1H} NMR (CDCl3, 101 MHz)
f1 (ppm)

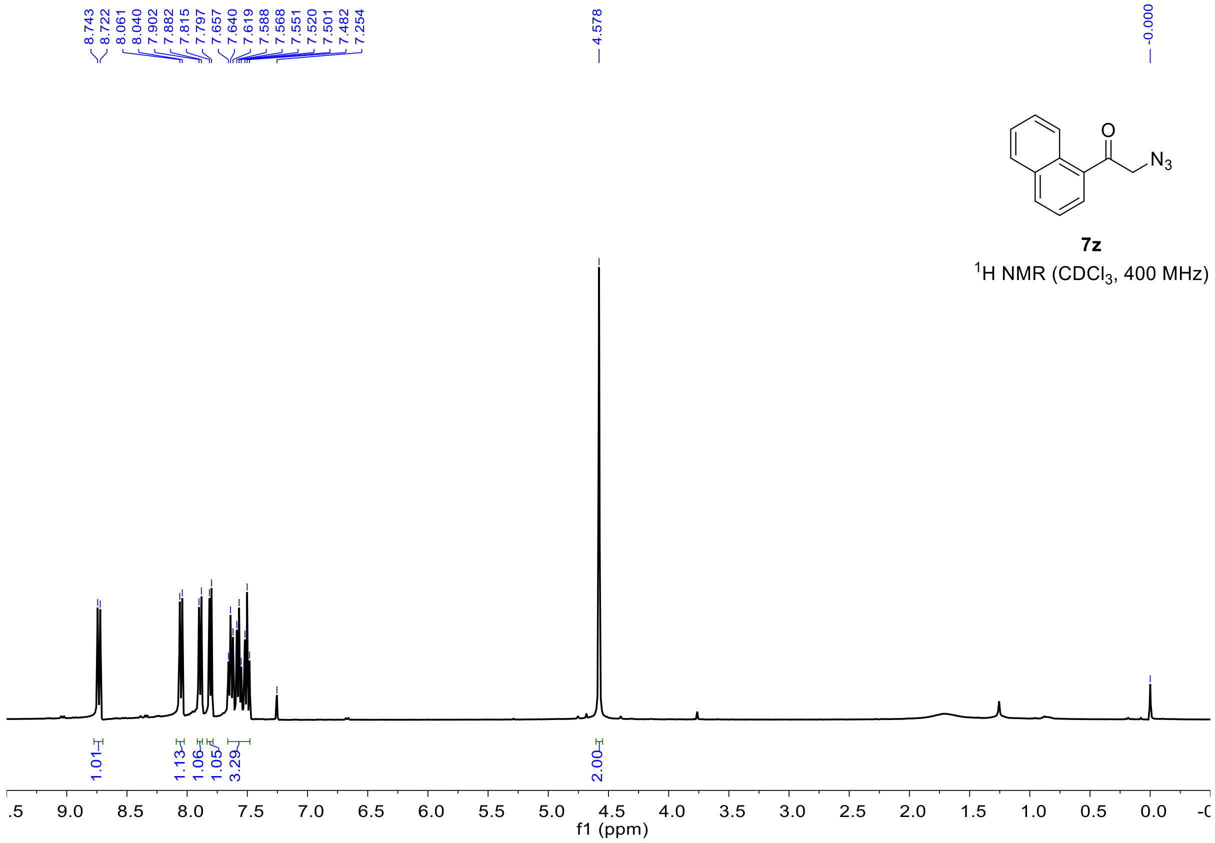
8.743
8.722
8.061
8.040
7.902
7.882
7.815
7.797
7.657
7.640
7.619
7.588
7.568
7.551
7.520
7.501
7.482
7.254
4.578
-0.000
O
N3
7z
1H NMR (CDCl3, 400 MHz)
1.01
1.13
1.06
1.05
3.29
2.00
f1 (ppm)

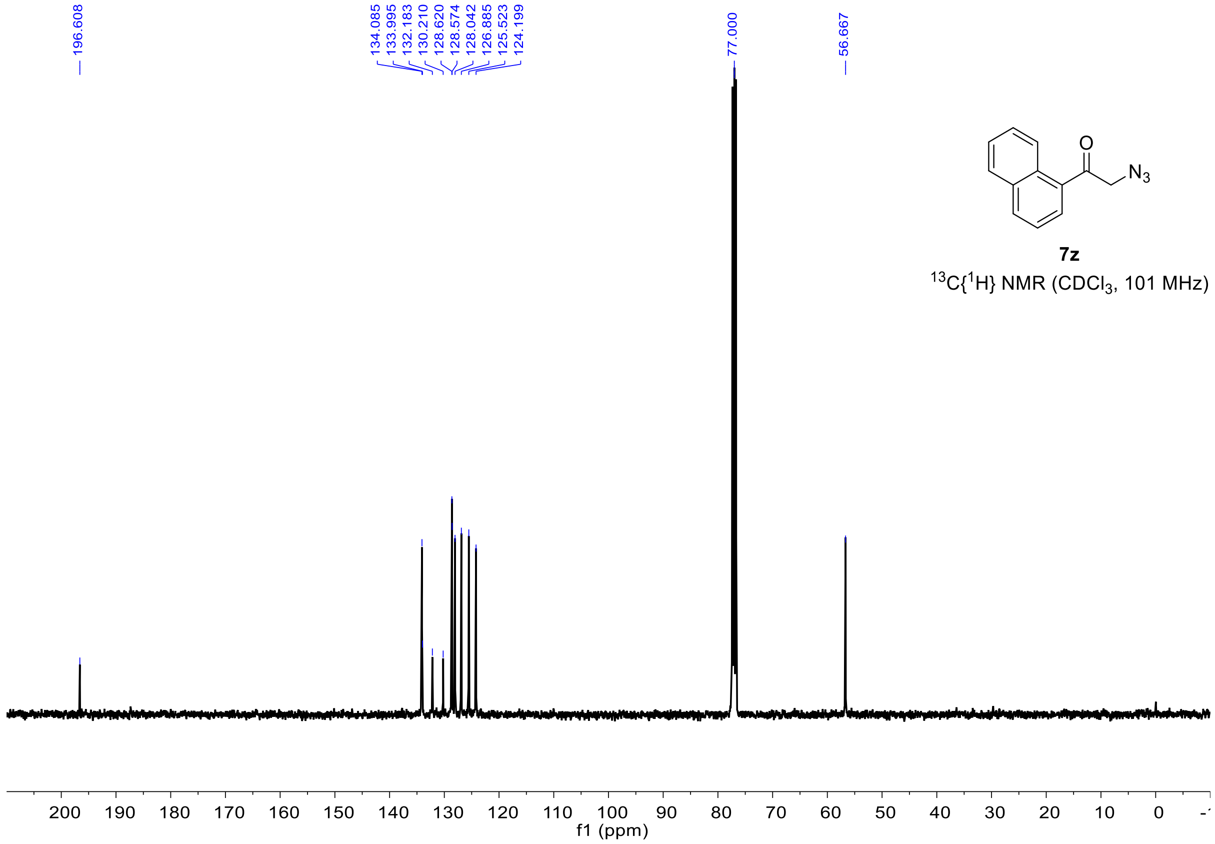
196.608
134.085
133.995
132.183
130.210
128.620
128.574
128.042
126.885
125.523
124.199
77.000
56.667
O
N3
7z
13C{1H} NMR (CDCl3, 101 MHz)
f1 (ppm)

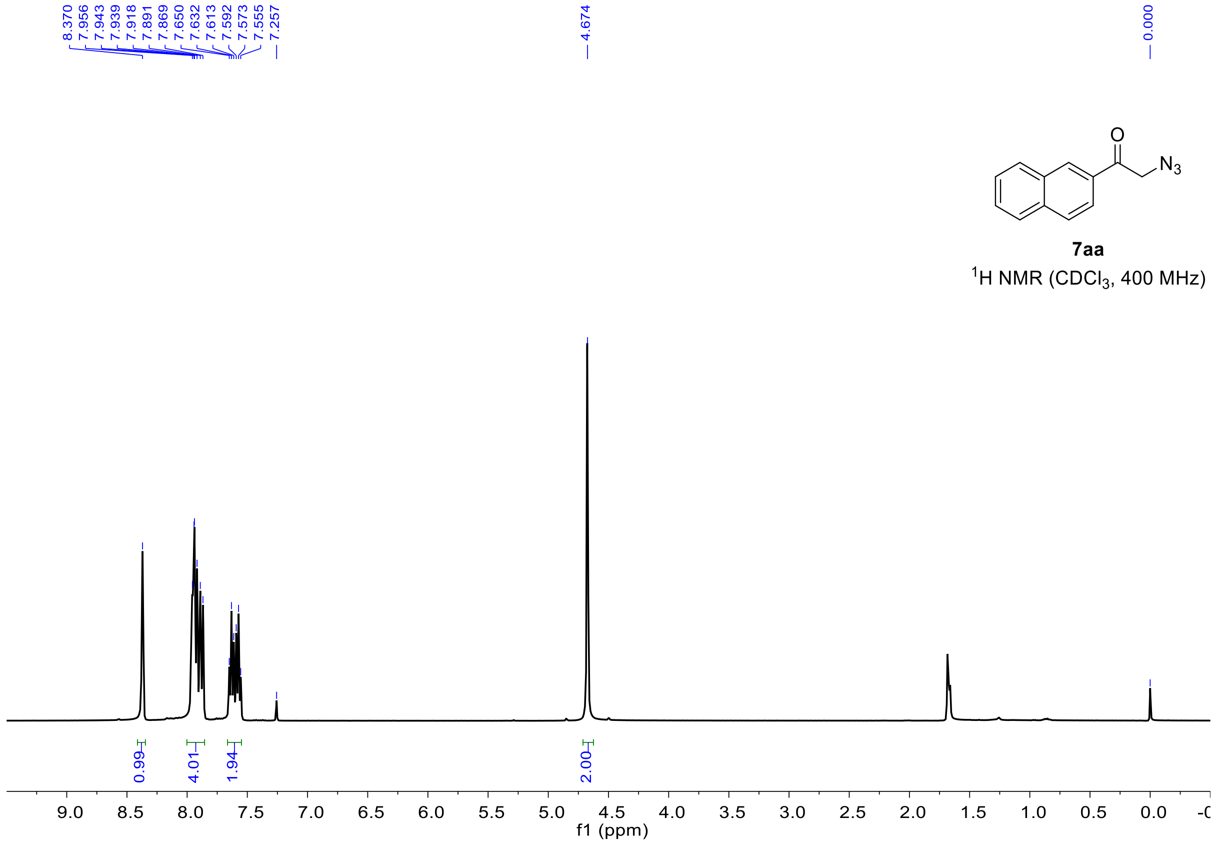
8.370
7.956
7.943
7.939
7.918
7.891
7.869
7.650
7.632
7.613
7.592
7.573
7.555
7.257
4.674
0.000
O
N3
7aa
1H NMR (CDCl3, 400 MHz)
0.99
4.01
1.94
2.00
f1 (ppm)

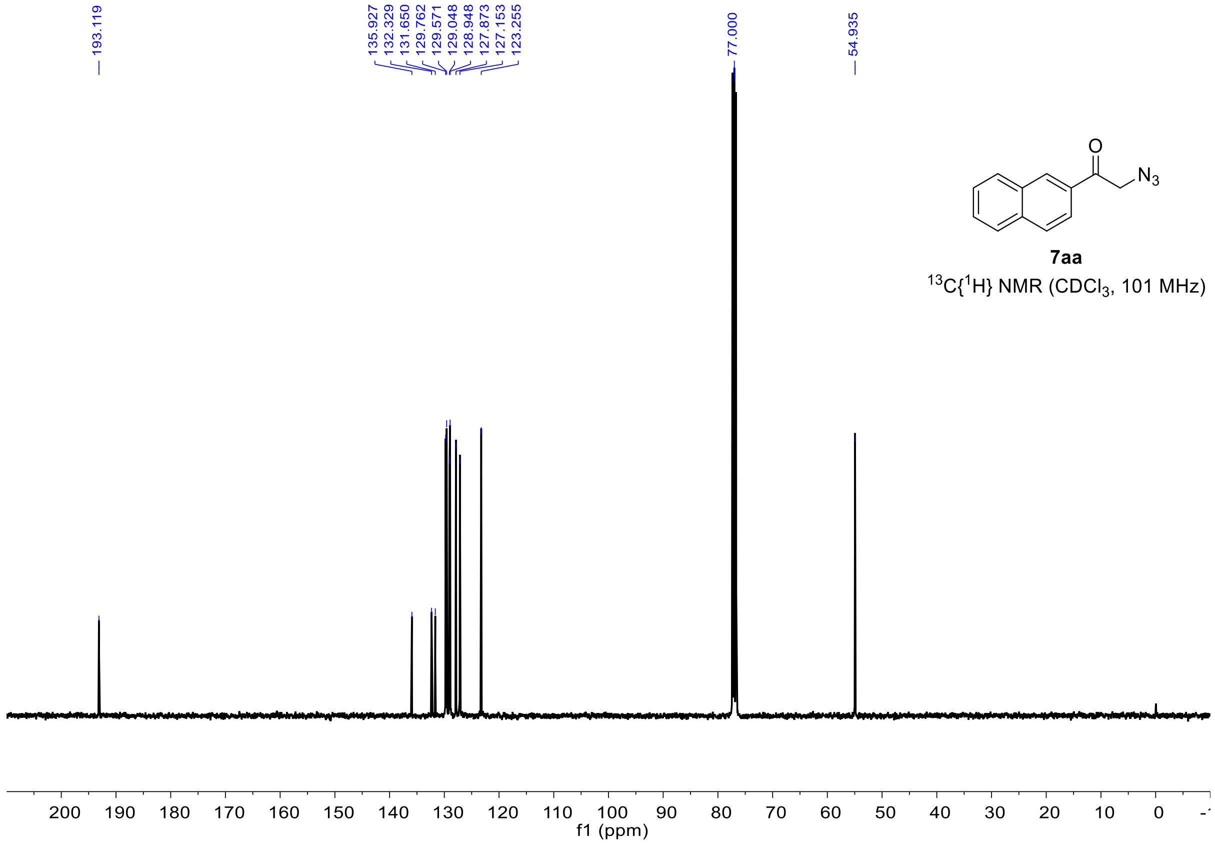
193.119
135.927
132.329
131.650
129.762
129.571
129.048
128.948
127.873
127.153
123.255
77.000
54.935
O
N3
7aa
13C{1H} NMR (CDCl3, 101 MHz)
f1 (ppm)

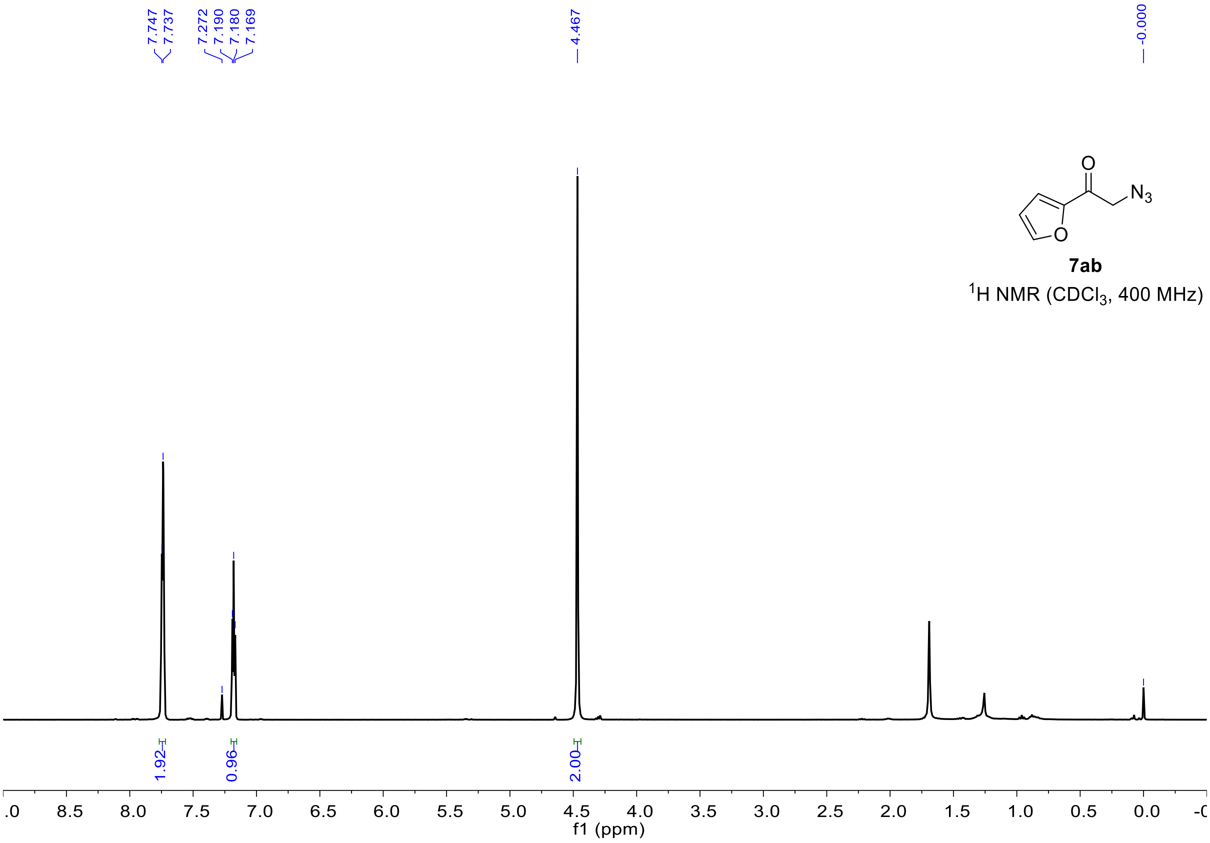
7.747
7.737
7.272
7.190
7.180
7.169
4.467
-0.000
O
N_3
O
7ab
^{1}H NMR ($CDCl_3$, 400 MHz)
1.92
0.96
2.00
f1 (ppm)

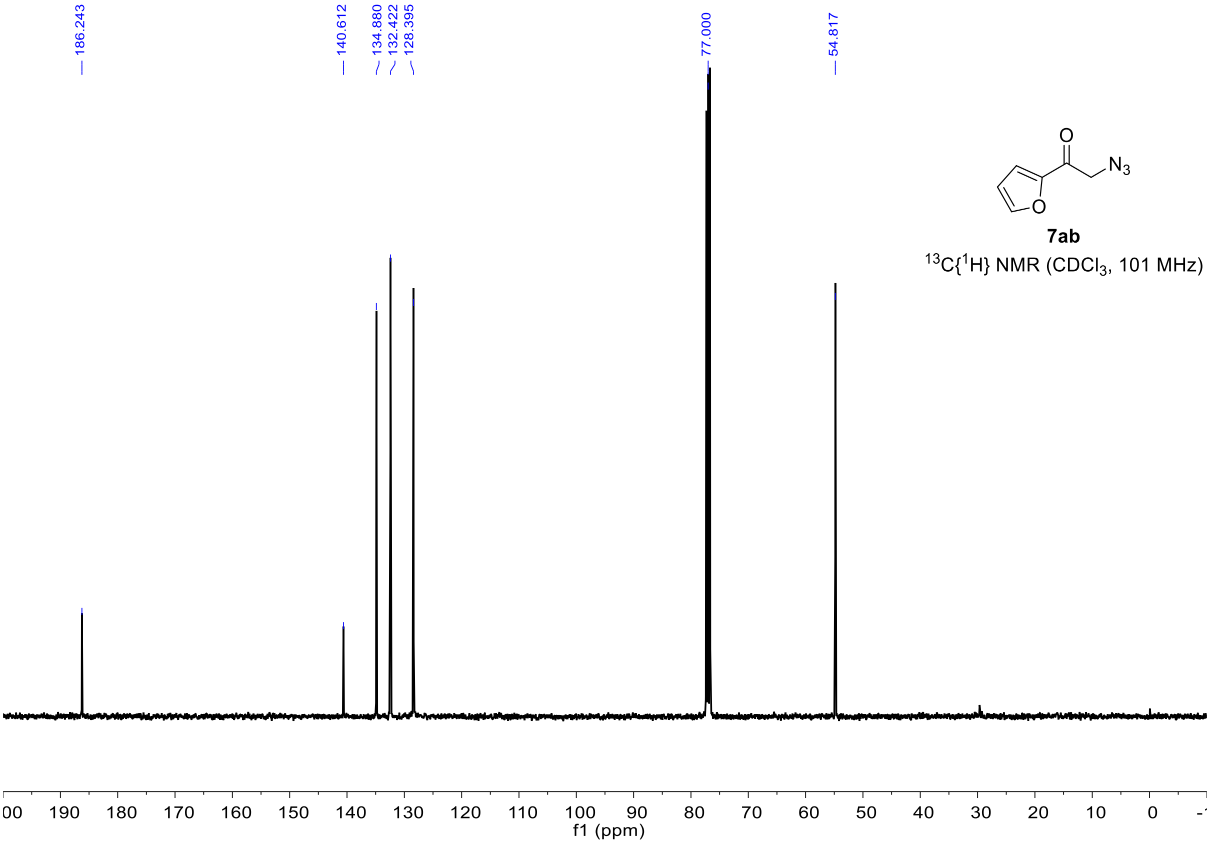
186.243
140.612
134.880
132.422
128.395
77.000
54.817
O
N_3
O
7ab
^{13}C{^{1}H} NMR ($CDCl_3$, 101 MHz)
f1 (ppm)

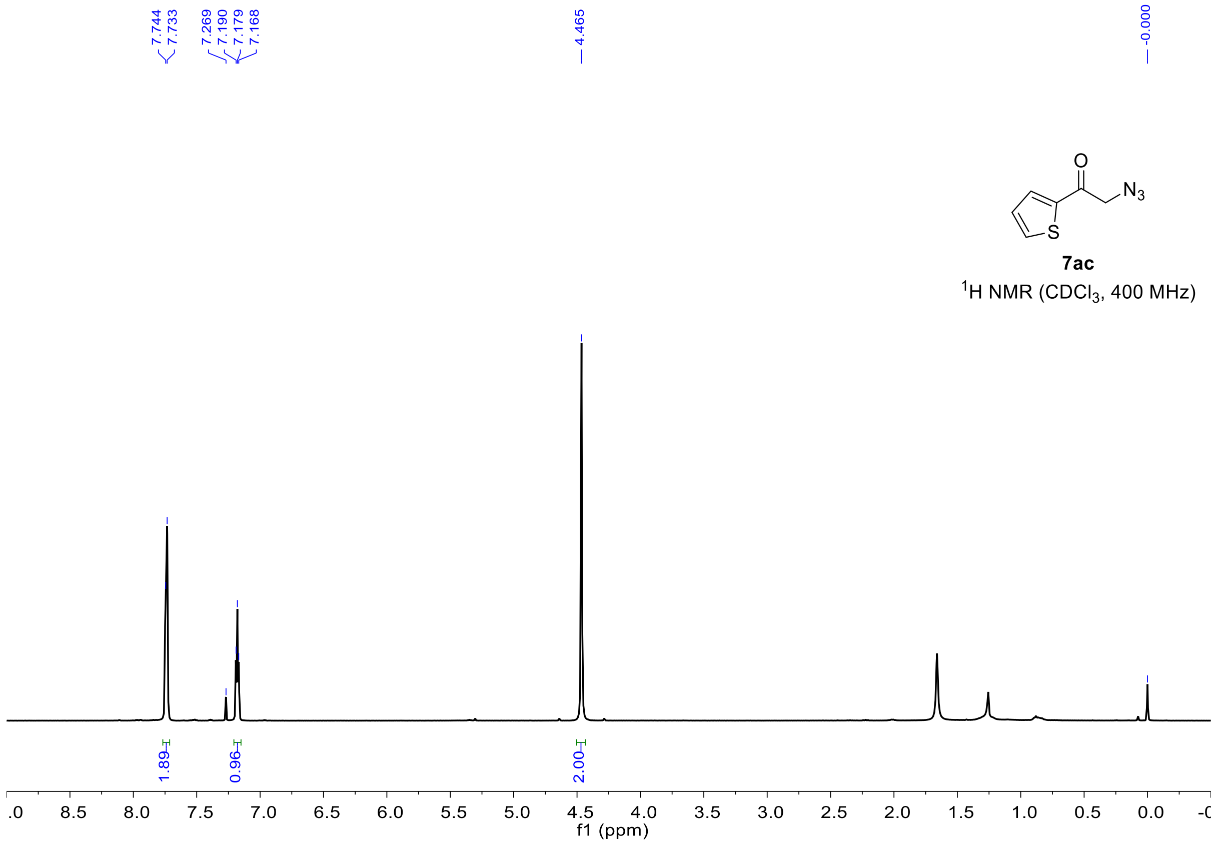
7.744
7.733
7.269
7.190
7.179
7.168
4.465
-0.000
O
N3
S
7ac
1H NMR (CDCl3, 400 MHz)
1.89
0.96
2.00
f1 (ppm)

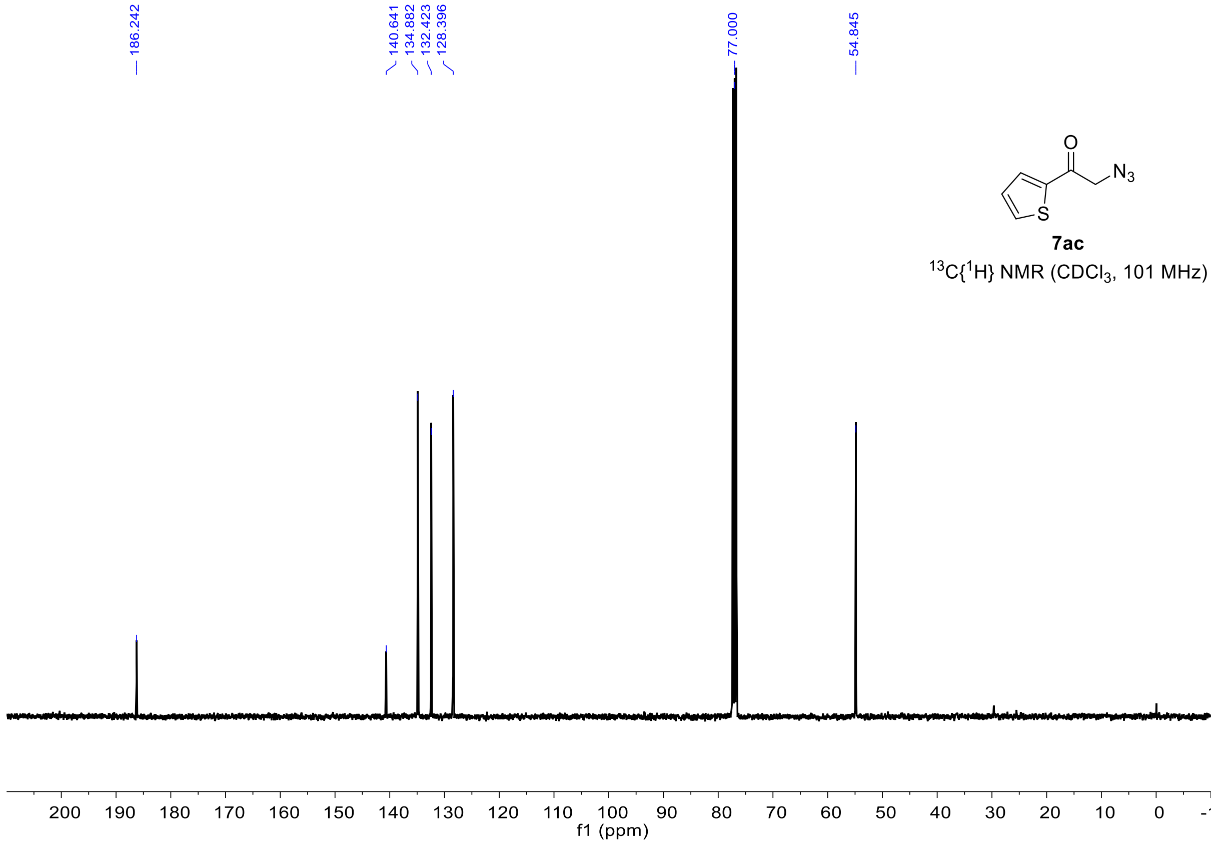
186.242
140.641
134.882
132.423
128.396
77.000
54.845
O
N3
S
7ac
13C{1H} NMR (CDCl3, 101 MHz)
f1 (ppm)

VI. Computational details

All the calculations in this work were performed on the basis of density functional theory (DFT) using the Gaussian G16 package (Revision B.01).[23] All the reactants, complexes, transition states, intermediates and products were fully optimized at the M06-2X[24]/6-31+G(d, p)[25,26,27] level of theory. The nature of the local minima was established with analytical frequencies calculations and the single point energies were subsequently obtained at the same functional and basis set with SMD (dimethylsulfoxide) solvent model[28] and the temperature was set to 298.15 K. Intrinsic reaction coordinate (IRC)[29,30] calculations were carried out to ascertain the true nature of the transition states. Three dimensional diagrams of the computed species were generated using CYL view visualization software.[31]

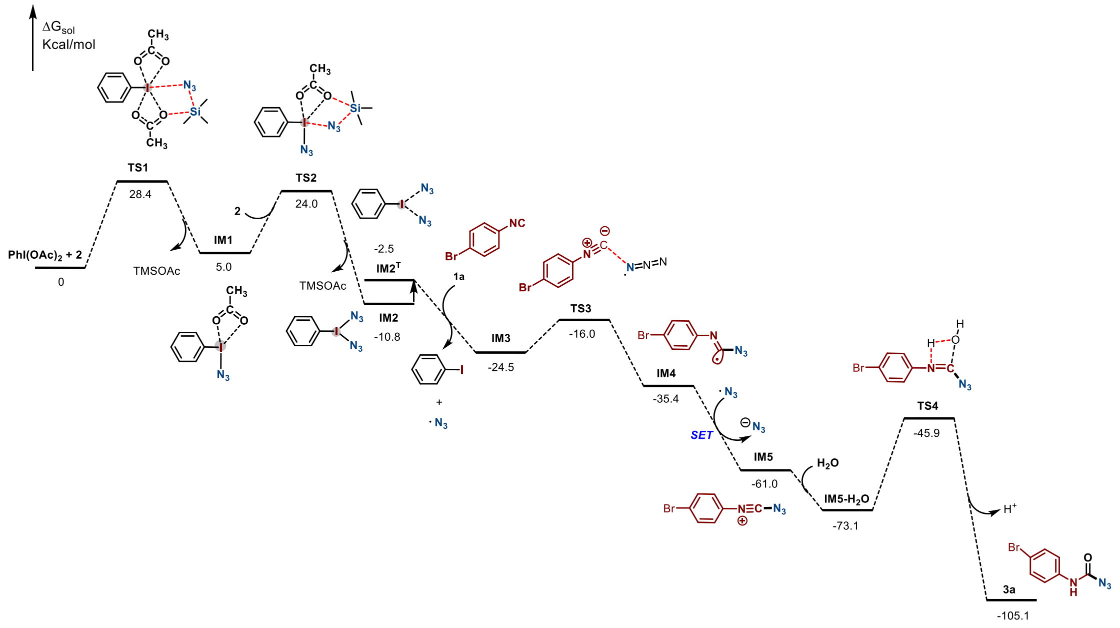

Figure S1. Gibbs free energy profiles of the overall reaction. (The data were optimized at the M06-2X/6-31+G(d,p), Lanl2DZ for I//SMO(DMSO) level of theory).

Cartesian coordination and energies (in Hartree) for the calculated species

$PhI(OAc)_2$ + **2**

Sum of electronic and thermal Free Energies = -1272.712408

I	0.40028300	0.33785600	-0.42492500
C	2.24016000	-0.51341200	0.20056400
C	3.36531600	-0.31558200	-0.58768400

C	2.26784500	-1.24096000	1.38216900
C	4.57232000	-0.87536100	-0.16543300
H	3.31349400	0.25958100	-1.50602400
C	3.48330800	-1.79550300	1.78648500
H	1.37152700	-1.38091700	1.97728400
C	4.63157400	-1.61281100	1.01667900
H	5.46250800	-0.73595400	-0.77080100
H	3.52612300	-2.36396700	2.71019900
H	5.57359500	-2.04576800	1.33822100
O	1.88735800	2.60294000	1.12876700
C	2.06330800	2.79766500	-0.06372500
O	1.56278500	2.02379000	-1.01402500
C	2.89153100	3.93072900	-0.61315300
H	3.20228700	4.59373900	0.19396000
H	3.77391100	3.51516700	-1.10922300
H	2.31878100	4.48563600	-1.35989000
O	0.41492100	-2.63982800	-1.40956400
C	-0.27089000	-2.58121000	-0.40203000
O	-0.49474800	-1.45939900	0.26807300
C	-0.91261300	-3.78281000	0.24276300
H	-1.00026500	-4.59147200	-0.48335800
H	-0.26835800	-4.11011500	1.06623900
H	-1.89055000	-3.52891900	0.65509600
Si	-3.69203900	0.81058000	0.60926400
C	-5.00409600	1.97126300	-0.03135600
H	-5.58760300	2.38050000	0.80121900
H	-4.55238400	2.80875400	-0.57353900
H	-5.69107500	1.45249200	-0.70819200
C	-4.41323700	-0.70277400	1.43173300
H	-5.10589200	-1.22900100	0.76522600

H	-3.62589500	-1.40155500	1.73545200
H	-4.96985100	-0.41119800	2.32946400
C	-2.44971200	1.70101000	1.67560100
H	-1.70950400	1.01129500	2.09389200
H	-1.92138100	2.47296800	1.10502600
H	-2.96749600	2.19215700	2.50776300
N	-2.77704100	0.22638400	-0.83647200
N	-2.90315200	-0.92218100	-1.22373200
N	-2.96692500	-1.97983100	-1.62527500

TS1

Sum of electronic and thermal Free Energies = -1272.669074

I	-0.72891300	-0.48493700	-1.03253200
C	-2.10227000	0.82838000	-0.12536700
C	-3.24592000	1.18163000	-0.83196400
C	-1.82680200	1.29023600	1.15658100
C	-4.15779500	2.03847700	-0.21413700
H	-3.42669600	0.80598000	-1.83378700
C	-2.74868200	2.15080200	1.75222800
H	-0.92230100	0.99355800	1.67717200
C	-3.90852900	2.52053500	1.07057600
H	-5.05886700	2.32875000	-0.74493600
H	-2.55730400	2.52477800	2.75301000
H	-4.62182500	3.18845200	1.54333600
O	-1.56196500	-2.01015500	1.51267700
C	-2.17954200	-2.47763100	0.57710000
O	-2.05512900	-2.02996800	-0.67797200
C	-3.17238800	-3.60217400	0.67347300
H	-3.23573700	-3.94892600	1.70417800

H	-4.15056900	-3.25056800	0.33520900
H	-2.86353400	-4.42034000	0.01820900
O	0.36089600	1.43963900	-1.51047400
C	1.25249200	1.81862800	-0.69689100
O	1.35951100	1.30195200	0.46378600
C	2.16988900	2.92977800	-1.09620300
H	1.88102800	3.36127500	-2.05333300
H	2.17506900	3.69221000	-0.31331900
H	3.18037900	2.51142600	-1.16620300
Si	2.62208300	0.00400800	1.16190500
C	2.92043400	-1.87187400	1.39974300
H	3.15877400	-2.08470300	2.44836900
H	1.99683700	-2.41680800	1.16023900
H	3.71926200	-2.29016500	0.77729300
C	4.21533500	0.98299400	1.35081800
H	4.01304700	2.05971800	1.34727800
H	4.67557700	0.73472700	2.31487900
H	4.94775100	0.76866100	0.56422600
C	1.61853300	0.24473200	2.76937800
H	1.39080100	1.29621300	2.97278000
H	0.67042800	-0.30765300	2.72421000
H	2.18823200	-0.16156900	3.61413900
N	2.69854000	-0.30942300	-0.81829100
N	3.67887600	-0.84736000	-1.25949600
N	4.61227700	-1.35154200	-1.69386700

IM1

Sum of electronic and thermal Free Energies = -1272.692865

I	-0.95401900	0.22263000	-0.41916400
C	0.63130400	1.59545200	-0.08072500

C	0.79321300	2.15160200	1.18673800
C	1.49035600	1.91291100	-1.12999400
C	1.83895500	3.04809300	1.40405000
H	0.10985000	1.89744500	1.99216600
C	2.54280500	2.80094100	-0.89811800
H	1.34334800	1.48611800	-2.11843600
C	2.71357100	3.36878300	0.36421400
H	1.97161000	3.49012000	2.38670000
H	3.21950500	3.05251800	-1.70898300
H	3.52957900	4.06325300	0.53893400
O	-3.17951100	-0.70819900	-0.84618300
C	-3.71625900	-1.29691000	0.16562900
O	-3.35452800	-1.13669300	1.34195100
C	-4.87331000	-2.22369700	-0.16799600
H	-5.67667200	-1.64491600	-0.63387000
H	-4.54688100	-2.97490900	-0.89259000
H	-5.25114600	-2.71438700	0.72974000
O	1.18619100	-1.33078800	-2.62148600
C	1.97589600	-1.31408500	-1.70275000
O	1.50888100	-1.37579300	-0.44075100
C	3.45982500	-1.16565000	-1.88477900
H	3.70362200	-1.26775500	-2.94144300
H	3.75807300	-0.16948700	-1.53878500
H	4.01515100	-1.90704200	-1.30517300
Si	2.32491700	-1.62051400	1.07089300
C	3.01915900	-3.35155500	1.06724300
H	3.45898000	-3.56232100	2.04931000
H	2.22585800	-4.08561400	0.89027700
H	3.80024200	-3.49711700	0.31487300
C	3.63283800	-0.30732400	1.29508100

H	4.58946600	-0.59401800	0.84764000
H	3.31538300	0.64549900	0.85646600
H	3.79670300	-0.14338700	2.36680800
C	0.94888200	-1.46620400	2.31607500
H	0.56937300	-0.44246200	2.39227000
H	0.11374300	-2.12915400	2.06461100
H	1.32661100	-1.75899500	3.30284300
N	-2.17839600	1.91497700	-0.28142700
N	-3.23049900	1.72165600	0.33844400
N	-4.21823800	1.65508800	0.87920800

TS2

Sum of electronic and thermal Free Energies = -1208.426271

I	0.59635200	0.99597900	-0.50278600
C	1.95640400	-0.53220000	0.02280800
C	1.79704100	-1.16339900	1.25115100
C	2.97231900	-0.85158100	-0.87141200
C	2.69994000	-2.17139000	1.58994300
H	0.99115500	-0.88408500	1.92277800
C	3.87198400	-1.85361100	-0.50656500
H	3.06348600	-0.33723200	-1.82311600
C	3.73256900	-2.51128000	0.71557600
H	2.59607600	-2.68109000	2.54235000
H	4.67407900	-2.12178700	-1.18649200
H	4.43322400	-3.29403200	0.98848100
O	-2.33755500	1.79561400	-1.05207800
C	-2.44112400	1.79725700	0.17807800
O	-2.36152600	0.73022900	0.91571600
C	-2.63668000	3.09319700	0.93184500
H	-3.57162700	3.55532100	0.60098200

H	-2.66821200	2.93426800	2.00960900
H	-1.82276800	3.77974700	0.68092700
N	2.26931600	2.41038900	-0.45407900
N	3.00671500	2.21487800	0.50196800
N	3.72678800	2.04500700	1.36479800
Si	-2.71353300	-1.06260000	0.33950700
C	-4.34121600	-0.51280000	-0.46272500
H	-5.08906100	-1.30246100	-0.32625400
H	-4.74322400	0.42510900	-0.06896200
H	-4.19458300	-0.39142900	-1.54224000
C	-2.76527600	-2.87250800	-0.37850000
H	-2.81070800	-2.93491700	-1.47420600
H	-1.94703600	-3.51900300	-0.03324900
H	-3.69607500	-3.31063500	0.00436300
C	-2.63724200	-1.52592800	2.17046400
H	-2.99095700	-2.54591700	2.34846400
H	-1.59439200	-1.46894700	2.50887600
H	-3.21361100	-0.83067800	2.78769100
N	-0.95344500	-0.80905500	-0.51497200
N	-0.46935600	-1.71602100	-1.19562400
N	0.03532300	-2.51148600	-1.81616500

IM2

Sum of electronic and thermal Free Energies= -570.985954

I	1.39405000	0.03307700	-0.00532600
C	-0.71919800	-0.01517800	-0.00634200
C	-1.38459100	0.03877000	1.21436900
C	-1.38131000	-0.10064400	-1.22698200
C	-2.77936000	0.00712000	1.20265400
H	-0.83514600	0.10343200	2.14810500

C	-2.77592300	-0.13785800	-1.21457700
H	-0.82959600	-0.13752300	-2.16084400
C	-3.46973000	-0.08271300	-0.00585100
H	-3.32016900	0.04522900	2.14274700
H	-3.31468500	-0.20238800	-2.15442100
H	-4.55489000	-0.10995800	-0.00568800
N	1.30366700	-2.16803700	-0.56314900
N	0.39171000	-2.74445800	-0.00870300
N	-0.47673600	-3.29495000	0.49071000
N	1.20771200	2.22959100	0.55214300
N	0.24640800	2.75767800	0.03490900
N	-0.66840100	3.26177200	-0.42980000

IM2-T

Sum of electronic and thermal Free Energies = -570.972699

I	-0.00683600	-1.69919800	0.00173400
C	0.00023600	0.41257600	0.00177100
C	-0.13825100	1.09528500	1.20992100
C	0.14467500	1.09431800	-1.20629500
C	-0.13627200	2.49181600	1.20050200
H	-0.25104500	0.55091400	2.14217400
C	0.15427200	2.49079600	-1.19674100
H	0.25341800	0.54916500	-2.13858300
C	0.01178800	3.18936000	0.00193500
H	-0.24704600	3.02949400	2.13737000
H	0.26975300	3.02762400	-2.13352700
H	0.01639000	4.27493000	0.00201400
N	-3.21620500	-0.66280300	0.52426700
N	-3.08807900	0.34393200	-0.07044000
N	-2.95710100	1.34937400	-0.66142100

N	3.21761100	-0.67977800	-0.54019700
N	3.09307100	0.32257900	0.06253300
N	2.96529200	1.32390200	0.66127200

IM3

Sum of electronic and thermal Free Energies = -3062.043280

C	-0.12253000	-0.56094200	-1.21692900
C	1.25497200	-0.72464000	-1.21792800
C	1.93023200	-0.80593800	-0.00049800
C	1.25520400	-0.72581400	1.21716800
C	-0.12229400	-0.56218700	1.21664900
C	-0.79510600	-0.48331800	-0.00002000
H	-0.66008100	-0.49103800	-2.15377300
H	1.80714300	-0.78214200	-2.14763300
H	1.80760400	-0.78424400	2.14668200
H	-0.65966300	-0.49336000	2.15367200
Br	-2.67834200	-0.26063800	0.00031900
C	4.47290200	-1.05383000	-0.00096800
N	3.31242800	-0.94507900	-0.00067300
N	-0.15576000	2.44447500	-0.00382500
N	1.00073300	2.27924100	0.00049900
N	2.15784200	2.10323700	0.00472100

TS3

Sum of electronic and thermal Free Energies = -3062.029544

C	1.23863500	1.21564000	0.13956200
C	-0.13533200	1.21737800	0.33050600

C	-0.80838700	0.00068500	0.42278200
C	-0.13574100	-1.21631300	0.33156600
C	1.23823000	-1.21520100	0.14066900
C	1.90716100	0.00006500	0.04608400
H	1.77499800	2.15273000	0.06462900
H	-0.68434700	2.14756000	0.40684800
H	-0.68505500	-2.14624600	0.40880400
H	1.77427900	-2.15253900	0.06666300
Br	3.78850400	-0.00038300	-0.21857800
C	-3.33931600	0.00092600	0.75163600
N	-2.18190500	0.00097300	0.60715600
N	-4.56954100	-0.00092600	-0.90182600
N	-5.69874000	-0.00062700	-0.49822100
N	-6.77395600	-0.00044800	-0.10333000

IM4

Sum of electronic and thermal Free Energies = -3062.060658

C	-0.94412100	1.02318200	0.18231800
C	0.41882900	0.76851000	0.24047200
C	0.89281200	-0.54124600	0.11911200
C	-0.00806400	-1.59316000	-0.04411800
C	-1.37384300	-1.34543300	-0.10562200
C	-1.82636800	-0.03765200	0.00875600
H	-1.30895100	2.03864200	0.27579400
H	1.11608800	1.58544500	0.38447200
H	0.36768700	-2.60619000	-0.12645800
H	-2.06884900	-2.16536900	-0.23656000
Br	-3.69692300	0.31151100	-0.06318500
C	3.22670200	-0.11314700	0.00395100
N	2.25958000	-0.86224700	0.18923700

N	4.54565700	-0.47029700	0.16350600
N	5.38553700	0.41305500	-0.11063500
N	6.23360400	1.10209800	-0.31567800

IM5

Sum of electronic and thermal Free Energies = -3061.900701

C	1.18843200	-1.21807700	-0.05590300
C	-0.19304200	-1.22165100	-0.16740500
C	-0.85851600	-0.00014800	-0.22233600
C	-0.19315000	1.22142000	-0.16759400
C	1.18832600	1.21798500	-0.05610800
C	1.85900100	-0.00001100	-0.00340000
H	1.72905800	-2.15433600	-0.01069400
H	-0.74904100	-2.14960100	-0.21149600
H	-0.74923300	2.14931400	-0.21183200
H	1.72887500	2.15429500	-0.01105200
Br	3.74980700	0.00008900	0.14275900
C	-3.38890600	-0.00023300	-0.40653700
N	-2.24362700	-0.00019700	-0.33810300
N	-4.65841400	-0.00002200	-0.68317600
N	-5.47533000	0.00015500	0.28914300
N	-6.31059400	0.00027800	1.00702300

TS4

Sum of electronic and thermal Free Energies = -3138.276463

C	-0.91781500	-0.48146600	0.95946000
C	0.44193800	-0.20163100	1.02222900
C	0.97991800	0.77002900	0.18171500

C	0.17559400	1.48825700	-0.69804200
C	-1.18188300	1.20844700	-0.76424400
C	-1.71090700	0.22503400	0.06443200
H	-1.34489100	-1.23497600	1.60896700
H	1.07407100	-0.72312800	1.73350000
H	0.61401200	2.25093000	-1.33019400
H	-1.81336700	1.75433500	-1.45341000
Br	-3.57297500	-0.14990600	-0.01874000
C	3.35158900	0.31656000	0.05338700
N	2.36067300	1.08359900	0.23932200
N	3.68631000	-0.94594100	-0.25228500
N	2.72537900	-1.74906800	-0.43420900
N	2.01179900	-2.56982800	-0.62748500
O	4.44753800	1.20258000	0.17611300
H	3.52864600	1.92406800	0.41605900
H	5.09560400	0.97213400	0.88104400

3a

Sum of electronic and thermal Free Energies = -3137.967644

C	0.78966900	0.23717400	1.07547000
C	-0.56745000	-0.06278400	1.05192000
C	-1.09843100	-0.80366800	-0.00271500
C	-0.26555400	-1.27618900	-1.01493200
C	1.09566800	-1.00377200	-0.98310500
C	1.60624000	-0.23821100	0.05812500
H	1.19869100	0.82117300	1.89013400
H	-1.20676500	0.28186500	1.85712600
H	-0.68671700	-1.85365200	-1.82991800
H	1.74073800	-1.37445500	-1.76972700
Br	3.46735500	0.16126200	0.09407400

C	-3.54554800	-0.32337400	0.25914100
N	-2.48276400	-1.11563900	-0.06118000
N	-3.34659000	1.09249600	0.26992900
N	-2.49064400	1.55685900	-0.49922200
N	-1.77155200	2.09591100	-1.15983700
O	-4.64067600	-0.75136000	0.53146000
H	-2.72467300	-2.09067100	-0.20312600

VII. Mechanistic considerations

Experiments were conducted to assess potential interactions between the substrate and PhI(OAc)$_2$. The results from these experiments are shown in Figure S2. The addition of **1a** to the reaction system after 30 minutes yielded the expected product **3a**. However, we failed to capture the intermediate by conventional column chromatography methods (mainly because of PhI(N$_3$)$_2$ being unstable[32]).

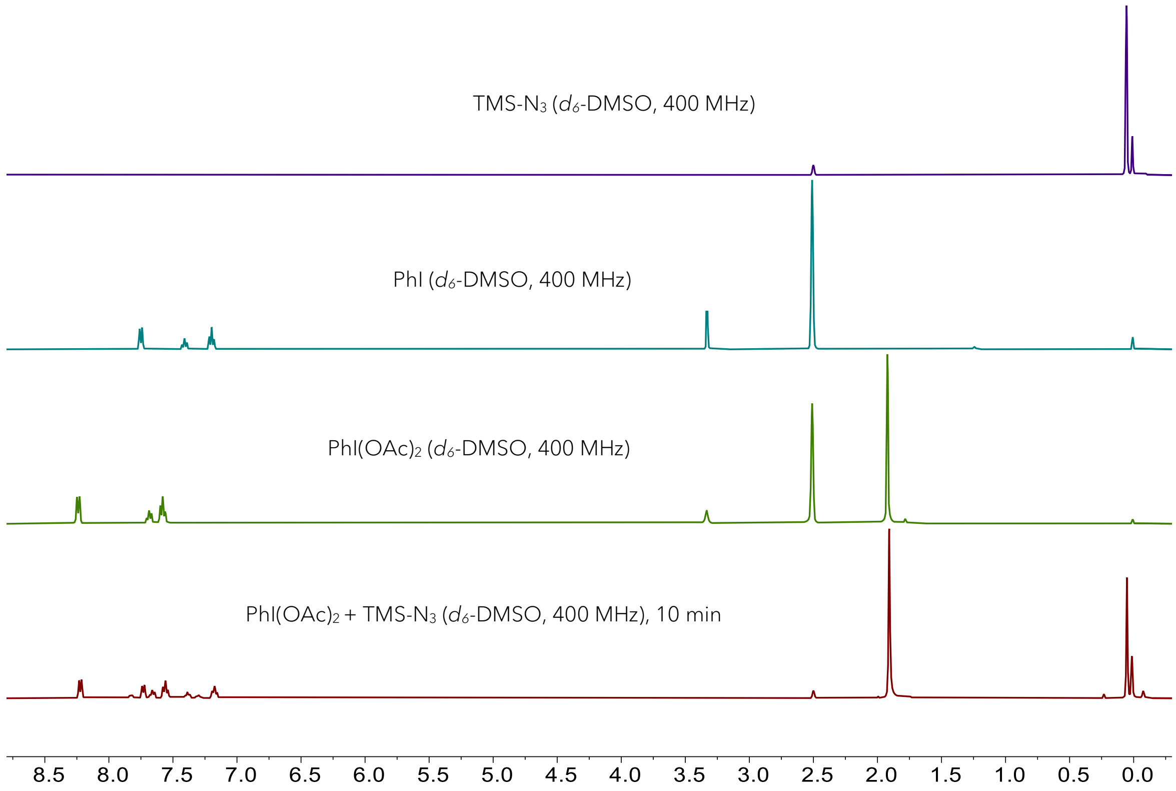

Figure S2. ^{1}H NMR (400 MHz, d_6-DMSO) of PhI, PhI(OAc)$_2$ and TMS-N$_3$.

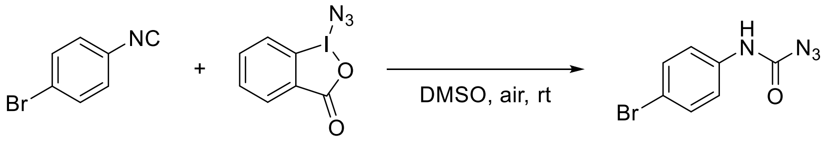

Figure S3. Control experiment with Zhdankin-type iodine(III)-N$_3$ reagent.

VIII. References

(1) González-Bobes, F.; Kopp, N.; Li, L.; Deerberg, J.; Sharma, P.; Leung, S.; Davies, M.; Bush, J.; Hamm, J.; Hrytsak, M. Scale-Up of Azide Chemistry: A Case Study. *Org. Process Res. Dev*. **2012**, *16*, 2051–2057. https://doi.org/10.1021/op3002646

(2) Yuan, X.; Qu, Y.; Li, Y.; Bao, H. Synthesis of Carbamoyl Azides from Redox-Active Esters and $TMSN_3$. *Synlett* **2023**, *35*, 464-468. https://doi.org/10.1055/a-2106-5108

(3) Feng, P.; Sun, X.; Su, Y.; Li, X.; Zhang, L. H.; Shi, X.; Jiao, N. Ceric Ammonium Nitrate (CAN) Catalyzed Modification of Ketones via Two C–C Bond Cleavages with the Retention of the Oxo-Group. *Org. Lett*. **2014**, *16*, 3388–3391. https://doi.org/10.1021/ol5014476

(4) Reddy, P. S.; Yadagiri, P.; Lumin, S.; Shin, D.-S.; Falck, J. R. Modified Pyridinium Chlorochromate Oxidation of Aldehydes to Carbamoyl Azides/Acyl Azides or Carboxylic Acids. *Synth. Commun*. **1988**, *18*, 545–551. https://doi.org/10.1080/00397918808060749

(5) Li, X.-Q.; Zhao, X.-F.; Zhang, C. Iodobenzene Dichloride in Combination with Sodium Azide for the Effective Synthesis of Carbamoyl Azides from Aldehydes. *Synthesis* **2008**, 2589–2593. https://doi.org/10.1055/s-2008-1067196

(6) Zhang, C.; Wang, W. K.; He, T. Dramatic Solvent Effect in the One-Pot Synthesis of Substituted Ureas Directly from Primary Alcohols Using the Combined Reagent of Iodobenzene Dichloride and Sodium Azide in Ethyl Acetate. *Synthesis* **2012**, *44*, 3006–3014.
https://doi.org/10.1055/s-0032-1316745

(7) Abbott Laboratories. Phenylcarbamoylazides in Reducing Blood Pressure. U.S. Patent WO US3424844A, January 28, 1969.

(8) Salama, T. A.; Elmorsy, S. S.; Khalil, A.-G. M.; Ismail, M. A. Silicon-Mediated Direct Conversion of Acyl Chlorides to Carbamoyl Azides or/and Tetrazolinones under Mild Conditions. *Chem. Lett*. **2011**, *40*, 1149–1151. https://doi.org/10.1246/cl.2011.1149

(9) Kobayashi, S.; Yamaguchi, R.; Yamamoto, F.; Komori, J.; Sakamoto, H.; Kasashima, T.; Adriaenssens, L.; Lear, M. J. One-Pot Conversion of Benzyl Alcohols to N-Protected Anilines and Alkyl Alcohols to Carbamoyl Azides. *Eur. J. Org. Chem*. **2023**, *26*, e202300786.
https://doi.org/10.1002/ejoc.202300786

(10) Panday, P.; Garg, P.; Singh, A. Manganese-Dioxide-Catalyzed Trifluoromethylation and Azidation of Styrenyl Olefins via Radical Intermediates. *Asian J. Org. Chem*. **2018**, *7*, 111–115. https://doi.org/10.1002/ajoc.201700508

(11) Prakash, O.; Pannu, K.; Prakash, R.; Batra, A. [Hydroxy(tosyloxy)iodo]benzene Mediated α-Azidation of Ketones. *Molecules* **2006**, *11*, 523–527. https://doi.org/10.3390/11070523

(12) Li, Y.-L.; Shi, Z.; Shen, T.; Ye, K.-Y. Electrochemical Vicinal Oxyazidation of α-Arylvinyl Acetates. *Beilstein J. Org. Chem*. **2022**, *18*, 1026–1031. https://doi.org/10.3762/bjoc.18.103

(13) Chandra, A.; Parida, K. N.; Moorthy, J. N. One-Pot Synthesis of α-Bromo- and α-Azidoketones from Olefins by Catalytic Oxidation with In Situ-Generated Modified IBX as the Key Reaction. *Tetrahedron* **2017**, *73*, 5827–5832. https://doi.org/10.1016/j.tet.2017.08.019

(14) Muthukrishnan, S.; Mandel, S. M.; Hackett, J. C.; Singh, P. N. D.; Hadad, C. M.; Krause, J. A.; Gudmundsdóttir, A. D. Competition between α-Cleavage and Energy Transfer in α-Azidoacetophenones. *J. Org. Chem*. **2007**, *72*, 2757–2768. https://doi.org/10.1021/jo062160k

(15) Ye, Z.; Zhu, R.; Wang, F.; Jiang, H.; Zhang, F. Electrochemical Difunctionalization of Styrenes via Chemoselective Oxo-Azidation or Oxo-Hydroxyphthalimidation. *Org. Lett*. **2021**, *23*, 8240–8245. https://doi.org/10.1021/acs.orglett.1c02991

(16) Moumné, R.; Larue, V.; Seijo, B.; Lecourt, T.; Micouin, L.; Tisné, C. Tether Influence on the Binding Properties of $tRNA^{Lys}{}_3$ Ligands Designed by a Fragment-Based Approach. *Org. Biomol. Chem*. **2010**, *8*, 1154–1159. https://doi.org/10.1039/B921232A

(17) Brenelli, E. C. S.; Brenelli, J. A.; Pinto, R. C. L. A Fast Procedure for the Preparation of Vicinal Azidoalcohols Using Polymer Supported Reagents. *Tetrahedron Lett*. **2005**, *46*, 4531–4533. https://doi.org/10.1016/j.tetlet.2005.05.023

(18) Prasad, B.; Phanindrudu, M.; Tiwari, D. K.; Kamal, A. Transition-Metal-Free One-Pot Tandem Synthesis of 3-Ketoisoquinolines from Aldehydes and Phenacyl Azides. *J. Org. Chem*. **2019**, *84*, 12334–12343. https://doi.org/10.1021/acs.joc.9b01534

(19) Okumuş, S.; Tanyeli, C.; Demir, A. S. Asymmetric Aldol Addition of α-Azido Ketones to Ethyl Pyruvate Mediated by a Cinchona-Based Bifunctional Urea Catalyst. *Tetrahedron Lett*. **2014**, *55*, 4302–4305. https://doi.org/10.1016/j.tetlet.2014.06.018

(20) Bangalore, P. K.; Vagolu, S. K.; Bollikanda, R. K.; Veeragoni, D. K.; Choudante, P. C.; Misra, S.; Sriram, D.; Sridhar, B.; Kantevari, S. Usnic Acid Enaminone-Coupled 1,2,3-Triazoles as Antibacterial and Antitubercular Agents. *J. Nat. Prod*. **2020**, *83*, 26–35. https://doi.org/10.1021/acs.jnatprod.9b00475

(21) Hussain, M. I.; Feng, Y.; Hu, L.; Deng, Q.; Zhang, X.; Xiong, Y. Copper-Catalyzed Oxidative Difunctionalization of Terminal Unactivated Alkenes. *J. Org. Chem*. **2018**, *83*, 7852–7859. https://doi.org/10.1021/acs.joc.8b00729

(22) Takeuchi, H.; Yanagida, S.; Ozaki, T.; Hagiwara, S.; Eguchi, S. Synthesis of Novel Carbo- and Heteropolycycles. 12. A New Versatile Synthesis of Oxazoles by Intramolecular Aza-Wittig Reaction. *J. Org. Chem.* **1989**, *54*, 431–434. https://doi.org/10.1021/jo00263a033

(23) Frisch, M. J.; Trucks, G. W.; Schlegel, H. B.; Scuseria, G. E.; Robb, M. A.; Cheeseman, J. R.; Scalmani, G.; Barone, V.; Petersson, G. A.; Nakatsuji, H.; Li, X.; Caricato, M.; Marenich, A. V.; Bloino, J.; Janesko, B. G.; Gomperts, R.; Mennucci, B.; Hratchian, H. P.; Ortiz, J. V.; Izmaylov, A. F.; Sonnenberg, J. L.; Williams, Ding, F.; Lipparini, F.; Egidi, F.; Goings, J.; Peng, B.; Petrone, A.; Henderson, T.; Ranasinghe, D.; Zakrzewski, V. G.; Gao, J.; Rega, N.; Zheng, G.; Liang, W.; Hada, M.; Ehara, M.; Toyota, K.; Fukuda, R.; Hasegawa, J.; Ishida, M.; Nakajima, T.; Honda, Y.; Kitao, O.; Nakai, H.; Vreven, T.; Throssell, K.; Montgomery Jr., J. A.; Peralta, J. E.; Ogliaro, F.; Bearpark, M. J.; Heyd, J. J.; Brothers, E. N.; Kudin, K. N.; Staroverov, V. N.; Keith, T. A.; Kobayashi, R.; Normand, J.; Raghavachari, K.; Rendell, A. P.; Burant, J. C.; Iyengar, S. S.; Tomasi, J.; Cossi, M.; Millam, J. M.; Klene, M.; Adamo, C.; Cammi, R.; Ochterski, J. W.; Martin, R. L.; Morokuma, K.; Farkas, O.; Foresman, J. B.; Fox, D. J. Gaussian 16, Revision B.01; Gaussian, Inc.: Wallingford, CT, 2016.

(24) Zhao, Y.; Truhlar, D. G. The M06 suite of density functionals for main group thermochemistry, thermochemical kinetics, noncovalent interactions, excited states, and transition elements: two new functionals and systematic testing of four M06-class functionals and 12 other functionals. *Theor. Chem. Acc*. **2008**, *120*, 215–241. https://doi.org/10.1007/s00214-007-0310-x

(25) Becke, A. D. Density-functional exchange-energy approximation with correct asymptotic behavior. *Phys. Rev. A.* **1988**, *38*, 3098–3100. https://doi.org/10.1103/PhysRevA.38.3098

(26) Becke, A. D. Density‐functional thermochemistry. III. The role of exact exchange. *J. Chem. Phys*. **1993**, *98*, 5648–5652. https://doi.org/10.1063/1.464913

(27) Lee, C.; Yang, W.; Parr, R. G. Development of the Colle-Salvetti correlation-energy formula into a functional of the electron density. *Phys. Rev. B.* **1988**, *37*, 785–789. https://doi.org/10.1103/PhysRevB.37.785

(28) Marenich, A. V.; Cramer, C. J.; Truhlar, D. G. Performance of SM6, SM8, and SMD on the SAMPL1 Test Set for the Prediction of Small-Molecule Solvation Free Energies†. *J. Phys. Chem. B.* **2009**, *113*, 4538–4543. https://doi.org/10.1021/jp809094y

(29) Fukui, K. The path of chemical reactions - the IRC approach. *Acc. Chem. Res.* **1981**, *14*, 363–368. https://doi.org/10.1021/ar00072a001

(30) Gonzalez, C.; Schlegel, B. An improved algorithm for reaction path following. *J. Chem. Phys.* **1989**, *90*, 2154–2161. https://doi.org/10.1063/1.456010

(31) Legault, C. Y. CYLview, 1.0b; Université de Sherbrooke: Canada, 2009. Available at: http://www.cylview.org (accessed September, 2014).

(32)Yoshimura, A.; Zhdankin, V. V. Advances in Synthetic Applications of Hypervalent Iodine Compounds. *Chem. Rev*. **2016**, *116*, 3328–3435. https://pubs.acs.org/doi/10.1021/acs.chemrev.5b00547
